# Pd‐Catalyzed Asymmetric N‐Allylation of Amino Acid Esters with Exceptional Levels of Catalyst Control: Stereo‐Divergent Synthesis of ProM‐15 and Related Bicyclic Dipeptide Mimetics

**DOI:** 10.1002/chem.202000307

**Published:** 2020-02-18

**Authors:** Stephan Dohmen, Martin Reiher, Dominik Albat, Sema Akyol, Matthias Barone, Jörg‐Martin Neudörfl, Ronald Kühne, Hans‐Günther Schmalz

**Affiliations:** ^1^ Department of Chemistry University of Cologne Greinstrasse 4 50939 Köln Germany; ^2^ Leibniz-Institut für Molekulare Pharmakologie (FMP) 13125 Berlin Germany

**Keywords:** asymmetric catalysis, chiral diphosphine ligands, peptide mimetics, protein interactions, transition-metal catalysis

## Abstract

A general and powerful method for the stereo‐controlled Pd‐catalyzed N‐allylation of amino acid esters is reported, as a previously largely unsolved synthetic challenge. Employing a new class of tartaric acid‐derived *C*_2_‐symmetric chiral diphosphane ligands the developed asymmetric amination protocol allows the conversion of various amino acid esters to the N‐allylated products with highest levels of enantio‐ or diastereoselectivity in a fully catalyst‐controlled fashion and predictable configuration. Remarkably, the in situ generated catalysts also exhibit outstanding levels of activity (ligand acceleration). The usefulness of the method was demonstrated in the stereo‐divergent synthesis of a set of new conformationally defined dipeptide mimetics, which represent new modular building blocks for the development of peptide‐inspired bioactive compounds.

## Graphical abstract

**Identification of a powerful** ligand for the catalytic asymmetric N‐allylation of amino acid esters paved the way for a short and fully stereo‐controlled access to new dipeptide building blocks with a defined 3D structure (see scheme).

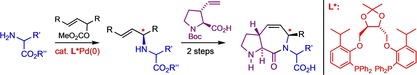

In the course of our research into the inhibition of PPII helix‐mediated protein–protein interactions, we had designed and synthesized proline‐derived modules, such as **ProM‐1** 1 and **ProM‐2**.^2^ This enabled us to develop a powerful inhibitor of the ena/VASP EVH1 domains involved in cell migration and chemotaxis (Figure 1).^3^

**Figure 1:**
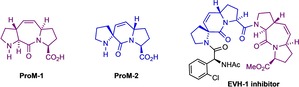
Proline‐derived modules **ProM‐1** and **ProM‐2** and their combined appearance in a synthetic small‐molecule EVH‐1 inhibitor.

More recently, modeling studies suggested that compounds of type **1** (including **ProM‐15**, formally derived from **ProM‐1** by opening the eastern proline ring) would represent promising building blocks for a new generation of EVH1 inhibitors, due to an enhanced flexibility of the C‐terminus in combination with the option to address additional lipophilic or polar interaction sites at the protein surface by means of the substituent R’ (Scheme 1).

**Scheme 1:**
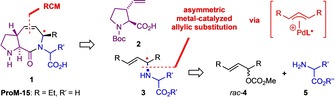
Design and retrosynthetic analysis of **ProM‐15** and related dipeptide analogues exploiting asymmetric N‐allylation of amino acid esters as a key step.

Following our established strategy, we intended to assemble such compounds from the known 3‐vinylproline derivative **2** (Zaminer's acid)^4^ and an allylamine **3** through peptide coupling and subsequent ring‐closing metathesis. Building blocks of type **3** in turn could be prepared by stereo‐controlled Pd‐catalyzed N‐allylation of an amino acid ester **5** by using a racemic carbonate of type *rac*‐**4** (Scheme 1). The Pd‐catalyzed asymmetric allylic substitution, that is, Tsuji–Trost reaction proceeding via pseudo‐symmetric (*meso*‐type) π‐allyl‐Pd intermediates carrying chiral ligands, has been intensively studied.^5^ However, while a number of useful protocols exist for Pd‐catalyzed^6^ (and Ir‐catalyzed^7^) enantioselective allylic aminations, we were surprised to learn that only few (and little convincing) examples have been reported for the asymmetric N‐allylation of amino acid esters, despite them representing a well‐accessible and highly relevant class of N‐nucleophiles.7c, 8, 9, 10 Therefore, we were challenged to develop an efficient methodology for such reactions, which we disclose herein.

Having **ProM‐15** (R=Et; R’=H) as a target structure in mind, we commenced our study by investigating the N‐allylation of *tert*‐butyl glycinate (**5 a**) employing the racemic carbonate *rac*‐**4 a** (Table 1). Initial experiments using dppe as a ligand under the conditions of Williams10a unexpectedly led to the formation of carbamate products.^11^ However, this phenomenon could successfully be suppressed by increasing the concentration to 10 mol L^−1^. In this case, we observed a complete and clean conversion of *rac*‐**4 a** after 5.5 hours at room temperature, and the product *rac*‐**3 a** was isolated in 78 % yield. This material was used as a racemic reference sample to establish reliable conditions for the enantiomeric analysis by means of GC by using a chiral stationary phase. As a most prominent chiral ligand, we first tested the commercial Trost ligand **L1** (Figure 2).^12^ However, the enantioselectivity was unsatisfactory (e.r.≤83:17) even upon lowering the temperature to −10 °C (entries 2–4). After screening a variety of other chiral ligands (see Table SI‐1 in the Supporting Information), we were pleased to find that some of the *C*_2_‐symmetric chiral diphosphine ligands **L2**–**L8**, recently developed in our laboratory,^13^ gave superior results (Figure 2).

**Table 1:** Optimizing the asymmetric N‐allylation of **5 a**.^[a]^

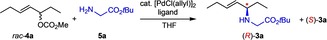							
Entry	Ligand	Pd/L [mol %]	Conc.^[b]^ [m]	*T* [°C]	*t* [h]	Conv.^[c]^ [%]	e.r. ^[d]^ [*S*/*R*]
1	dppe	2.5:6	10	RT	5.5	100	–
2	**L1**	2.5:6	10	RT	5	100	27:73
3	**L1**	2.5:6	10	0	22	100	19:81
4	**L1**	2.5:6	10	−10	20	75	17:83
5	**L2**	2.5:6	10	0	22	91	73:27
6	**L3**	2.5:6	10	0	2.5	100	90:10
7	**L4**	2.5:6	10	0	2.5	100	94:6
8	**L5**	2.5:6	10	0	2.5	100	91:9
9	**L6**	2.5:6	10	0	2.5	100	96:4
10	**L7**	2.5:6	10	0	2.5	100	96:4
11	**L8**	2.5:6	10	0	2.5	100	90:10
12	**L4**	1:2.4	10	−10	2.5	100	96:4
13	**L4**	0.5:1.2	10	−10	5	100	96:4
14	**L6**	1:2.4	10	−10	2.5	100	97:3
15	**L7**	1:2.4	10	−10	2.5	100	97:3
16	**L6**	1:2.4	5	−10	2.5	100	96:4
17	**L6**	1:2.4	2.5	−10	2.5	100	98:2
18	**L6**	1:2.4	1.25	−10	5	100	98:2
19	**L1**	1:2.4	2.5	−10	22	5	–

[a] Reactions were performed on a 1 mmol scale by using **5 a** (2 equiv). [b] Concentration of *rac*‐**4 a**. [c] Conversion was determined by means of GC. [d] Enantiomeric ratio was determined by means of GC using a chiral stationary phase; configurational assignments are based on the X‐ray crystal structure analysis of the **ProM‐15** derivative **7 a**; dppe=1,2‐bis(diphenylphosphino)ethane.

**Figure 2:**
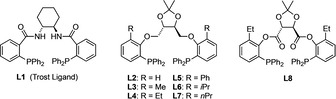
Chiral ligands used in this study (see Table 1).

In particular, ligands **L6** and **L7** gave respectable initial enantioselectivities of 96:4 e.r. at 0 °C (Table 1, entries 9 and 10). Remarkably, these ligands also exhibited a substantial ligand acceleration allowing the reactions to rapidly take place also at lower temperatures and catalyst loadings (entries 12 to 18). Under optimized conditions by using only 1 mol % of [PdCl(allyl)]_2_ as a pre‐catalyst and 2.4 mol % of ligand **L6**, the reaction proceeded smoothly at −10 °C within only 2.5 hours to afford (*S*)‐**3 a** with high enantiomeric purity (98:2 e.r.) in 83 % isolated yield (entry 17).

To probe the applicability of the developed protocol, the carbonate *rac*‐**4 a** was reacted with a set of different (l)‐amino acid esters (*S*)‐**5** employing **L6** as a chiral ligand. The results summarized in Scheme 2 show that outstanding stereoselectivities and isolated yields were observed in all cases, independent of the amino acid sidechain and the nature of the ester group. Even methyl prolinate, as a secondary amine, was N‐allylated with high diastereoselectivity.

**Scheme 2:**
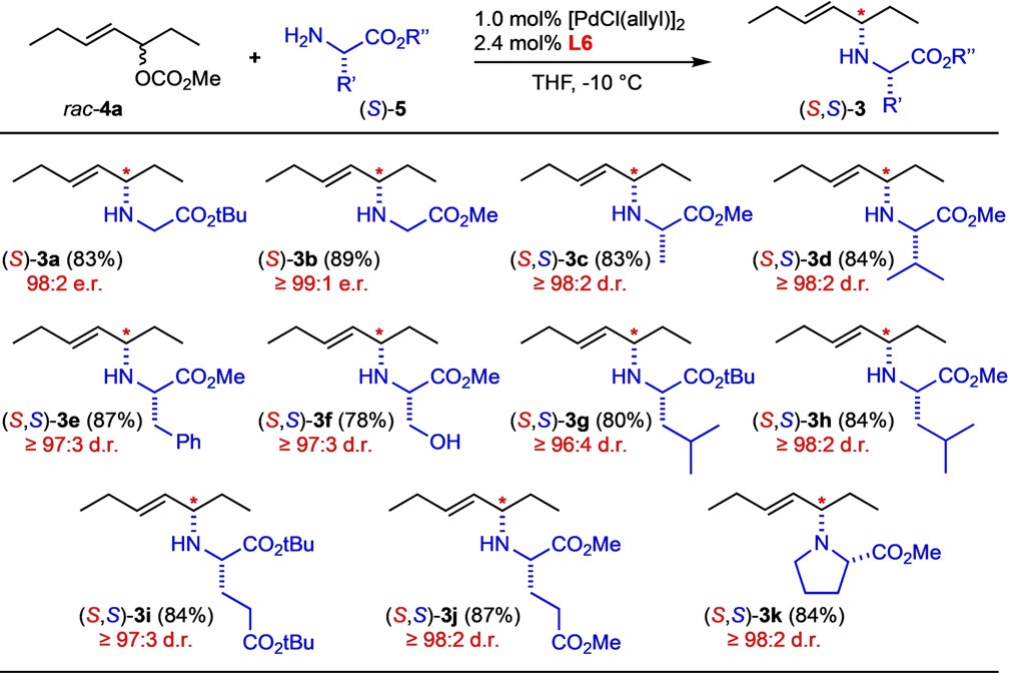
Asymmetric N‐allylation of different l‐amino acid esters employing the chiral catalyst **L6**. Diastereoselectivity values based on NMR of the crude product.

Using the enantiomeric ligand *ent*‐**L6**, the same set of (l)‐amino acid esters (*S*)‐**5** was reacted with *rac*‐**4 a** under the standard conditions (Scheme 3). And again, the products were obtained with exceedingly high stereoselectivity (≥98:2 in most cases). Actually, the minor diastereomer could often not even be detected in the crude product mixture or was chromatographically easily separated off to yield the analytically pure stereoisomers in all cases. The results summarized in Schemes 2 and Scheme 3 demonstrate the outstanding level of catalyst control with only marginal matched/mismatched effects.^14^

**Scheme 3:**
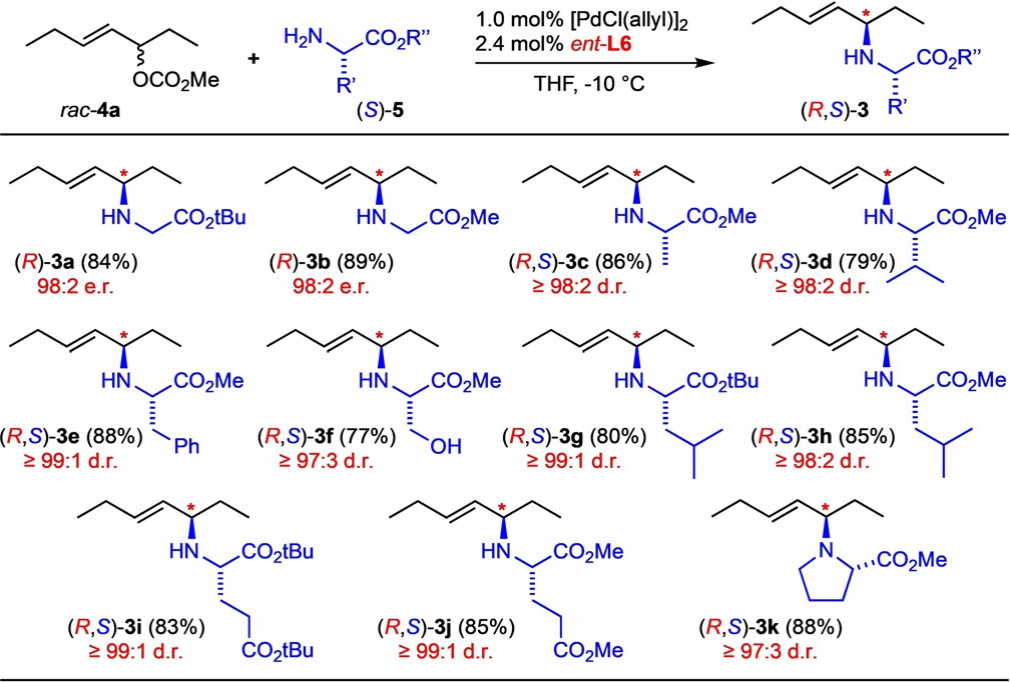
Asymmetric N‐allylation of different l‐amino acid esters employing the chiral catalyst *ent*‐**L6**. Diastereoselectivity values based on NMR of the crude product.

Having successfully elaborated a reliable and broadly applicable protocol for the ligand‐controlled asymmetric N‐allylation of various amino acid esters, we turned our attention back to the original goal, that is, the synthesis of the **ProM‐15** derivative **7 a** and related bicyclic dipeptides (compare Scheme 1). Not completely unexpected, coupling of the 3‐vinylproline building block **2** with the secondary amine **3 a** proved to be very difficult and could not be achieved employing common peptide coupling reagents (such as HATU or PyBOP)^15^ due to steric reasons. However, after considerable experimentation (Table SI‐3 in the Supporting Information), we found that the desired transformation could be efficiently achieved by using Ghosez's reagent (**8**)^16^ to convert the acid **2** into the corresponding acid chloride prior to addition of the amine **3 a** (98:2 e.r.) and DIPEA as a base (Scheme 4). The metathesis cyclization of the resulting dipeptide **6 a** was then best achieved using the Hoveyda–Grubbs II catalyst (**9**)^17^ to give stereochemically pure **7 a** as a crystalline product in 70 % overall yield from **2**.

**Scheme 4:**
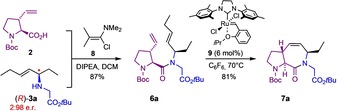
Synthesis of the **ProM‐15** derivative **7 a** under optimized conditions.

By proving the structure of **7 a** through X‐ray crystallography (Figure 3, left), we could unambiguously confirm the absolute configuration of the glycine derivative **3 a** prepared by enantioselective N‐allylation (compare Table 1).

**Figure 3:**
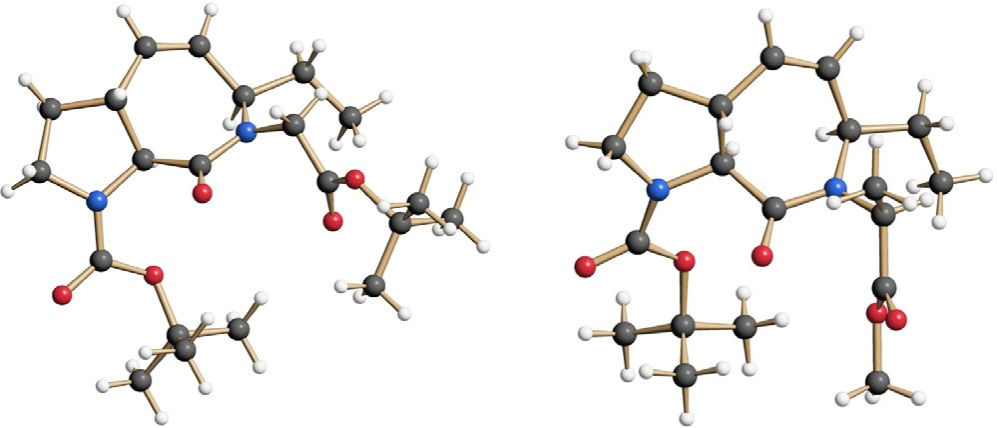
Structure of Boc‐[**ProM‐15**]‐O*t*Bu (**7 a**; left) and the corresponding alanine‐derived compound **7 c** (right) in the crystalline state.^18^

The power of the modular concept was further demonstrated in the stereo‐divergent synthesis of all four diastereomers of the phenylalanine‐derived **ProM‐15** analogues of type **7 b**, in this case employing the enantiomeric proline building block *ent*‐**2 b** (Scheme 5). Starting from either (*R*)‐ or (*S*)‐phenylalanine methyl ester (**5 b**), the stereochemically pure amines **3 e** were obtained by asymmetric N‐allylation and chromatography. Peptide coupling with *ent*‐**2 b** and subsequent ring‐closing metathesis under the developed conditions then afforded the diastereomeric dipeptide modules **7 b**. Noteworthy, the following ring‐closing metathesis step proceeded much slower in the case of the (*S,R,R,S*)‐diastereomer indicating an unfavorable conformational preorganization of this substrate. In all cases, the bicyclic target products were obtained as crystalline compounds. X‐ray crystallographic analysis confirmed their configuration and revealed the individual conformational preferences of all four diastereomers (Scheme 5).

**Scheme 5:**
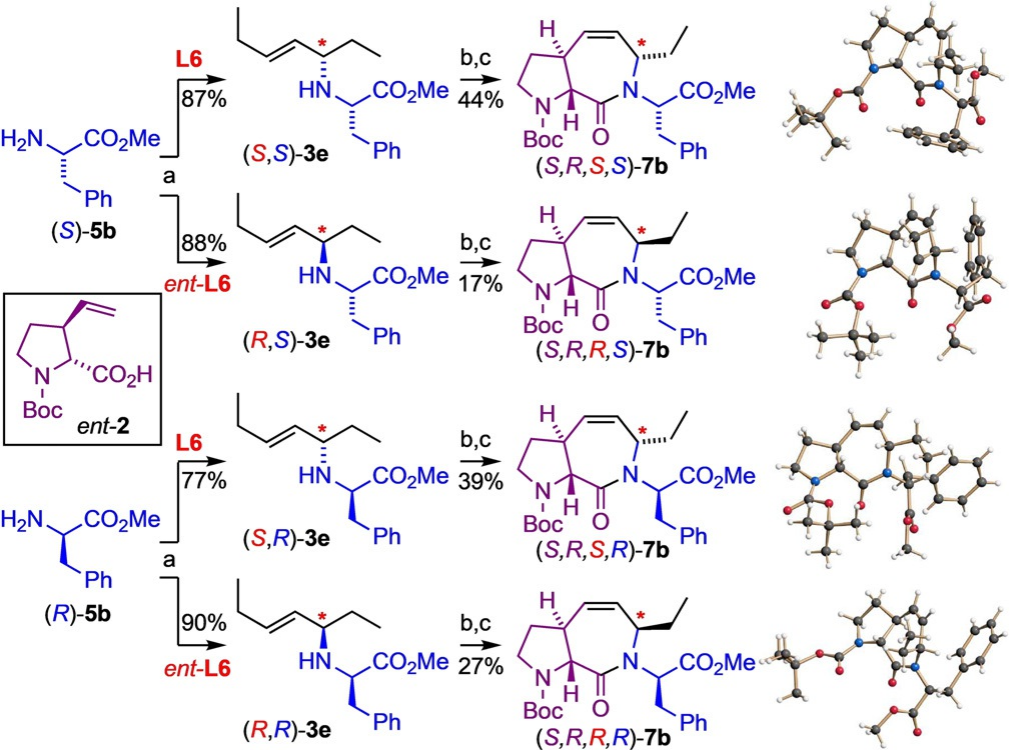
Stereo‐divergent synthesis of all four diastereomers of **7 b** derived from the 3‐vinylproline building block *ent*‐**2** under standard conditions and their structure in the crystalline state.^18^ Reagents and conditions: a) *rac*‐**4 a**, [PdCl(allyl)]_2_ (1.0 mol %), **L6** or *ent*‐**L6** (2.4 mol %), THF, −10 °C, 22 h, purification by chromatography; b) *ent*‐**2**, Ghosez reagent, 0 °C, DIPEA; c) Hoveyda–Grubbs II (6 mol %), C_6_F_6_, 70 °C, 24 h.

In a related fashion, the alanine‐derived **ProM‐15** analogue **7 c** was prepared and characterized by X‐ray crystallography to further prove the generality of the concept and to confirm the absolute configuration of the N‐allylated alanine derivative **3 c** (Scheme 6 and Figure 3, right). In this case, we started from the racemic acid building block *rac*‐**2** and exploited the facile chromatographic separation of the diastereomeric coupling products to obtain **6 c** as a single stereoisomer, which again smoothly cyclized to give **7 c**.

**Scheme 6:**
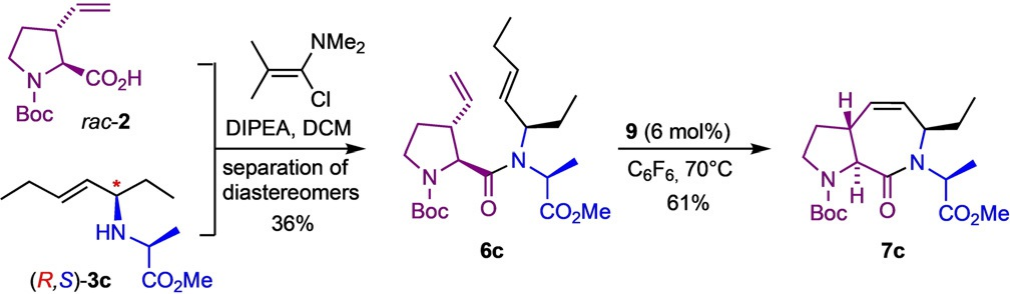
Synthesis of the **ProM‐15** derivative **7 c** starting from *rac*‐**2**.

Finally, the chiral substituted *ϵ*‐caprolactam derivatives **11 a** and **e** were prepared in high yield from the amine building blocks (*R*)‐**3 a** and (*R*,*S*)‐**3 e**, respectively, and 4‐pentenoic acid (**10**; Scheme 7).

**Scheme 7:**
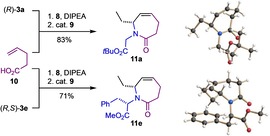
Synthesis of configurationally defined ϵ‐caprolactam derivatives **11 a** and **e** from 4‐pentenoic acid (**10**).^18^

The absolute configuration and the conformational preferences of the tetrahydroazepin‐2‐one products (*ϵ*‐lactams) **11 a** and **e** were determined once again by X‐ray crystallography.

In conclusion, we have developed a first reliable and general protocol for the asymmetric Pd‐catalyzed N‐allylation of amino acid esters. The in situ generated catalysts, derived from a previously unrecognized class of readily accessible *C*_2_‐symmetric diphosphane ligands, were found to exhibit high activities and outstanding levels of enantio‐ or diastereocontrol giving the products with predictable (catalyst‐controlled) configuration. The value of the method was demonstrated in the stereo‐divergent synthesis of **ProM‐15** and related cyclic, proline‐derived dipeptide mimetics, which represent attractive building blocks for the development of modular peptide‐inspired bioactive molecules with a defined three‐dimensional structure. Current investigations in our laboratory are focused on the further exploration of the developed N‐allylation protocol with respect to mechanistic aspects, the evaluation of the substrate scope and applications in natural‐product synthesis. Moreover, we are currently expanding our ProM toolbox^1^, ^2^, ^3^, ^19^ with further **ProM‐15**‐related compounds, which are then incorporated as modules into PPII‐helix secondary structure mimetics acting as inhibitors of disease‐relevant protein–interactions.

## Conflict of interest

The authors declare no conflict of interest.

## Supporting information

As a service to our authors and readers, this journal provides supporting information supplied by the authors. Such materials are peer reviewed and may be re‐organized for online delivery, but are not copy‐edited or typeset. Technical support issues arising from supporting information (other than missing files) should be addressed to the authors.

Supplementary
