# Supplementary material for: Pd‐Catalyzed Asymmetric N‐Allylation of Amino Acid Esters with Exceptional Levels of Catalyst Control: Stereo‐Divergent Synthesis of ProM‐15 and Related Bicyclic Dipeptide Mimetics
Source: Chemistry. 2020 Feb 18;26(14):3049–53. doi: 10.1002/chem.202000307 (PMC7078984; doi:10.1002/chem.202000307)
Supplement: Supplementary file 1 — Supplementary [file CHEM-26-3049-s001.pdf]

CHEMISTRY
A European Journal

Supporting Information

Pd-Catalyzed Asymmetric N-Allylation of Amino Acid Esters with Exceptional Levels of Catalyst Control: Stereo-Divergent Synthesis of ProM-15 and Related Bicyclic Dipeptide Mimetics

Stephan Dohmen,[a] Martin Reiher,[a] Dominik Albat,[a] Sema Akyol,[a] Matthias Barone,[b] Jörg-Martin Neudörfl,[a] Ronald Kühne,[b] and Hans-Günther Schmalz*[a]

chem_202000307_sm_miscellaneous_information.pdf

Table of Contents

Asymmetric N-allylation: Ligand Screening 3

Asymmetric N-allylation: Reaction conditions optimization 4

Optimization of the peptide coupling between trans-*N*-Boc-3-vinyl-proline (2) and N-allylated amino esters (3) 5

General information 6

Experimental procedures 7

Synthesis of carbonate *rac*-4a[1–3] 7

General procedure 1: Asymmetric Pd-catalyzed N-allylation amino acid esters 8

General procedure 2: Peptide coupling using *Ghosez* reagent 26

General procedure 3: Ru-catalyzed ring closing metathesis 26

NMR Spectra 50

GC Spectra of *rac*-3a and (*S*)-3a 88

References 89

Author Contributions 97

Asymmetric *N*-allylation: Ligand Screening

Table S1. Screening of various chiral ligands under standard conditions

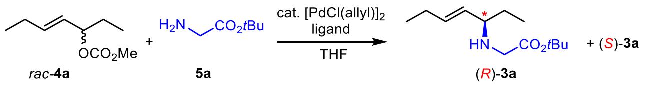

Entry	Ligand	Pd/L[a] [mol%]	Conc [M]	Temp [°C]	Time [h]	Conv.[b] [%]	e.r.[c] [*S*:*R*]
1	dppe	2.5/6	10	r.t.	5.5	100	--
2	**S-L1**	2.5/6	10	0	22	0	--
3	**S-L2**	2.5/6	10	0	22	6	--
4	**S-L3**	2.5/6	10	0	22	90	41:59
5	**S-L4**	2.5/6	10	0	22	57	89:11
6	**S-L5**	2.5/6	10	0	22	100	77:23
7	**S-L6**	2.5/6	10	0	22	0	--
8	**S-L7**	2.5/6	10	0	69	20	39:61
9	**S-L8**	2.5/6	10	0	69	100	41:59
10	**S-L9**	2.5/6	10	0	2.5	100	89:11
11	**S-L10**	2.5/6	10	0	2.5	100	92:8
12	**S-L11**	2.5/6	10	0	2.5	100	56:44
13	**L1**	2.5/6	10	r.t.	5	100	27:73
14	**L1**	2.5/6	10	0	22	100	19:81
15	**L1**	2.5/6	10	-10	20	75	17:83
16	**L2**	2.5/6	10	0	22	91	73:27
17	**L3**	2.5/6	10	0	2.5	100	90:10
18	**L4**	2.5/6	10	0	2.5	100	94:6
19	**L5**	2.5/6	10	0	2.5	100	91:9
20	**L6**	2.5/6	10	0	2.5	100	96:4
21	**L7**	2.5/6	10	0	2.5	100	96:4
22	**L8**	2.5/6	10	0	2.5	100	90:10

[a] Reactions were performed on a 1 mmol scale using 2 equiv. of **5a.** [b] concentration of *rac*-**4a**; [c] the conversion was determined by means of GC; [d] The enantiomeric ratio was determined by means of GC using a chiral stationary phase; configurational assignments are based on the X-ray crystal structure analysis of the **ProM-15** derivative **7a**.

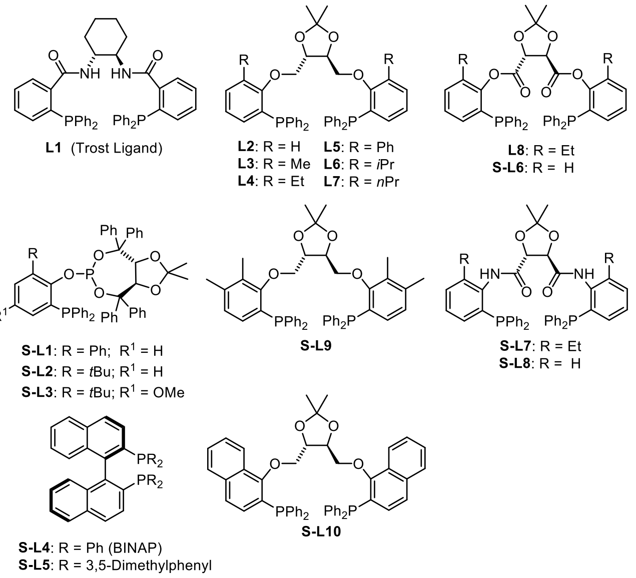

Asymmetric *N*-allylation: Reaction conditions optimization

Table S2. Screening of various conditions using diphosphine ligands **L1**, **L4-7**

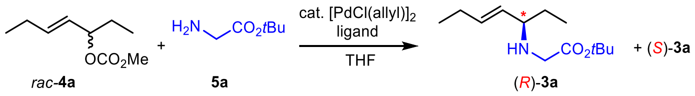

Entry	Ligand	Pd/L[a] [mol%]	Conc [M]	Temp [°C]	Time [h]	Conv.[b] [%]	e.r. [c] [*S*:*R*]
1	**L4**	1/2.4	10	-10	2.5	100	96:4
2	**L4**	0.5/1.2	10	-10	5	100	96:4
3	**L6**	1/2.4	10	-10	2.5	100	97:3
4	**L7**	1/2.4	10	-10	2.5	100	97:3
5	**L6**	1/2.4	5	-10	2.5	100	96:4
6	**L6**	1/2.4	2.5	-10	2.5	100	98:2
7	**L6**	1/2.4	1.25	-10	5	100	98:2
8	**L1**	2.5/6	10	r.t.	5	100	27:73
9	**L1**	2.5/6	10	-10	20	75	17:83
10	**L1**	1/2.4	2.5	-10	22	5	--
11	**L4**	1/2.4	25	-10	2.5	100	94:6
12	**L4**	1/2.4	10	-20	5	100	96:4
14	**L4**	1/2.4	10	-30	21.5	100	97:3
15	**L4**	1/2.4	1.25	-10	5	100	98:2
16	**L4**	0.25/0.6	10	-10	>5	100	96:4

[a] Reactions were performed on a 1 mmol scale using 2 equiv. of **5a.** [b] concentration of *rac*-**4a**; [c] the conversion was determined by means of GC; [d] The enantiomeric ratio was determined by means of GC using a chiral stationary phase; configurational assignments are based on the X-ray crystal structure analysis of the **ProM-15** derivative **7a**.

Optimization of the peptide coupling between trans-*N*-Boc-3-vinyl-proline (2) and *N*-allylated amino esters (3)

While the glycine derivatives *rac*-**3a** and 3**b** afforded the expected product (**6**, R = H) using either PyBOP in acetonitrile or HATU in NMP as a solvent in the presence of DIPEA (Table S2, entries 1 and 2) the sterically more bulky amines (such as **3e**) derived from other amino acids required the search for more powerful coupling conditions.

Table S3. Searching for peptide coupling conditions of the acid **2** with *N*-allylated amino acid esters of type **3**.

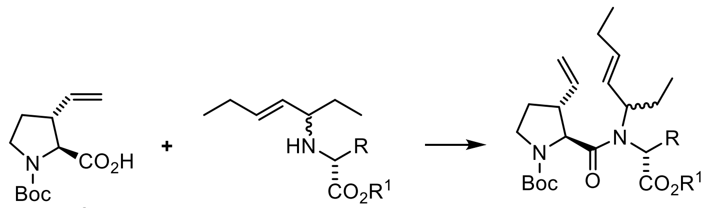

2 **3** **6**

Entry	Amine	reagent	base	Temp [°C]	solvent	yield [%]
1	*rac*-**3a**	PyBOP	DIPEA	60	MeCN	60
2	*rac*-**3a**	HATU	DIPEA	85	NMP	71
3	*ambo*-**3c**	HATU	DIPEA	85	NMP	--
4	*ambo*-**3e**	HATU	DIPEA	85	NMP	--
5	*ambo*-**3e**	Pfp-OH	--	RT	CH_2Cl_2	--
6	*ambo*-**3e**	PyCloP	DIPEA	RT – 45	CH_2Cl_2	12
7	*ambo*-**3e**	PyCloP	DIPEA	RT – 80	DMF	--
8	*ambo*-**3e**	EDC/DMAP	--	0 – 50	CH_2Cl_2	--
9	*ambo*-**3e**	BTFFH	DIPEA	0 – 85	DMF	--
10	*ambo*-**3e**	BTFFH	Pyridin	0 - RT	DMF	--
11	*ambo*-**3e**	cyanuric fluoride	DIPEA	RT	CH_2Cl_2	--
12	*ambo*-**3e**	*Ghosez* reagent	DIPEA	0 - RT	CH_2Cl_2	66
13	*rac*-**3a**	*Ghosez* reagent	DIPEA	0 - RT	CH_2Cl_2	87

Reactions were generally performed on a 0.3 (±0.05) mmol scale. Yields refer to the purified product after chromatography.

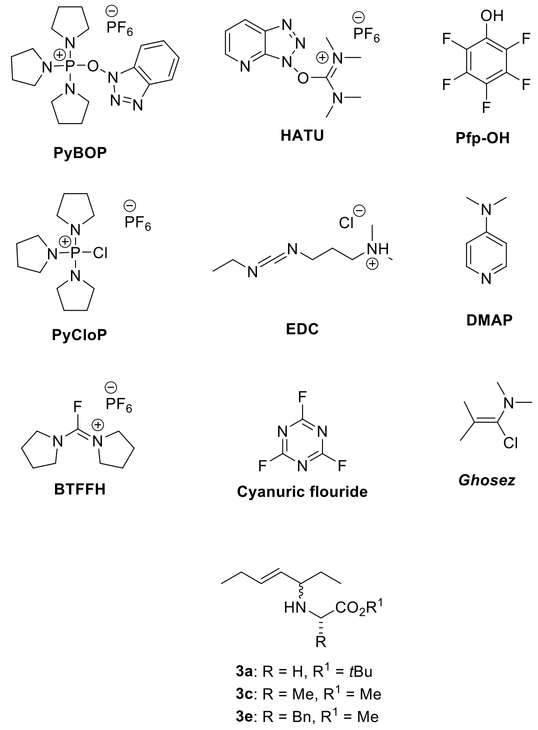

General information

All moisture sensitive reactions were carried out under argon atmosphere using Schlenk technique. Glassware was flame-dried under vacuum (<1 mbar) and allowed to cool down under argon atmosphere. Syringes, needles and transfer cannulas were dried in an oven at 100 °C and were flushed with argon directly prior to use. Flash chromatography was performed using silica 60 (0.035 – 0.07 mm) supplied by *Acros*.

NMR spectroscopy: ^{1}H and ^{13}C NMR spectra were recorded on *Bruker* AV 400, *Bruker* AV 300, or *Bruker* DPX 300 instruments. Chemical shifts (δ) are given in ppm relative to the solvent reference as an internal standard (^{1}H NMR: δ 7.26 ppm for $CDCl_3$ and δ 3.31 ppm for CD_3OD; ^{13}C NMR: δ 77.16 ppm for $CDCl_3$; δ 49.00 ppm for CD_3OD). The assignments of ^{1}H NMR are supported by H,H-COSY, HMQC(HSQC), and HMBC spectra. Carbon multiplicity assignment is based on APT or DEPT spectra. *Fourier transform infrared spectroscopy* (FT-IR): IR spectra were recorded on a *Perkin Elmer* FT-IR Paragon 1000 spectrometer using Fourier transform infrared (FTIR) multiple-point attenuated total reflection (ATR) technique. Absorption bands are given in wave numbers (ṽ, cm^{-1}). Intensive bands are marked with (s), medium with (m), weak with (w). Broad bands are marked as (br).

Mass spectrometry: Mass spectra (ESI) were recorded on a *Thermo Fischer* LTQ Orbitrap XL – FTMS Analyser.

GC-MS experiments were carried out on *Agilent* 6890 system with mass detector (MSD) 5937 N. Separation was accomplished using an *Optima*-5 *Accent* column by *Macherey-Nagel*. For the detection TIC as well as FID was used. Hydrogen was used as carrier gas with a flow of 1.7 ml/min. The column temperature was first hold at 50 °C for 2 min and then increased to 300 °C at 25 °C/min.

For the determination of enantiomeric ratios an *Agilent* 6890 system with FID detection was used. The separation was performed using a *CP-Chiral-Dex CB* column by *Varian*. The carrier gas was nitrogen with a flow of 0.9 ml/min. Alternatively a *Hewlett Packard* 6890 system with FID detection was used with hydrogen as carrier gas and a flow of 1.5 ml/min. The used temperature programs are specified in the analytical data of the respective substance as well as the used column.

Experimental procedures

Synthesis of carbonate *rac*-4a[1–3]

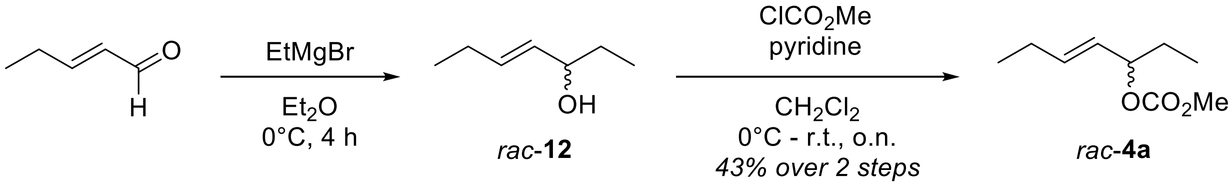

To a solution of 5.0 ml (51.12 mmol, 1.0 eq.) *trans*-2-pentenal in 75 ml abs. Et_2O 3.0 ml (66.45 mmol, 1.3 eq.) ethylmagnesium bromide were added slowly at 0°C. The reaction mixture was stirred at the same temperature for 2 h followed by the addition of 75 ml saturated NH_4Cl-solution. The phases were separated, and the aqueous layer was extracted three times with Et_2O. Combined organic layers were dried over $MgSO_4$ and the solvent was evaporated under reduced pressure. **Due to the volatility of the product the pressure should not be reduced below 200 mbar** (at 40°C water bath temperature). The yellow raw product was purified by flash column chromatography ($CH_2Cl_2/CHCl_3$ = 10/1). The resulting alcohol *rac*-**12** was solved in 104 ml abs. CH_2Cl_2 and cooled to 0°C. At this temperature 12.2 ml (150.81 mmol, 3.0 eq.) pyridine were added over 1 h followed by one additional hour of stirring at 0°C. Subsequently 7.8 ml (100.54 mmol, 2.0 eq.) methyl chloroformate was added over 1 h. After complete addition the ice bath was removed, and the reaction was stirred over night at room temperature. Afterwards saturated NaCl-solution was added, and the phases separated. The aqueous layer was diluted with H_2O until the precipitation vanished completely and then extracted three times with CH_2Cl_2. The combined organic phases were dried over $MgSO_4$ and the solvent was evaporated under reduced pressure. Due to the volatility of the carbonate pressure was not reduced below 400 mbar (at 40°C water bath temperature). The purification was performed using bulb tube distillation yielding 3.80 g (22.06 mmol, 43% over 2 steps) of the desired product as a colorless liquid.

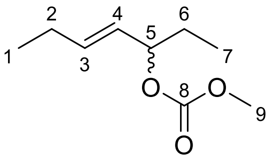

$C_9H_{16}O_3$

M: 172.22 g/mol.

^{1}H NMR (300 MHz, $CDCl_3$): δ [ppm] = 0.91 (t, ^3J = 7.5 Hz, 3H, H-7); 0.99 (t, ^3J = 7.5 Hz, 3H, H-1); 1.55 – 1.80 (m, 2H, H-6); 2.06 ($\Psi_{quint.}$, ^3J = 6.4 Hz, 2H, H-2); 3.76 (s, 3H, H-9); 4.95 (Ψ_q, ^3J = 6.9 Hz, 1H, H-5); 5.39 (dd, ^3J = 15.4 Hz, 3J = 7.7 Hz, 1H, H-4); 5.81 (dt, ^3J = 15.4 Hz, ^3J = 6.2 Hz, 1H, H-3).

^{13}C NMR (75 MHz, $CDCl_3$): δ [ppm] = 9.5 (C-7); 13.2 (C-1); 25.3 (C-2); 27.6 (C-6); 54.5 (C-9); 80.8 (C-5); 126.6 (C-4); 137.0 (C-3); 155.4 (C-8).

IR (ATR): ṽ [cm-1] = 2967 (w); 2938 (w); 2879 (w); 2852 (w); 1744 (s); 1672 (w); 1583 (w); 1456 (w); 1442 (m); 1382 (w); 1368 (w); 1348 (w); 1304 (w); 1253 (s); 1200 (w); 1169 (w); 1137 (w); 1095 (w); 1075 (w); 1056 (w); 1034 (w); 968 (m); 946 (m); 921 (m); 884 (w); 828 (w); 792 (m); 742 (w); 714 (w).

GC/MS (EI, 70 eV): m/z (%) = 172 ([M]$^+$, 1); 143 ([M]+-C_2H_5, 3); 96 (45); 81 (100); 67 (30); 55 ([C_4H_7]$^+$, 45).

General procedure 1: Asymmetric Pd-catalyzed *N*-allylation of amino acid esters

A solution of a chiral ligand **L*** (2.4 mol%) and $[PdCl(allyl)]_2$ (1 mol%) in anhydrous THF (0.4 ml per mmol of the carbonate) was cooled to -10°C before carbonate *rac*-**4a** (1.0 eq.) was added by means of a syringe. After 30 min the amino acid ester (2.0 eq.) was added and stirring was continued at -10°C until complete conversion was observed by means of GC-MS (22 h if not mentioned otherwise). The clear yellow solution was then filtered with MTBE through a small pad of Celite and the solvent was evaporated. The crude product was purified by flash column chromatography as specified. In the case of glycine and serine derivatives the enantiomeric excess was determined by means of GC (FID) using a chiral stationary phase. In the case of all other amino acid derivatives the diastereomeric excess was determined by means of GC (FID) using an achiral *Optima*-5 *Accent* column.

For the synthesis of racemic reference samples 6 mol% of dppe and 2.0 mol% of $[PdCl(allyl)]_2$ were used at room temperature.

(*S*,*E*)-Hept-4-en-3-yl-glycin *tert*-butyl ester ((*S*)-3a)

According to **general procedure 1**, 344 mg (2.00 mmol) of carbonate *rac*-**4a** were reacted with glycine *tert*-butyl ester (**5a**) using the chiral ligand **L6** to yield 376 mg (1.65 mmol, 83%, *er = 98/2*) of the allylic amine (*R*)-**3a** after purification by flash column chromatography (Silica, cHex/EtOAc = 4/1) as a yellow oil.

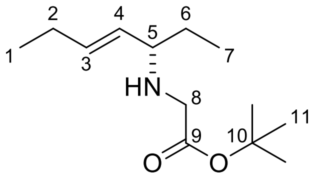

$C_{13}H_{25}NO_2$

M: 227.34 g/mol.

TLC: R_f = 0.18 (Silica, cHex/EtOAc = 4/1), $KMnO_4$-reagent.

^{1}H NMR (500 MHz, $CDCl_3$): δ [ppm] = 0.87 (t, $^3J = 7.5$ *Hz*, 3H, H-7); 0.99 (t, $^3J = 7.5$ *Hz*, 3H, H-1); 1.35 – 1.44 (m, 1H, H-6); 1.46 (s, 9H, H-11); 1.49 – 1.57 (m, 1H, H-6'); 1.85 (s, br, 1H, NH); 2.01 – 2.07 (m, 2H, H-2); 2.84 (td, $^3J = 8.3$ *Hz,* $^3J = 5.3$ *Hz,* 1H, H-5); 3.21 – 3.31 (m, 2H, H-8); 5.12 (ddt, $^3J = 15.3$ *Hz*, $^3J = 8.6$ *Hz*, $^4J = 1.5$ *Hz*, 1H, H-4); 5.70 (dt, $^3J = 15.3$ *Hz*, $^3J = 6.3$ *Hz*, 1H, H-3).

^{13}C NMR (125 MHz, $CDCl_3$): δ [ppm] = 10.6 (C-7); 13.9 (C-1); 25.5 (C-2); 28.3 (C-11/12/13); 29.0 (C-6); 49.3 (C-8); 62.5 (C-5); 81.1 (C-10); 131.1 (C-4); 135.2 (C-3); 172.3 (C-9).

IR (ATR): $\tilde{\nu}$ [cm-1] = 3450 (w); 3336 (w); 3002 (w); 2964 (m); 2933 (w); 2876 (w); 2857 (w); 2812 (w); 1733 (s); 1667 (w); 1458 (w); 1421 (w); 1393 (w); 1367 (m); 1350 (w); 1251 (w); 1227 (m); 1218 (m); 1151 (s); 1089 (w); 1071 (w); 1035 (w); 970 (m); 937 (w); 917 (w); 876 (w); 850 (m); 792 (w); 755 (w); 701 (w).

GC/MS (EI, 70 eV): m/z (%) = 227 ($[M]^+$, 2); 198 ($[M]^+$-C_2H_5, 24); 170 ($[M]^+$-C_4H_{10}, 10); 142 (100); 126 ($[M]^+$-CO_2*t*Bu, 25); 112 ($[C_7H_{14}N]^+$, 12); 97 ($[C_7H_{13}]^+$, 49); 83 (8); 69 (15); 55 (50).

HR/MS (ESI): calculated for $[M+H]^+$: 228.1958; found: 228.1955;
calculated for $[M+Na]^+$: 250.1778; found: 250.1778.

$[\boldsymbol{\alpha}]_{\lambda}^{\mathbf{20}}$(CHCl$_3$, c = 0.510 g/100 ml): $[\alpha]_{436}^{20}$= -45.9 $[\alpha]_{546}^{20}$ = -28.5°; $[\alpha]_{579}^{20}$= -25.6°; $[\alpha]_{589}^{20}$= -25.2°.

Determination of enantiomeric excess: *Agilent 6890N* GC system using a *CP-Chirasil-Dex CB* column by *Varian* (flow (H_2): 0.9 ml/min; 90 °C to 110 °C with 1 °C/min, inlet temp.: 170 °C).

(*R*,*E*)-Hept-4-en-3-ylglycin *tert*-butyl ester ((*R*)-3a)

According to **general procedure 1**, 517 mg (3.00 mmol) of carbonate *rac*-**4a** were reacted with glycine *tert*-butyl ester (**5a**) using the chiral ligand *ent*-**L6** to yield 570 mg (2.51 mmol, 84%, *er = 98/2*) of the allylic amine (*S*)-**3a** after purification by flash column chromatography (Silica, cHex/EtOAc = 4/1) as a yellow oil.

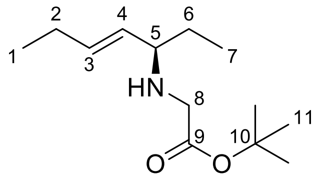

$C_{13}H_{25}NO_2$

M: 227.34 g/mol.

TLC, **NMR**, **IR** and **GC-MS** data identical with (*R*)-**3a**

TLC: R$_f$ = 0.18 (Silica, cHex/EtOAc = 4/1), $KMnO_4$-reagent.

$[\boldsymbol{\alpha}]_{\lambda}^{\mathbf{20}}$(CHCl$_3$, c = 0.515 g/100 ml): $[\alpha]_{436}^{20}$= +47.6 $[\alpha]_{546}^{20}$ = +30.9°; $[\alpha]_{579}^{20}$= +27.3°; $[\alpha]_{589}^{20}$= +25.8°.

Determination of enantiomeric excess: *Agilent 6890N* GC system using a *CP-Chirasil-Dex CB* column by *Varian* (flow (H_2): 0.9 ml/min; 90 °C to 110 °C with 1 °C/min, inlet temp.: 170 °C).

(*S*,*E*)-Hept-4-en-3-yl-glycin methyl ester ((*S*)-3b)

According to **general procedure 1**, 104 mg (0.604 mmol) of carbonate *rac*-**4a** were reacted with glycine methyl ester hydrochloride (**5b**) using the chiral ligand **L6** and 0.11 ml Et_3N to yield 99.6 mg (0.54 mmol, 89%, *er = 98/2*) of the allylic amine (*R*)-**3b** after purification by flash column chromatography (Silica, cHex/EtOAc = 2/1) as a yellow oil.

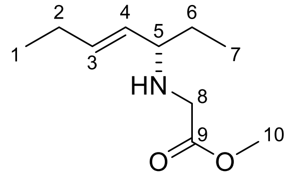

$C_{10}H_{19}NO_2$

M: 185.27 g/mol.

TLC: R$_f$ = 0.16 (Silica, cHex/EtOAc = 2/1), $KMnO_4$-reagent.

^{1}H NMR (500 MHz, CDCl$_3$): δ [ppm] = 5.57 (dt, ^3J = 15.3, 6.3 Hz, 1H, H-3), 5.13 (ddt, ^3J = 15.3, 8.6, ^4J = 1.6 Hz, 1H, H-4), 3.73 (s, 3H, H-10), 3.52 – 3.26 (m, 2H, H-8), 2.85 (td, ^3J = 8.3, 5.2 Hz, 1H, H-5), 2.06 (qdd, ^3J = 7.5, 6.2, ^4J = 1.6 Hz, 2H, H-2), 1.71 (s, 1H, NH), 1.65 – 1.36 (m, 2H, H-6), 1.00 (t, ^3J = 7.5 Hz, 3H, H-1), 0.89 (t, ^3J = 7.5 Hz, 3H, H-7).

^{13}C NMR (125 MHz, CDCl$_3$): δ [ppm] = 10.4 (C-7); 13.8 (C-1); 25.3 (C-2); 28.8 (C-6); 48.3 (C-8); 51.8 (C-10); 62.5 (C-5); 130.8 (C-4); 135.3 (C-3); 173.4 (C-9).

IR (ATR): $\tilde{\nu}$ [cm-1] = 3329 (w), 2962 (m), 2933 (w), 2878 (w), 1742 (s), 1459 (m), 1436 (m), 1350 (w), 1200 (s), 1153 (s), 970 (s), 877 (w), 744 (m), 686 (m), 571 (w).

GC/MS (EI, 70 eV): m/z (%) = 156 ($[M]^+$-C_4H_{10}, 100); 126 ($[M]^+$-CO_2*t*Bu, 6); 112 ($[C_7H_{14}N]^+$, 3); 96 ($[C_7H_{13}]^+$ 26, 81 (5), 55 (11), 41 (9).

$[\boldsymbol{\alpha}]_{\lambda}^{\mathbf{20}}$(CHCl3, c = 0.939 g/100 ml): $[\alpha]_{436}^{20}$= -52.4 $[\alpha]_{546}^{20}$ = -32.2°; $[\alpha]_{579}^{20}$= -28.6°; $[\alpha]_{589}^{20}$= -27.6°.

Determination of enantiomeric excess: *Agilent 6890N* GC system using a *Mega-Dex Det-Beta* column by *Varian* (flow (H_2): 3.8 ml/min; 50 °C hold for 5 min, 50 °C to 75 °C with 1 °C/min, 75 °C to 82 °C with 0.2 °C/min, inlet temp.: 170 °C).

(*R*,*E*)-Hept-4-en-3-ylglycin *tert*-butyl ester ((*R*)-3b)

According to **general procedure 1**, 104 mg (0.604 mmol) of carbonate *rac*-**4a** were reacted with glycine methyl ester hydrochloride (**5b**) using the chiral ligand **L6** and 0.11 ml Et_3N to yield 99.5 mg (0.54 mmol, 89%, *er = 98/3*) of the allylic amine (*R*)-**3b** after purification by flash column chromatography (Silica, cHex/EtOAc = 2/1) as a yellow oil

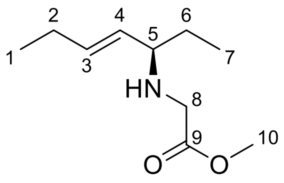

$C_{10}H_{19}NO_2$

M: 185.27 g/mol.

TLC, **NMR**, **IR** and **GC-MS** data identical with (*S*)-**3b**

TLC: R_f = 0.16 (Silica, cHex/EtOAc = 2/1), $KMnO_4$-reagent.

$[\boldsymbol{\alpha}]_{\lambda}^{\mathbf{20}}$(CHCl3, c = 0.809 g/100 ml): $[\alpha]_{436}^{20}$= +59.0 $[\alpha]_{546}^{20}$ = +37.4°; $[\alpha]_{579}^{20}$= +33.2°; $[\alpha]_{589}^{20}$= +31.75°.

Determination of enantiomeric excess: *Agilent 6890N* GC system using a *Mega-Dex Det-Beta* column by *Varian* (flow (H_2): 3.8 ml/min; 50 °C hold for 5 min, 50 °C to 75 °C with 1 °C/min, 75 °C to 82 °C with 0.2 °C/min, inlet temp.: 170 °C).

(*S*)-Methyl 2-((*S,E*)-hept-4-en-3-ylamino)propanoate ((*S,S*)-3c)

According to **general procedure 1**, 172 mg (1.00 mmol) of carbonate *rac*-**4a** were reacted with L-alanine methyl ester ((*S*)-**5c**) using the chiral ligand **L6** to yield 164 mg (0.824 mmol, 83%, *dr* > 2/98) of the allylic amine (*S,S*)-**3c** after purification by flash column chromatography (Silica, cHex/EtOAc = 4/1) as a yellow oil.

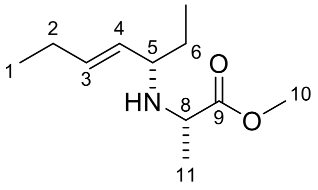

$C_{11}H_{21}NO_2$

M: 199.29 g/mol.

TLC: R_f = 0.25 (Silica, cHex/EtOAc = 3/1), $KMnO_4$-reagent.

^{1}H NMR (500 MHz, $CDCl_3$): δ [ppm] = 0.86 (t, ^3J = 7.5 Hz, 3H, H-7); 0.99 (t, ^3J = 7.5 Hz, 3H, H-1); 1.26 (d, ^3J = 7.1 Hz, 3H, H-11); 1.35 – 1.51 (m, 2H, H-6); 1.79 (s, br, 1H, NH); 2.01 – 2.07 (m, 2H, H-2); 2.76 (td, ^3J = 8.2 Hz, ^3J = 5.6 Hz, 1H, H-5); 3.43 (q, ^3J = 7.1 Hz, 1H, H-8); 3.72 (s, 3H, H-10); 5.10 (ddt, ^3J = 15.3 Hz, ^3J = 8.6 Hz, ^4J = 1.5 Hz, 1H, H-4); 5.50 (dt, ^3J = 15.3 Hz, ^3J = 6.3 Hz, 1H, H-3).

^{13}C NMR (125 MHz, $CDCl_3$): δ [ppm] = 10.6 (C-7); 13.9 (C-1); 19.9 (C-11); 25.5 (C-2); 29.3 (C-6); 51.8 (C-10); 53.8 (C-8); 61.6 (C-5); 131.0 (C-4); 135.3 (C-3); 177.1 (C-9).

IR (ATR): $\tilde{\nu}$ [cm-1] = 3465 (w); 3334 (w); 3030 (w); 2963 (m); 2934 (w); 2876 (w); 2857 (w); 2817 (w); 1737 (s); 1667 (w); 1455 (m); 1435 (m); 1373 (w); 1342 (w); 1330 (w); 1304 (w); 1247 (w); 1201 (m); 1169 (s); 1157 (s); 1076 (m); 1058 (m); 1038 (w); 1010 (w); 970 (s); 909 (w); 891 (w); 876 (w); 849 (w); 828 (w); 793 (w); 770 (w); 750 (m); 699 (w); 656 (w); 628 (w).

GC/MS (EI, 70 eV): m/z (%) = 198 (1); 170 ([M]+-C2H5, 100); 140 ([M]+-CO2Me, 20); 110 (30); 97 ([C7H13]+, 18); 82 (10); 67 (10); 55 (40).

HR/MS (ESI): calculated for $[M+H]^+$: 220.1645; found: 220.1644;
calculated for $[M+Na]^+$: 222.1465; found: 222.1466.

$[\alpha]_{\lambda}^{20}$(CHCl3, c = 0.530 g/100 ml): $[\alpha]_{436}^{20}$= -164.0 $[\alpha]_{546}^{20}$ = -97.9°; $[\alpha]_{579}^{20}$= -87.1°; $[\alpha]_{589}^{20}$= -84.0°.

(*S*)-Methyl 2-((*R,E*)-hept-4-en-3-ylamino)propanoate ((*R,S*)-3c)

According to **general procedure 1**, 431 mg (2.50 mmol) of carbonate *rac*-**4a** were reacted with L-alanine methyl ester ((*S*)-**3c**) using the chiral ligand *ent*-**L6** to yield 456 mg (2.29 mmol, 86%, *dr* > *98/2*) of the allylic amine (*R,S*)-**3c** after purification by flash column chromatography (Silica, cHex/EtOAc = 3/1) as a yellow oil.

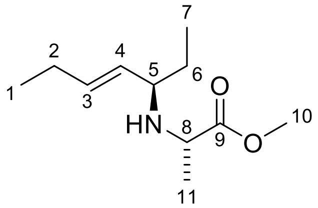

$C_{11}H_{21}NO_2$

M: 199.29 g/mol.

TLC: R_f = 0.25 (Silica, cHex/EtOAc = 3/1), $KMnO_4$-reagent.

^{1}H NMR (500 MHz, $CDCl_3$): δ [ppm] = 0.84 (t, ^3J = 7.5 Hz, 3H, H-7); 0.97 (t, ^3J = 7.5 Hz, 3H, H-1); 1.26 (d, ^3J = 6.9 Hz, 3H, H-11); 1.30 – 1.39 (m, 1H, H-6); 1.51 – 1.59 (m, 1H, H-6'); 1.61 (s, br, 1H, NH); 1.98 – 2.05 (m, 2H, H-2); 2.85 (td, ^3J = 8.6 Hz, ^3J = 4.8 Hz, 1H, H-5); 3.38 (q, ^3J = 6.9 Hz, 1H, H-8); 3.69 (s, 3H, H-10); 5.11 (ddt, ^3J = 15.3 Hz, ^3J = 8.7 Hz, 4J = 1.5 Hz, 1H,H-4); 5.54 (dt, ^3J = 15.3 Hz, ^3J = 6.3 Hz, 1H, H-3).

^{13}C NMR (125 MHz, $CDCl_3$): δ [ppm] 10.6 (C-7); 13.8 (C-1); 19.3 (C-11); 25.4 (C-2); 28.5 (C-6); 51.9 (C-10); 54.2 (C-8); 61.4 (C-5); 131.3 (C-4); 134.7 (C-3); 176.8 (C-9).

IR (ATR): $\tilde{\nu}$ [cm-1] = 3460 (w); 3325 (w); 3027 (w); 2964 (m); 2934 (w); 2875 (w); 2847 (w); 1736 (s); 1666 (w); 1453 (m); 1434 (m); 1372 (w); 1348 (w); 1332 (w); 1301 (w); 1249 (w); 1198 (m); 1164 (s); 1136 (m); 1095 (w);

1059 (m); 1035 (w); 969 (s); 920 (w); 909 (w); 894 (w); 849 (w); 831 (w); 790 (w); 749 (m); 730 (m); 653 (w).

GC/MS (EI, 70 eV): m/z (%) = 198 (1); 170 ([M]$^+$ -C_2H_5, 100); 140 ([M]$^+$ -CO_2Me, 20); 110 (50); 97 ([C_7H_{13}]$^+$, 20); 82 (17); 67 (20); 55 (75).

HR/MS (ESI): calculated for [M+H]$^+$: 220.1645; found: 220.1647;
calculated for [M+Na]$^+$: 222.1465; found: 222.1466.

$[\alpha]_\lambda^{20}$(CHCl$_3$, c = 0.530 g/100 ml): $[\alpha]_{436}^{20}$= -64.2 $[\alpha]_{546}^{20}$ = -36.0°; $[\alpha]_{579}^{20}$= -31.2°; $[\alpha]_{589}^{20}$= -30.0°.

(*S*)-Methyl 2-((*S,E*)-hept-4-en-3-ylamino)-3-methylbutanoate ((*S,S*)-3d)

According to **general procedure 1**, 172 mg (1.00 mmol) of carbonate *rac*-**4a** were reacted with L-alanine methyl ester ((*S*)-**5d**) using the chiral ligand **L6** to yield 190 mg (0.837 mmol, 84%, *dr* > *98/2*) of the allylic amine (*S,S*)-**3d** after purification by flash column chromatography (Silica, cHex/EtOAc = 20/1) as a yellow oil.

$C_{13}H_{25}NO_2$

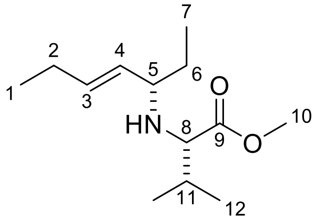

M: 227.34 g/mol.

TLC: R$_f$ = 0.25 (Silica, cHex/EtOAc = 20/1), $KMnO_4$-reagent.

^{1}H NMR (500 MHz, CDCl$_3$): δ [ppm] = 0.85 (t, ^3J = 7.5 Hz, 3H, H-7); 0.91 (Ψ dd, *J* = 6.8 Hz, *J* = 2.8 Hz, 6H, H-12); 0.98 (t, ^3J = 7.5 Hz, 3H, H-1); 1.35 – 1.49 (m, 2H, H-6); 1.62 (s, br, 1H, NH); 1.81 – 1.90 (m, 1H, H-11); 2.00 – 2.06 (m, 2H, H-2); 2.67 (td, ^3J = 8.0 Hz, ^3J = 5.7 Hz, 1H, H-5); 3.10 (d, ^3J = 5.8 Hz, 1H, H-8); 3.71 (s, 3H, H-10); 5.07 (ddt, ^3J = 15.3 Hz, ^3J = 8.5 Hz, ^4J = 1.5 Hz, 1H, H-4); 5.49 (dt, ^3J = 15.3 Hz, ^3J = 6.3 Hz, 1H, H-3).

^{13}C NMR (125 MHz, CDCl$_3$): δ [ppm] = 10.6 (C-7); 14.0 (C-1); 18.9 (C-12); 19.3 (C-12); 25.5 (C-2); 29.4 (C-6); 31.9 (C-11); 51.4 (C-10); 61.8 (C-5); 64.2 (C-8); 131.6 (C-4); 134.9 (C-3); 176.4 (C-9).

IR (ATR): $\tilde{\nu}$ [cm-1] = 3455 (w); 3336 (w); 3027 (w); 2962 (m); 2934 (w); 2876 (w); 2860 (w); 2809 (w); 1734 (s); 1697 (w); 1672 (w); 1463 (m); 1434 (w); 1385 (w); 1366 (w); 1350 (w); 1335 (w); 1308 (w); 1298 (w); 1268 (w); 1235 (w); 1198 (m); 1180 (m); 1159 (s); 1114 (m); 1080 (w); 1066 (w); 1021 (w); 997 (m); 970 (m); 927 (w); 903 (w); 886 (w); 846 (w); 831 (w); 795 (w); 765 (w); 738 (w); 695 (w); 676 (w); 651 (w); 620 (w).

GC/MS (EI, 70 eV): m/z (%) = 227 (5); 198 ([M]+-C2H5, 100); 168 ([M]+-CO2Me, 24); 138 (35); 112 ([$C_7H_{14}N$]$^+$, 12); 97 ([C7H13]+, 30); 72 (40); 55 (70).

HR/MS (ESI): calculated for [M+H]$^+$: 228.1958; found: 228.1957;
calculated for [M+Na]$^+$: 250.1778; found: 250.1778.

$[\alpha]_\lambda^{20}$(CHCl$_3$, c = 0.515 g/100 ml): $[\alpha]_{436}^{20}$= -114.8°; $[\alpha]_{546}^{20}$ = -70.6°; $[\alpha]_{579}^{20}$= -62.6°; $[\alpha]_{589}^{20}$= -61.0°.

(*S*)-Methyl 2-((*R,E*)-hept-4-en-3-ylamino)-3-methylbutanoate ((*R,S*)-3d)

According to **general procedure 1**, 172 mg (1.00 mmol) of carbonate *rac*-**4a** were reacted with L-alanine methyl ester ((*S*)-**5d**) using the chiral ligand *ent*-**L6** to yield 181 mg (0.796 mmol, 79%, *dr* > *98/2*) of the allylic amine (*R,S*)-**3d** after purification by flash column chromatography (Silica, cHex/EtOAc = 20/1) as a yellow oil.

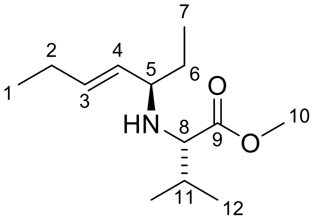

$C_{13}H_{25}NO_2$

M: 227.34 g/mol.

TLC: R_f = 0.25 (Silica, cHex/EtOAc = 20/1), $KMnO_4$-reagent.

^{1}H NMR (500 MHz, $CDCl_3$): δ [ppm] 0.85 (t, ^3J = 7.4 Hz, 3H, H-7); 0.92 (Ψ t, ^3J = 7.2 Hz, 6H, H-12); 0.96 (t, ^3J = 7.5 Hz, 3H, H-1); 1.29 – 1.37 (m, 1H, H-6); 1.48 – 1.56 (m, 2H, H-6', NH); 1.81 – 1.90 (m, 1H, H-11); 1.96 – 2.02 (m, 2H, H-2); 2.73 (td, ^3J = 8.3 Hz, ^3J = 5.2 Hz, 1H, H-5); 2.97 (d, ^3J = 5.8 Hz, 1H, H-8); 3.67 (s, 3H, H-10); 5.14 (ddt, ^3J = 15.3 Hz, ^3J = 8.7 Hz, ^4J = 1.5 Hz, 1H, H-4); 5.50 (dt, ^3J = 15.3 Hz, ^3J = 6.2 Hz, 1H, H-3).

^{13}C NMR (125 MHz, $CDCl_3$): δ [ppm] = 10.6 (C-7); 13.7 (C-1); 18.9 (C-12 o. C-13); 19.2 (C-12 o. C-13); 25.4 (C-2); 28.4 (C-6); 32.1 (C-11); 51.4 (C-10); 62.9 (C-5); 65.2 (C-8); 132.0 (C-4); 133.9 (C-3); 176.5 (C-9).

IR (ATR): $\tilde{\nu}$ [cm-1] = 3458 (w); 3330 (w); 3022 (w); 2962 (m); 2933 (m); 2875 (w); 1735 (s); 1697 (w); 1666 (w); 1463 (m); 1434 (m); 1384 (w); 1365 (w); 1332 (w); 1309 (w); 1269 (w); 1236 (w); 1196 (m); 1179 (m); 1156 (s); 1110 (m); 1082 (w); 1064 (w); 1022 (w); 998 (m); 968 (m); 899 (w); 883 (w); 844 (w); 834 (w); 790 (m); 768 (m); 730 (w); 680 (w); 663 (w); 623 (w).

GC/MS (EI, 70 eV): m/z (%) = 226 (1); 198 ($[M]^+$ -C_2H_5, 100); 168 ($[M]^+$ -CO_2Me, 24); 138 (35); 112 ($[C_7H_{14}N]^+$, 12); 97 ($[C_7H_{13}]^+$, 49); 72 (53); 55 (80).

HR/MS (ESI): calculated for $[M+H]^+$: 228.1958; found: 228.1954;
calculated for $[M+Na]^+$: 250.1778; found: 250.1777.

$[\alpha]_\lambda^{20}$($CHCl_3$, c = 0.505 g/100 ml): $[\alpha]_{436}^{20}$= -54.9°; $[\alpha]_{546}^{20}$ = -31.7°; $[\alpha]_{579}^{20}$= -27.9°; $[\alpha]_{589}^{20}$= -27.5°.

(*S*)-Methyl 2-((*S,E*)-hept-4-en-3-ylamino)-3-phenylpropanoate ((*S,S*)-3e)

According to **general procedure 1**, 172 mg (1.00 mmol) of carbonate *rac*-**4a** were reacted with L-phenylalanine methyl ester ((S)-**3e**) using the chiral ligand **L6** to yield 239 mg (0.798 mmol, 87%, *dr =97/3*) of the allylic amine (*S,S*)-**3e** after purification by flash column chromatography (Silica, cHex/EtOAc = 10/1) as a yellow oil.

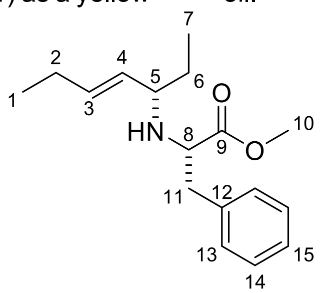

$C_{17}H_{25}NO_2$

M: 275.39 g/mol.

TLC: R_f = 0.26 (Silica, cHex/EtOAc = 10/1), $KMnO_4$-reagent.

^{1}H NMR (500 MHz, $CDCl_3$): δ [ppm] = 0.82 (t, ^3J = 7.5 Hz, 3H, H-7); 0.96 (t, ^3J = 7.5 Hz, 3H, H-1); 1.31 – 1.39 (m, 1H, H-6); 1.40 – 1.48 (m, 1H, H-6'); 1.64 (s, br, 1H, NH); 1.97 – 2.03 (m, 2H, H-2); 2.74 (td, ^3J = 8.1 Hz, ^3J = 5.5 Hz, 1H, H-5); 2.86 – 2.95 (m, 2H, H-11); 3.60 – 3.63 (m, 4H, H-8/10); 4.99 (ddt, ^3J = 15.3 Hz, ^3J = 8.5 Hz, ^4J = 1.4 Hz, 1H, H-4); 5.44 (dt, ^3J = 15.3 Hz, ^3J = 6.3 Hz, 1H, H-3); 7.15 – 7.16 (m, 2H, H-13); 7.19 – 7.22 (m, 1H, H-15); 7.25 – 7.28 (m, 2H, H-14).

^{13}C NMR (125 MHz, $CDCl_3$): δ [ppm] = 10.5 (C-7); 13.9 (C-1); 25.4 (C-2); 29.3 (C-6); 40.3 (C-11); 51.6 (C-10); 60.1 (C-8); 61.3 (C-5); 126.7 (C-15); 128.4 (C-14); 129.3 (C-13); 130.8 (C-4); 135.2 (C-3); 137.6 (C-12); 175.8 (C-9).

IR (ATR): $\tilde{\nu}$ [cm-1] = 3458 (w); 3334 (w); 3111 (w); 3088 (w); 3065 (w); 3029 (w); 2962 (m); 2933 (w); 2875 (w); 2855 (w); 2812 (w); 1735 (s); 1669 (w); 1604 (w); 1586 (w); 1496 (w); 1455 (m); 1434 (m); 1380 (w); 1363 (w); 1350 (w); 1272 (w); 1195 (m); 1170 (s); 1131 (m); 1078 (w); 1031 (w); 1021 (w); 971 (m); 932 (w); 909 (w); 889 (w); 881 (w); 846 (w); 831 (w); 793 (w); 741 (m); 699 (s); 663 (w).

GC/MS (EI, 70 eV): m/z (%) = 276 (5); 246 ($[M]^+$ -C_2H_5, 30); 216 ($[M]^+$ -CO_2Me, 13); 184 ($[M]^+$ -C_7H_7, 26); 120 (24); 97 ($[C_7H_{13}]^+$, 40); 91 ($[C_7H_7]^+$, 100); 88 (20); 77 (10); 65 (18); 55 (60).

HR/MS (ESI): calculated for $[M+H]^+$: 276.1958; found: 276.1959;
calculated for $[M+Na]^+$: 298.1778; found: 298.1779.

$[\alpha]_\lambda^{20}$($CHCl_3$, c = 0.535 g/100 ml): $[\alpha]_{365}^{20}$= +31.1°; $[\alpha]_{436}^{20}$= +6.0°; $[\alpha]_{546}^{20}$ = -2.6°; $[\alpha]_{579}^{20}$= -3.6°; $[\alpha]_{589}^{20}$= -3.8°.

(*S*)-Methyl 2-((*R,E*)-hept-4-en-3-ylamino)-3-phenylpropanoate ((*R,S*)-3e)

According to **general procedure 1**, 172 mg (1.00 mmol) of carbonate *rac*-**4a** were reacted with L-phenylalanine methyl ester ((*S*)-**5e**) using the chiral ligand *ent*-**L6** to yield 241 mg (0.875 mmol, 88%, *dr > 99/1*) of the allylic amine (*R,S*)-**3e** after purification by flash column chromatography (Silica, cHex/EtOAc = 18/1) as a yellow oil.

TLC: R_f = 0.22 (Silica, cHex/EtOAc = 10/1), $KMnO_4$-reagent.

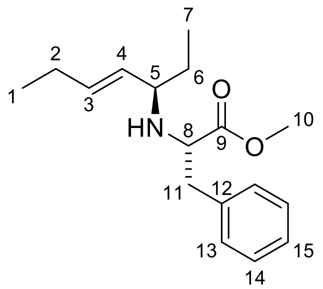

^{1}H NMR (500 MHz, $CDCl_3$): δ [ppm] 0.77 (t, ^3J = 7.4 Hz, 3H, H-7); 0.96 (t, ^3J = 7.5 Hz, 3H, H-1); 1.29 – 1.37 (m, 1H, H-6); 1.43 – 1.51 (m, 1H, H-6'); 1.62 (s, br, 1H, NH); 1.97 – 2.03 (m, 2H, H-2); 2.80 (td, ^3J = 8.3 Hz, ^3J = 5.4 Hz, 1H, H-5); 2.88 – 2.95 (m, 2H, H-11); 3.53 (t, ^3J = 6.9 Hz, 1H, H-8); 3.58 (s, 3H, H-10); 5.11 (ddt, ^3J = 15.3 Hz, ^3J = 8.7 Hz, ^4J = 1.5 Hz, 1H, H-4); 5.52 (dt, ^3J = 15.3 Hz, ^3J = 6.3 Hz, 1H, H-3); 7.16 – 7.18 (m, 2H, H-13); 7.20 – 7.23 (m, 1H, H-15); 7.26 – 7.29 (m, 2H, H-14).

^{13}C NMR (125 MHz, $CDCl_3$): δ [ppm] = 10.5 (C-7); 13.7 (C-1); 25.4 (C-2); 28.5 (C-6); 39.8 (C-11); 51.7 (C-10); 61.2 (C-8); 62.3 (C-5); 126.8 (C-15); 128.5 (C-14); 129.3 (C-13); 131.4 (C-4); 134.7 (C-3); 137.6 (C-12); 176.7 (C-9).

IR (ATR): $\tilde{\nu}$ [cm^{-1}] = 3455 (w); 3325 (w); 3113 (w); 3090 (w); 3066 (w); 3027 (w); 2962 (m); 2933 (w); 2875 (w); 2855 (w); 1735 (s); 1668 (w); 1604 (w); 1585 (w); 1496 (w); 1455 (m); 1434 (m); 1376 (w); 1350 (w); 1340 (w);

1268 (w); 1198 (m); 1166 (s); 1122 (m); 1077 (w); 1030 (w); 1022 (w); 970 (m); 936 (w); 907 (w); 889 (w); 881 (w); 844 (w); 831 (w); 790 (w); 743 (m); 699 (s); 667 (w); 620 (w); 602 (w).

GC/MS (EI, 70 eV): m/z (%) = 274 (1); 246 ([M]$^+$ -C_2H_5, 45); 216 ([M]$^+$ -CO_2Me, 13); 184 ([M]$^+$ -C_7H_7, 55); 120 (30); 97 ([C_7H_{13}]$^+$, 75); 91 ([C_7H_7]$^+$, 65); 88 (35); 81 (13); 77 (13); 67 (15); 65 (18); 55 (100).

HR/MS (ESI): calculated for [M+H]$^+$: 276.1958; found: 276.1957;
calculated for [M+na]$^+$: $^+$ 298.1778; found: 298.1779.

$[\alpha]_{\lambda}^{20}$(CHCl$_3$, c = 0.530 g/100 ml): $[\alpha]_{436}^{20}$= +88.7°, $[\alpha]_{546}^{20}$ = +48.6°; $[\alpha]_{579}^{20}$= +42.0°; $[\alpha]_{589}^{20}$= +39.4°.

(*R*)-Methyl 2-((*S,E*)-hept-4-en-3-ylamino)-3-phenylpropanoate ((S,*R*)-3e)

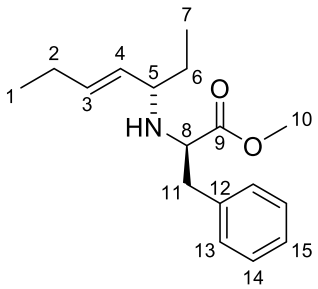

According to **general procedure 1**, 172 mg (1.00 mmol) of carbonate *rac*-**4a** were reacted with D-phenylalanine methyl ester ((*R*)-**5e**) using the chiral ligand **L6** to yield 213mg (0.773 mmol, 77%, *dr > 99/1*) of the allylic amine (*S,R*)-**3e** after purification by flash column chromatography (Silica, cHex/EtOAc = 18/1) as a yellow oil.

TLC, **NMR**, **IR** and **GC-MS** data identical with (*R,S*)-**3e**

$[\alpha]_{\lambda}^{20}$ (CHCl$_3$, c = 0.535 g/100 ml): $[\alpha]_{436}^{20}$= -90.2°; $[\alpha]_{546}^{20}$ = -48.1°; $[\alpha]_{579}^{20}$= +41.7°; $[\alpha]_{589}^{20}$= +40.3°.

(*R*)-Methyl 2-((*R,E*)-hept-4-en-3-ylamino)-3-phenylpropanoate ((R,R)-3e)

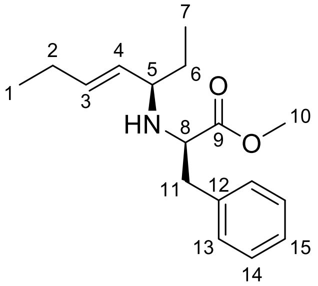

According to **general procedure 1**, 172 mg (1.00 mmol) of carbonate *rac*-**4a** were reacted with D-phenylalanine methyl ester ((*R*)-**5e**) using the chiral ligand *ent*-**L6** to yield 222 mg (0.773 mmol, 90%, *dr > 97/3*) of the allylic amine (*R,R*)-**3e** after purification by flash column chromatography (Silica, cHex/EtOAc = 18/1) as a yellow oil.

TLC, **NMR**, **IR** and **GC-MS** data identical with (*S,S*)-**3e**

$[\alpha]_{\lambda}^{20}$ (CHCl$_3$, c = 1.010 g/100 ml): $[\alpha]_{365}^{20}$= -31.4°; $[\alpha]_{436}^{20}$= -6.7 $[\alpha]_{546}^{20}$ = +2.5°; $[\alpha]_{579}^{20}$= +3.5°; $[\alpha]_{589}^{20}$= +4.0°.

(*S*)-Methyl 1-((*S,E*)-hept-4-en-3-ylamino)-3-hydroxypropanoate ((*S,S*)-3f)

According to **general procedure 1**, 172 mg (1.00 mmol) of carbonate *rac*-**4a** were reacted with L-serine methyl ester ((*S*)-**5k**) using the chiral ligand **L6** to yield 168 mg (0.780 mmol, 78%, *dr =97/3*) of the allylic amine (*S,S*)-**3k** after purification by flash column chromatography (Silica, CH_2Cl_2/EtOAc = 1/1) as a yellow oil.

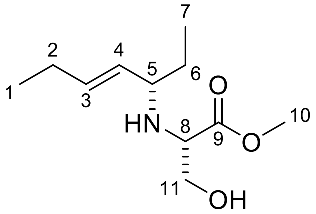

$C_{11}H_{21}NO_2$

M: 215.29 g/mol.

TLC: R_f = 0.13 (Silica, CH_2Cl_2/EtOAc = 1/1), $KMnO_4$-reagence.

^{1}H NMR (500 MHz, $CDCl_3$): δ [ppm] = 0.88 (t, ^3J = 7.5 Hz, 3H, H-7); 0.98 (t, ^3J = 7.5 Hz, 3H, H-1); 1.39 – 1.55 (m, 2H, H-6); 2.01 – 2.07 (m, 2H, H-2); 2.54 (s, br, 2H, NH, OH); 2.89 (td, ^3J = 8.2 Hz, ^3J = 5.7 Hz, 1H, H-5); 3.47 – 3.56 (m, 2H, H-8/11); 3.37 – 3.76 (m, 4H, H-11'/10); 5.08 (ddt, ^3J = 15.3 Hz, ^3J = 8.6 Hz, ^4J = 1.5 Hz, 1H, H-4); 5.53 (dt, ^3J = 15.3 Hz, ^3J = 6.3 Hz, 1H, H-3).

^{13}C NMR (125 MHz, $CDCl_3$): δ [ppm] = 10.8 (C-7); 13.9 (C-1); 25.5 (C-2); 29.3 (C-6); 52.2 (C-10); 59.9 (C-8); 61.8 (C-5); 63.3 (C-11); 130.9 (C-4); 135.8 (C-3); 174.3 (C-9).

IR (ATR): $\tilde{\nu}$ [cm^{-1}] = 3438 (w, br); 3309 (w); 3032 (w); 2962 (m); 2934 (m); 2876 (w); 2857 (w); 1737 (s); 1669 (w); 1459 (m); 1435 (m); 1403 (w); 1378 (w); 1363 (w); 1347 (w); 1334 (w); 1269 (m); 1199 (s); 1175 (s); 1155 (s); 1134 (m); 1093 (m); 1063 (s); 1043 (m); 971 (s); 909 (w); 891 (w); 865 (w); 851 (m); 825 (m); 793 (m); 768 (m); 734 (m); 661 (w); 647 (m).

GC/MS (EI, 70 eV): m/z (%) = 214 (1); 186 ($[M]^+$ $-C_2H_5$, 100); 156 ($[M]^+$ $-CO_2Me$, 12); 126 (20); 112 ($[C_7H_{14}N]^+$, 8); 97 ($[C_7H_{13}]^+$, 30); 88 (20); 81 (15); 69 (14); 60 (20); 55 (60).

HR/MS (ESI): calculated for $[M+H]^+$: 216.1594; found: 226.1591;
calculated for $[M+Na]^+$: 238.1414; found: 238.1415.

$[\alpha]_\lambda^{20}$($CHCl_3$, c = 0.510 g/100 ml): $[\alpha]_{365}^{20}$= -243.7°; $[\alpha]_{436}^{20}$= -160.0°; $[\alpha]_{546}^{20}$ = -96.0°; $[\alpha]_{579}^{20}$= -84.8°; $[\alpha]_{589}^{20}$= -81.8°.

Determination of diastereomeric excess: *Hewlett Packard 6890* GC-System using a *HP-5* column by *Agilent* (flow (H_2): 1.5 ml/min; 50 °C to 130 °C with 1 °C/min; inlet temp.: 275 °C.

(*S*)-Methyl 1-((*S*,*E*)-hept-4-en-3-ylamino)-3-hydroxypropanoate ((*R,S*)-3f)

According to **general procedure 1**, 172 mg (1.00 mmol) of carbonate *rac*-**4a** were reacted with L-serine methyl ester ((*S*)-**5k**) using the chiral ligand *ent*-**L6** to yield 166 mg (0.771 mmol, 77%, *dr = 97/3*) of the allylic amine (*R,S*)-**3f** after purification by flash column chromatography (Silica, CH_2Cl_2/EtOAc = 1/1) as a yellow oil.

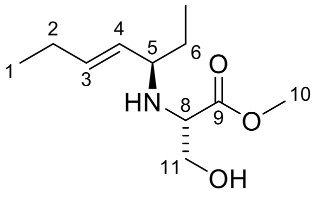

$C_{11}H_{21}NO_2$

TLC: R_f = 0.18 (Silica, CH_2Cl_2/EtOAc = 1/1), $KMnO_4$-reagent.

^{1}H NMR (500 MHz, $CDCl_3$): δ [ppm] = 0.88 (t, ^3J = 7.4 Hz, 3H, H-7); 0.97 (t, ^3J = 7.5 Hz, 3H, H-1); 1.34 – 1.43 (m, 1H, H-6); 1.53 – 1.62 (m, 1H, H-6'); 1.98 – 2.04 (m, 2H, H-2); 2.44 (s, br, 2H, NH, OH); 2.81 (td, ^3J = 8.3 Hz, ^3J = 5.7 Hz, 1H, H-5); 3.41 (dd, ^3J = 5.9 Hz, ^3J = 4.5 Hz, 1H, H-8); 3.56 (dd, ^2J = 10.6 Hz, ^3J = 5.9 Hz, 1H, H-11); 3.70

(dd, ^2J = 10.6 Hz, ^3J = 4.5 Hz, 1H, H-11'); 3.72 (s, 3H, H-10); 5.10 (ddt, ^3J = 15.3 Hz, ^3J = 8.8 Hz, ^4J = 1.5 Hz, 1H, H-4); 5.52 (dt, ^3J = 15.3 Hz, ^3J = 6.3 Hz, 1H, H-3).

^{13}C NMR (125 MHz, $CDCl_3$): δ [ppm] = 10.8 (C-7); 13.8 (C-1); 25.4 (C-2); 28.8 (C-6); 52.3 (C-10); 60.0 (C-8); 61.8 (C-5); 62.1 (C-11); 131.1 (C-4); 135.0 (C-3); 174.3 (C-9).

IR (ATR): $\tilde{\nu}$ [cm^{-1}] = 3434 (w, br); 3321 (w); 3026 (w); 2962 (m); 2934 (m); 2876 (m); 2853 (w); 1737 (s); 1668 (w); 1459 (m); 1435 (m); 1402 (w); 1376 (w); 1335 (m); 1265 (m); 1252 (m); 1198 (s); 1174 (s); 1123 (m); 1062 (s); 1031 (m); 971 (s); 912 (w); 892 (w); 854 (w); 790 (m); 765 (m); 733 (m); 696 (m); 661 (m); 646 (m).

GC/MS (EI, 70 eV): m/z (%) = 186 ([M]$^+$ -C_2H_5, 100); 156 ([M]$^+$ -CO_2Me, 12); 126 (25); 112 ([$C_7H_{14}N$]$^+$, 10); 97 ([C_7H_{13}]$^+$, 50); 88 (20); 81 (12); 67 (17); 60 (27); 55 (80).

HR/MS (ESI): calculated for [M+H]$^+$: 216.1594; found: 226.1592;
calculated for [M+Na]$^+$: 238.1414; found: 238.1415.

$[\boldsymbol{\alpha}]_{\boldsymbol{\lambda}}^{\mathbf{20}}$(CHCl$_3$, c = 0.510 g/100 ml): $[\alpha]_{365}^{20}$= -163.4°; $[\alpha]_{436}^{20}$= -100.6°; $[\alpha]_{546}^{20}$ = -57.7°; $[\alpha]_{579}^{20}$= -50.5°; $[\alpha]_{589}^{20}$= -48.6°.

Determination of diastereomeric excess: *Hewlett Packard 6890* GC-System using a *HP-5* column by *Agilent* (flow (H_2): 1.5 ml/min; 50 °C to 130 °C with 1 °C/min; inlet temp.: 275 °C.

(*S*)-*tert*-Butyl 2-((*S,E*)-hept-4-en-3-ylamino)-4-methylpentanoate ((*S,S*)-3g)

According to **general procedure 1**, 172 mg (1.00 mmol) of carbonate *rac*-**4a** were reacted with L-leucine *tert* butyl ester ((*S,S*)-**5g**) using the chiral ligand **L6** to yield 225 mg (0.798 mmol, 80%, 92% *dr >97/3*) of the allylic amine (*S,S*)-**3g** after purification by flash column chromatography (Silica, cHex/EtOAc = 18/1) as a yellow oil.

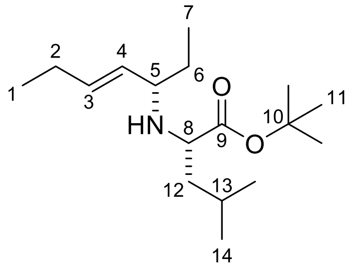

$C_{17}H_{33}NO_2$

M: 283.45 g/mol.

TLC: R_f = 0.21 (Silica, cHex/EtOAc = 18/1), $KMnO_4$-reagent.

^{1}H NMR (500 MHz, $CDCl_3$): δ [ppm] = 0.84 – 0.88 (m, 6H, H-7/H-11); 0.91 (d, ^3J = 6.6 Hz, 3H, H-11); 1.00 (t, ^3J = 7.5 Hz, 3H, H-1); 1.32 – 1.45 (m, 4H, H-6/12); 1.47 (s, 9H, H-11); 1.60 (s, br, 1H, NH); 1.68 – 1.76 (m, 1H, H-13); 2.02 – 2.08 (m, 2H, H-2); 2.73 – 2.77 (m, 1H, H-5); 3.22 (t, ^3J = 7.4 Hz, 1H, H-8); 5.09 (ddt, ^3J = 15.3 Hz, ^3J = 8.6 Hz, ^4J = 1.4 Hz, 1H, H-4); 5.52 (dt, ^3J = 15.3 Hz, ^3J = 6.4 Hz, 1H, H-3).

^{13}C NMR (125 MHz, $CDCl_3$): δ [ppm] = 10.7 (C-7); 14.1 (C-1); 22.6 (C-14); 22.7 (C-14); 25.1 (C-15); 25.5 (C-2); 28.3 (C-11); 29.5 (C-6); 43.4 (C-12); 57.7 (C-8); 61.4 (C-5); 80.6 (C-10); 131.4 (C-4); 135.0 (C-3); 176.1 (C-9).

IR (ATR): $\tilde{\nu}$ [cm-1] = 3445 (w); 3326 (w); 3007 (w); 2961 (m); 2933 (w); 2873 (w); 2852 (w); 2819 (w); 1728 (s); 1672 (w); 1459 (w); 1436 (w); 1392 (w); 1382 (w); 1367 (m); 1337 (w); 1309 (w); 1271 (w); 1256 (w); 1242 (w);

1210 (w); 1149 (s); 1086 (w); 1066 (w); 1037 (w); 1012 (w); 969 (m); 941 (w); 921 (w); 890 (w); 850 (w); 836 (w); 800 (w); 775 (w); 756 (w); 731 (w); 701 (w); 686 (w); 646 (w).

GC/MS (EI, 70 eV): m/z (%) = 254 ($[M]^+$ -C_2H_5, 5); 198 (35); 182 ($[M]^+$ -CO_2tBu, 100); 152 (10); 112 ($[C_7H_{14}N]^+$, 10); 110 (10); 97 ($[C_7H_{13}]^+$, 26); 86 (75); 81 (11); 67 (11); 55 (48).

HR/MS (ESI): calculated for $[M+H]^+$: 284.2584; found: 242.2583;
calculated for $[M+Na]^+$: 306.2404; found: 306.2405.

$[\alpha]_\lambda^{20}$(CHCl3, c = 0.515 g/100 ml): $[\alpha]_{365}^{20}$= -129.8°; $[\alpha]_{436}^{20}$= -88.1°; $[\alpha]_{546}^{20}$ = -53.5°; $[\alpha]_{579}^{20}$= -47.4°; $[\alpha]_{589}^{20}$= -45.8°.

(*S*)-*tert*-Butyl 2-((*R,E*)-hept-4-en-3-ylamino)-4-methylpentanoate ((*R,S*)-4g)

According to **general procedure 1**, 172 mg (1.00 mmol) of carbonate *rac*-**4a** were reacted with L-leucine *tert* butyl ester ((*S*)-**4g**) using the chiral ligand *ent*-**L6** to yield 226 mg (0.798 mmol, 80%, *dr* > *99/1*) of the allylic amine (*R,S*)-**4g** after purification by flash column chromatography (Silica, cHex/EtOAc = 18/1) as a yellow oil.

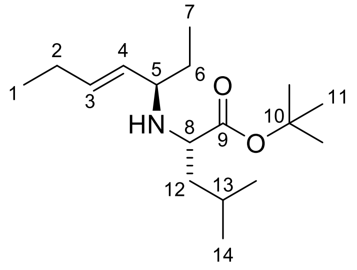

$C_{17}H_{33}NO_2$

M: 283.45 g/mol.

TLC: R_f = 0.21 (Silica, cHex/EtOAc = 18/1), $KMnO_4$-reagent.

^{1}H NMR (500 MHz, $CDCl_3$): δ [ppm] = 0.83 (t, ^3J = 7.4 Hz, 3H, H-7); 0.90 (d, ^3J = 6.6 Hz, 3H, H-14); 0.92 (d, ^3J = 6.6 Hz, 3H, H-14); 0.98 (t, ^3J = 7.5 Hz, 3H, H-1); 1.26 – 1.35 (m, 1H, H-6); 1.38 – 1.41 (m, 2H, H-12); 1.45 (s, 9H, H-11); 1.53 – 1.61 (m, 1H, H-6'); 1.64 (s, br, 1H, NH); 1.71 – 1.79 (m, 1H, H-13); 1.98 – 2.04 (m, 2H, H-2); 2.82 (td, ^3J = 8.4 Hz, ^3J = 4.5 Hz, 1H, H-5); 3.16 (t, ^3J = 7.2 Hz, 1H, H-8); 5.20 (ddt, ^3J = 15.3 Hz, ^3J = 8.4 Hz, ^4J = 1.4 Hz, 1H, H-4); 5.53 (dt, ^3J = 15.3 Hz, ^3J = 6.3 Hz, 1H, H-3).

^{13}C NMR (125 MHz, $CDCl_3$): δ [ppm] = 10.5 (C-7); 13.9 (C-1); 22.8 (C-14); 22.9 (C-14); 25.1 (C-13); 25.6 (C-2); 27.8 (C-6); 28.3 (C-11); 43.5 (C-12); 58.1 (C-8); 61.6 (C-5); 80.6 (C-10); 132.0 (C-4); 134.1 (C-3); 175.9 (C-9).

IR (ATR): $\tilde{\nu}$ [cm-1] = 3444 (w); 3318 (w); 3005 (w); 2960 (m); 2933 (m); 2873 (w); 2846 (w); 2820 (w); 1728 (s); 1669 (w); 1462 (w); 1435 (w); 1382 (w); 1367 (m); 1336 (w); 1308 (w); 1269 (w); 1257 (w); 1206 (w); 1149 (s); 1080 (w); 1067 (w); 1033 (w); 968 (m); 944 (w); 921 (w); 910 (w); 892 (w); 850 (w); 794 (w); 775 (w); 755 (w); 729 (w); 685 (w).

GC/MS (EI, 70 eV): m/z (%) = 254 ($[M]^+$ -C_2H_5, 5); 198 (40); 182 ($[M]^+$ -CO_2tBu, 100); 152 (12); 110 (10); 97 ($[C_7H_{13}]^+$, 35); 86 (90); 81 (12); 67 (11); 55 (50).

HR/MS (ESI): calculated for $[M+H]^+$: 284.2584; found: 242.2586;
calculated for $[M+Na]^+$ 306.2404; found: 306.2406.

$[\alpha]_{\lambda}^{20}$(CHCl3, c = 0.520 g/100 ml): $[\alpha]_{365}^{20}$= -27.9°; $[\alpha]_{436}^{20}$= -19.2°; $[\alpha]_{546}^{20}$ = -11.5°; $[\alpha]_{579}^{20}$= -10.3°; $[\alpha]_{589}^{20}$= -10.2°.

(*S*)-Methyl 2-((*S,E*)-hept-4-en-3-ylamino)-4-methylpentanoate ((*S,S*)-3h)

According to **general procedure 1**, 172 mg (1.00 mmol) of carbonate *rac*-**4a** were reacted with L-leucine methyl ester ((*S*)-**5h**) using the chiral ligand **L6** to yield 204 mg (0.845 mmol, 84%, *dr* > *98/2*) of the allylic amine (*S,S*)-**3h** after purification by flash column chromatography (Silica, cHex/EtOAc = 15/1) as a yellow oil.

$C_{14}H_{27}NO_2$

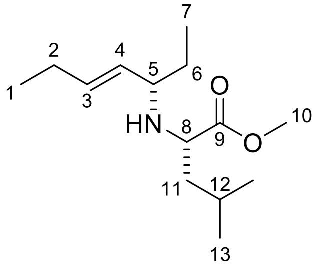

M: 241.37 g/mol.

TLC: R_f = 0.25 (Silica, cHex/EtOAc = 15/1), $KMnO_4$-reagent.

^{1}H NMR (500 MHz, $CDCl_3$): δ [ppm] = 0.84 – 0.87 (m, 6H, H-7/H-13 o. H14); 0.90 (d, ^3J = 6.7 Hz, 3H, H-13 o. H-14); 0.99 (d, ^3J = 7.5 Hz, 3H, H-1); 1.34 – 1.48 (m, 4H, H-6/11); 1.60 (s, br, 1H, NH); 1.69 – 1.77 (m, 1H, H-12); 2.01 – 2.07 (m, 2H, H-2); 2.70 (td, ^3J = 8.1 Hz, ^3J = 5.7 Hz, 1H, H-5); 3.37 (t, ^3J = 7.3 Hz, 1H, H-8); 3.71 (s, 3H, H-10); 5.08 (ddt, ^3J = 15.3 Hz, ^3J = 8.6 Hz, ^4J = 1.5 Hz, 1H, H-4); 5.51 (dt, ^3J = 15.3 Hz, ^3J = 6.3 Hz, 1H, H-3).

^{13}C NMR (125 MHz, $CDCl_3$): δ [ppm] = 10.6 (C-7); 14.0 (C-1); 22.2 (C-13); 22.9 (C-13); 25.0 (C-12); 25.5 (C-2); 29.4 (C-6); 43.3 (C-11); 51.6 (C-10); 57.0 (C-8); 61.6 (C-5); 131.3 (C-4); 135.1 (C-3); 177.3 (C-9).

IR (ATR): $\tilde{\nu}$ [cm-1] = 3457 (w); 3332 (w); 3020 (w); 2960 (m); 2934 (w); 2873 (w); 2845 (w); 2814 (w); 1737 (s); 1700 (w); 1669 (w); 1462 (m); 1434 (w); 1384 (w); 1368 (w); 1345 (w); 1330 (w); 1309 (w); 1270 (w); 1233 (w); 1196 (m); 1166 (s); 1145 (m); 1120 (m); 1088 (w); 1066 (w); 1042 (w); 1015 (w); 988 (w); 970 (m); 912 (w); 890 (w); 879 (w); 858 (w); 826 (w); 793 (w); 757 (w); 739 (w); 685 (w); 668 (w); 628 (w).

GC/MS (EI, 70 eV): m/z (%) = 242 (5); 212 ($[M]^+$ -C_2H_5, 100); 182 ($[M]^+$ -CO_2Me, 24); 152 (27); 110 (12); 97 ($[C_7H_{13}]^+$, 22); 86 (45); 84 (17); 67 (18); 55 (74).

HR/MS (ESI): calculated for $[M+H]^+$: 242.2115; found: 242.2112;
calculated for $[M+Na]^+$ 264.1934; found: 264.1934.

$[\alpha]_{\lambda}^{20}$(CHCl3, c = 0.515 g/100 ml): $[\alpha]_{365}^{20}$= -154.8°; $[\alpha]_{436}^{20}$= -104.3°; $[\alpha]_{546}^{20}$ = -63.9°; $[\alpha]_{579}^{20}$= -56.4°; $[\alpha]_{589}^{20}$= -54.5°.

(*S*)-Methyl 2-((*R,E*)-hept-4-en-3-ylamino)-4-methylpentanoate ((*R,S*)-3h)

According to **general procedure 1**, 172 mg (1.00 mmol) of carbonate *rac*-**4a** were reacted with L-leucine methyl ester ((*S*)-**5h**) using the chiral ligand *ent*-**L6** to yield 206 mg (0.863 mmol, 85%, *dr* > *98/2*) of the allylic amine (*R,S*)-**3h** after purification by flash column chromatography (Silica, cHex/EtOAc = 15/1) as a yellow oil.

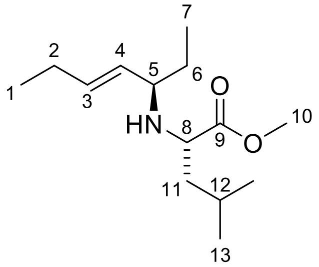

$C_{14}H_{27}NO_2$

M: 241.37 g/mol.

TLC: R_f = 0.21 (Silica, cHex/EtOAc = 15/1), $KMnO_4$-reagent.

^{1}H NMR (500 MHz, $CDCl_3$): δ [ppm] 0.84 (t, ^3J = 7.4 Hz, 3H, H-7); 0.89 (d, ^3J = 6.6 Hz, 3H, H-13); 0.91 (d, ^3J = 6.6 Hz, 3H, H-13); 0.97 (t, ^3J = 7.5 Hz, 3H, H-1); 1.29 – 1.39 (m, 1H, H-6); 1.40 – 1.46 (m, 2H, H-11); 1.47 – 1.57 (m, 2H, H-6', NH); 1.66 – 1.75 (m, 1H, H-12); 1.97 – 2.03 (m, 2H, H-2); 2.80 (td, ^3J = 8.4 Hz, ^3J = 4.9 Hz, 1H, H-5); 3.27 (t, ^3J = 7.2 Hz, 1H, H-8); 3.67 (s, 3H, H-10); 5.14 (ddt, ^3J = 15.3 Hz, ^3J = 8.6 Hz, ^4J = 1.5 Hz, 1H, H-4); 5.53 (dt, ^3J = 15.3 Hz, ^3J = 6.3 Hz, 1H, H-3).

^{13}C NMR (125 MHz, $CDCl_3$): δ [ppm] = 10.5 (C-7); 13.7 (C-1); 22.6 (C-13 o. C-14); 22.8 (C-13 o. C-14); 25.0 (C-12); 25.4 (C-2); 28.3 (C-6); 43.3 (C-11); 51.6 (C-10); 57.9 (C-8); 62.9 (C-5); 131.8 (C-4); 134.3 (C-3); 177.3 (C-9).

IR (ATR): $\tilde{\nu}$ [cm-1] = 3463 (w); 3325 (w); 3025 (w); 2959 (m); 2933 (m); 2873 (w); 2847 (w); 1738 (s); 1700 (w); 1666 (w); 1463 (m); 1434 (m); 1382 (w); 1368 (w); 1332 (w); 1309 (w); 1267 (m); 1230 (w); 1194 (m); 1162 (s); 1139 (m); 1118 (m); 1079 (w); 1066 (w); 1040 (w); 1022 (w); 969 (m); 915 (w); 889 (w); 876 (w); 860 (w); 842 (w); 827 (w); 790 (w); 757 (w); 732 (w); 675 (w).

GC/MS (EI, 70 eV): m/z (%) = 240 (1); 212 ($[M]^+$ -C_2H_5, 100); 182 ($[M]^+$ -CO_2Me, 24); 152 (22); 112 ($[C_7H_{14}N]^+$, 10); 97 ($[C_7H_{13}]^+$, 49); 86 (43); 81 (10); 69 (10); 55 (45).

HR/MS (ESI): calculated for $[M+H]^+$: 242.2115; found: 242.2113;
calculated for $[M+Na]^+$ 264.1934; found: 264.193.

$[\alpha]_{\lambda}^{20}$(CHCl3, c = 0.520 g/100 ml): $[\alpha]_{365}^{20}$= -43.2°; $[\alpha]_{436}^{20}$= -27.0°; $[\alpha]_{546}^{20}$ = -15.3°; $[\alpha]_{579}^{20}$= -13.5°; $[\alpha]_{589}^{20}$= -13.0°.

(*S*)-Di-*tert*-butyl 2-((*S,E*)-hept-4-en-3-ylamino)glutarate ((*S,S*)-3i)

According to **general procedure 1**, 172 mg (1.00 mmol) of carbonate *rac*-**4a** were reacted with L-glutamic acid di-*tert*-butyl ester ((*S*)-**5i**) using the chiral ligand **L6** to yield 235 mg (0.83 mmol, 84%, *dr* = *98/2*) of the allylic amine (*S,S*)-**3i** after purification by flash column chromatography (Silica, cHex/EtOAc = 10/1) as a yellow oil.

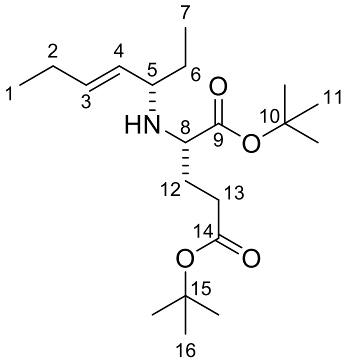

$C_{20}H_{37}NO_4$

M: 355.51 g/mol.

TLC: R_f = 0.14 (Silica, cHex/EtOAc = 10/1), $KMnO_4$-reagent.

^{1}H NMR (500 MHz, $CDCl_3$): δ [ppm] = 0.85 (t, ^3J = 7.4 Hz, 3H, H-7); 0.99 (t, ^3J = 7.5 Hz, 3H, H-1); 1.33 – 1.44 (m, 2H, H-6); 1.44 (s, 9H, H-11 o. H-16); 1.47 (s, 9H, H-11 o. H-16); 1.63 (s, br, 1H, NH); 1.70 – 1.78 (m, 1H, H-12); 1.81 – 1.88 (m, 1H, H-12'); 2.00 – 2.06 (m, 2H, H-2); 2.24 – 2.39 (m, 2H, H-13); 2.74 – 2.79 (m, 1H, H-5); 3.16 (dd, ^3J = 8.3 Hz, ^3J = 5.8 Hz, 1H, H-8); 5.07 (ddt, ^3J = 15.3 Hz, ^3J = 8.6 Hz, ^4J = 1.4 Hz, 1H, H-4); 5.50 (dt, ^3J = 15.3 Hz, ^3J = 6.4 Hz, 1H, H-3).

^{13}C NMR (125 MHz, $CDCl_3$): δ [ppm] = 10.6 (C-7); 14.0 (C-1); 25.5 (C-2); 28.2 (C-11/16); 29.2 (C-14); 29.5 (C-6); 32.5 (C-15); 58.6 (C-8); 61.5 (C-5); 80.2 (C-10 o. C-15); 81.0 (C-10 o. C-15); 131.4 (C-4); 134.9 (C-3); 172.7 (C-16); 175.2 (C-9).

IR (ATR): $\tilde{\nu}$ [cm^{-1}] = 3445 (w); 3327 (w); 3006 (w); 2966 (w); 2933 (w); 2876 (w); 2858 (w); 2821 (w); 1726 (s); 1671 (w); 1481 (w); 1457 (w); 1421 (w); 1392 (w); 1367 (m); 1324 (w); 1296 (w); 1255 (m); 1220 (w); 1148 (s); 1066 (w); 1037 (w); 1022 (w); 969 (m); 930 (w); 912 (w); 889 (w); 848 (w); 809 (w); 754 (w); 737 (w); 683 (w).

GC/MS (EI, 70 eV): m/z (%) = 355 ([M]+, 1); 326 ($[M]^+$ $-C_2H_5$, 7); 298 ($[M]^+$ $-C_4H_9$, 2); 270 (7); 254 ($[M]^+$ $-CO_2$tBu, 50); 214 (52); 198 (90); 186 (10); 168 (5); 146 (12); 130 (24); 124 (10); 112 (10); 102 (100); 97 ($[C_7H_{13}]^+$, 50); 84 (25); 81 (15); 67 (10); 57 (35); 56 (50); 55 (80).

HR/MS (ESI): calculated for $[M+H]^+$: 356.2795; found: 356.2795;
calculated for $[M+Na]^+$ 378.2615; found: 378.2617.

$[\alpha]_\lambda^{20}$ ($CHCl_3$, c = 0.515 g/100 ml): $[\alpha]_{365}^{20}$= -78.3°; $[\alpha]_{436}^{20}$= -53.3°; $[\alpha]_{546}^{20}$ = -32.6°; $[\alpha]_{579}^{20}$= -28.9°; $[\alpha]_{589}^{20}$= -28.4°.

(*S*)-Di-*tert*-butyl 2-((*R,E*)-hept-4-en-3-ylamino)glutarate ((*R,S*)-3i)

According to **general procedure 1**, 172 mg (1.00 mmol) of carbonate *rac*-**4a** were reacted with L-glutamic acid di-*tert*-butyl ester ((*S*)-**5i**) using the chiral ligand *ent*-**L6** to yield 296 mg (0.833 mmol, 83%, *dr* > *99/1*) of the allylic amine (*R,S*)-**3i** after purification by flash column chromatography (Silica, cHex/EtOAc = 10/1) as a yellow oil.

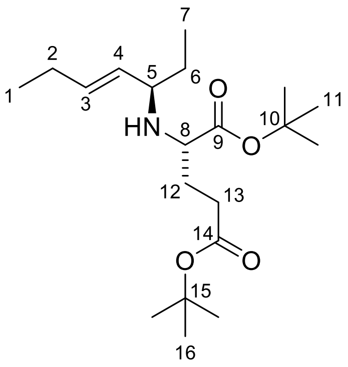

$C_{20}H_{37}NO_4$

TLC: R_f = 0.21 (Silica, cHex/EtOAc = 10/1), $KMnO_4$-reagent.

^{1}H NMR (500 MHz, $CDCl_3$): δ [ppm] = 0.84 (t, ^3J = 7.4 Hz, 3H, H-7); 0.97 (t, ^3J = 7.5 Hz, 3H, H-1); 1.30 – 1.36 (m, 1H, H-6); 1.44 (s, 9H, H-11 o. H-16); 1.45 (s, 9H, H-11 o. H-16); 1.49 – 1.58 (m, 1H, H-6'); 1.61 (s, br, 1H, NH); 1.71 – 1.78 (m, 1H, H-12); 1.83 – 1.90 (m, 1H, H-12'); 1.97 – 2.03 (m, 2H, H-2); 2.28 – 2.38 (m, 2H, H-15); 2.78 (td, ^3J = 8.3 Hz, ^3J = 4.9 Hz, 1H, H-5); 3.14 (dd, ^3J = 7.7 Hz, ^3J = 5.5 Hz, 1H, H-8); 5.15 (ddt, ^3J = 15.3 Hz, ^3J = 8.5 Hz, ^4J = 1.4 Hz, 1H, H-4); 5.52 (dt, ^3J = 15.3 Hz, ^3J = 6.3 Hz, 1H, H-3).

^{13}C NMR (125 MHz, $CDCl_3$): δ [ppm] = 10.5 (C-7); 13.9 (C-1); 25.5 (C-2); 28.2 (C-6); 28.2 (C-11 o. C-16); 28.3 (C-11 o. C-16); 29.0 (C-12); 32.0 (C-13); 58.6 (C-8); 61.7 (C-5); 80.3 (C-10 o. C-15); 81.0 (C-10 o. C-15); 131.8 (C-4); 134.1 (C-3); 172.9 (C-16); 175.1 (C-9).

IR (ATR): $\tilde{\nu}$ [cm^{-1}] = 3445 (w); 3325 (w); 3006 (w); 2968 (w); 2933 (w); 2873 (w); 2850 (w); 1726 (s); 1663 (w); 1475 (w); 1457 (w); 1426 (w); 1392 (w); 1367 (m); 1324 (w); 1293 (w); 1253 (m); 1217 (w); 1147 (s); 1084 (w); 1071 (w); 1037 (w); 1024 (w); 969 (m); 936 (w); 920 (w); 904 (w); 849 (m); 800 (w); 754 (w); 727 (w); 680 (w).

GC/MS (EI, 70 eV): m/z (%) = 355 ($[M]^+$, 2); 326 ($[M]^+$ -C_2H_5, 10); 298 ($[M]^+$ -C_4H_9, 2); 270 (7); 254 ($[M]^+$ -CO_2tBu, 45); 214 (70); 198 (90); 186 (8); 168 (10); 146 (15); 130 (25); 124 (10); 112 (10); 102 (98); 97 ($[C_7H_{13}]^+$, 47); 84 (27); 81 (30); 79 (20); 67 (20); 57 (45); 56 (70); 55 (100)

HR/MS (ESI): calculated for $[M+H]^+$: 356.2795; found: 356.2795;
calculated for $[M+Na]^+$ 378.2615; found: 378.2616.

$[\alpha]_\lambda^{20}$($CHCl_3$, c = 0.510 g/100 ml): $[\alpha]_{436}^{20}$= -17.4°; $[\alpha]_{546}^{20}$ = -10.3°; $[\alpha]_{579}^{20}$= -8.2°; $[\alpha]_{589}^{20}$= -7.2°.

(*S*)-Dimethyl 2-((*S,E*)-hept-4-en-3-ylamino)glutarate ((*S,S*)-3j)

According to **general procedure 1**, 172 mg (1.00 mmol) of carbonate *rac*-**4a** were reacted with L-glutamic acid dimethyl ester ((*S*)-**5j**) using the chiral ligand **L6** to yield 239 mg (0.881 mmol, 87%, *dr = 98/2*) of the allylic amine (*S,S*)-**3j** after purification by flash column chromatography (Silica, cHex/EtOAc = 4/1) as a yellow oil.

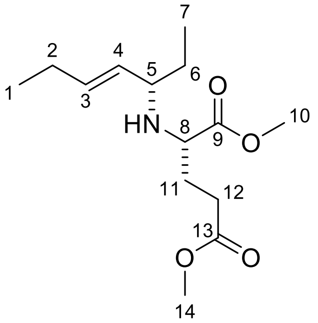

$C_{14}H_{25}NO_4$

M: 271.35 g/mol.

TLC: R_f = 0.21 (Silica, cHex/EtOAc = 4/1), $KMnO_4$-reagent.

^{1}H NMR (500 MHz, $CDCl_3$): δ [ppm] = 0.85 (t, ^3J = 7.5 Hz, 3H, H-7); 0.98 (t, ^3J = 7.5 Hz, 3H, H-1); 1.34 – 1.49 (m, 2H, H-6); 1.75 (s, br, 1H, NH); 1.77 – 1.84 (m, 1H, H-11); 1.93 – 1.99 (m, 1H, H-11'); 2.00 – 2.06 (m, 2H, H-2); 2.37 – 2.49 (m, 2H, H-12); 2.74 (td, ^3J = 8.0 Hz, 3J = 5.9 Hz, 1H, H-5); 3.32 (dd, ^3J = 8.7 Hz, ^3J = 5.3 Hz, 1H, H-8); 3.66 (s, 3H, H-14); 3.72 (s, 3H, H-10); 5.06 (ddt, ^3J = 15.3 Hz, ^3J = 8.6 Hz, ^4J = 1.3 Hz, 1H, H-4); 5.49 (dt, ^3J = 15.3 Hz, ^3J = 6.3 Hz, 1H, H-3).

^{13}C NMR (125 MHz, $CDCl_3$): δ [ppm] = 10.5 (C-7); 14.0 (C-1); 25.4 (C-2); 28.9 (C-11); 29.4 (C-6); 30.9 (C-12); 51.7 (C-14); 51.9 (C-10); 57.7 (C-8); 61.5 (C-5); 131.2 (C-4); 135.3 (C-3); 173.8 (C-13); 176.1 (C-9).

IR (ATR): $\tilde{\nu}$ [cm-1] = 3457 (w); 3330 (w); 2962 (w); 2934 (w); 2876 (w); 2856 (w); 2815 (w); 1734 (s); 1671 (w); 1460 (w); 1436 (m); 1367 (w); 1349 (w); 1326 (w); 1296 (w); 1255 (m); 1197 (s); 1169 (s); 1140 (m); 1068 (w); 1039 (w); 1017 (w); 971 (m); 943 (w); 913 (w); 891 (w); 872 (w); 821 (w); 795 (w); 773 (w); 737 (w); 670 (w).

GC/MS (EI, 70 eV): m/z (%) = 271 ($[M]^+$, 1); 242 ($[M]^+$ -C_2H_5, 100); 212 ($[M]^+$ -CO_2Me, 20); 182 (10); 159 (6); 150 (8); 144 (12); 122 (6); 116 (25); 97 ($[C_7H_{13}]^+$, 18); 84 (20); 81 (7); 67 (10); 55 (45).

HR/MS (ESI): calculated for $[M+H]^+$: 272.1856; found: 272.1856;
calculated for $[M+Na]^+$ 294.1676; found: 294.1678.

$[\alpha]_\lambda^{20}$(CHCl3, c = 0.505 g/100 ml): $[\alpha]_{365}^{20}$= -120.5°; $[\alpha]_{436}^{20}$= -83.6°; $[\alpha]_{546}^{20}$ = -51.4°; $[\alpha]_{579}^{20}$= -44.4°; $[\alpha]_{589}^{20}$= -41.9°.

(*S*)-Dimethyl 2-((*R,E*)-hept-4-en-3-ylamino)glutarate ((*R,S*)-3j)

According to **general procedure 1**, 172 mg (1.00 mmol) of carbonate *rac*-**4a** were reacted with L-glutamic acid dimethyl ester ((*S*)-**5j**) using the chiral ligand *ent*-**L6** to yield 232 mg (0.856 mmol, 85%, *dr = 99/1*) of the allylic amine ((*R,S*)-**3j**) after purification by flash column chromatography (Silica, cHex/EtOAc = 4/1) as a yellow oil.

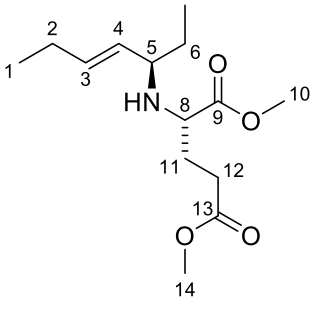

$C_{14}H_{25}NO_4$

TLC: R_f = 0.21 (Silica, cHex/EtOAc = 4/1), $KMnO_4$-reagent.

^{1}H NMR (500 MHz, $CDCl_3$): δ [ppm] = 0.84 (t, ^3J = 7.4 Hz, 3H, H-7); 0.96 (t, ^3J = 7.5 Hz, 3H, H-1); 1.28 – 1.37 (m, 1H, H-6); 1.47 – 1.55 (m, 1H, H-6'); 1.64 (s, br, 1H, NH); 1.82 – 1.89 (m, 1H, H-11); 1.91 – 2.03 (m, 3H, H-2/11'); 2.37 – 2.48 (m, 2H, H-12); 2.77 (td, ^3J = 8.3 Hz, ^3J = 5.2 Hz, 1H, H-5); 3.27 (dd, ^3J = 7.4 Hz, ^3J = 5.7 Hz, 1H, H-8); 3.67 (s, 3H, H-14); 3.69 (s, 3H, H-10); 5.09 (ddt, ^3J = 15.3 Hz, ^3J = 8.8 Hz, ^4J = 1.5 Hz, 1H, H-4); 5.51 (dt, ^3J = 15.3 Hz, ^3J = 6.3 Hz, 1H, H-3).

^{13}C NMR (125 MHz, $CDCl_3$): δ [ppm] = 10.6 (C-7); 13.8 (C-1); 25.4 (C-2); 28.5 (C-6/11); 30.4 (C-12); 51.7 (C-10 o. C-14); 51.9 (C-10 o. C-14); 58.2 (C-8); 62.0 (C-5); 131.5 (C-4); 134.5 (C-3); 173.9 (C-13); 176.1 (C-9).

IR (ATR): $\tilde{\nu}$ [cm^{-1}] = 3434 (w, br); 3321 (w); 3026 (w); 2962 (m); 2934 (m); 2876 (m); 2853 (w); 1737 (s); 1668 (w); 1459 (m); 1435 (m); 1402 (w); 1376 (w); 1335 (m); 1265 (m); 1252 (m); 1198 (s); 1174 (s); 1123 (m); 1062 (s); 1031 (m); 971 (s); 912 (w); 892 (w); 854 (w); 790 (m); 765 (m); 733 (m); 696 (m); 661 (m); 646 (m).

GC/MS (EI, 70 eV): m/z (%) = 186 ([M]$^+$ -C_2H_5, 100); 156 ([M]$^+$ -CO_2Me, 12); 126 (25); 112 ([$C_7H_{14}N$]$^+$, 10); 97 ([C_7H_{13}]$^+$, 50); 88 (20); 81 (12); 67 (17); 60 (27); 55 (80).

HR/MS (ESI): calculated for [M+H]$^+$: 216.1594; found: 226.1592;
calculated for [M+Na]$^+$ 238.1414; found: 238.1415.

$[\alpha]_\lambda^{20}$(CHCl$_3$, c = 0.510 g/100 ml): $[\alpha]_{365}^{20}$= -163.4°; $[\alpha]_{436}^{20}$= -100.6°; $[\alpha]_{546}^{20}$ = -57.7°; $[\alpha]_{579}^{20}$= -50.5°; $[\alpha]_{589}^{20}$= -48.6°.

(*S*)-Methyl 1-((*S*,*E*)-hept-4-en-3-yl)pyrrolidin-2-carboxylate ((S,S)-3k)

According to **general procedure 1**, 172 mg (1.00 mmol) of carbonate *rac*-**4a** were reacted with L-proline methyl ester ((*S*)-**5k**) using the chiral ligand **L6** to yield 190 mg (0.843 mmol, 84%, *dr = 99/1*) of the allylic amine (*S,S*)-**3k** after purification by flash column chromatography (Silica, cHex/EtOAc = 3/1) as a yellow oil.

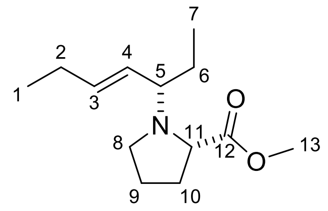

$C_{13}H_{23}NO_2$

M: 225.33 g/mol.

TLC: R_f = 0.24 (Silica, cHex/EtOAc = 3/1), $KMnO_4$-reagent.

^{1}H NMR (500 MHz, $CDCl_3$): δ [ppm] 0.82 (t, ^3J = 7.4 Hz, 3H, H-7); 0.99 (t, ^3J = 7.5 Hz, 3H, H-1); 1.33 – 1.42 (m, 1H, H-6); 1.62 – 1.70 (m, 1H, H-6'); 1.72 – 1.79 (m, 1H, H-9); 1.83 – 1.92 (m, 2H, H-9'/10); 2.00 – 2.09 (m, 3H, H-10'/2); 2.61 – 2.66 (m, 1H, H-8); 2.90 (td, ^3J = 9.4 Hz, ^3J = 4.4 Hz, 1H, H-5); 3.01 – 3.05 (m, 1H, H-8'); 3.44 (dd, ^3J = 9.1 Hz, ^3J = 4.6 Hz, 1H, H-11); 3.69 (s, 3H, H-13); 5.27 (ddt, ^3J = 15.3 Hz, ^3J = 9.3 Hz, ^4J = 1.5 Hz, 1H, H-4); 5.52 (dt, ^3J = 15.3 Hz, ^3J = 6.3 Hz, 1H, H-3).

^{13}C NMR (125 MHz, $CDCl_3$): δ [ppm] = 11.1 (C-7); 14.0 (C-1); 23.4 (C-9); 25.6 (C-2); 27.1 (C-6); 29.6 (C-10); 48.6 (C-8); 51.7 (C-13); 62.8 (C-5); 65.9 (C-11); 127.9 (C-4); 136.2 (C-3); 175.7 (C-12).

IR (ATR): $\tilde{\nu}$ [cm^{-1}] = 2962 (m); 2932 (m); 2874 (w); 2846 (w); 1735 (s); 1661 (w); 1459 (m); 1435 (m); 1374 (w); 1360 (w); 1343 (w); 1276 (w); 1193 (s); 1165 (s); 1106 (m); 1067 (w); 1054 (w); 1037 (w); 1019 (w); 999(w); 973 (m); 935 (w); 910 (w); 877 (w); 836 (w); 793 (w); 758 (w); 714 (w); 669 (w); 638 (w).

GC/MS (EI, 70 eV): m/z (%) = 225 ([M]$^+$, 1); 196 ([M]$^+$ -C_2H_5, 100); 166 ([M]$^+$ -CO_2Me, 28); 136 (10); 122 (5); 108 (8); 97 ([C_7H_{13}]$^+$, 15); 81 (10); 70 (90); 55 (48).

HR/MS (ESI): calculated for [M+H]$^+$: 226.1802; found: 226.1798;
calculated for [M+Na]$^+$ 248.1621; found: 248.1621.

$[\alpha]_\lambda^{20}$(CHCl$_3$, c = 0.520 g/100 ml): $[\alpha]_{436}^{20}$= -130.5°, $[\alpha]_{546}^{20}$ = -79.7°; $[\alpha]_{579}^{20}$= -70.6°; $[\alpha]_{589}^{20}$= -68.1°.

(*S*)-Methyl 1-((*R,E*)-hept-4-en-3-yl)pyrrolidin-2-carboxylate ((*R,S*)-3k)

According to **general procedure 1**, 172 mg (1.00 mmol) of carbonate *rac*-**4a** were reacted with L-proline methyl ester ((*S*)-**5k**) using the chiral ligand *ent*-**L6** to yield 197 mg (0.874 mmol, 88%, *dr = 98/2*) of the allylic amine (*R,S*)-**3k** after purification by flash column chromatography (Silica, cHex/EtOAc = 3/1) as a yellow oil.

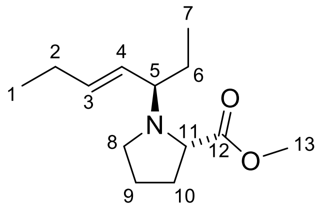

$C_{13}H_{23}NO_2$

M: 225.33 g/mol.

TLC: R_f = 0.18 (Silica, cHex/EtOAc = 3/1), $KMnO_4$-reagent.

^{1}H NMR (500 MHz, CDCl$_3$): δ [ppm] 0.81 (t, ^3J = 7.4 Hz, 3H, H-7); 0.98 (t, ^3J = 7.5 Hz, 3H, H-1); 1.35 – 1.42 (m, 1H, H-6); 1.69 – 1.76 (m, 1H, H-6'); 1.77 – 1.97 (m, 3H, H-9/10); 1.97 – 2.05 (m, 2H, H-2); 2.06 – 2.14 (m, 1H, H-10'); 2.46 – 2.50 (m, 1H, H-8); 2.66 – 2.70 (m, 1H, H-5); 3.20 – 3.23 (m, 1H, H-8'); 3.32 – 3.38 (m, 1H, H-11); 3.68 (s, 3H, H-13); 5.22 (dd, ^3J = 15.3 Hz, 3J = 9.1 Hz, 1H, H-4); 5.59 (dt, ^3J = 15.3 Hz, ^3J = 6.2 Hz, 1H, H-3).

^{13}C NMR (125 MHz, CDCl$_3$): δ [ppm] = 11.0 (C-7); 13.6 (C-1); 23.7 (C-9); 25.4 (C-2); 26.6 (C-6); 30.4 (C-10); 51.8 (C-13); 52.3 (C-8); 63.9 (C-5); 69.5 (C-11); 129.8 (C-4); 135.8 (C-3); 176.1 (C-12).

IR (ATR): $\tilde{\nu}$ [cm^{-1}] = 2962 (m); 2934 (m); 2875 (w); 2848 (w); 2785 (w); 1753 (m); 1733 (s); 1671 (w); 1459 (m); 1435 (m); 1376 (w); 1360 (w); 1343 (w); 1275 (m); 1192 (m); 1162 (s); 1105 (m); 1086 (m); 1057 (w); 1036 (w); 1001 (w); 973 (m); 932 (w); 910 (w); 864 (w); 839 (w); 790 (w); 752 (w); 733 (w); 699 (w); 674 (w); 646 (w); 620 (w).

GC/MS (EI, 70 eV): m/z (%) = 225 ([M]$^+$, 1); 196 ([M]$^+$ -C_2H_5, 100); 166 ([M]$^+$ -CO_2Me, 20); 136 (10); 122 (5); 108 (7); 97 ([C_7H_{13}]$^+$, 10); 81 (10); 70 (45); 55 (30).

HR/MS (ESI): calculated for [M+H]$^+$: 226.1802; found: 226.1799;

calculated for [M+Na]$^+$ 248.1621; found: 248.1622.

$[\alpha]_\lambda^{20}$(CHCl$_3$, c = 0.505 g/100 ml): $[\alpha]_{436}^{20}$= -159.3°; $[\alpha]_{546}^{20}$ = -92.5°; $[\alpha]_{579}^{20}$= -80.8°; $[\alpha]_{589}^{20}$= -78.4°.

General procedure 2: Peptide coupling using *Ghosez* reagent

Under inert conditions 1.1 eq. *Ghosez* reagent was added to a solution 1.0 eq. *Zaminer's* acid (**2**) in CH_2Cl_2 (0.95 ml/mmol) at 0 °C over 1 h. Afterwards 1.1 eq. DIPEA and a solution of 1.1 eq. of amine **3** in CH_2Cl_2 (0.85 ml/mmol) was added simultaneously to the solution at 0 °C. The reaction solution was then stirred at r.t. over night. During this process the yellow solution turned to orange. After completion the solution was washed with citric acid solution (10 vol%, 22 ml/mmol *Zaminer's acid*) and the aqueous phase was extracted three times using CH_2Cl_2. The combined organic phases were washed with sat. $NaHCO_3$ solution and sat. NaCl solution and dried over $MgSO_4$. After filtration the solvent was removed under reduced pressure and the resulting yellow raw product was purified by flash column chromatography.

General procedure 3: Ru-catalyzed ring closing metathesis

Under inert conditions a solution of 1.0 eq. of the dipeptide and 4 mol% of the *Hoveyda-Grubbs* II catalyst (**9**) in C_6F_6 (10 ml/mmol, 0.1 M) was warmed to 70 °C and stirred for 3.5 h before another 2 mol% of *Hoveyda-Grubbs* II catalyst (**9**) were added. (Note: the mixture turns dark upon heating). After complete conversion the solution was allowed to cool down before the solvent was removed under reduced pressure. The resulting dark green oil was purified by flash column chromatography. The resulting oily product was dissolved in little CH_2Cl_2 and stirred over *QuadraSil AP* for 15 min to remove residual ruthenium. After filtration the solvent was removed under reduced pressure and the product was dried in high vacuum to afford the desired bicyclic product.

Synthesis of (2*S*,3*R*)-*tert*-Butyl 2-((2-(*tert*-butoxy)-2-oxoethyl)-((*R*,*E*)-hept-4-en-3-yl) carbamoyl)-3-vinylpyrrolidine-1-carboxylate (6a)

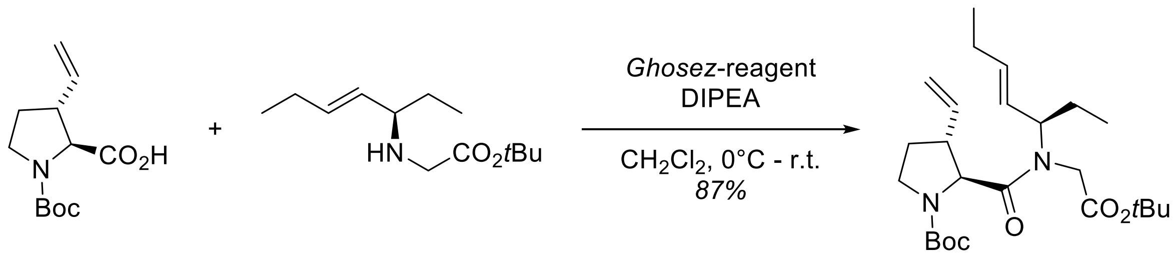

2 (*R*)-**3a** **6a**

The reaction was performed according to the **general procedure 2** using 119 mg (0.495 mmol) of *Zaminer's acid* (**2**) in combination with amine (*R*)-**3a** and stopped after 18.5 h.

The crude product was purified by flash column chromatography (Silica, cHex/EtOAc = 4/1) to yield 152 mg (0.305 mmol, 62%) of the desired dipeptide **6a** as a colourless viscose oil.

$C_{25}H_{42}N_2O_5$

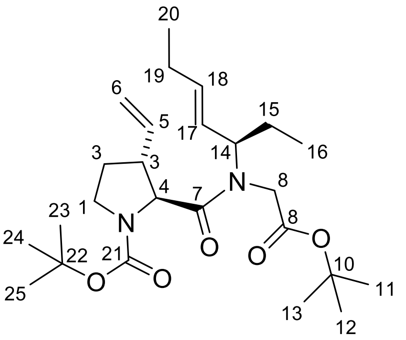

M: 450.61 g/mol.

TLC: R_f = 0.19 (Silica, cHex/EtOAc = 5/1), $KMnO_4$-reagent.

^{1}H NMR (500 MHz, $CDCl_3$, rotameric mixture[1]): δ [ppm] = 0.88 – 1.01 (m, 6H, H-16/20); 1.36 – 1.54 (m, 18.6H, H-11/12/13/15$_{rot3+4}$/15'$_{rot3+4}$/23/24/25); 1.61 – 1.79 (m, 2.4H, H-15$_{rot1+2}$/15'$_{rot1+2}$/2); 1.99 – 2.10 (m, 2H, H-19); 2.12 – 2.19 (m, 0.35H, H-2'$_{rot1}$); 2.20 – 2.27 (m, 0.35H, H-2'$_{rot2}$); 2.45 – 2.53 (m, 0.15H, H-2'$_{rot3}$); 2.58 – 2.66 (m, 0.15H, H-2'$_{rot4}$); 2.93 – 2.96 (m, 0.7H, H-3$_{rot1+2}$); 3.01 – 3.07 (m, 0.3H, H-3$_{rot3+4}$); 3.39 – 3.50 (m, 1.35H, H-1/8$_{rot1}$); 3.53 – 3.65 (m, 0.85H, H-1'$_{rot1+3}$/8$_{rot2}$); 3.69 – 3.75 (m, 0.8H, H-1'$_{rot2+4}$/8$_{rot3}$/8'$_{rot3}$); 3.98 (d, ^2J = 16.7 Hz, 0.35H, H-8'$_{rot2}$); 4.06 – 4.20 (m, 1.5H, H-4$_{rot3+4}$/8'$_{rot1}$/8$_{rot4}$/14$_{rot1+2}$); 4.45 (s, 0.35H, H-4$_{rot1}$); 4.61 (s, 0.35H, H-4$_{rot2}$); 4.71 (d, ^2J = 19.1 Hz, 0.15H, H-8'$_{rot4}$); 4.95 – 5.05 (m, 0.9H, H-6$_{rot3+4}$/6'$_{rot3+4}$/14$_{rot3+4}$); 5.07 – 5.19 (m, 1.4H, H-6$_{rot1+2}$/6'$_{rot1+2}$); 5.27 – 5.37 (m, 0.3H, H-17$_{rot3+4}$); 5.38 – 5.44 (m, 0.7H, H-17$_{rot1+2}$); 5.60 – 5.76 (m, 1H, H-18); 5.77 – 5.85 (m, 0.3H, H-5$_{rot3+4}$); 5.87 – 5.95 (m, 0.7H, H-5$_{rot1+2}$).

^{13}C NMR (125 MHz, $CDCl_3$, rotameric mixture[1]): δ [ppm] = 11.2, 11.3, 11.3 (C-16); 13.5, 13.5, 13.6, 13.7 (C-20); 24.6, 24.9 (C-15$_{rot3+4}$); 25.6, 25.7 (C-19); 26.1, 26.2 (C-15$_{rot1+2}$); 28.1, 28.2, 28.2, 28.2 (C-11/12/13 o. C-23/24/25); 28.6, 28.7 (C-11/12/13 o. C-23/24/25); 28.5, 28.8, 29.8, 29.9 (C-2); 44.5, 44.6 (C-8$_{rot1+2}$); 45.4, 45.6, 45.8, 46.0 (C-1); 46.0, 46.5 (C-8$_{rot3+4}$); 46.1, 46.3, 47.1, 47.3 (C-3); 57.0, 57.1, 59.4, 59.4 (C-14); 61.3, 61.5, 62.0, 62.6 (C-4); 79.4, 79.6, 80.0, 80.1 (C-10 o. C-22); 81.1, 81.1, 81.8, 82.1 (C-10 o. C-22); 114.3, 114.6, 114.9, 115.0 (C-6); 126.1, 126.3, 126.4, 126.6 (C-17); 134.9, 135.8, 135.9, 136.5 (C-18); 139.1, 139.1, 139.5, 139.6 (C-5); 153.9, 154.5, 154.6, 154.7 (C-21); 168.4, 168.7, 169.0, 169.8 (C-9); 171.3, 171.5, 173.1, 174.0 (C-7).

IR (ATR): $\tilde{\nu}$ [cm^{-1}] = 3081 (w); 3005 (w); 2972 (w); 2933 (w); 2879 (w); 1746 (m); 1700 (m); 1657 (m); 1479 (w); 1452 (w); 1435 (w); 1392 (s); 1366 (s); 1308 (w); 1256 (m); 1221 (m); 1150 (s); 1117 (m); 1071 (w); 1056 (w); 992 (w); 973 (w); 945 (w); 912 (m); 866 (w); 845 (w); 820 (w); 795 (w); 771 (w); 754 (w); 737 (w); 694 (w); 661 (w).

HR/MS (ESI): calculated for $[M+H]^+$: 451.3166; found: 451.3175;
calculated for $[M+Na]^+$ 473.2986; found: 473.2988.

$[\boldsymbol{\alpha}]_{\lambda}^{\mathbf{20}}$ ($CHCl_3$, c = 0.510 g/100 ml): $[\alpha]_{365}^{20}$= +297.8°; $[\alpha]_{436}^{20}$= +174.2°; $[\alpha]_{546}^{20}$ = +97.0°; $[\alpha]_{579}^{20}$= +83.9°; $[\alpha]_{589}^{20}$= +80.3°.

[1] The substance forms four rotamers: Two main rotamers (1 & 2) in 1:1 ratio and two side rotamers (3 & 4) in 1:1 ratio. The ratio between main and side rotamers is around 0.7:0.3.

Synthesis of (2*R*,3*S*)-*tert*-Butyl 2-((2-(*tert*-butoxy)-2-oxoethyl)-((*R*,*E*)-hept-4-en-3-yl)carbamoyl)-3-vinyl pyrrolidine-1-carboxylate (*dia*-6a)

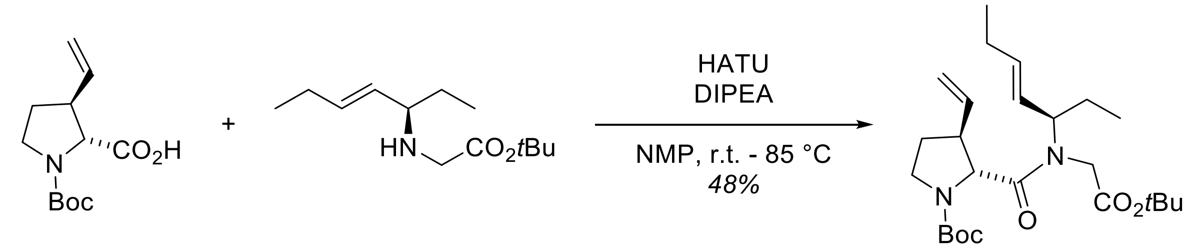

ent-**2** (*R*)-**3a** *dia*-**6a**

To a solution of 96 mg (0.40 mmol, 1.1 eq.) (*R,S*)-*Zaminer's acid* (*ent*-**2**) and 167 mg (0.44 mmol, 1.1 eq.) HATU in 2.2 ml dry NMP, 0.15 ml (0.86 mmol, 1.95 eq.) DIPEA were added at r.t. under inert conditions and the solution was stirred for 20 min. 100 mg (0.44 mmol, 1.1 eq.) of Amine (*R*)-**3a** were added and the reaction was heated to 85 °C for 43 h. After cooling the reaction mixture to r.t. 3.3 ml citric acid solution (10 vol%) were added and the solution was extracted four times using MTBE. The combined organic phases were washed twice with 1 m HCl and once with sat. $NaHCO_3$ solution and sat. NaCl solution each, dried over $MgSO_4$ and the solvent was removed under reduced pressure. The yellow high viscose crude product was purified by flash column chromatography (Silica, cHex/EtOAc = 5/1) yielding clean product *dia*-**6a** as a pale-yellow viscose oil in 48% yield. Furthermore 19 mg (0.042, 11%) of the product could be isolated which still contained traces of the amine (*R*)-**3a**.

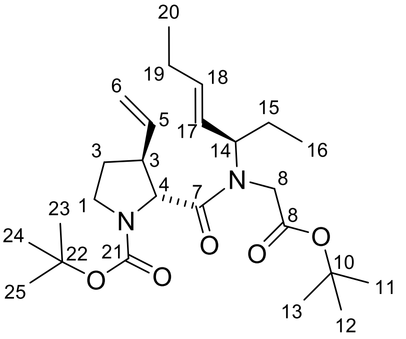

$C_{25}H_{42}N_2O_5$

M: 450.61 g/mol.

TLC: R_f = 0.20 (Silica, cHex/EtOAc = 5/1), $KMnO_4$-reagent.

^{1}H NMR (500 MHz, CDCl3, rotameric mixture[2]): δ [ppm] = 0.82 – 1.00 (m, 6H, H-16/20); 1.39 – 1.80 (m, 21H, H-2/11/12/13/15/23/24/25); 1.96 – 2.10 (m, 2H, H-19); 2.15 – 2.24 (m, 0.35H, H-2'$_{rot1}$); 2.25 – 2.33 (m, 0.25H, H-2'$_{rot2}$); 2.47 – 2.54 (m, 0.15H, H-2'$_{rot4}$); 2.65 – 2.73 (m, 0.25H, H-2'$_{rot3}$); 2.89 – 2.95 (m, 0.6H, H-3$_{rot1+2}$); 3.01 – 3.08 (m, 0.4H, H-3$_{rot3+4}$); 3.38 – 3.52 (m, 1.6H, H-1/8$_{rot1+3}$); 3.57 – 3.71 (m, 1.4H, H-1'/8$_{rot2+4}$); 4.03 – 4.15 (m, 1.5H, H-4$_{rot3+4}$/8'$_{rot1+3+4}$/14$_{rot1}$); 4.24 – 4.27 (m, 0.25H, H-14$_{rot3}$); 4.45 (d, ^3J = 1.6 Hz, 0.35H, H-4$_{rot1}$); 4.61 (d, ^3J = 1.6 Hz, 0.25H, H-4$_{rot2}$); 4.68 (d, ^2J = 19.0 Hz, 0.25H, H-8'$_{rot2}$); 4.77 (Ψ q, J 6.8 Hz,0.15H, H-14$_{rot4}$); 4.96 – 5.19 (m, 2.25H, H-6/14$_{rot2}$); 5.32 – 5.42 (m, 1H, H-17); 5.59 – 5.74 (m, 1H, H-18); 5.76 – 5.95 (m, 1H, H-5).

^{13}C NMR (125 MHz, $CDCl_3$, rotameric mixture[2]): δ [ppm] = 10.3, 11.2, 11.3, 11.4 (C-16); 13.5, 13.5, 13.6 (C-20); 23.9, 24.5, 25.3, 25.5 (C-15); 25.6, 25.7, 25.7, 25.7 (C-19); 28.2, 28.2 (C-11/12/13 o. C-23/24/25); 28.6, 28.6, 8.6, 28.7 (C-11/12/13 o. C-23/24/25); 29.4, 29.8, 30.3 (C-2); 44.7, 45.0, 45.4, 45.5, 45.7, 45.8, 45.9, 46.3 (C-1/8); 45.9,

[2] The substance forms four rotamers: Two main rotamers (1 & 2) in 0.6:0.4 ratio and two side rotamers (3 & 4) in 0.65:0.35 ratio. The ratio between main and side rotamers is around 0.6:0.4.

46.7, 47.2, 47.8 (C-3); 55.7, 58.0, 59.4, 59.7 (C-14); 61.3, 61.5, 61.6, 62.4 (C-4); 79.4, 79.6, 79.8, 80.2 (C-10 o. C-22); 81.1, 81.3, 81.6, 82.1 (C-10 o. C-22); 114.1, 114.5, 115.1, 115.3 (C-6); 126.0, 126.1, 126.5, 127.3 (C-17); 135.2, 135.3, 135.7, 136.4 (C-18); 139.0, 139.1, 139.5, 139.7 (C-5); 153.8, 154.3, 154.5, 154.7 (C-21); 168.5, 168.8, 168.9, 169.7 (C-9); 171.4, 171.9, 173.0, 173.9 (C-7).

IR (ATR): $\tilde{\nu}$ [cm^{-1}] = 3079 (w); 2972 (w); 2934 (w); 2879 (w); 1746 (m); 1700 (m); 1692 (m); 1655 (m); 1478 (w); 1442 (m); 1392 (s); 1366 (s); 1304 (w); 1256 (m); 1222 (m); 1150 (s); 1117 (m); 1071 (w); 1058 (w); 1043 (w); 1011 (w); 990 (w); 975 (w); 938 (w); 910 (m); 865 (w); 845 (w); 823 (w); 797 (w); 771 (w); 753 (w); 701 (w); 657 (w).

HR/MS (ESI): calculated for $[M+H]^+$: 451.3166; found: 451.3169;
calculated for $[M+Na]^+$ 473.2986; found: 473.2983.

$[\alpha]_{\lambda}^{20}$ **(XX):** ($CHCl_3$, c = 0.510 g/100 ml):): $[\alpha]_{365}^{20}$= -18.5°; $[\alpha]_{436}^{20}$= -12.9°; $[\alpha]_{546}^{20}$ = -7.7°.

Synthesis of (3aR,6R,8aS)-tert-Butyl 7-(2-(tert-Butoxy)-2-oxoethyl)-6-ethyl-8-oxo-3,3a,6,7,8,8a-hexahydro pyrrolo[2,3-c]azepin-1(2H)-carboxylate ((*R,S,R*)-7a)

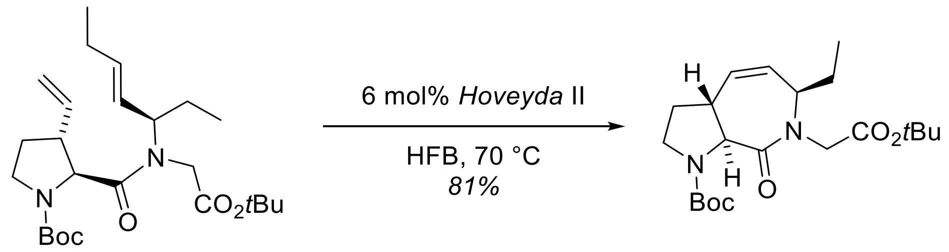

6a (*R,S,R*)-**7a**

Under inert conditions a solution of 375 mg (0.832 mmol 1.0 eq.) of the dipeptide **6a** and 20.9 mg (33 µmol, 4 mol%) of the *Hoveyda-Grubbs II* catalyst (**9**) in 8.3 ml C_6F_6 (0.1 M) was warmed to 70 °C and stirred for 3.5 h before another 10.4 mg (16.5 µmol, 2 mol%) of *Hoveyda-Grubbs II* catalyst (**9**) were added. (Note: the mixture turns dark upon heating). After 6 h the solution was allowed to cool down before the solvent was removed under reduced pressure. The resulting dark green oil was purified by flash column chromatography (SiO_2, cHex/EtOAc = 1/1). The resulting oily product was dissolved in little CH_2Cl_2 and stirred over *QuadraSil AP* for 15 min to remove residual ruthenium. After filtration the solvent was removed under reduced pressure and the product was dried in high vacuum to afford 266 mg (0.674, 81%) of the desired bicyclic product (*R,S,R*)-**7a**.

$C_{21}H_{34}N_2O_5$

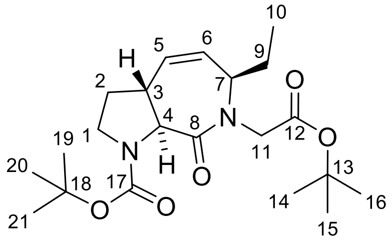

M: 394.51 g/mol.

TLC: R_f = 0.20 (Silica, cHex/EtOAc = 1/1), $KMnO_4$-reagent.

Mp: 108 – 112 °C.

^{1}H NMR (600 MHz, $CDCl_3$, rotameric mixture[3]): δ [ppm] = 1.01 (t, ^3J = 7.2 Hz, 1.5H, H-10_{rot1}); 1.04 (t, ^3J = 7.1 Hz, 1.5H, H-10_{rot2}); 1.42 – 1.54 (m, 18H, H-14/15/16/19/20/21); 1.54 – 1.62 (m, 1H, H-9); 1.63 – 1.71 (m, 1H, H-2); 1.73 – 1.86 (m, 1H, H-9'); 2.05 (Ψ dt, J = 11.5 Hz, J = 5.7 Hz, 1H, H-9'); 2.89 – 3.00 (m, 1H, H-3); 3.44 (dtd, ^2J = 16.5 Hz, ^3J = 11.3 Hz, ^4J = 5.6 Hz, 1H, H-1); 3.59 (d, ^2J = 17.6 Hz, 0.5H, H-11_{rot1}); 3.67 – 3.70 (m, 0.5H, H-1'rot1); 3.74 – 3.77 (m, 0.5H, H-$1'_{rot2}$); 3.94 (d, ^2J = 17.4 Hz, 0.5H, H-11_{rot2}); 4.25 (d, ^2J = 17.4 Hz, 0.5H, H-$11'_{rot2}$); 4.57 – 4.62 (m, 0.5H, H-7_{rot1}); 4.65 – 4.70 (m, 0.5H, H-7_{rot2}); 4.81 (d, ^2J = 17.6 Hz, 0.5H, H-$11'_{rot1}$); 4.85 (d, ^3J = 11.8 Hz, 0.5H, H-4_{rot1}); 4.91 (d, ^3J = 11.8 Hz, 0.5H, H-4_{rot2}); 5.56 – 5.58 (m, 1H, H-6); 5.84 (Ψ t, J = 9.5 Hz, 1H, H-5).

^{13}C NMR (150 MHz, $CDCl_3$, rotameric mixture[3]): δ [ppm] = 11.8, 11.9 (C-10); 26.1 (C-9); 28.1 (C-14/15/16 o. C-19/20/21); 28.3, 28.6 (C-14/15/16 o. C-19/20/21); 30.3, 30.8 (C-2); 43.2, 43.8 (C-3); 44.2, 44.5 (C-11); 46.7, 47.3 (C-1); 55.4, 55.6 (C-7); 60.3, 60.5 (C-4); 79.8, 79.9 (C-13 o. C-18); 81.4 (C-13 o. C-18); 130.8 (C-5_{rot1}); 130.9 (C-6); 131.3 (C-5_{rot2}); 154.5, 155.0 (C-17); 169.1, 169.3 (C-12); 172.4, 172.8 (C-8).

IR (ATR): $\tilde{\nu}$ [cm^{-1}] = 3022 (w); 2970 (w); 2931 (w); 2879 (w); 2855 (w); 1745 (s); 1691 (s); 1670 (s); 1573 (w); 1535 (w); 1477 (w); 1457 (w); 1429 (m); 1404 (m); 1390 (s); 1364 (m); 1335 (m); 1306 (w); 1277 (w); 1260 (m); 1247 (m); 1220 (m); 1153 (s); 1127 (s); 1116 (m); 1101 (m); 1050 (w); 1033 (w); 975 (w); 962 (w); 951 (w); 937 (w); 925 (w); 917 (w); 905 (w); 867 (w); 845 (m); 823 (w); 786 (m); 762 (w); 753 (m); 730 (w); 686 (w); 675 (w); 630 (w); 607 (w).

GC/MS (EI, 70 eV): m/z (%) = 394 ($[M]^+$, 1); 338 (3); 321 ($[M]^+$ -C_4H_9O, 13); 309 (4); 294 (20); 282 (30); 265 (37); 253 (18); 238 (40); 237 (40); 225 (13); 209 (38); 195 (16); 180 (48); 165 (13); 151 (8); 136 (46); 123 (30); 116 (36); 108 (20); 94 (14); 80 (18); 69 (21); 67 (21); 57 (100); 41 (80).

HR/MS (ESI): calculated for $[M+H]^+$: 395.2540; found: 395.2545;
calculated for $[M+Na]^+$ 417.2360; found: 417.2361.

$[\alpha]_\lambda^{20}$ ($CHCl_3$, c = 0.540 g/100 ml): $[\alpha]_{365}^{20}$ = -546.2°; $[\alpha]_{436}^{20}$ = -344.0°; $[\alpha]_{546}^{20}$ = -200.4°; $[\alpha]_{579}^{20}$ = -175.7°; $[\alpha]_{589}^{20}$= -169.3°.

[3] The substance forms two rotamers in 1:1 ratio.

Synthesis of (2*R*,3*S*)-*tert*-Butyl 2-((*R*,*E*)-hept-4-en-3-yl((*S*)-1-methoxy-1-oxo-3-phenylpropane-2-yl)-carbamoyl)-3-vinylpyrrolidine-1-carboxylate ((*S,R,R,S*)-6b)

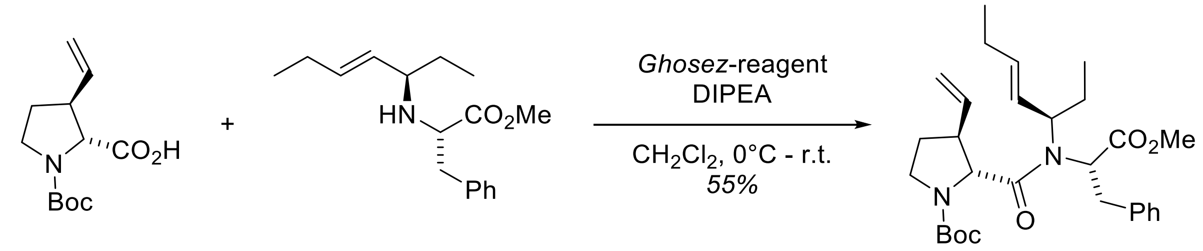

ent-**2** (*R,S*)-**3e** (*S,R,R,S*)-**6b**

The reaction was performed according to the **general procedure 2** using 80 mg (0.330 mmol) (*R,S*)-*Zaminer's acid* (*ent*-**2**) and stopped after 17 h.
The crude product was purified by flash column chromatography (Silica, cHex/EtOAc = 4/1) to yield 104 mg (0.209 mmol, 63%) of the desired dipeptide (*R,S,R,S*)-**6b** as a pale-yellow viscose oil.

$C_{29}H_{42}N_2O_5$

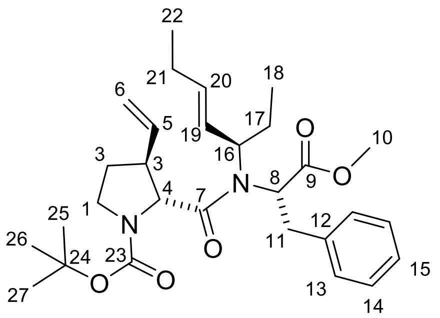

M: 489.65 g/mol.

TLC: R_f = 0.17 (Silica, cHex/EtOAc = 4/1), $KMnO_4$-reagent.

^{1}H NMR (600 MHz, $CDCl_3$, rotameric mixture[4]): δ [ppm] = 0.86 – 0.92 (m, 4.2H, H-18/22$_{rot2}$/22'$_{rot2}$); 0.98 (t, ^3J = 7.5 Hz, 1.8H, H-22$_{rot1}$/22'$_{rot1}$); 1.44, 1.48 (2 × s, 9H, H-25/26/27); 1.62 – 1.92 (m, 3.8H, H-2/17/21$_{rot2}$/21'$_{rot2}$); 2.01 – 2.08 (m, 1.2H, H-21$_{rot1}$/21'$_{rot1}$); 2.13 – 2.19 (m, 0.6H, H-2'$_{rot1}$); 2.27 – 2.23 (m, 0.4H, H-2'$_{rot2}$); 2.81 (dd, ^2J = 14.1 Hz, 3J = 3.2 Hz, 0.6H, H-11$_{rot1}$); 2.85 – 2.90 (m, 1H, H-3); 3.14 (dd, ^3J = 14.2 Hz, 3J = 6.0 Hz, 0.4H, H-11$_{rot2}$); 3.45 – 3.52 (m, 1H, H-1); 3.60 – 3.73 (m, 4.4H, H-1'/10/11'$_{rot2}$); 3.78 (Ψ t, *J* = 6.4 Hz, 0.4H, H-8$_{rot2}$); 3.84 (dd, ^3J = 8.4 Hz, ^3J = 3.2 Hz, 0.6H, H-8$_{rot1}$); 3.92 (dd, 2J = 14.1 Hz, ^3J = 8.4 Hz, 0.6H, H-11'$_{rot1}$); 4.04 – 4.08 (m, 0.6H, H-16$_{rot1}$); 4.20 – 4.23 (m, 0.4H, H-16$_{rot2}$); 4.48 (s, 0.6H, H-4$_{rot1}$); 4.58 (d, ^4J = 2.2 Hz, 0.4H, H-4$_{rot2}$); 4.86 (dd, ^3J = 15.6 Hz, ^3J = 6.4 Hz, 0.4H, H-19$_{rot2}$); 5.02 – 5.23 (m, 2.6H, H-6/19$_{rot2}$); 5.57 (dt, ^3J = 14.5 Hz, 3J = 6.2 Hz, 0.4H, H-20$_{rot2}$); 5.71 (dt, ^3J = 15.1 Hz, ^3J = 6.2 Hz, 0.6H, H-20$_{rot1}$); 5.83 – 5.95 (m, 1H, H-5); 7.16 – 7.23 (m, 1H, H-15); 7.26 – 7.30 (m, 4H, H-13/14).

^{13}C NMR (150 MHz, $CDCl_3$, rotameric mixture[5]): δ [ppm] = 11.2, 11.3 (C-18); 13.4, 13.5 (C-22); 25.2, 25.4, 25.6, 25.8 (C-17/21); 28.4, 28.5 (C-25/26/27); 29.2, 30.6 (C-2); 36.6, 38.2 (C-11); 45.5, 45.9 (C-1); 46.2, 47.1 (C-3); 52.2, 52.3 (C-10); 59.3, 59.4 (C-8); 60.5, 60.9 (C-16); 61.7, 62.5 (C-4); 79.6, 79.8 (C-24); 115.3, 115.3 (C-6); 126.3, 126.4, 126.5, 126.7 (C-15/19); 128.3, 128.5 (C-14); 129.6, 130.1 (C-13); 136.2, 136.6 (C-20); 138.9, 139.0 (C-5); 139.9, 140.2 (C-12); 154.1, 154.6 (C-23); 171.0, 171.5, 171.8, 172.1 (C-7/9).

[4] The substance forms two rotamers in 0.6/0.4 ratio.

IR (ATR): $\tilde{\nu}$ [cm^{-1}] = 3084 (w); 3061 (w); 3028 (w); 2969 (w); 2934 (w); 2877 (w); 2843 (w); 1742 (m); 1698 (s); 1652 (s); 1605 (w); 1587 (w); 1546 (w); 1497 (w); 1478 (w); 1453 (m); 1433 (m); 1392 (s); 1365 (s); 1297 (m); 1257 (m); 1237 (m); 1214 (m); 1167 (s); 1117 (s); 1081 (m); 1063 (m); 1029 (w); 1024 (w); 990 (m); 967 (m); 913 (m); 864 (w); 846 (w); 833 (w); 790 (w); 771 (m); 753 (m); 734 (w); 700 (s); 661 (w); 645 (w); 620 (w).

HR/MS (ESI): calculated for [M+Na]$^+$ 521.2086; found: 521.2988.

$[\boldsymbol{\alpha}]_{\boldsymbol{\lambda}}^{\mathbf{20}}$ (CHCl$_3$, c = 0.505 g/100 ml):): $[\alpha]_{365}^{20}$= -325.0°; $[\alpha]_{436}^{20}$= -190.2°; $[\alpha]_{546}^{20}$ = -105.1°; $[\alpha]_{579}^{20}$= -91.5°; $[\alpha]_{589}^{20}$= -87.9°.

Synthesis of (3aS,6R,8aR)-tert-Butyl 6-ethyl-7-((S)-1-methoxy-1-oxo-3-phenylpropane-2-yl)-8-oxo-3,3a,6,7,8,8a-hexahydropyrrolo[2,3-c]azepin-1(2H)-carboxylate ((*S,R,R,S*)-7b)

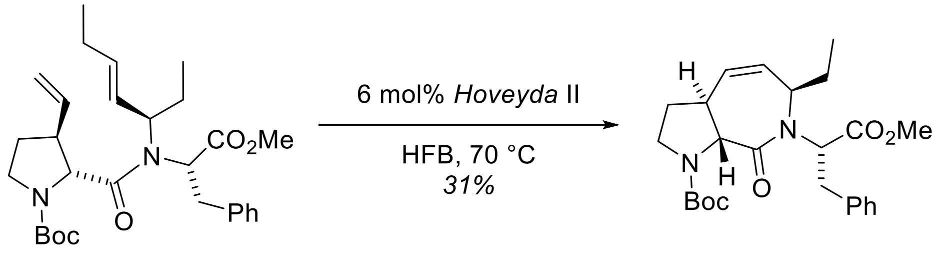

(*S,R,R,S*)-**6b** (*S,R,R,S*)-**7b**

Under inert conditions a solution of 125 mg (0.251 mmol 1.0 eq.) of the dipeptide (*S,R,R,S*)-**6b** and 6.3 mg (10.0 µmol, 4 mol%) of the *Hoveyda-Grubbs II* catalyst (**9**) in 2.5 ml C_6F_6 (0.1 M) was warmed to 70 °C and stirred for 3.5 h before another 3.1 mg (5.0 µmol, 2 mol%) of *Hoveyda-Grubbs II* catalyst (**9**) were added. (Note: The mixture turns dark upon heating). After 24 h the solution was allowed to cool down before the solvent was removed under reduced pressure. The resulting dark green oil was purified by flash column chromatography (SiO_2, cHex/EtOAc = 1/1). The resulting oily product was dissolved in little CH_2Cl_2 and stirred over *QuadraSil AP* for 15 min to remove residual ruthenium. After filtration the solvent was removed under reduced pressure and a second column chromatography (SiO_2, CH_2Cl_2/EtOAc = 4/1) was performed due to still present impurities (Note: The obtained substance was a brown solid).The product was dried in high vacuum to afford 34 mg (0.077, 31%) of the desired bicyclic product (*S,R,R,S*)-**7b**.

$C_{25}H_{34}N_2O_5$

M: 442.55 g/mol.

TLC: R_f = 0.27 (Silica, cHex/EtOAc = 1/1), $KMnO_4$-reagent.

Mp: 173 - 177 °C.

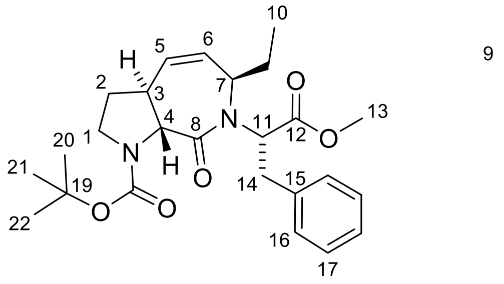

^{1}H NMR (500 MHz, $CDCl_3$, rotameric mixture[5]): δ [ppm] = 0.92 (t, ^3J = 7.5 Hz, 0.6H, H-10_{rot2}); 0.98 (t, ^3J = 7.5 Hz, 2.4H, H-10_{rot1}); 1.42 – 1.55 (m, 10H, H-2/21/22/23); 1.67 – 1.76 (m, 1H, H-9); 1.86 – 1.95 (m, 2H, H-2'/9'); 2.03 – 2.10 (m, 1H, H-3); 2.88 (dd, ^2J = 14.6 Hz, ^3J = 9.3 Hz, 0.8H, H-14_{rot1}); 2.97 (dd, ^2J = 14.2 Hz, ^3J = 9.4 Hz, 0.2H, H-14_{rot2}); 3.20 – 3.23 (m, 2H, H-1/14'); 3.59 – 3.69 (m, 4H, H-1'/13); 3.81 – 3.86 (m, 1H, H-7); 4.28 (d, ^3J = 10.8 Hz, 0.8H, H-4_{rot1}); 4.32 (d, ^3J = 10.9 Hz, 0.2H, H-4_{rot2}); 5.48 – 5.51 (m, 0.2H, H-11_{rot2}); 5.67 – 5.75 (m, 2.8H, H-5/6/11_{rot1}); 7.14 – 7.18 (m, 1H, H-18); 7.20 – 7.24 (m, 4H, H-16/17).

^{13}C NMR (150 MHz, $CDCl_3$, rotameric mixture[6]): δ [ppm] = 12.0, 12.1 (C-10); 28.2, 28.5 (C-21/22/23); 30.8, 30.8, 31.2, 31.2 (C-2/9); 35.8, 36.3 (C-14); 41.1, 41.5 (C-3); 46.0, 46.9 (C-1); 51.9, 52.1 (C-13); 57.4, 57.8, 59.1, 59.3 (C-7/11); 63.2, 63.4 (C-4); 79.4, 79.6 (C-20); 126.5, 126.6 (C-18); 128.1 (C-17); 128.6, 128.9 (C-6); 129.5, 129.7 (C-16); 129.8, 130.3 (C-5); 136.5, 136.9 (C-15); 154.1, 154.5 (C-19); 171.6 (C-8/12).

IR (ATR): $\tilde{\nu}$ [cm^{-1}] = 3088 (w); 3058 (w); 3027 (w); 2997 (w); 2970 (w); 2954 (w); 2928 (w); 2895 (w); 2865 (w); 1735 (m); 1694 (s); 1678 (s); 1654 (m); 1607 (w); 1498 (w); 1480 (w); 1452 (m); 1407 (s); 1392 (s); 1380 (m); 1364 (m); 1345 (m); 1317 (w); 1298 (w); 1278 (w); 1268 (w); 1252 (m); 1243 (m); 1230 (m); 1216 (m); 1179 (s); 1159 (s); 1117 (s); 1098 (w); 1084 (m); 1064 (w); 1044 (w); 1027 (w); 968 (m); 953 (w); 928 (m); 903 (w); 868 (m); 836 (w); 820 (w); 806 (w); 793 (w); 778 (m); 744 (m); 696 (s); 619 (w).

GC/MS (EI, 70 eV): m/z (%) = 342 (22); 313 (10); 285 (30); 274 (9); 251 (6); 220 (100); 207 (7); 179 (17); 160 (10); 151 (5); 136 (40); 122 (30); 104 (25); 91 ([C7H7]+, 47); 77 (22); 69 (16); 51 (11); 41 (15).

HR/MS (ESI): calculated for $[M+H]^+$: 443.2450; found: 443.2544;
calculated for $[M+Na]^+$ 465.2360; found: 465.2361.

$[\alpha]_\lambda^{20}$ ($CHCl_3$, c = 0.500 g/100 ml): $[\alpha]_{365}^{20}$= -226.9°; $[\alpha]_{436}^{20}$= -110.3°; $[\alpha]_{546}^{20}$ = -52.0°; $[\alpha]_{579}^{20}$= -43.9°; $[\alpha]_{589}^{20}$= -42.5°.

Synthesis of (2*R*,3*S*)-*tert*-Butyl 2-((*S*,*E*)-hept-4-en-3-yl((*S*)-1-methoxy-1-oxo-3-phenylpropane-2-yl)carbamoyl)-3-vinylpyrrolidine-1-carboxylate (*S*,*R*,*S*,*S*)-6b)

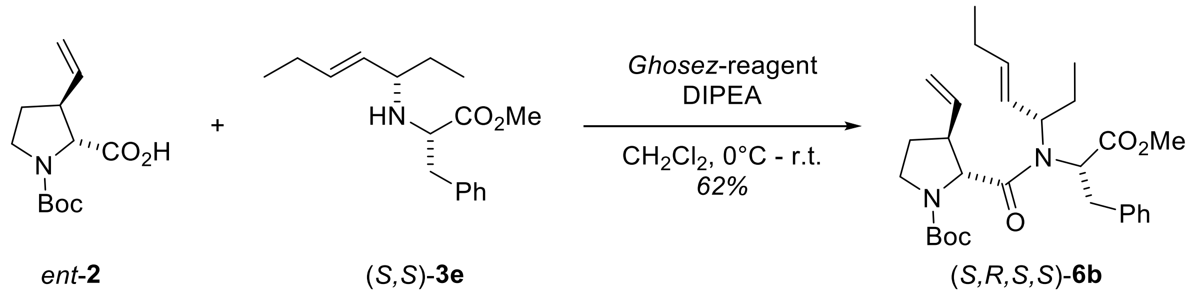

ent-**2** (*S*,*S*)-**3e** (*S*,*R*,*S*,*S*)-**6b**

The reaction was performed according to the **general procedure 2** using 119 mg (0.495 mmol) (*S*,*R*)-*Zaminer's acid* (*ent*-**2**) and stopped after 18.5 h.

---

[5] The substance forms two rotamers in 0.8:0.2 ratio.

The crude product was purified by flash column chromatography (Silica, cHex/EtOAc = 4/1) to yield 152 mg (0.305 mmol, 62%) of the desired dipeptide (*S,R,S,S*)-**6b** as a colourless viscose oil.

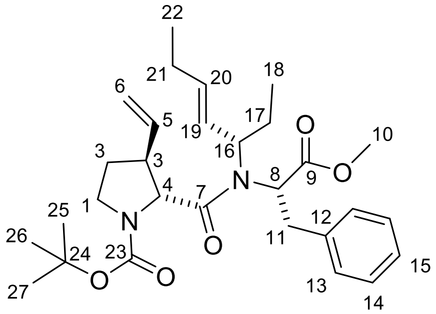

$C_{29}H_{42}N_2O_5$

M: 489.65 g/mol.

TLC: R_f = 0.18 (Silica, cHex/EtOAc = 4/1), $KMnO_4$-reagent.

^{1}H NMR (500 MHz, $CDCl_3$, rotameric mixture[6]): δ [ppm] = 0.72 (d, ^3J = 7.3Hz, 1.5H, H-18$_{rot1}$); 0.89 (d, ^3J = 7.3 Hz, 1.5H, H-18$_{rot2}$); 0.97 (Ψ dt, J = 1.1 Hz, J = 7.4 Hz, 3H, H-22); 1.03 – 1.17 (m, 1H, H-17); 1.38 – 1.56 (m, 10H, H-17'/25/26/27); 1.70 – 1.76 (m, 1H, H-2); 1.97 – 2.24 (m, 3H, H-2'/21); 2.87 – 2.95 (m, 1.5H, H-3/11$_{rot1}$); 3.26 (dd, ^3J = 14.2 Hz, ^3J = 7.2 Hz, 0.5H, H-11$_{rot2}$); 3.42 – 3.50 (m, 1H, H-1); 3.57 – 3.68 (m, 4H, H-1'rot1/10/11'$_{rot2}$); 3.69 – 3.74 (m, 0.5H, H-1'$_{rot2}$); 3.80 – 3.83 (m, 0.5H, H-8$_{rot1}$); 3.86 – 3.92 (m, 1H,H-8rot2/11'$_{rot1}$); 3.99 – 4.07 (m, 1H, H-16); 4.41 (s, 0.5H, H-4$_{rot1}$); 4.49 (d, ^4J = 1.8 Hz, 0.5H, H-4$_{rot2}$); 5.07 – 5.16 (m, 2H, H-6); 5.37 (dd, ^3J = 15.7 Hz, ^3J = 6.4 Hz, 0.5H, H-19$_{rot1}$); 5.50 (dd, ^3J = 15.7 Hz, ^3J = 6.0 Hz, 0.5H, H-19$_{rot2}$); 5.60 (dt, ^3J = 15.7 Hz, ^3J = 6.3 Hz, 0.5H, H-20$_{rot1}$); 5.69 (dt, ^3J = 15.7 Hz, ^3J = 6.2 Hz, 0.5H, H-20$_{rot2}$); 5.89 (Ψ tdd, J = 17.9 Hz, J = 10.2 Hz, J = 8.1 Hz, 1H, H-5); 7.16 –7.25 (m, 1H, H-15); 7.26 – 7.36 (m, 4H, H-13/14).

^{13}C NMR (125 MHz, $CDCl_3$, rotameric mixture[7]): δ [ppm] = 11.6, 11.7 (C-18); 13.6, 13.6 (C-22); 25.6, 25.7 (C-21); 25.8, 26.5 (C-17); 28.5 (C-2$_{rot1}$); 28.7, 28.8 (C-25/26/27); 30.1 (C-2$_{rot2}$); 36.5, 38.0 (C-11); 45.6 (C-1$_{rot1}$); 45.7 (C-3$_{rot1}$); 45.8 (C-1$_{rot2}$); 46.5 (C-3$_{rot2}$); 52.2, 52.2 (C-10); 59.2, 59.5 (C-8); 60.4 (C-16); 62.4, 62.8 (C-4); 79.5, 80.0 (C-24); 115.0, 115.1 (C-6); 125.9, 126.0 (C-19); 126.4, 126.6 (C-15); 128.4, 128.5 (C-14); 129.6, 130.1 (C-13); 136.4, 136.7 (C-20); 139.0, 139.0 (C-5); 139.8, 140.1 (C-12); 154.3, 154.6 (C-23); 171.4, 171.4 (C-7$_{rot1}$/9$_{rot1}$); 171.6 (C-9$_{rot2}$); 172.0 (C-7$_{rot2}$).

IR (ATR): $\tilde{\nu}$ [cm^{-1}] = 3085 (w); 3063 (w); 3027 (w); 2967 (w); 2933 (w); 2877 (w); 1742 (m); 1696 (s); 1653 (s); 1606 (w); 1583 (w); 1545 (w); 1496 (w); 1478 (w); 1455 (m); 1431 (m); 1393 (s); 1365 (s); 1305 (m); 1257 (m); 1239 (m); 1215 (m); 1167 (s); 1150 (s); 1117 (m); 1082 (m); 1065 (m); 1030 (w); 977 (m); 914 (m); 864 (w); 846 (w); 829 (w); 790 (w); 770 (m); 752 (m); 701 (s); 661 (w); 643 (w); 623 (w).

HR/MS (ESI): calculated for $[M+H]^+$ 499.3166; found: 499.3170, $[M+Na]^+$ 521.2086; found: 521.2985.

$[\boldsymbol{\alpha}]_{\lambda}^{\mathbf{20}}$ ($CHCl_3$, c = 0.500 g/100 ml):): $[\alpha]_{365}^{20}$= -427.4°; $[\alpha]_{436}^{20}$= -249.8°; $[\alpha]_{546}^{20}$ = -138.3°; $[\alpha]_{579}^{20}$= -120.1°; $[\alpha]_{589}^{20}$= -155.5°.

[6] The substance forms two rotamers in 1/1 ratio.

Synthesis of (3aS,6S,8aR)-tert-Butyl 6-ethyl-7-((S)-1-methoxy-1-oxo-3-phenylpropane-2-yl)-8-oxo-3,3a,6,7,8,8a-hexahydropyrrolo[2,3-c]azepin-1(2H)-carboxylate ((*S,R,S,S*)-7b)

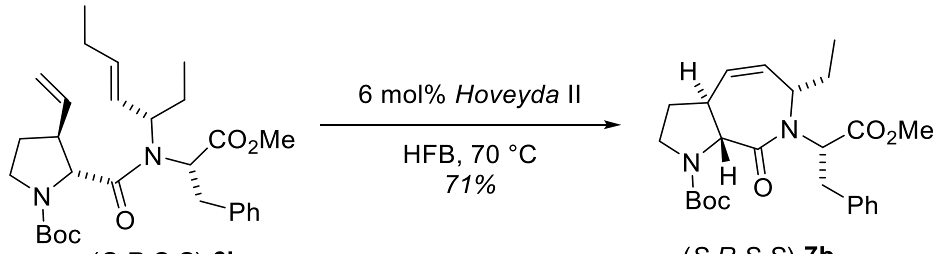

Under inert conditions a solution of 142 mg (0.285 mmol, 1.0 eq.) of dipeptide (*S,R,S,S*)-**6b** and 7.1 mg (11.4 µmol, 4 mol%) of the *Hoveyda-Grubbs II* catalyst (**9**) in 2.9 ml C_6F_6 (0.1 M) was warmed to 70 °C and stirred for 3.5 h before another 3.6 mg (5.7 µmol, 2 mol%) of *Hoveyda-Grubbs II* catalyst (**9**) were added. (Note: the mixture turns dark upon heating). After 24 h the solution was allowed to cool down before the solvent was removed under reduced pressure. The resulting dark green oil was purified by flash column chromatography (Silica, cHex/ EtOAc = 1/1). The resulting oily product was dissolved in little CH_2Cl_2 and stirred over *QuadraSil AP* for 15 min to remove residual ruthenium. After filtration the solvent was removed under reduced pressure and the product was dried in high vacuum to afford 90 mg (0.203 mmol, 71%) of the bicyclic product (*S,R,S,S*)-**7b** as a colorless viscous oil.

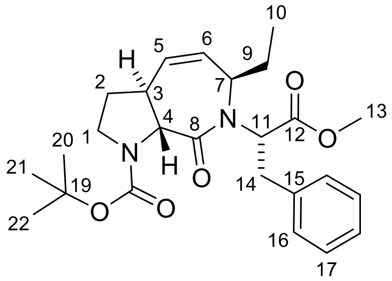

$C_{25}H_{34}N_2O_5$

M: 442.55 g/mol.

TLC: R_f = 0.18 (Silica, cHex/EtOAc = 1/1), $KMnO_4$-reagent.

Mp: 145 – 149 °C

^{1}H NMR (500 MHz, $CDCl_3$, rotameric mixture[7]): δ [ppm] = 0.68 – 0.71 (m, 1.5H, H-10_{rot1}); 0.75 – 0.86 (m, 2H, H-10_{rot2}/9_{rot1}); 0.88 – 0.96 (m, 0.5H, H-9_{rot2}); 1.10 – 1.31 (m, 1H, H-9'); 1.50 (s, 9H, H-21/22/23); 1.60 – 1.69 (m, 1H, H-2); 2.03 (Ψ dt, *J* = 11.4 Hz, *J* = 5.6 Hz, 1H, H-2'); 2.82 – 2.88 (m, 1H, H-3); 3.23 (dd, ^2J = 13.6 Hz, ^3J = 7.0 Hz, 0.5H, H-14_{rot1}); 3.40 – 3.54 (m, 2H, H-1/14_{rot2}/$14'_{rot2}$); 3.66 – 3.71 (m, 4H, H-$1'_{rot1}$/$14'_{rot1}$); 3.76 – 3.79 (m, 0.5H, H-$1'_{rot2}$); 3.97 – 4.21 (m, br, 1H, H-11); 4.33 – 4.42 (m, 1H, H-7); 4.82 (d, ^3J = 11.8 Hz, 1H, H-4); 5.45 (Ψ d, *J* = 10.6 Hz, 1H, H-6); 5.82 (Ψ d, *J* = 11.1 Hz, 1H, H-5); 7.20 – 7.33 (m, 5H, H-16/17/18).

^{13}C NMR (125 MHz, $CDCl_3$, rotameric mixture[8]): δ [ppm] = 11.7, 12.0 (C-10); 26.2, 26.3 (C-9); 28.7 (C-21/22/23); 30.0, 30.7 (C-2); 36.4, 37.4 (C-14); 42.5, 43.6 (C-3); 47.2, 47.5 (C-1); 52.4, 52.4 (C-13); 56.2, 56.4 (C-7); 59.2 (C-11); 60.3, 60.5 (C-4); 79.7, 80.2 (C-20); 126.5, 126.7 (C-18); 128.5 (C-16 o. C-17); 129.7, 130.0, 130.1, 130.4

[7] The substance forms two rotamers in 1:1 ratio.

(C-6/C-16 o. C-17); 131.4, 131.7 (C-5); 139.4, 139.6 (C-15); 154.5, 155.0 (C-19); 171.5, 171.6 (C-12); 172.0, 172.2 (C-8).

IR (ATR): $\tilde{\nu}$ [cm^{-1}] = 3102 (w); 3087 (w); 3062 (w); 3026 (w); 2972 (w); 2952 (w); 2934 (w); 2882 (w); 1740 (m); 1695 (s); 1674 (s); 1605 (w); 1585 (w); 1544 (w); 1496 (w); 1479 (w); 1454 (m); 1432 (m); 1405 (s); 1392 (s); 1365 (s); 1341 (m); 1323 (m); 1292 (m); 1257 (m); 1248 (m); 1229 (m); 1217 (m); 1159 (s); 1127 (s); 1104 (m); 1081 (m); 1064 (m); 1026 (m); 976 (w); 923 (m); 865 (w); 851 (w); 829 (w); 765 (m); 751 (m); 729 (s); 704 (s); 674 (w); 661 (m); 645 (m).

GC/MS (EI, 70 eV): m/z (%) = 342 (17); 313 (13); 285 (22); 274 (11); 251 (5); 220 (100); 207 (7); 179 (28); 160 (11); 151 (13); 136 (59); 122 (38); 108 (30); 91 ([C_7H_7]$^+$, 50); 77 (26); 69 (28); 51 (13); 41 (20).

HR/MS (ESI): calculated for [M+H]$^+$: 443.2450; found: 443.2543; for [M+Na]$^+$ 465.2360; found: 465.2360.

$[\alpha]_{\lambda}^{20}$ ($CHCl_3$, c = 0.520 g/100 ml): $[\alpha]_{365}^{20}$= +126.1°; $[\alpha]_{436}^{20}$= +92.6°; $[\alpha]_{546}^{20}$ = -59.7°; $[\alpha]_{579}^{20}$= -53.5°; $[\alpha]_{589}^{20}$= -50.7°.

Synthesis of (2*R*,3*S*)-*tert*-Butyl 2-((*S*,*E*)-hept-4-en-3-yl((*R*)-1-methoxy-1-oxo-3-phenylpropane-2-yl)carbamoyl)-3-vinylpyrrolidine-1-carboxylate ((*S,R,S,R*)-6b)

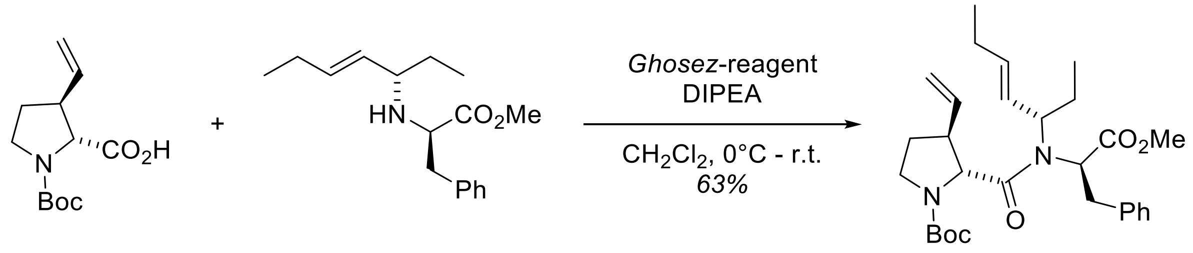

ent-**2** (*S,R*)-**3e** (*S,R,S,R*)-**6b**

The reaction was performed according to the **general procedure 2** using 80 mg (0.330 mmol) (*S,R*)-*Zaminer's acid* (*ent*-**2**) and stopped after 17 h.
The crude product was purified by flash column chromatography (Silica, cHex/EtOAc = 4/1) to yield 104 mg (0.209 mmol, 63%) of the desired dipeptide (*S,R,S,R*)-**6b** as a pale-yellow viscose oil.

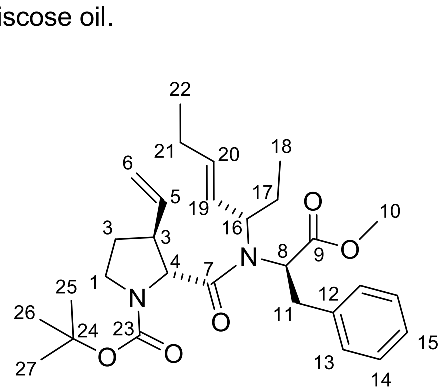

$C_{29}H_{42}N_2O_5$

M: 489.65 g/mol.

TLC: R_f = 0.17 (Silica, cHex/EtOAc = 4/1), $KMnO_4$-reagent.

^{1}H NMR (500 MHz, $CDCl_3$, rotameric mixture[8]): δ [ppm] = 0.90 – 0.96 (m, 6H, H-18/22); 1.45, 1.46 (2 × s, 9H, H-25/26/27); 1.61 – 1.72 (m, 1H, H-17); 1.75 – 1.80 (m, 1H, H-2); 1.82 – 2.10 (m, 3H, H-17'/21); 2.16 – 2.28 (m, 1H,

[8] The substance forms two rotamers in 0.6/0.4 ratio.

H-2'); 2.74 – 2.78 (m, 1H, H-3); 2.99 (dd, ^2J = 13.9 Hz, ^3J = 6.1 Hz, 0.4H, H-11_{rot2}); 3.06 (dd, 2J = 14.1 Hz, ^3J = 6.8 Hz, 0.6H, H-11_{rot1}); 3.44 – 3.53 (m, 1H, H-1); 3.59 – 3.75 (m, 5H, H-1'/10/11'); 3.83 (Ψ t, J = 6.6 Hz, 0.4H, H-8_{rot2}); 3.86 – 3.94 (m, 1.2H, H-8_{rot1}/16_{rot1}); 3.99 – 4.03 (m, 0.4H, H-16_{rot2}); 4.43 (s, 0.6H, H-4_{rot1}); 4.53 (d, ^4J = 2.0 Hz, 0.4H, H-4_{rot2}); 4.73 – 4.82 (m, 1H, H-19); 5.06 – 5.14 (m, 2H, H-6); 5.49 – 5.55 (m, 1H, H-20); 5.85 – 5.93 (m, 1H, H-5); 7.16 –7.22 (m, 3H, H-13/15); 7.24 – 7.30 (m, 2H, H-14).

^{13}C NMR (125 MHz, $CDCl_3$, rotameric mixture[9]): δ [ppm] = 11.6, 11.7 (C-18); 13.5 (C-22); 25.8, 25.8, 25.8, 26.2 (C-17/21); 28.4, 28.6 (C-25/26/27); 28.7, 30.2 (C-2); 36.3, 36.7 (C-11); 45.3, 45.7 (C-1); 46.6, 47.3 (C-3); 52.1, 52.3 (C-10); 58.4, 58.5 (C-8); 61.0, 61.1 (C-16); 62.2, 62.8 (C-4); 79.3, 80.1 (C-24); 115.0, 115.2 (C-6); 125.8, 126.2 (C-19); 126.5, 126.6 (C-15); 128.3, 128.3 (C-14); 129.8, 129.9 (C-13); 136.7, 136.9 (C-20); 139.0, 139.1 (C-5); 139.3, 139.6 (C-12); 154.3, 154.4 (C-23); 171.3, 171.3, 171.8, 171.8 (C-7/9).

IR (ATR): $\tilde{\nu}$ [cm^{-1}] = 3087 (w); 3064 (w); 3028 (w); 2966 (w); 2932 (w); 2876 (w); 2851 (w); 1741 (m); 1698 (s); 1654 (s); 1605 (w); 1587 (w); 1544 (w); 1496 (w); 1480 (w); 1453 (m); 1431 (m); 1392 (s); 1364 (s); 1331 (m); 1304 (m); 1254 (m); 1239 (m); 1217 (m); 1166 (s); 1116 (s); 1080 (w); 1064 (m); 1030 (w); 991 (m); 968 (m); 914 (m); 864 (w); 844 (w); 828 (w); 821 (w); 790 (w); 770 (m); 752 (m); 701 (s); 648 (w).

HR/MS (ESI): calculated for $[M+H]^+$ 499.3166; found: 499.3168, $[M+Na]^+$ 521.2086; found: 521.2988.

$[\boldsymbol{\alpha}]_{\boldsymbol{\lambda}}^{\mathbf{20}}$ ($CHCl_3$, c = 0.510 g/100 ml):): $[\alpha]_{436}^{20}$= +41.5°; $[\alpha]_{546}^{20}$ = +25.8°; $[\alpha]_{579}^{20}$= +22.7°; $[\alpha]_{589}^{20}$= +21.2°.

Synthesis of (3aS,6S,8aR)-tert-Butyl 6-ethyl-7-((R)-1-methoxy-1-oxo-3-phenylpropane-2-yl)-8-oxo-3,3a,6,7,8,8a-hexahydropyrrolo[2,3-c]azepin-1(2H)-carboxylate ((*S,R,S,R*)-7b)

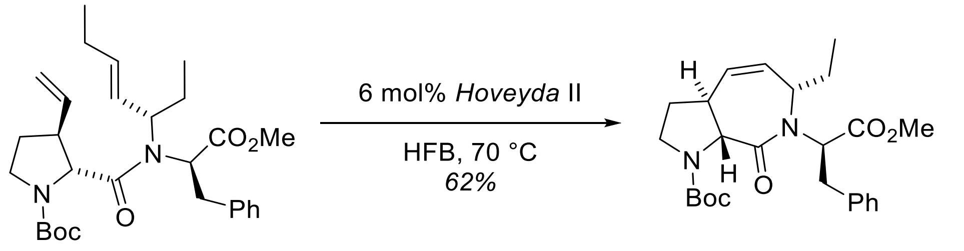

(*S,R,S,R*)-**6b** (*S,R,S,R*)-**7b**

Under inert conditions a solution of 140 mg (0.281 mmol, 1.0 eq.) of dipeptide (*S,R,S,R*)-**6b** and 7.0 mg (11.2 µmol, 4 mol%) of the *Hoveyda-Grubbs II* catalyst (**9**) in 2.8 ml C_6F_6 (0.1 M) was warmed to 70 °C and stirred for 3.5 h before another 3.5 mg (5.7 µmol, 2 mol%) of *Hoveyda-Grubbs II* catalyst (**9**) were added. (Note: the mixture turns dark upon heating). After 24 h the solution was allowed to cool down before the solvent was removed under reduced pressure. The resulting dark green oil was purified by flash column chromatography (Silica, cHex/ EtOAc = 1/1). The resulting oily product was dissolved in little CH_2Cl_2 and stirred over *QuadraSil AP* for 15 min to remove residual ruthenium. After filtration the solvent was removed under reduced pressure and the product was dried in high vacuum to afford 77 mg (0.174 mmol, 62%) of the bicyclic product (*S,R,S,R*)-**7b** as a colorless viscous oil.

$C_{25}H_{34}N_2O_5$

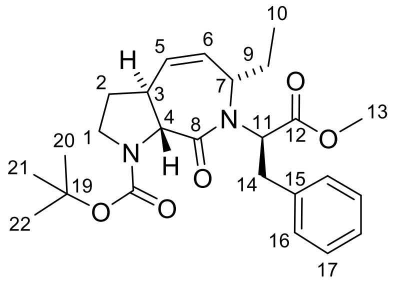

M: 442.55 g/mol.

TLC: R_f = 0.24 (Silica, cHex/EtOAc = 1/1), $KMnO_4$-reagent.

Mp: 161 - 165 °C.

^{1}H NMR (500 MHz, $CDCl_3$, rotameric mixture[9]): δ [ppm] = 0.92 – 1.02 (m, 3H, H-10); 1.36 – 1.48 (m, 10H, H-9/21/22/23); 1.54 – 1.62 (m, 1H, H-2); 1.75 – 1.81 (m, 1H, H-9'); 1.98 (Ψ dt, *J* = 11.3 Hz, *J* = 5.5 Hz, 1H, H-2'); 2.69 – 2.75 (m, 1H, H-3); 3.12 – 3.22 (m, 1H, H-14); 3.38 – 3.43 (Ψ td, *J* = 11.3 Hz, *J* = 5.4 Hz, 1H, H-14); 3.54 (dd, ^2J = 14.1 Hz, ^3J = 5.2 Hz, 1H, H-14'); 3.63 – 3.76 (m, 4H, H-1'/13); 4.13 – 4.16 (m, 1H, H-11); 4.48 – 4.56 (m, br, 0.75H, H-7_{rot1}); 4.56 – 4.67 (m, br, 0.25H, H7_{rot2}); 4.78 (d, ^3J = 11.9 Hz, 1H, H-4); 4.94 – 5.03 (m, 1H, H-6); 5.41 (Ψ d, *J* = 10.9 Hz, 1H, H-5); 7.14 – 7.18 (m, 3H, H-17/18); 7.21 – 7.24 (m, 2H, H-16).

^{13}C NMR (125 MHz, $CDCl_3$, rotameric mixture[10]): δ [ppm] = 11.7 (C-10); 26.7, 26.9 (C-9); 28.3, 28.5 (C-21/22/23); 30.2, 30.7 (C-2); 35.1, 35.3 (C-14); 43.3, 44.0 (C-3); 46.9, 47.5 (C-1); 52.5 (C-13); 55.6, 56.0 (C-7); 58.0, 58.2 (C-11); 60.1, 60.4 (C-4); 79.9 (C-20); 126.5 (C-18); 128.4 (C-17); 129.6 (C-16); 130.3, 130.6, 131.0 (C-5/6); 139.1 (C-15); 154.5 (C-19); 171.5 (C-12); 172.0 (C-8).

IR (ATR): $\tilde{\nu}$ [cm^{-1}] = 3087 (w); 3060 (w); 3026 (w); 3005 (w); 2966 (w); 2932 (w); 2869 (w); 1752 (m); 1734 (m); 1693 (s); 1672 (s); 1645 (w); 1604 (w); 1585 (w); 1541 (w); 1496 (w); 1478 (w); 1453 (m); 1447 (m); 1426 (m); 1404 (s); 1388 (s); 1365 (m); 1333 (w); 1318 (m); 1296 (w); 1279 (w); 1252 (m); 1233 (m); 1218 (s); 1178 (m); 1162 (s); 1125 (m); 1116 (m); 1103 (m); 1081 (m); 1060 (m); 1041 (m); 1033 (m); 1000 (w); 987 (w); 977 (w); 966 (w); 939 (w); 919 (m); 863 (w); 852 (m); 824 (w); 782 (m); 764 (m); 745 (s); 727 (m); 696 (s); 676 (w); 652 (m); 623 (w); 610 (m).

GC/MS (EI, 70 eV): m/z (%) = 342 (15); 313 (14); 285 (21); 274 (8); 251 (4); 220 (100); 207 (10); 179 (23); 160 (11); 151 (12); 136 (55); 122 (35); 108 (26); 91 ($[C_7H_7]^+$, 40); 77 (24); 69 (25); 51 (10); 41 (20).

HR/MS (ESI): calculated for $[M+H]^+$: 443.2450; found: 443.2544; for $[M+Na]^+$ 465.2360; found: 465.2360.

$[\alpha]_{\lambda}^{20}$ ($CHCl_3$, c = 0.520 g/100 ml): $[\alpha]_{365}^{20}$ = +780.0°; $[\alpha]_{436}^{20}$ = +479.7°; $[\alpha]_{546}^{20}$ = -275.6°; $[\alpha]_{579}^{20}$ = -239.6°; $[\alpha]_{589}^{20}$= -229.6°.

Synthesis of (2*R*,3*S*)-*tert*-Butyl 2-((*R*,*E*)-hept-4-en-3-yl((*R*)-1-methoxy-1-oxo-3-phenylpropane-2-yl)-carbamoyl)-3-vinylpyrrolidine-1-carboxylate ((S,R,R,R)-6b)

[9] The substance forms two rotamers in 0.75:0.25 ratio.

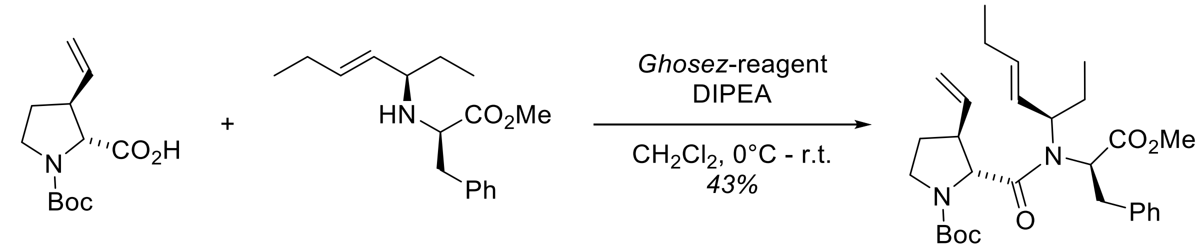

The reaction was performed according to the **general procedure 2** using 119 mg (0.495 mmol) (*S,R*)-*Zaminer's acid* (*ent*-**2**) and stopped after 18.5 h.

The crude product was purified by flash column chromatography (Silica, cHex/EtOAc = 4/1) to yield 106 mg (0.213 mmol, 43%) of the desired dipeptide (*S,R,R,R*)-**6b** as a white solid. The product still contained minor amounts of impurities which could not be separated by repetitive column chromatographic purification

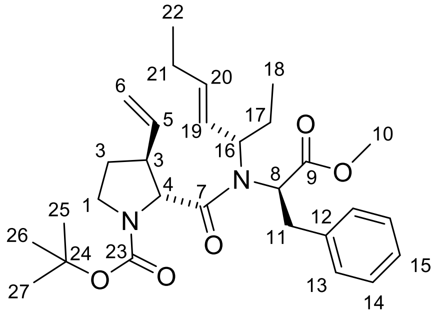

$C_{29}H_{42}N_2O_5$

M: 489.65 g/mol.

TLC: R_f = 0.19 (Silica, cHex/EtOAc = 4/1), $KMnO_4$-reagent.

Mp.: 101 – 103 °C.

^{1}H NMR (500 MHz, $CDCl_3$, rotameric mixture[10]): δ [ppm] = 0.70 – 0.73 (m, 3H, H-18); 0.83 – 1.03 (m, 4H, H-17/22); 1.19 – 1.34 (m, 1H, H-17'); 1.45, 1.45 (2 × s, 9H, H-25/26/27); 1.73 – 1.79 (m, 1H, H-2); 1.99 – 2.07 (m, 2H, H-21); 2.21 – 2.28 (m, 0.6H, H-2'$_{rot1}$); 2.31 – 2.38 (m, 0.4H, H-2'$_{rot2}$); 2.69 – 2.72 (m, 0.6H, H-3$_{rot1}$); 2.78 – 2.86 (m, 0.4H, H-3$_{rot2}$); 3.04 (dd, ^3J = 14.0 Hz, ^3J = 6.7 Hz, 0.4H, H-11$_{rot2}$); 3.14 (dd, ^3J = 14.1 Hz, ^3J = 7.2 Hz, 0.6H, H-11$_{rot1}$); 3.46 – 3.55 (m, 1H, H-1); 3.59 – 3.73 (m, 5H, H-1'/10/11'); 3.79 (Ψ t, *J* = 6.7 Hz, 0.4H, H-8$_{rot2}$); 3.83 (Ψ t, ^3J = 6.9 Hz, 0.6H, H-8$_{rot1}$); 3.89 – 3.93 (m, 0.6H, H-16$_{rot1}$); 4.23 – 4.27 (m, 0.4H, H-16$_{rot2}$); 4.47 (s, 0.6H, H-4$_{rot1}$); 4.54 (d, ^4J = 2.4 Hz, 0.4H, H-4$_{rot2}$); 5.03 – 5.10 (m, 2H, H-6); 5.41 – 5.66 (m, 2H, H-19/20); 5.79 – 5.91 (m, 1H, H-5); 7.19 –7.23 (m, 3H, H-13/15); 7.26 – 7.30 (m, 2H, H-14).

^{13}C NMR (125 MHz, $CDCl_3$, rotameric mixture[11]): δ [ppm] = 11.5, 11.6 (C-18); 13.3, 13.5 (C-22); 25.4, 25.6, 25.6 (C-17/21); 28.4, 28.6 (C-25/26/27); 29.1, 30.6 (C-2); 36.3, 36.6 (C-11); 45.2, 45.9 (C-1); 47.0, 47.8 (C-3); 51.9, 52.3 (C-10); 58.9, 59.0 (C-8); 60.4, 61.0 (C-16); 61.7, 62.9 (C-4); 79.3, 79.9 (C-24); 115.1, 115.3 (C-6); 126.1, 126.4 (C-19); 126.5, 126.7 (C-15); 128.4 (C-14); 129.8 (C-13); 135.3, 135.9 (C-20); 139.0, 139.0 (C-5); 139.5, 139.9 (C-12); 154.3, 154.3 (C-23); 171.0, 171.6, 171.8, 172.5 (C-7/9).

IR (ATR): $\tilde{\nu}$ [cm^{-1}] = 3104 (w); 3084 (w); 3064 (w); 3031 (w); 2962 (w); 2952 (w); 2934 (w); 2874 (w); 2846 (w); 1746 (m); 1731 (m); 1684 (s); 1653 (s); 1606 (w); 1541 (w); 1497 (w); 1482 (w); 1456 (m); 1445 (m); 1405 (s); 1363 (m); 1351 (m); 1331 (w); 1301 (m); 1278 (m); 1232 (m); 1215 (m); 1208 (m); 1194 (m); 1185 (m); 1167 (s); 1150

[10] The substance forms two rotamers in 0.6/0.4 ratio.

(m); 1125 (s); 1080 (w); 1062 (m); 1028 (m); 1013 (m); 997 (m); 988 (m); 969 (m); 914 (m); 865 (m); 829 (w); 816 (w); 797 (w); 767 (m); 756 (m); 705 (s); 644 (m).

HR/MS (ESI): calculated for $[M+Na]^+$ 521.2986; found: 521.2988.

$[\boldsymbol{\alpha}]_{\lambda}^{\mathbf{20}}$ ($CHCl_3$, c = 0.500 g/100 ml):): $[\alpha]_{365}^{20}$ = +172.3°; $[\alpha]_{436}^{20}$ = +106.7°; $[\alpha]_{546}^{20}$ = +61.8°; $[\alpha]_{579}^{20}$ = +53.8°; $[\alpha]_{589}^{20}$ = +51.2°.

Synthesis of (3aS,6R,8aR)-tert-Butyl 6-ethyl-7-((R)-1-methoxy-1-oxo-3-phenylpropane-2-yl)-8-oxo-3,3a,6,7,8,8a-hexahydropyrrolo[2,3-c]azepin-1(2H)-carboxylate ((*S,R,R,R*)-7b)

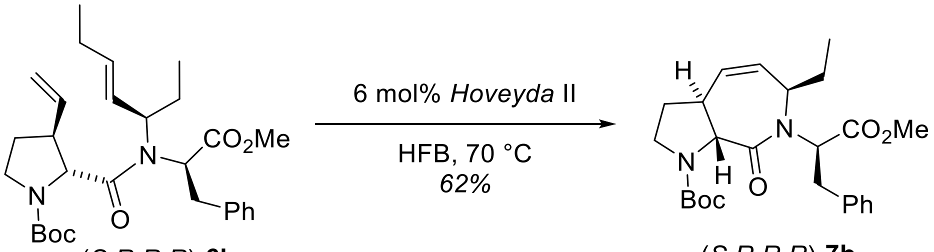

(*S,R,R,R*)-**6b** (*S,R,R,R*)-**7b**

Under inert conditions a solution of 120 mg (0.241 mmol, 1.0 eq.) of dipeptide (*S,R,R,R*)-**6b** and 6.0 mg (9.6 µmol, 4 mol%) of the *Hoveyda-Grubbs II* catalyst (**9**) in 2.4 ml C_6F_6 (0.1 M) was warmed to 70 °C and stirred for 3.5 h before another 3.0 mg (4.8 µmol, 2 mol%) of *Hoveyda-Grubbs II* catalyst (**9**) were added. (Note: the mixture turns dark upon heating). After 24 h the solution was allowed to cool down before the solvent was removed under reduced pressure. The resulting dark green oil was purified by flash column chromatography (Silica, cHex/ EtOAc = 1/1). The resulting oily product was dissolved in little CH_2Cl_2 and stirred over *QuadraSil AP* for 15 min to remove residual ruthenium. After filtration the solvent was removed under reduced pressure and the product was dried in high vacuum to afford 66 mg (0.149 mmol, 62%) of the bicyclic product (*S,R,R,R*)-7**b** as a colorless viscous oil.

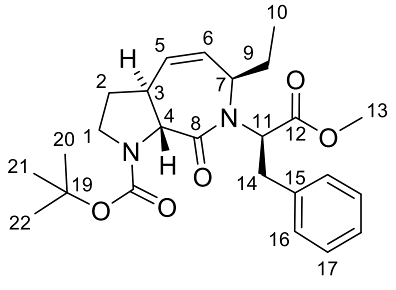

$C_{25}H_{34}N_2O_5$

M: 442.55 g/mol.

TLC: R_f = 0.24 (Silica, cHex/EtOAc = 1/1), $KMnO_4$-reagent.

Mp: 132 – 135 °C.

^{1}H NMR (500 MHz, $CDCl_3$, rotameric mixture[11]): δ [ppm] = 0.96 (t, ^3J = 7.2 Hz, 0.6H, H-10_{rot2}); 1.01 (t, ^3J = 7.5 Hz, 2.4H, H-10_{rot1}); 1.38, 1.47 (2 × s, 9H, H-21/22/23); 1.52 – 1.59 (m, 1H, H-2); 1.77 – 1.86 (m, 1H, H-9); 1.89 – 1.98 (m, 1H, H-9'); 2.03 (Ψ dt, ^3J = 11.4 Hz, J = 5.6 Hz, 1H, H-2'); 2.69 – 2.75 (m, 1H, H-3); 3.08 – 3.18 (m, 1H, H-14); 3.29 – 3.43 (m, 3H, H-1/7/14'); 3.65 – 3.78 (m, 4H, H-1'/13); 4.24 (d, ^3J = 10.6 Hz, 0.8H, H-4_{rot1}); 4.28 (d, ^3J = 10.7

[11] The substance forms two rotamers in 0.8:0.2 ratio.

Hz, 0.2H, H-4$_{rot2}$); 4.57 (dd, ^3J = 9.3 Hz, ^3J = 5.4 Hz, 1H, H11); 5.32 – 5.40 (m, 1H, H-6); 5.67 – 5.73 (m, 1H, H-5); 7.17 – 7.27 (m, 5H, H-16/17/18).

^{13}C NMR (125 MHz, $CDCl_3$, rotameric mixture[12]): δ [ppm] = 11.9, 11.9 (C-10); 28.3, 28.6 (C-21/22/23); 30.3, 30.4 (C-9); 31.1, 31.6 (C-2); 35.5, 35.6 (C-14); 41.3, 42.0 (C-3); 46.5, 47.1 (C-1); 52.1, 52.3 (C-13); 62.8, 63.3, 63.5 (C-4$_{rot2}$/7$_{rot2}$/11$_{rot2}$); 63.8, 63.8, 64.3 (C-4$_{rot1}$/7$_{rot1}$/11$_{rot1}$); 79.5, 79.8 (C-20); 126.6, 126.7 (C-18); 128.6, 128.7 (C-17); 128.9, 128.9 (C-6); 129.1, 129.2 (C-5); 129.3, 129.5 (C-16); 137.8, 138.0 (C-15); 154.3, 154.5 (C-19); 170.9, 171.0 (C-12); 171.3, 171.4 (C-8).

IR (ATR): $\tilde{\nu}$ [cm^{-1}] = 3088 (w); 3047 (w); 3028 (w); 3012 (w); 2973 (w); 2934 (w); 2906 (w); 2878 (w); 2857 (w); 1738 (s); 1698 (s); 1660 (s); 1654 (s); 1605 (w); 1497 (w); 1484 (m); 1458 (m); 1435 (m); 1394 (s); 1384 (s); 1367 (m); 1330 (m); 1309 (w); 1288 (m); 1273 (w); 1251 (m); 1239 (m); 1227 (m); 1218 (m); 1190 (m); 1163 (s); 1118 (s); 1094 (m); 1081 (m); 1042 (m); 1029 (w); 992 (w); 968 (w); 938 (m); 917 (m); 876 (w); 848 (w); 837 (w); 824 (w); 771 (w); 754 (m); 743 (m); 730 (m); 705 (m); 694 (m); 666 (w); 631 (w).

GC/MS (EI, 70 eV): m/z (%) = 342 (17); 313 (10); 285 (25); 274 (11); 251 (6); 220 (100); 207 (7); 179 (17); 160 (10); 151 (6); 136 (40); 122 (38); 104 (24); 91 ([C_7H_7]$^+$, 47); 77 (22); 69 (19); 51 (10); 41 (15).

HR/MS (ESI): calculated for [M+H]$^+$: 443.2450; found: 443.2543; for [M+Na]$^+$ 465.2360; found: 465.2361.

$[\alpha]_\lambda^{20}$ ($CHCl_3$, c = 0.515 g/100 ml): $[\alpha]_{365}^{20}$ = +459.8°; $[\alpha]_{436}^{20}$ = +284.7°; $[\alpha]_{546}^{20}$ = -165.1°; $[\alpha]_{579}^{20}$ = -144.5°; $[\alpha]_{589}^{20}$= -137.9°.

Synthesis of (2*S*,3*R*)-*tert*-Butyl 2-((*R*,*E*)-hept-4-en-3-yl((*S*)-1-methoxy-1-oxo-3-phenylpropane-2-yl)-carbamoyl)-3-vinylpyrrolidine-1-carboxylate (6c) and

(2*R*,3*S*)-*tert*-Butyl 2-((*R*,*E*)-hept-4-en-3-yl((*S*)-1-methoxy-1-oxo-3-phenylpropane-2-yl)carbamoyl)-3-vinyl-pyrrolidine-1-carboxylate (*dia*-6c)

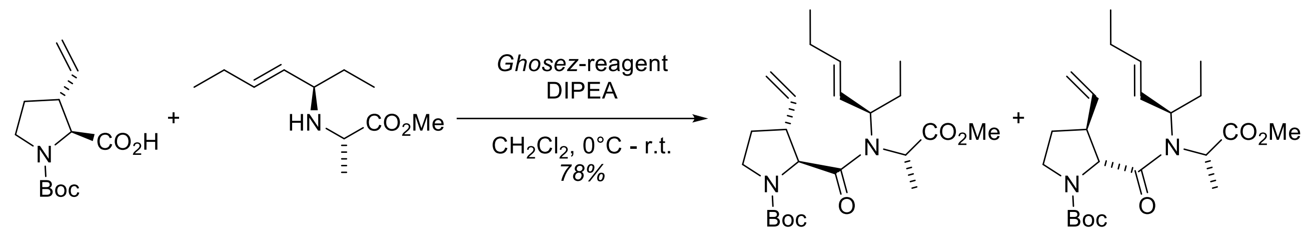

rac-**2** (*R*,*S*)-**3c** **6c** *dia*-**6c**

The reaction was performed according to the **general procedure 2** using 330 mg (1.37 mmol) racemic *Zaminer's* acid (*rac*-**2**) and stopped after 17 h.
The crude product was purified by flash column chromatography (Silica, cHex/EtOAc = 3/2) to yield 208 mg (0.376 mmol, 36%) of desired dipeptide **6c** as paly-yellow oil and 159 mg (0.376 mmol, 28%) of the diastereomer *dia*-**6c** as a pale yellow solid. Additional 82 mg (0.194 mmol, 14%) of a mixture of both products was isolated.

$C_{23}H_{38}N_2O_5$

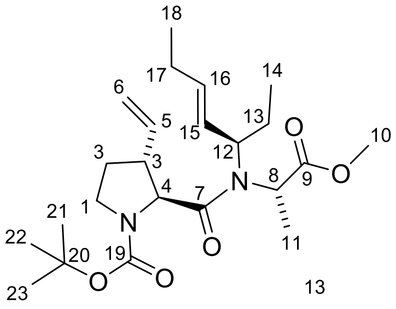

M: 422.56 g/mol.

Analytical data for **6c**:

TLC: R_f = 0.27 (Silica, cHex/EtOAc = 3/2), $KMnO_4$-reagent.

^{1}H NMR (500 MHz, $CDCl_3$, rotameric mixture[12]): δ [ppm] = 0.99 – 1.04 (m, 6H, H-14/18); 1.39 – 1.41 (m, 3H, H-11); 1.44, 1.45 (2 × s, 9H, H-21/22/23); 1.67 – 1.76 (m, 2H, H-2/13); 1.79 – 1.87 (m, 0.6H, H-13'$_{rot1}$); 1.91 – 2.00 (m, 0.4H, H-13'$_{rot2}$); 2.05 – 2.20 (m, 3H, H-2'/17); 2.71 – 2.77 (m, 1H, H-3); 3.41 – 3.49 (m, 1H, H-1); 3.57 – 3.73 (m, 5H, H-1'/8/10); 4.02 (Ψ q, J = 7.2 Hz, 0.6H, H-12$_{rot1}$); 4.10 (Ψ q, J = 7.0 Hz, 0.4H, H-12$_{rot2}$); 4.43 (s, 0.6H, H-4$_{rot1}$); 4.52 (d, ^4J = 2.1 Hz, 0.4H, H-4$_{rot2}$); 5.09 – 5.17 (m, 2H, H-6); 5.37 – 5.43 (m, 1H, H-15); 5.71 – 5.78 (m, 1H, H-16); 5.86 – 5.95 (m, 1H, H-5).

^{13}C NMR (125 MHz, $CDCl_3$, rotameric mixture[13]): δ [ppm] = 11.3, 11.4 (C-14); 13.5 (C-18); 15.7, 15.8 (C-11); 25.6, 25.8, 26.2 (C-13/17); 28.4, 28.5 (C-21/22/23); 28.7, 30.2 (C-2); 45.2, 45.7 (C-1); 46.7, 47.3 (C-3); 51.8, 51.9, 52.0, 52.2 (C-8/10); 60.4, 60.5 (C-12); 61.9, 62.6 (C-4); 79.2, 80.0 (C-20); 115.1, 115.2 (C-6); 126.0, 126.4 (C-15); 136.8, 137.0 (C-16); 138.9, 139.0 (C-5); 154.3, 154.4 (C-19); 170.0, 170.6 (C-7); 171.9, 172.5 (C-9).

IR (ATR): $\tilde{\nu}$ [cm^{-1}] = 3078 (w); 2967 (w); 2934 (w); 2877 (w); 2847 (w); 1743 (s); 1698 (s); 1653 (s); 1545 (w); 1480 (w); 1453 (m); 1432 (m); 1393 (s); 1365 (s); 1305 (m); 1215 (s); 1168 (s); 1113 (s); 1060 (m); 1047 (w); 1024 (w); 976 (m); 933 (w); 914 (m); 895 (w); 863 (m); 824 (w); 790 (w); 771 (m); 760 (w); 730 (w); 702 (w); 661 (w); 617 (w); 608 (w).

HR/MS (ESI): calculated for [M+Na]$^+$ 445.2673; found: 445.2676.

$[\boldsymbol{\alpha}]_{\boldsymbol{\lambda}}^{\mathbf{20}}$ ($CHCl_3$, c = 0.525 g/100 ml):): $[\alpha]_{365}^{20}$ = +185.8°; $[\alpha]_{436}^{20}$ = +103.8°; $[\alpha]_{546}^{20}$ = +55.9°; $[\alpha]_{579}^{20}$ = +48.1°; $[\alpha]_{589}^{20}$ = +46.0°.

Analytical data for *dia*-**6c**

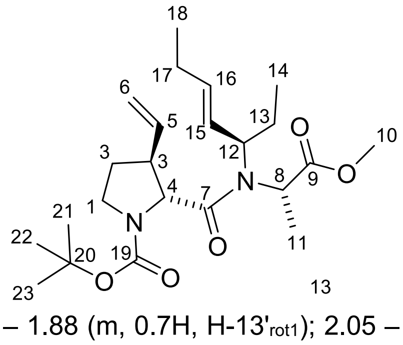

TLC: R_f = 0.19 (Silica, cHex/EtOAc = 3/2), $KMnO_4$-reagent.

Mp.: 88 – 91 °C.

^{1}H NMR (500 MHz, $CDCl_3$, rotameric mixture[13]): δ [ppm] = 0.92 – 0.97 (m, 3H, H-14); 1.01 (t, 3J = 7.5 Hz, 3H, H-18); 1.39 – 1.47 (m, 9.9H, H-11$_{rot2}$/21/22/23); 1.53 (d, 3J = 6.8 Hz, 2.1H, H-11$_{rot1}$); 1.67 – 1.77 (m, 2.3H, H-2/13/13'$_{rot2}$); 1.80 – 1.88 (m, 0.7H, H-13'$_{rot1}$); 2.05 – 2.19 (m, 2.7H, H-2'$_{rot1}$/17); 2.23 – 2.30 (m, 0.3H, H-2'$_{rot2}$); 2.84 – 2.91 (m, 1H, H-3); 3.42 – 3.49 (m, 1H, H-1); 3.53

[12] The substance forms two rotamers in 0.6/0.4 ratio.
[13] The substance forms two rotamers in 0.7/0.3 ratio.

– 3.77 (m, 5H, H-1'/8/10); 4.01 – 4.05 (m, 0.7H, H-12$_{rot1}$); 4.32 (Ψ q, J = 6.8 Hz, 0.3H, H-12$_{rot2}$); 4.40 (d, 4J = 1.2 Hz, 0.7H, H-4$_{rot1}$); 4.54 (d, 4J = 2.5 Hz, 0.3H, H-4$_{rot2}$); 5.03 – 5.19 (m, 2H, H-6); 5.37 (dd, 3J = 15.5 Hz, 3J = 7.5 Hz, 0.7H, H-15$_{rot1}$); 5.50 (dd, 3J = 15.7 Hz, 3J = 6.0 Hz, 0.3H, H-15$_{rot2}$); 5.73 (dt, 3J = 15.5 Hz, 3J = 6.3 Hz, 0.7H, H-16$_{rot1}$); 5.77 – 5.95 (m, 1.3H, H-5/16$_{rot2}$).

^{13}C NMR (125 MHz, $CDCl_3$, rotameric mixture[14]): δ [ppm] = 11.1, 11.2 (C-14); 13.4, 13.6 (C-18); 15.9, 16.4 (C-11); 25.0, 25.4, 25.5, 25.7 (C-13/17); 28.5 (C-21/22/23); 29.3, 30.6 (C-2); 45.4, 45.9 (C-1); 46.4, 47.2 (C-3); 52.1, 52.2, 52.3, 52.5 (C-8/10); 59.7, 60.3 (C-12); 61.3, 62.4 (C-4); 79.4, 79.7 (C-20); 115.2, 115.3 (C-6); 126.3, 126.7 (C-15); 136.2, 136.3 (C-16); 138.9, 138.9 (C-5); 154.0, 154.5 (C-19); 170.2, 170.9 (C-7); 172.0, 172.4 (C-9).

IR (ATR): $\tilde{\nu}$ [cm^{-1}] = 3091 (w); 2965 (w); 2950 (w); 2935 (w); 2920 (w); 2874 (w); 2847 (w); 1750 (s); 1698 (s); 1636 (s); 1549 (w); 1481 (w); 1459 (m); 1439 (m); 1392 (s); 1365 (s); 1337 (m); 1320 (m); 1306 (m); 1297 (m); 1248 (m); 1206 (s); 1164 (s); 1109 (s); 1094 (m); 1070 (w); 1060 (m); 1048 (w); 1028 (w); 993 (s); 974 (m); 951 (w); 921 (m); 890 (w); 877 (w); 865 (m); 838 (w); 817 (w); 804 (w); 786 (w); 773 (m); 761 (m); 730 (w); 703 (w); 658 (w); 628 (w).

HR/MS (ESI): calculated for $[M+Na]^+$ 445.2673; found: 445.2676.

$[\boldsymbol{\alpha}]_{\lambda}^{20}$ ($CHCl_3$, c = 0.525 g/100 ml):): $[\alpha]_{365}^{20}$ = -104.0°; $[\alpha]_{436}^{20}$ = -59.2°; $[\alpha]_{546}^{20}$ = -32.1°; $[\alpha]_{579}^{20}$ = -27.7°; $[\alpha]_{589}^{20}$ = -26.7°.

Synthesis of (3aR,6R,8aS)-*tert*-Butyl 6-ethyl-7-((S)-1-methoxy-1-oxo-3-phenylpropane-2-yl)-8-oxo-3,3a,6,7,8,8a-hexahydropyrrolo[2,3-c]azepin-1(2H)-carboxylate (7c)

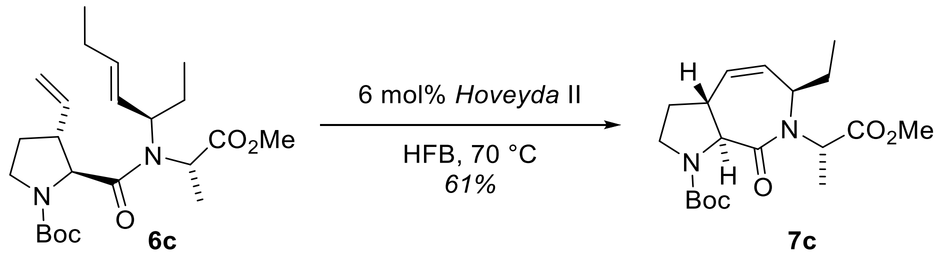

Under inert conditions a solution of 120 mg (0.285 mmol, 1.0 eq.) of dipeptide **6c** and 7.1 mg (11.4 µmol, 4 mol%) of the *Hoveyda-Grubbs II* catalyst (**9**) in 2.9 ml C_6F_6 (0.1 M) was warmed to 70 °C and stirred for 3.5 h before another 3.6 mg (5.7 µmol, 2 mol%) of *Hoveyda-Grubbs II* catalyst (**9**) were added. (Note: the mixture turns dark upon heating). After 6 h the solution was allowed to cool down before the solvent was removed under reduced pressure. The resulting dark green oil was purified by flash column chromatography (Silica, cHex/ EtOAc = 1/1). The resulting oily product was dissolved in little CH_2Cl_2 and stirred over *QuadraSil AP* for 15 min to remove residual ruthenium. After filtration the solvent was removed under reduced pressure and the product was dried in high vacuum to afford 64 mg (0.175 mmol, 61%) of the bicyclic product **7c** as a colorless viscous oil.

$C_{19}H_{30}N_2O_5$

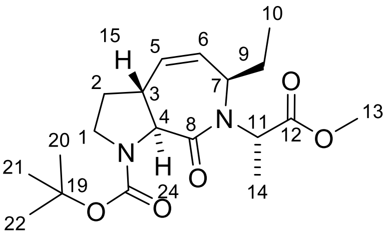

M: 366.45 g/mol.

TLC: R_f = 0.29 (Silica, cHex/EtOAc = 1/1), $KMnO_4$-reagent.

Mp: 149 - 153 °C

^{1}H NMR (500 MHz, $CDCl_3$, rotameric mixture[14]): δ [ppm] = 1.04 – 1.12 (m, 3H, H-10); 1.36 (d, ^3J = 6.7 Hz, 3H, H-14); 1.43 – 1.47 (m, 9H, H-17/18/19); 1.61 – 1.72 (m, 2H, H-2/9); 1.83 (Ψ dq, *J* = 14.8 Hz, *J* = 7.4 Hz, 1H, H-9'); 2.05 (Ψ dt, *J* = 11.3 Hz, *J* =5.5 Hz, 1H, H-2'); 2.71 – 2.78 (m, 1H, H-3); 3.40 (Ψ td, *J* =11.2 Hz, *J* = 5.6 Hz, 1H, H-1); 3.63 – 3.74 (m, 4H, H-1'/13); 3.92 (q, *J* = 6.7 Hz, 0.8H, H-11_{rot1}); 3.95 – 4.01 (m, 0.2H, H-11_{rot2}); 4.62 – 4.70 (m, 0.8H, H-7_{rot1}); 4.70 – 4.77 (m, 0.2H, H7_{rot2}); 4.88 (d, ^3J = 11.8 Hz, 1H, H-4); 5.60 (Ψ dt, *J* = 11.1 Hz, *J* = 2.8 Hz, 1H, H-6); 5.79 (Ψ dt, *J* = 11.1 Hz, *J* = 2.1 Hz, 1H, H-5).

^{13}C NMR (125 MHz, $CDCl_3$, rotameric mixture[15]): δ [ppm] = 11.5, 11.6 (C-10); 14.3, 14.6 (C-14); 26.8 (C-9); 28.2, 28.5 (C-17/18/19); 30.2, 30.7 (C-2); 43.4, 44.0 (C-3); 46.6, 47.4 (C-1); 51.0 (C-11); 52.2 (C-13); 55.3, 55.5 (C-7); 60.1, 60.2 (C-4); 79.7, 79.9 (C-16); 131.1, 131.3 (C-5); 131.8, 131.9 (C-6); 154.3, 154.7 (C-15); 171.5, 171.9 (C-12); 172.0, 172.3 (C-8).

IR (ATR): $\tilde{\nu}$ [cm^{-1}] = 3026 (w); 2980 (w); 2965 (w); 2949 (w); 2941 (w); 2884 (w); 2870 (w); 2842 (w); 1736 (s); 1699 (s); 1666 (s); 1653 (w); 1565 (w); 1535 (w); 1479 (w); 1461 (w); 1433 (m); 1404 (s); 1389 (s); 1373 (m); 1363 (m); 1346 (w); 1336 (w); 1316 (m); 1307 (m); 1273 (m); 1263 (m); 1240 (m); 1220 (s); 1159 (s); 1130 (s); 1120 (s); 1104 (m); 1085 (m); 1070 (m); 1056 (m); 1036 (w); 996 (w); 969 (m); 945 (w); 926 (m); 892 (w); 863 (m); 852 (m); 834 (m); 822 (w); 783 (m); 758 (m); 752 (m); 739 (w); 728 (m); 673 (w); 640 (m); 610 (m).

GC/MS (EI, 70 eV): m/z (%) = 366 ([M]+, 2); 337 ($[M]^+$-C_2H_5, 4); 310 (24); 293 ($[M]^+$-C_4H_9O, 28); 281 (32); 266 (38); 253 (29); 237 (30); 223 (15); 209 (23); 193 (7); 180 (44); 179 (40); 167 (16); 151 (11); 144 (95); 136 (42); 122 (41); 108 (29); 94 (20); 80 (21); 79 (22); 67 (32); 57 (100); 41 (95).

HR/MS (ESI): calculated for $[M+H]^+$: 367.2227; found: 367.2233;

calculated for $[M+Na]^+$: 389.2047; found: 389.2094.

$[\boldsymbol{\alpha}]_{\boldsymbol{\lambda}}^{\mathbf{20}}$ ($CHCl_3$, c = 0.510 g/100 ml): $[\alpha]_{365}^{20}$ = -456.9°; $[\alpha]_{436}^{20}$ = -295.8°; $[\alpha]_{546}^{20}$ = -175.2°; $[\alpha]_{579}^{20}$ = -153.9°; $[\alpha]_{589}^{20}$= -148.6°.

[14] The substance forms two rotamers in 0.8:0.2 ratio.

Synthesis of *tert*-Butyl (*R*,*E*)-*N*-(hept-3-en-3-yl)-*N*-(pent-4-enoyl)glycinate (13a)

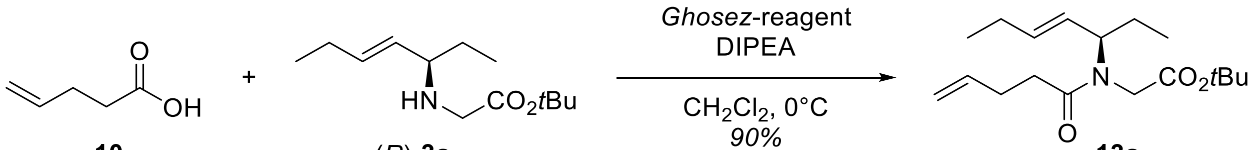

10 (*R*)-**3a** **13a**

The reaction was performed according to the **general procedure 2** using 80 µl (0.800 mmol) 4-Pentenoic acid (**10**) and stopped after 2.5 h.

The crude product was purified by flash column chromatography (Silica, cHex/EtOAc = 6/1) to yield 224 mg (0.724 mmol, 90%) of the desired dipeptide **12a** as a colorless oil.

$C_{18}H_{31}NO_3$

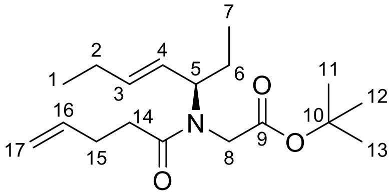

M: 309.45 g/mol.

TLC: R_f = 0.21 (Silica, cHex/EtOAc = 6/1), $KMnO_4$-reagent.

^{1}H NMR (500 MHz, $CDCl_3$, rotameric mixture[15]): δ [ppm] = 0.87 (t, ^3J = 7.4 Hz, 1.2H, H-7_{rot2}); 0.94 – 1.00 (m, 4.8H, H-1/7_{rot1}); 1.42 – 1.51 (m, 9.4H, H-6_{rot2}/11/12/13); 1.51 – 1.66 (m, 1.6H, H-6_{rot1}/6'); 2.00 – 2.08 (m, 2H, H-2); 2.30 – 2.33 (m, 0.8H, H-15_{rot2}/$15'_{rot2}$); 2.40 – 2.45 (m, 2H, H-15_{rot1}/16/$16'_{rot2}$); 2.48 – 2.51 (m, 1.2H, H-$15'_{rot1}$/$16'_{rot1}$); 3.71 – 3.82 (m, 2H, H-8); 4.17 (Ψ q, J = 6.4 Hz, 0.6H, H-5_{rot1}); 4.96 – 5.00 (m, 1H, H-18); 5.03 – 5.09 (m, 1.4H, H-5_{rot2}/18'); 5.32 (ddt, ^3J = 15.6 Hz, ^3J = 6.1 Hz, ^4J = 1.5 Hz, 0.4H, H-4_{rot2}); 5.39 (ddt, ^3J = 15.6 Hz, ^3J = 5.6 Hz, ^4J =1.5 Hz, 0.6H, H-4_{rot1}); 5.60 – 5.67 (m, 1H, H-3); 5.82 – 5.92 (m, 1H, H-17).

^{13}C NMR (125 MHz, $CDCl_3$, rotameric mixture[17]): δ [ppm] = 11.0, 11.2 (C-7); 13.6, 13.6 (C-1); 24.4 (C-6_{rot2}); 25.6, 25.7 (C-2); 25.8 (C-6_{rot1}); 28.1, 28.2 (C-11/12/13); 29.4, 29.4 (C-16); 32.7, 33.0 (C-15); 44.6, 46.1 (C-8); 56.0, 60.1 (C-5); 81.1, 82.2 (C-10); 115.1, 115.2 (C-18); 127.1, 127.1 (C-4); 135.1, 135.6 (C-3); 137.8, 137.9 (C-17); 168.7, 169.1 (C-9); 172.6, 172.9 (C-14).

IR (ATR): $\tilde{\nu}$ [cm^{-1}] = 3078 (w); 3005 (w); 2967 (w); 2935 (w); 2878 (w); 2855 (w); 1746 (m); 1647 (m); 1437 (m); 1408 (m); 1395 (w); 1368 (m); 1304 (w); 1255 (w); 1219 (m); 1150 (s); 1058 (w); 1032 (w); 975 (m); 940 (w); 910 (m); 848 (w); 793 (w); 783 (w); 749 (w); 641 (w).

GC/MS (EI, 70 eV): m/z (%) = 309 ($[M]^+$, 2); 280 ($[M]^+$-C_2H_5, 7); 253 (13); 238 (8); 224 (12); 210 (8); 208 ($[M]^+$-CO_2tBu, 9); 198 (16); 194 (11); 170 (48); 142 (100); 112 (25); 97 ($[C_7H_{13}]^+$, 52); 81 (10); 69 (10); 57 (40); 55 (75); 41 (30).

[15] The substance forms two rotamers in 0.6/0.4 ratio.

HR/MS (ESI): calculated for $[M+Na]^+$ 310.2377; found: 310.2380; $[M+Na]^+$ 332.2196; found: 332.2196.

$[\alpha]_{\lambda}^{20}$ (CHCl3, c = 0.500 g/100 ml):): $[\alpha]_{365}^{20}$ = +216.4°; $[\alpha]_{436}^{20}$ = +125.9°; $[\alpha]_{546}^{20}$ = +69.5°; $[\alpha]_{579}^{20}$ = +60.5°; $[\alpha]_{589}^{20}$ = +57.4°.

Synthesis of *tert*-Butyl (*R*)-2-(7-ethyl-2-oxo-2,3,4,7-tetrahydro-1*H*-azepin-1-yl)acetate (11a)

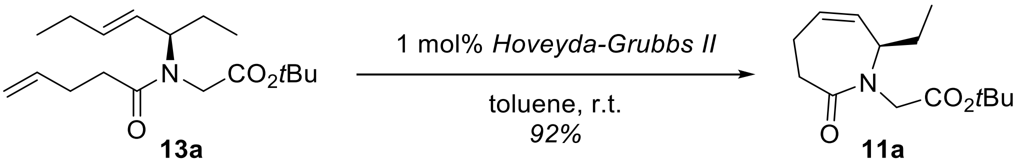

Under inert conditions a solution of 150 mg (0.485 mmol, 1.0 eq.) of peptide **12a** and 3.0 mg (4.9 µmol, 1 mol%) of the *Hoveyda-Grubbs II* catalyst (**9**) in 24.3 ml toluene (0.02 M) was stirred for 1 h at room temperature. After completion 200 mg *QuadraSil AP* ® was added and the solution stirred for another 1 h. The mixture was filtrated and the solvent was removed under reduced pressure before the colorless crude product was purified by flash column chromatography (Silica, cHex/ EtOAc = 2/1). The product **11a** was isolated as viscose colorless oil in 92% (113 mg, 0.446 mmol) yield and crystallized at 4 °C to a white solid.

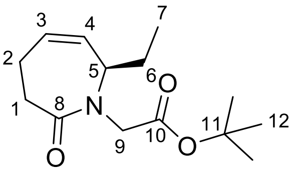

$C_{14}H_{23}NO_3$

M: 253.34 g/mol.

TLC: R_f = 0.20 (Silica, cHex/EtOAc = 3/2), $KMnO_4$-reagent.

Mp.: 56 – 58 °C.

^{1}H NMR (500 MHz, CDCl3): δ [ppm] = 1.00 (t, ^3J = 7.4 Hz, 3H, H-7); 1.45 (s, 9H, H-12/13/14); 1.75 – 1.84 (m, 1H, H-6); 1.86 – 1.94 (m, 1H, H-6'); 2.25 – 2.32 (m, 1H, H-2); 2.37 – 2.46 (m, 1H, H-2'); 2.61 – 2.65 (m, 1H, H-1); 2.80 – 2.86 (m, 1H, H-1'); 3.56 (d, ^2J = 17.1 Hz, 1H, H-9); 3.67 (Ψ q, ^3J = 6.9 Hz, 1H, H-5); 4.53 (d, ^3J = 17.1 Hz, 1H, H-9'); 5.68 – 5.72 (m, 1H, H-4); 5.79 – 5.83 (m, 1H, H-3).

^{13}C NMR (125 MHz, CDCl3): δ [ppm] = 11.8 (C-7); 24.5 (C-2); 28.1 (C-12/13/14); 29.5 (C-6); 5.2 (C-1); 51.8 (C-9); 61.8 (C-5); 81.7 (C-11); 128.5 (C-4); 129.7 (C-3); 168.8 (C-10); 174.1 (C-8).

IR (ATR): $\tilde{\nu}$ [cm^{-1}] = 3429 (w); 3262 (w); 3028 (w); 3003 (w); 2974 (m); 2940 (w); 2924 (w); 2899 (w); 2881 (w); 2848 (w); 1723 (s); 1671 (w); 1634 (s); 1600 (w); 1491 (m); 1465 (w); 1455 (m); 1404 (w); 1392 (w); 1364 (m); 1327 (m); 1295 (m); 1267 (m); 1255 (m); 1214 (m); 1181 (m); 1156 (s); 1193 (w); 1044 (w); 1035 (w); 1014 (w); 986 (w); 972 (w); 949 (m); 931 (m); 872 (w); 861 (w); 852 (m); 825 (w); 784 (w); 759 (w); 750 (w); 696 (m); 648 (w); 618 (w); 604 (w).

GC/MS (EI, 70 eV): m/z (%) = 309 ([M]$^+$, 2); 280 ([M]$^+$-C_2H_5, 7); 253 (13); 238 (8); 224 (12); 210 (8); 208 ([M]$^+$-CO_2*t*Bu, 9); 198 (16); 194 (11); 170 (48); 142 (100); 112 (25); 97 ([C_7H_{13}]$^+$, 52); 81 (10); 69 (10); 57 (40); 55 (75); 41 (30).

HR/MS (ESI): calculated for [M+Na]$^+$ 254.1751; found: 254.1752; [M+Na]$^+$ 276.1570; found: 276.1572.

$[\alpha]_{\lambda}^{20}$ ($CHCl_3$, c = 0.510 g/100 ml): $[\alpha]_{365}^{20}$ = -308.6°; $[\alpha]_{436}^{20}$ = -183.7°; $[\alpha]_{546}^{20}$ = -102.8°; $[\alpha]_{579}^{20}$ = -89.7°; $[\alpha]_{589}^{20}$ = -86.1°.

Synthesis of *tert*-Butyl (*R*,*E*)-*N*-(hept-3-en-3-yl)-*N*-(pent-4-enoyl)-*L*-phenylalaninate (11b)

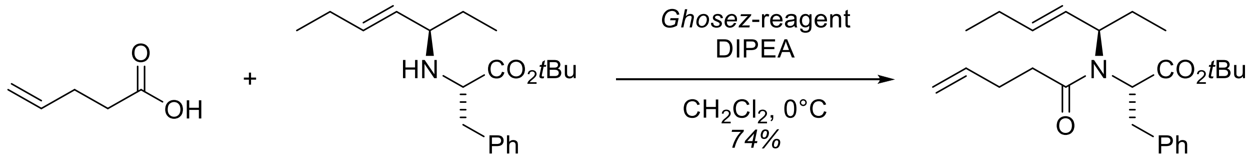

10 (*R,S*)-**3e** **13b**

The reaction was performed according to the **general procedure 2** using 50 µl (0.495 mmol) 4-Pentenoic acid (**10**) and stopped after 1 h. The crude product was purified by flash column chromatography (Silica, cHex/EtOAc = 5/1) to yield 131 mg (0.366 mmol, 74%) of the desired peptide **12b** as a colorless oil.

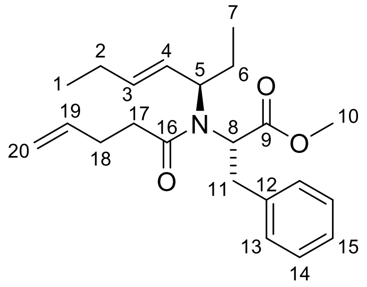

$C_{22}H_{31}NO_3$

M: 357.49 g/mol.

TLC: R_f = 0.20 (Silica, cHex/EtOAc = 5/1), $KMnO_4$-reagent.

^{1}H NMR (500 MHz, $CDCl_3$, rotameric mixture[16]): δ [ppm] = 0.90 – 0.94 (m, 6H, H-1/7); 1.57 – 1.74 (m, 2H, H-6); 1.89 (Ψ quint., ^3J = 7.2 Hz, 2H, H-2); 2.41 – 2.50 (m, 4H, H-17/18); 3.06 (dd, ^2J = 14.0 Hz, ^3J = 6.2 Hz, 1H, H-11); 3.64 (dd, ^3J = 14.0 Hz, ^3J = 7.0 Hz, 1H, H-11'); 3.69 (s, 3H, H-10); 3.82 (Ψ t, ^3J = 6.6 Hz, 1H, H-8); 4.04 (Ψ q, ^3J = 7.0 Hz, 1H, H-5); 4.75 (ddt, ^3J = 15.6 Hz, ^3J = 6.8 Hz, ^4J = 1.4 Hz, 1H, H-4); 5.01 – 5.10 (m, 2H, H-20); 5.52 (dt, ^3J = 15.6 Hz, ^3J = 6.4 Hz, 1H, H-3); 5.86 – 5.94 (m, 1H, H-19); 7.18 – 7.20 (m, 3H, H-13/15); 7.14 – 7.27 (m, 2H, H-14).

^{13}C NMR (125 MHz, $CDCl_3$, rotameric mixture[18]): δ [ppm] = 11.4 (C-7); 13.5 (C-1); 25.6 (C-6); 25.7 (C-2); 29.1 (C-18); 33.1 (C-17); 36.5 (C-11); 52.1 (C-10); 58.6 (C-8); 61.2 (C-5); 115.2 (C-20); 126.3 (C-15); 127.1 (C-4); 128.2 (C-14); 129.7 (C-13); 135.9 (C-3); 137.6 (C-19); 139.6 (C-12); 171.7 (C-9); 172.0 (C-16).

[16] The substance forms two rotamers in 1.0/0.8 ratio. Because of the weak signal intensity only the main rotamer is given.

IR (ATR): $\tilde{\nu}$ [cm^{-1}] = 3107 (w); 3084 (w); 3065 (w); 3028 (w); 2963 (w); 2934 (w); 2875 (w); 2851 (w); 1740 (s); 1639 (s); 1605 (w); 1585 (w); 1497 (w); 1455 (m); 1430 (s); 1378 (w); 1351 (w); 1300 (m); 1217 (s); 1182 (m); 1162 (m); 1153 (m); 1115 (w); 1080 (m); 1065 (m); 1030 (m); 1021 (m); 977 (m); 910 (m); 846 (w); 796 (w); 751 (m); 700 (s); 646 (w); 623 (w).

GC/MS (EI, 70 eV): m/z (%) = 357 ([M]$^+$, 8); 342 ([M]$^+$-CH_3, 5); 328 ([M]$^+$-C_2H_5, 6); 298 ([M]$^+$-CO_2Me, 7); 274 ([M]$^+$-C_5H_7O, 18); 246 (73); 202 (10); 194 (15); 184 (22); 170 (16); 166 (10); 162 (7); 120 (12); 103 (11); 97 ([C_7H_{13}]$^+$, 35); 91 (30); 81 (14); 67 (13); 55 (100).

HR/MS (ESI): calculated for [M+Na]$^+$ 358.2377; found: 358.2377; [M+Na]$^+$ 380.2169; found: 380.2194.

$[\alpha]_{\lambda}^{20}$ ($CHCl_3$, c = 0.505 g/100 ml): $[\alpha]_{365}^{20}$ = -231.2°; $[\alpha]_{436}^{20}$ = -136.0°; $[\alpha]_{546}^{20}$ = -75.5°; $[\alpha]_{579}^{20}$ = -65.7°; $[\alpha]_{589}^{20}$ = -63.7°.

Synthesis of *tert*-Butyl (*S*)-((R)-7-ethyl-2-oxo-2,3,4,7-tetrahydro-1*H-azepin-1-yl)-3-phenylpropanoate* (11b)

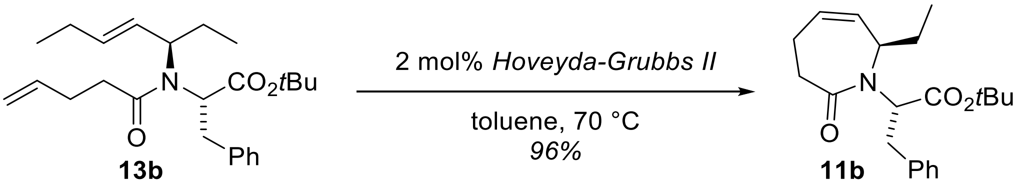

Under inert conditions a solution of 125 mg (0.350 mmol, 1.0 eq.) of peptide **12b** and 4.4 mg (7.0 µmol, 2 mol%) of the *Hoveyda-Grubbs II* catalyst (**9**) in 24.3 ml toluene (0.02 M) was heated to 70 °C and stirred for 3 h. After completion 200 mg *QuadraSil AP* ® was added and the solution stirred for another 1 h. The solution was allowed to cool down before the solvent was removed under reduced pressure. The resulting dark green oil was purified by flash column chromatography (Silica, cHex/ EtOAc = 3/2). The resulting oily product was dissolved in little CH_2Cl_2 and stirred over *QuadraSil AP* for 15 min to remove residual ruthenium. After filtration the solvent was removed under reduced pressure and the product was dried in high vacuum to afford 101 mg (0.335 mmol, 96%) of the cyclic product **11b** as a colorless viscous oil.

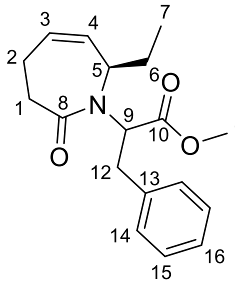

$C_{18}H_{23}NO_3$

M: 301.38 g/mol.

TLC: R_f = 0.24 (Silica, cHex/EtOAc = 3/2), $KMnO_4$-reagent.

Mp.: 75 – 76 °C

^{1}H NMR (500 MHz, $CDCl_3$): δ [ppm] = 0.90 (t, ^3J = 7.5 Hz, 3H, H-7); 1.53 – 1.61 (m, 1H, H-6); 1.71 – 1.80 (m, 1H, H-6'); 1.92 – 2.02 (m, 1H, H-2); 2.10 – 2.16 (m, 1H, H-2'); 2.49 (ddd, ^2J = 13.5 Hz, ^3J = 5.7 Hz, ^3J = 3.0 Hz, 1H, H-1); 2.84 (Ψ td, $^{2,3}J$ = 13.1 Hz, ^3J = 3.6 Hz, 1H, H-1'); 2.99 (dd, ^3J = 14.3 Hz, ^3J = 9.1 Hz, 1H, H-12); 3.29 (dd, ^3J =

14.3 Hz, ^3J = 6.4 Hz, 1H, H-12'); 3.69 – 3.74 (m, 4H, H-5/11); 5.31 (dd, ^3J = 9.1 Hz, ^3J = 6.4 Hz, 1H, H-9); 5.59 – 5.66 (m, 2H, H-3/4); 7.15 – 7.24 (m, 5H, H-14/15/16).

^{13}C NMR (125 MHz, $CDCl_3$): δ [ppm] = 12.0 (C-7); 24.3 (C-2); 30.7 (C-6); 35.2 (C-1); 35.8 (C-12); 52.1 (C-11); 57.3 (C-5); 59.3 (C-9); 126.6 (C-16); 128.0 (C-4); 128.2 (C-15); 129.5 (C-14); 130.1 (C-3); 137.3 (C-13); 171.7 (C-10); 174.3 (C-8).

IR (ATR): $\tilde{\nu}$ [cm^{-1}] = 3463 (w); 3259 (w); 3111 (w); 3087 (w); 3072 (w); 3028 (w); 2970 (w); 2947 (w); 2928 (w); 2911 (w); 2891 (w); 2855 (w); 2839 (w); 1740 (s); 1700 (w); 1630 (s); 1608 (w); 1498 (m); 1449 (m); 1433 (s); 1416 (m); 1384 (w); 1345 (m); 1322 (w); 1309 (w); 1295 (m); 1283 (m); 1263 (m); 1245 (m); 1223 (s); 1209 (m); 1192 (s); 1176 (m); 1162 (m); 1153 (m); 1124 (m); 1100 (w); 1081 (m); 1047 (w); 1029 (s); 1010 (m); 993 (w); 962 (w); 954 (w); 941 (w); 930 (w); 920 (w); 907 (m); 869 (w); 853 (w); 819 (m); 771 (m); 747 (s); 703 (s); 672 (w); 658 (w); 618 (s); 602 (w).

GC/MS (EI, 70 eV): m/z (%) = 301 ([M]$^+$, 12); 272 ([M]$^+$-C_2H_5, 100); 242 ([M]$^+$-CO_2Me, 50); 210 ([M]$^+$-C_7H_7, 87); 206 (55); 182 (73); 180 (26); 178 (72); 162 (23); 150 (25); 146 (60); 139 (34); 138 (35); 131 (20); 128 (22); 120 (55); 110 (35); 103 (27); 95 (60); 93 (38); 91 ([C7H7]+, 95); 81 (27); 77 (30); 67 (78); 65 (34); 59 (20); 55 (35); 53 (25).

HR/MS (ESI): calculated for [M+Na]$^+$ 302.1751; found: 302.1753; [M+Na]$^+$ 324.1570; found: 324.1569.

$[\boldsymbol{\alpha}]_{\boldsymbol{\lambda}}^{\mathbf{20}}$ ($CHCl_3$, c = 0.510 g/100 ml): $[\alpha]_{365}^{20}$ = -818.2°; $[\alpha]_{436}^{20}$ = -475.8°; $[\alpha]_{546}^{20}$ = -262.5°; $[\alpha]_{579}^{20}$ = -228.1°; $[\alpha]_{589}^{20}$ = -219.1°.

NMR Spectra

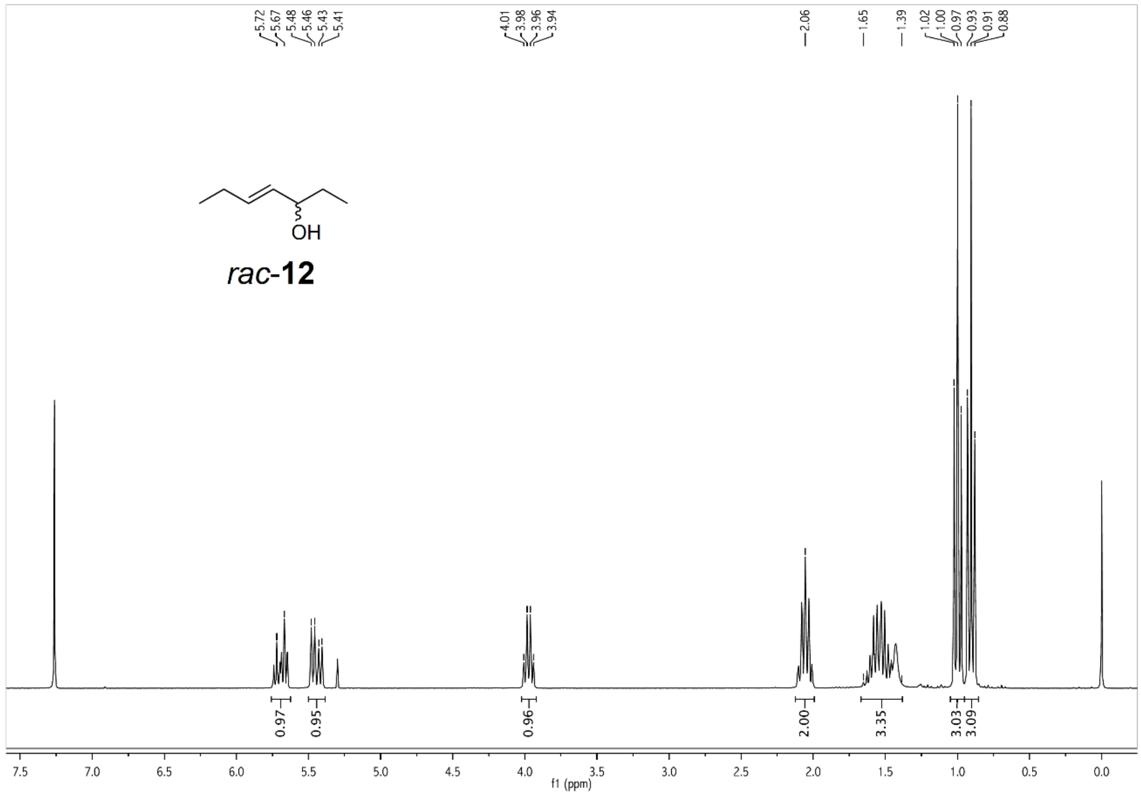

^{1}H NMR (300 MHz) of *rac*-**12** in $CDCl_3$

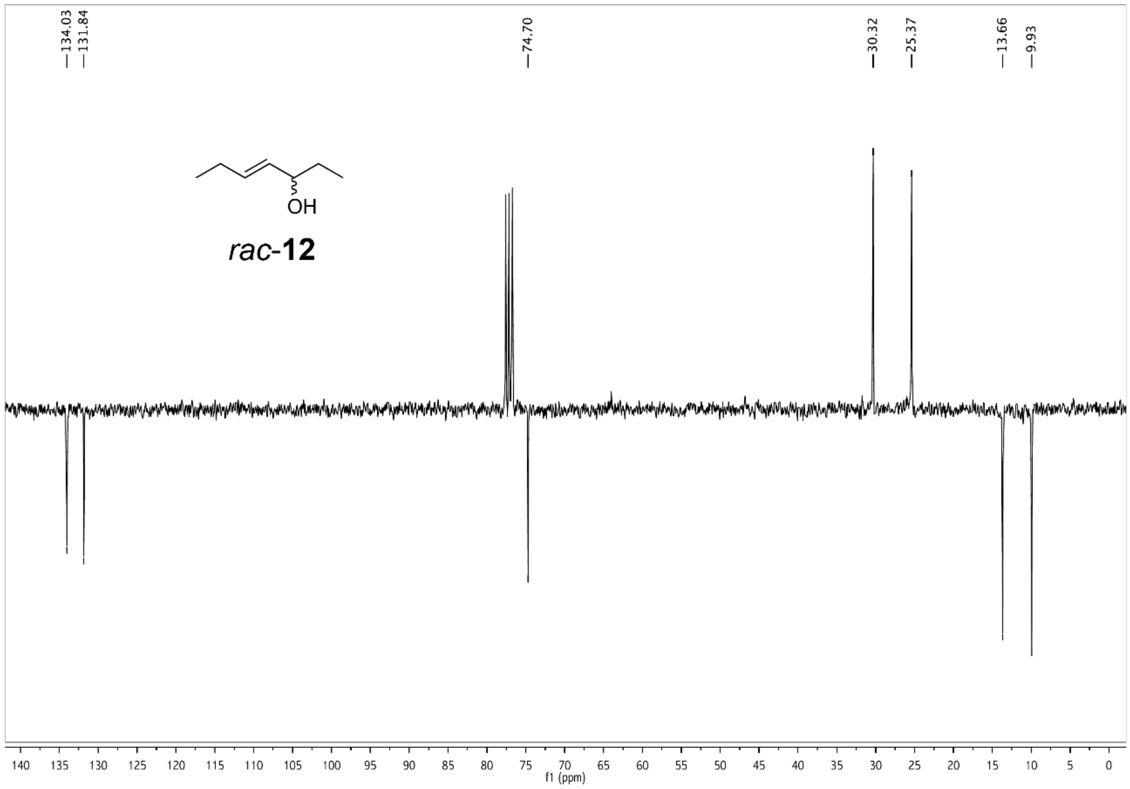

^{13}C-NMR (75 MHz) of *rac*-**12** in $CDCl_3$

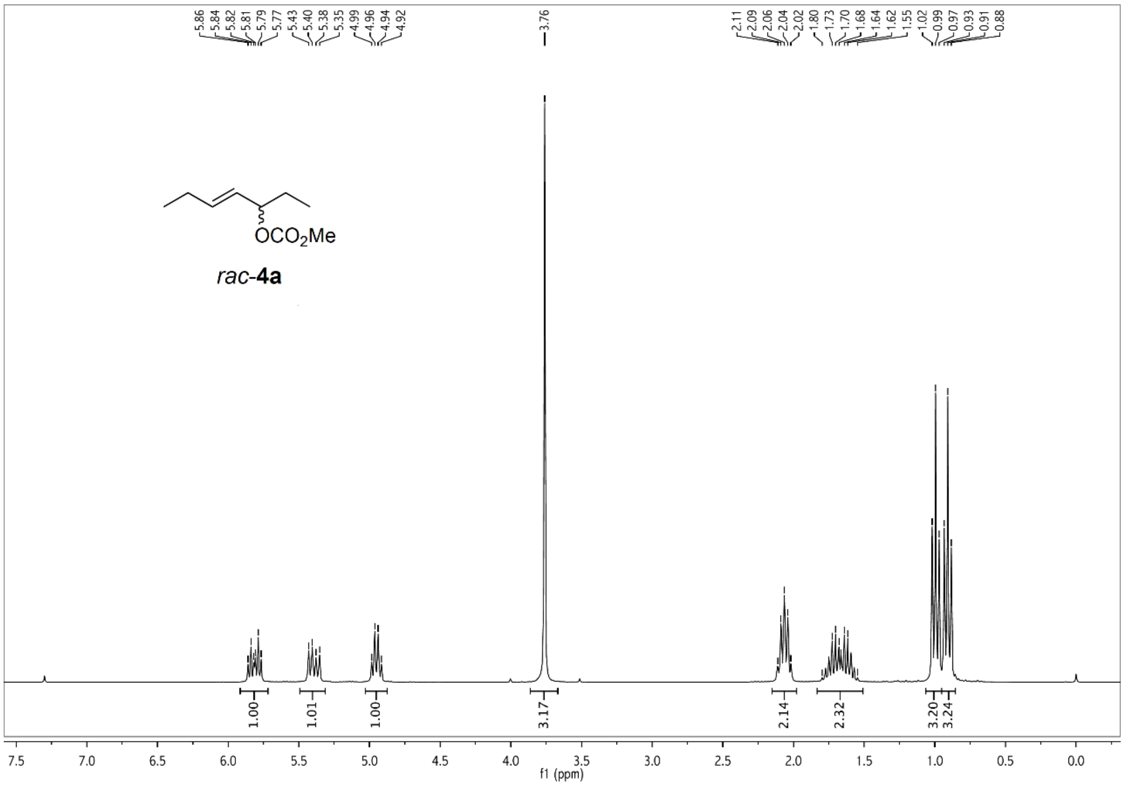

^{1}H NMR (300 MHz) of *rac*-**4a** in $CDCl_3$

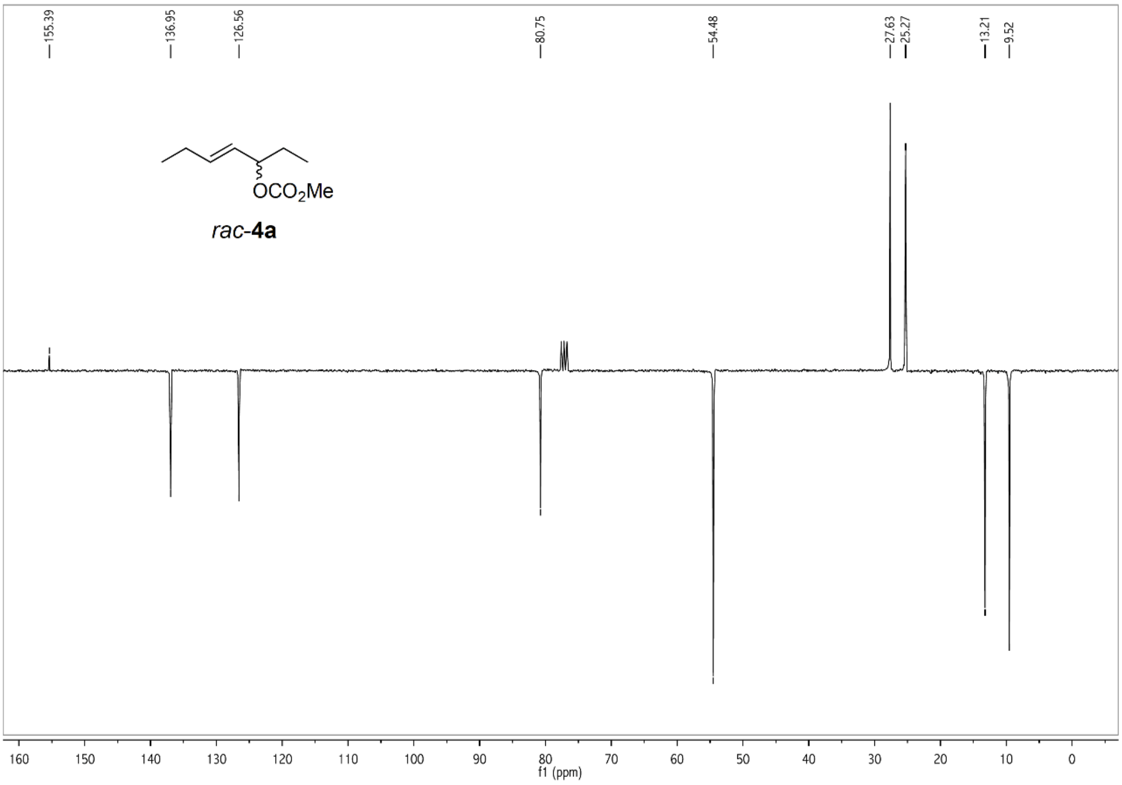

^{13}C-NMR (75 MHz) of *rac*-**4a** in $CDCl_3$

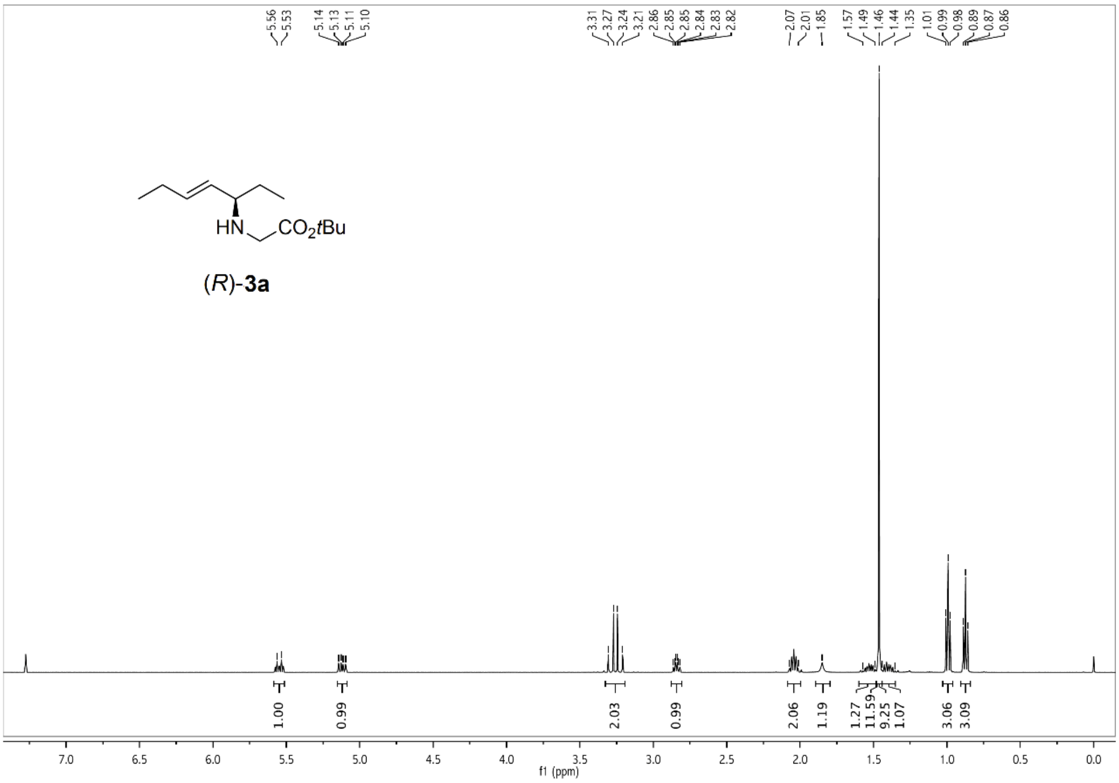

^{1}H NMR (300 MHz) of *rac*-**3a** in $CDCl_3$

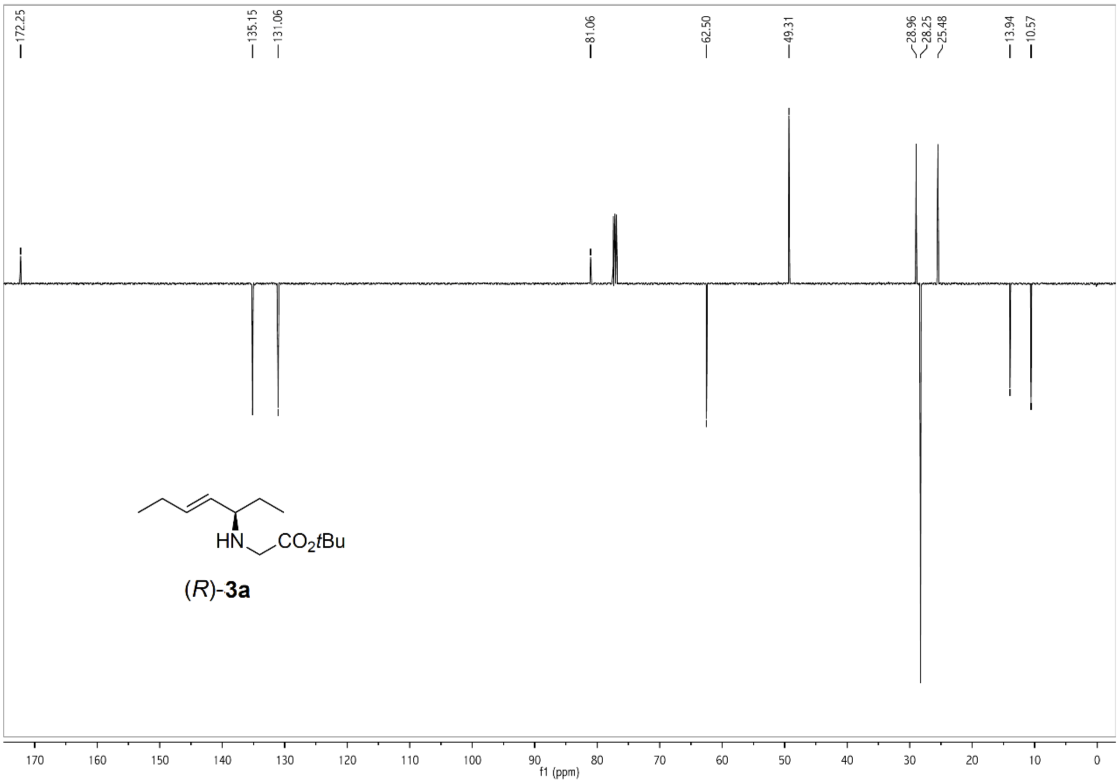

^{13}C-NMR (75 MHz) of *rac*-**3a** in $CDCl_3$

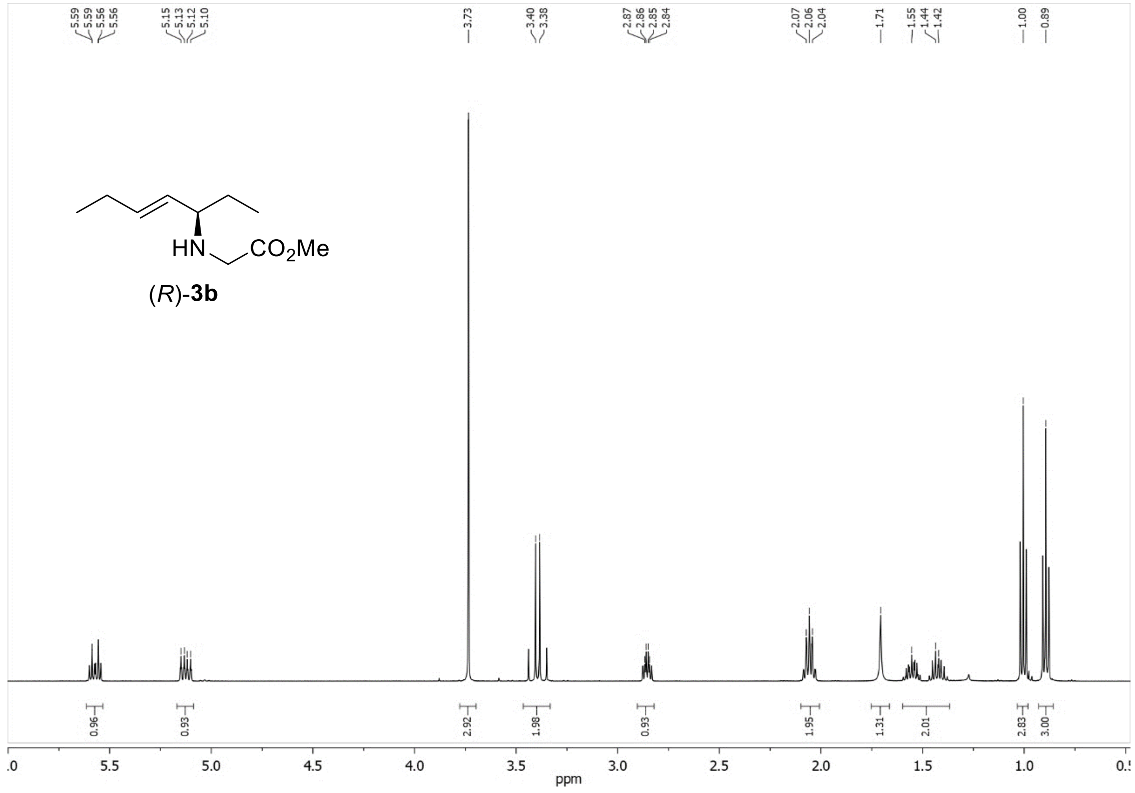

^{1}H NMR (300 MHz) of *rac*-**3b** in $CDCl_3$

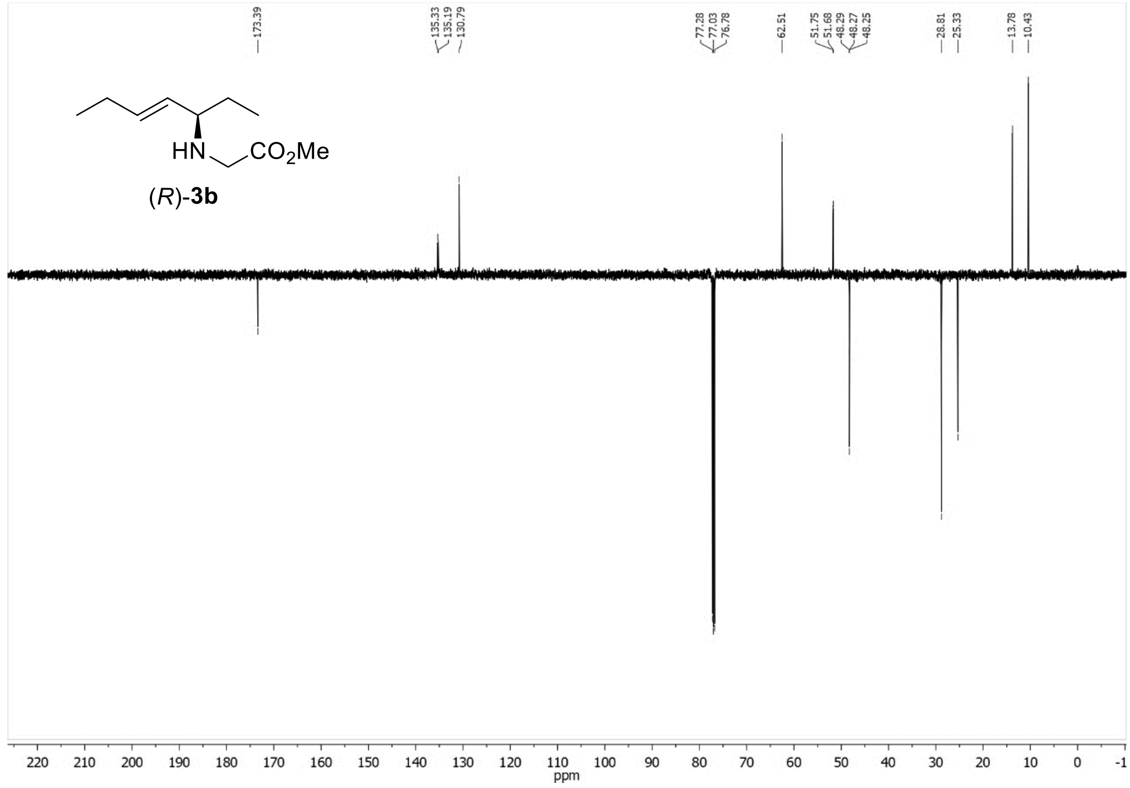

^{13}C-NMR (75 MHz) of *rac*-**3b** in $CDCl_3$

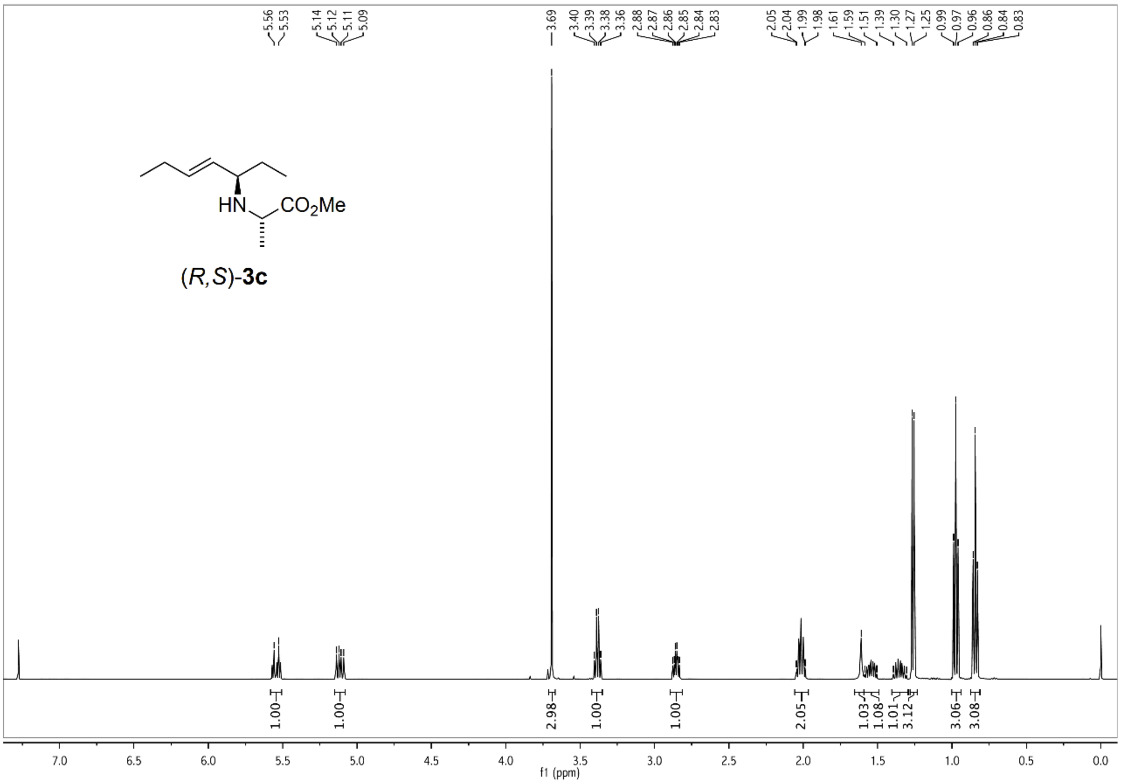

^{1}H NMR (500 MHz) of (*R,S*)-**3c** in $CDCl_3$

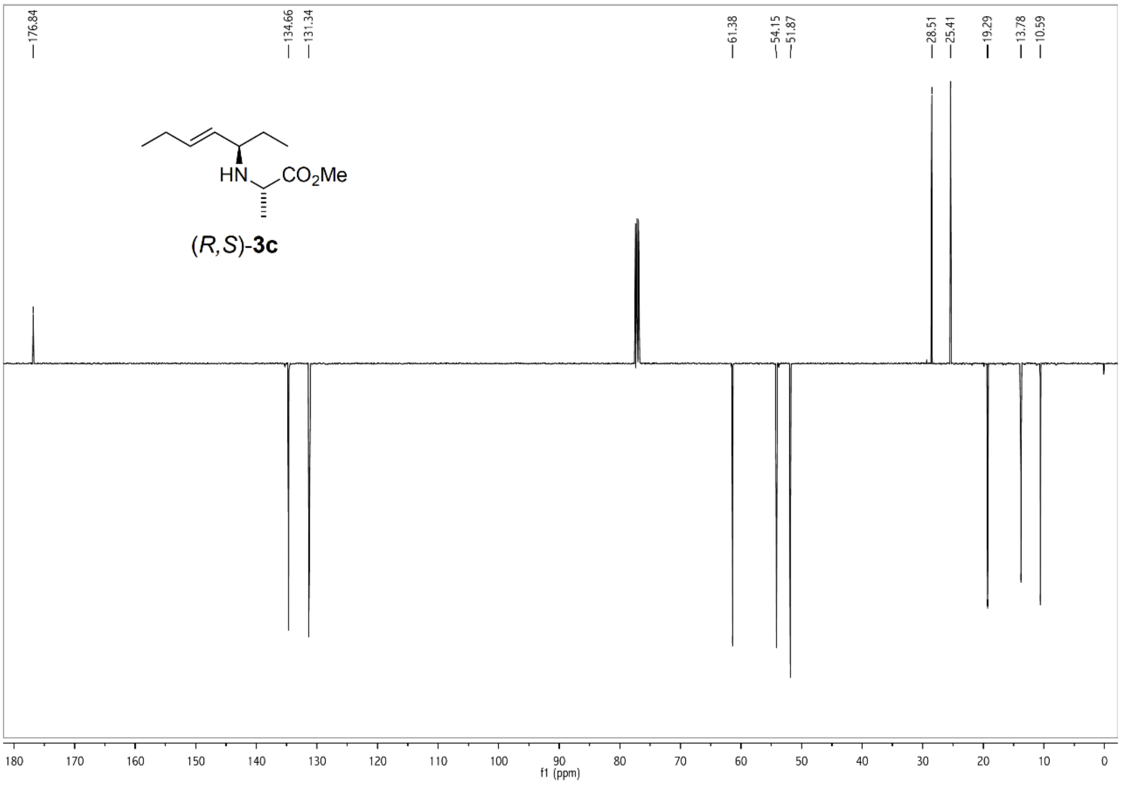

^{13}C-NMR (125 MHz) of (*R,S*)-**3c** in $CDCl_3$

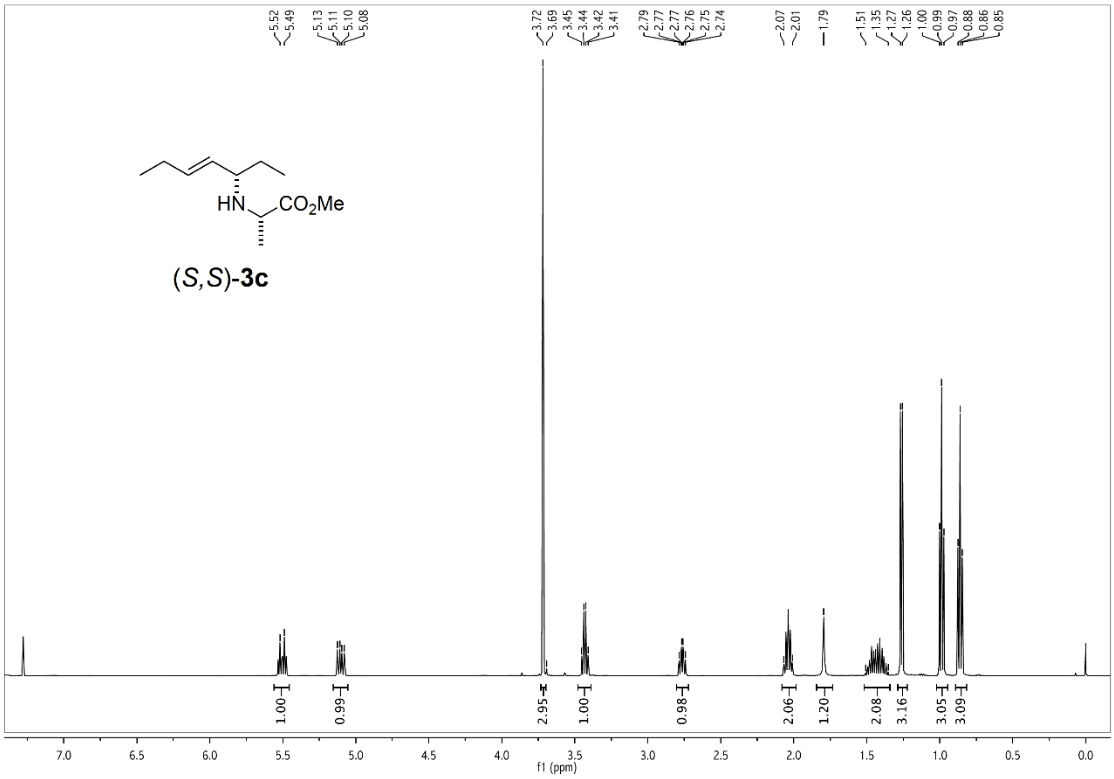

^{1}H NMR (500 MHz) of (*S,S*)-**3c** in $CDCl_3$

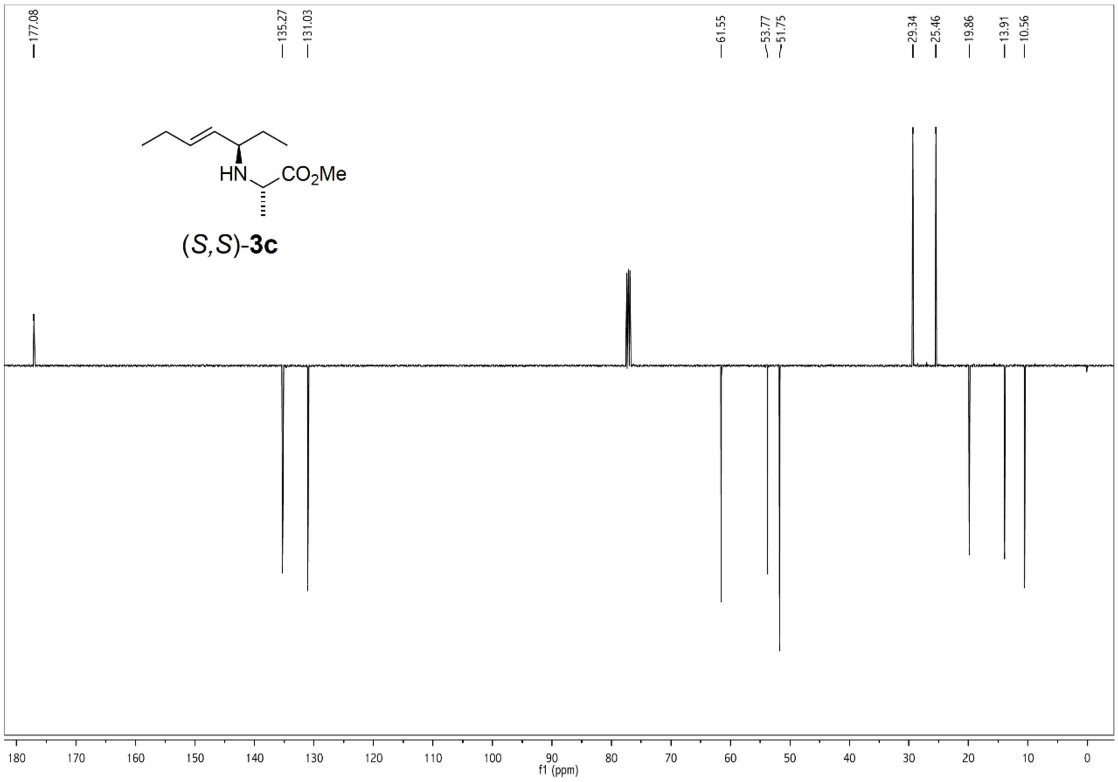

^{13}C-NMR (125 MHz) of (*S,S*)-**3c** in $CDCl_3$

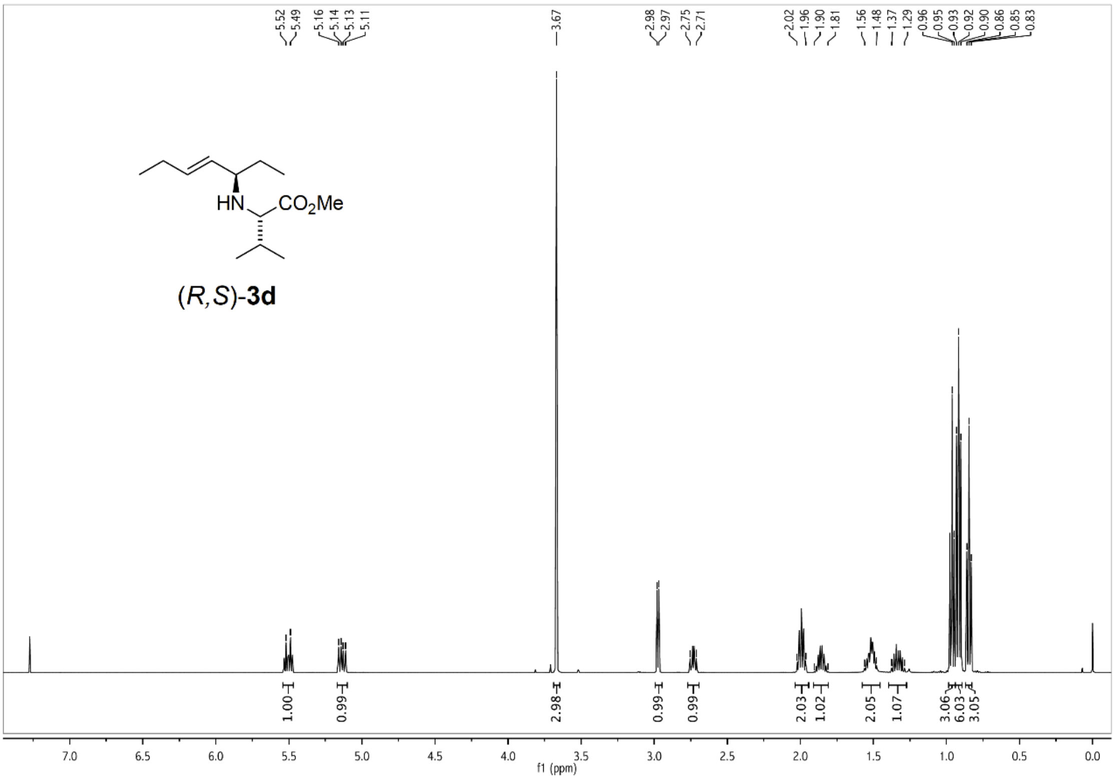

^{1}H NMR (500 MHz) of (*R,S*)-**3d** in $CDCl_3$

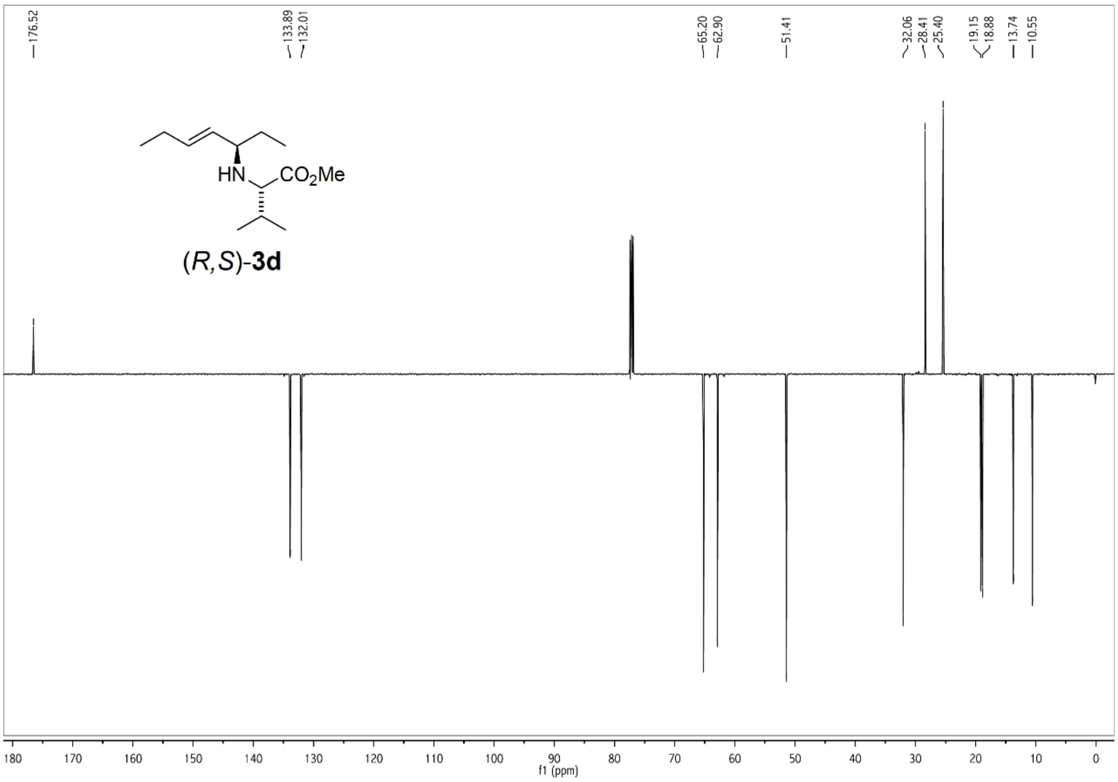

^{13}C-NMR (125 MHz) of (*R,S*)-**3d** in $CDCl_3$

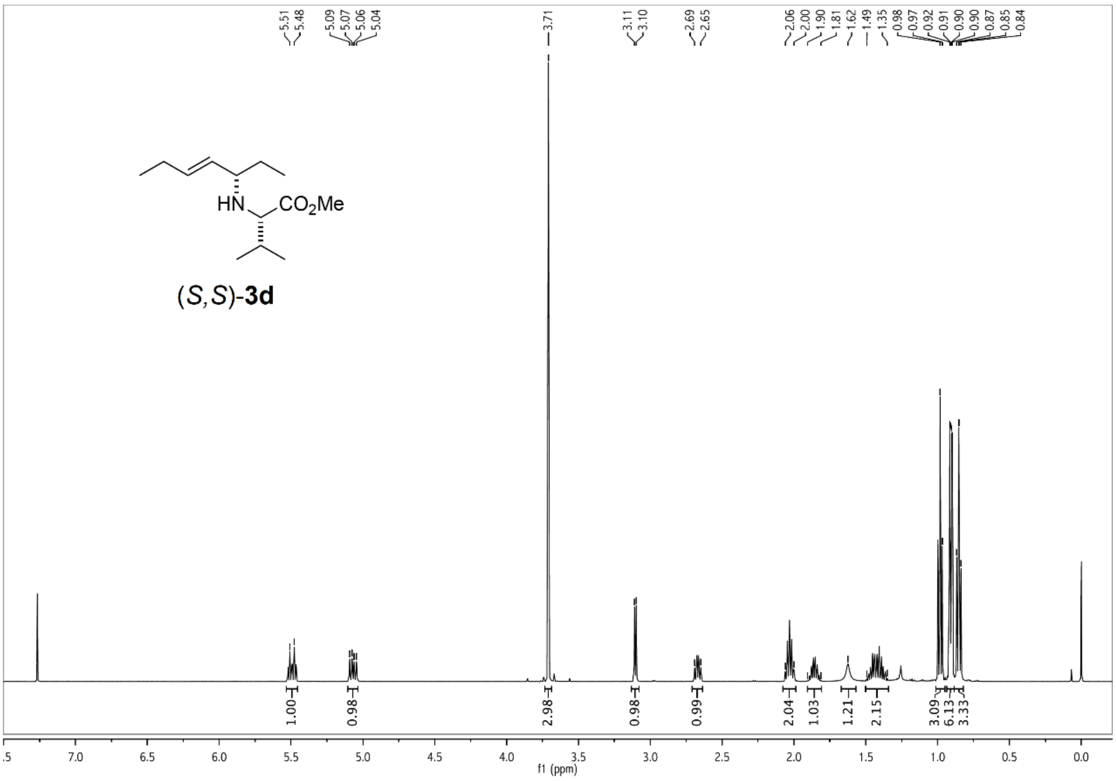

^{1}H NMR (500 MHz) of (S,*S*)-**3d** in $CDCl_3$

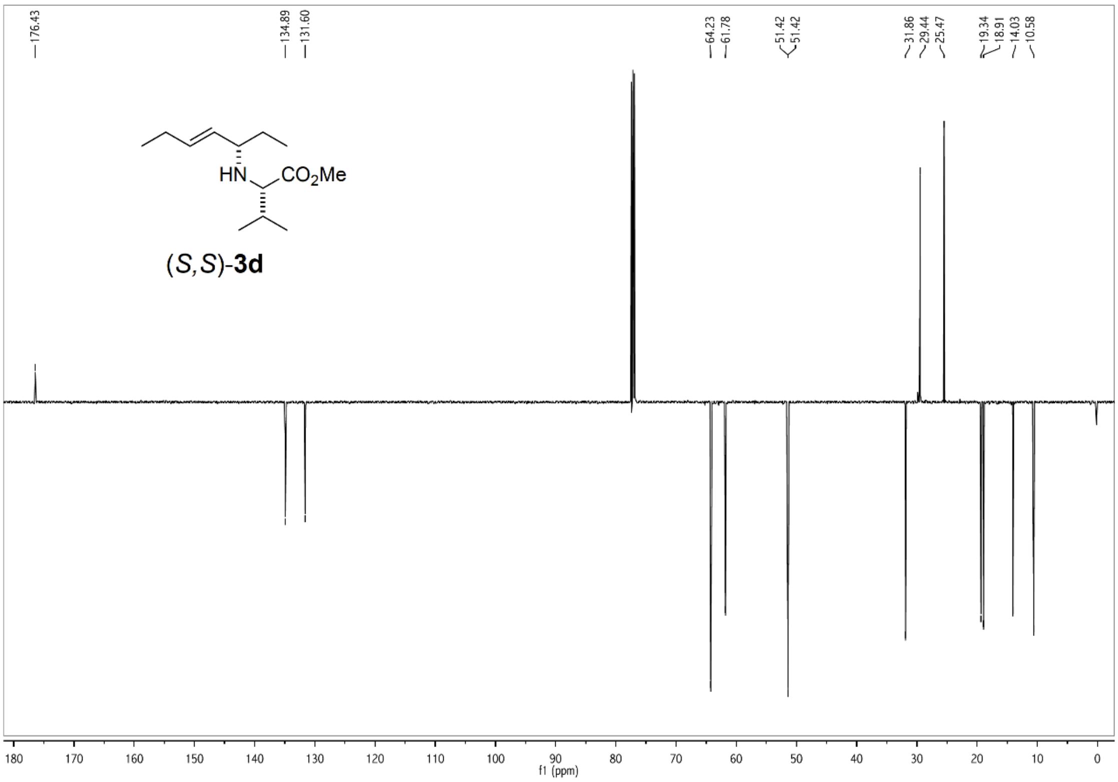

^{13}C-NMR (125 MHz) of (*S,S*)-**3d** in $CDCl_3$

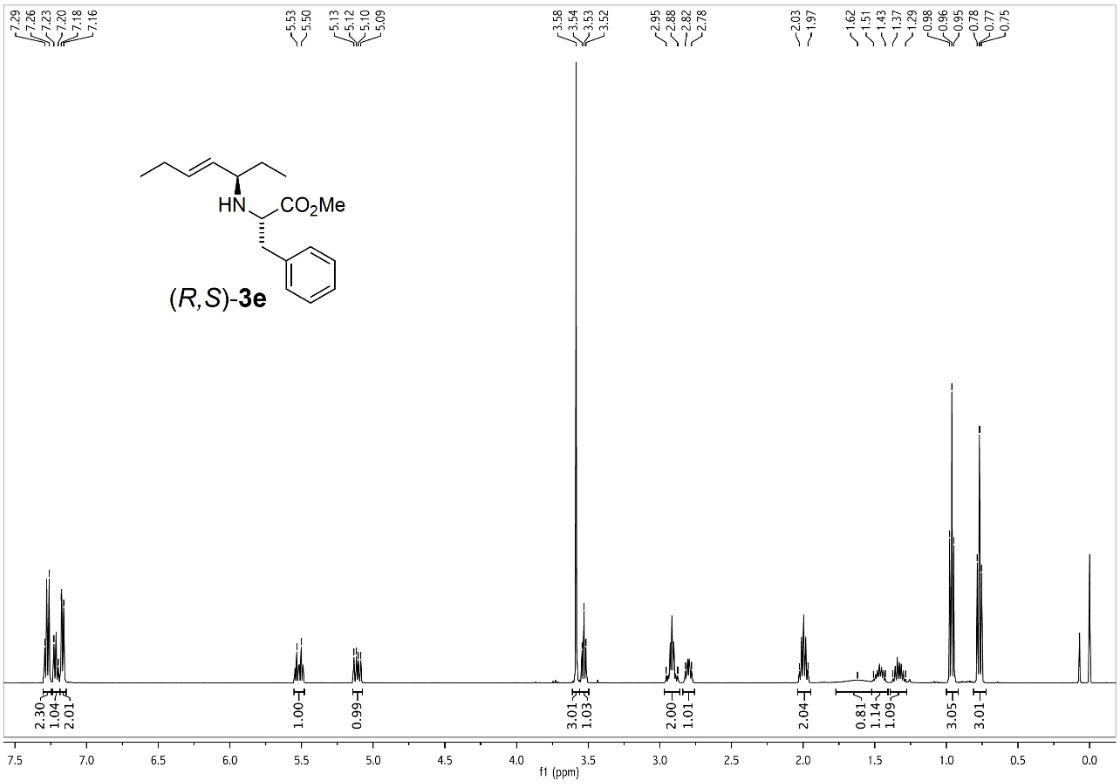

^{1}H NMR (500 MHz) of (*R,S*)-**3e** in $CDCl_3$

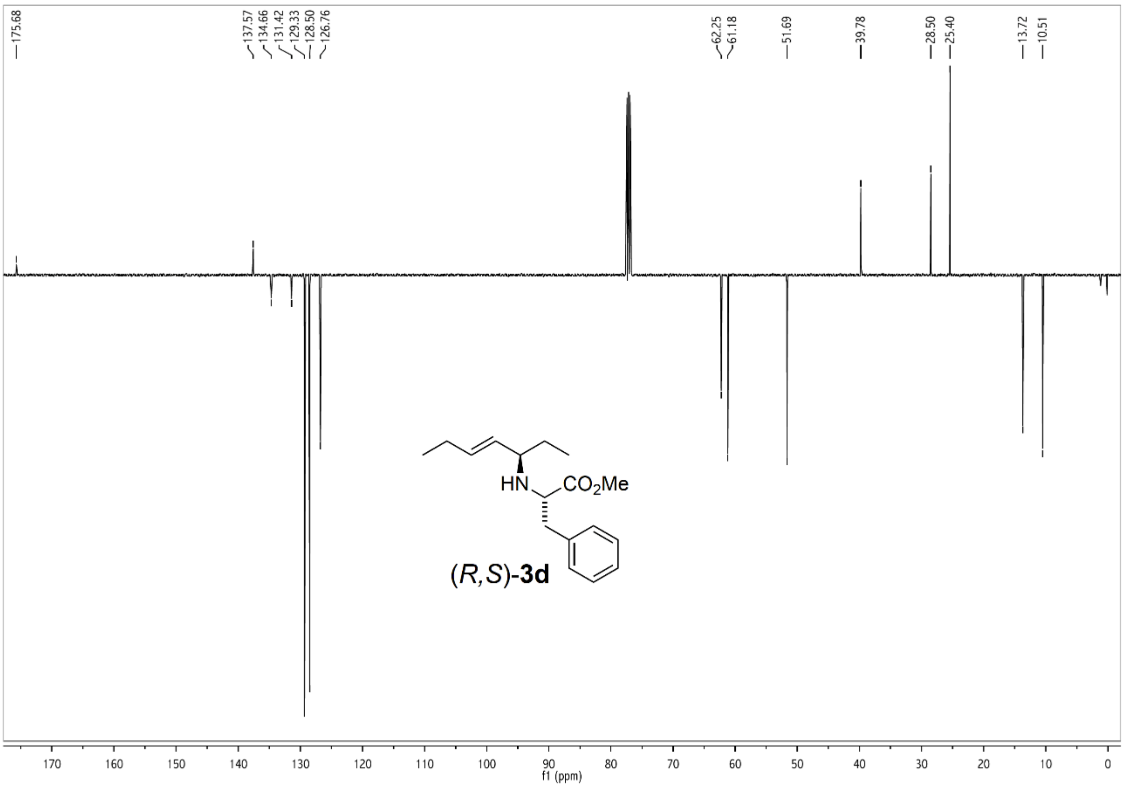

^{13}C-NMR (125 MHz) of (*R,S*)-**3e** in $CDCl_3$

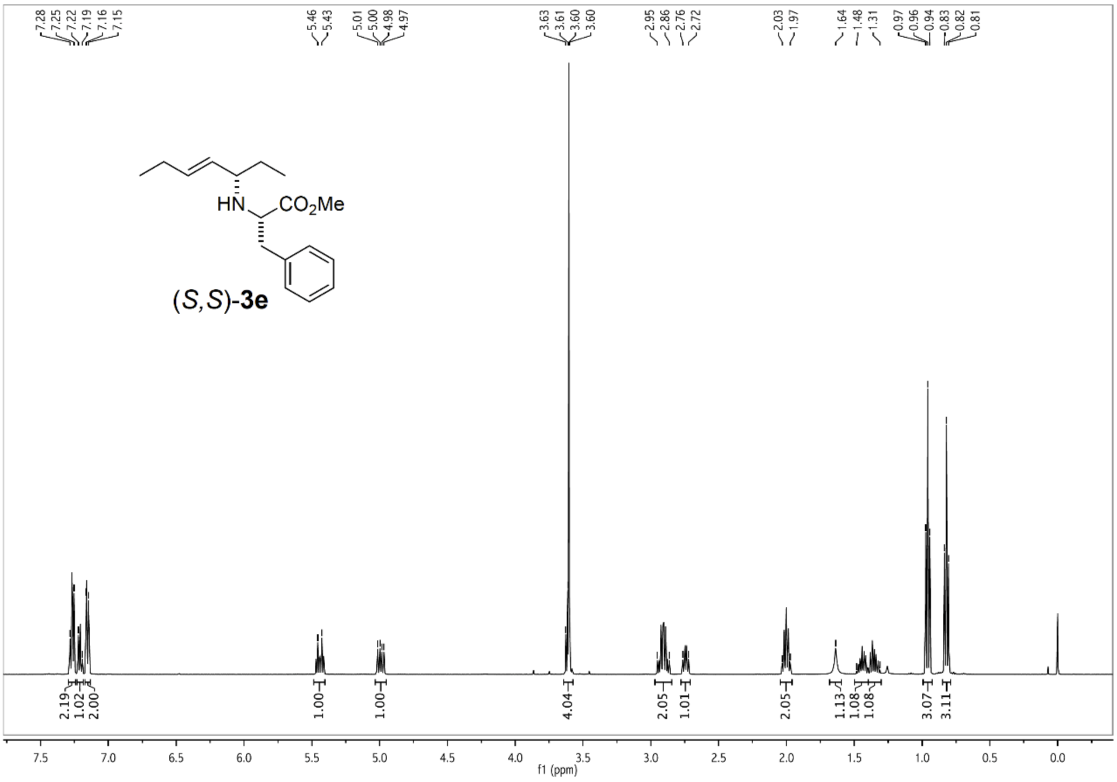

^{1}H NMR (500 MHz) of (*S,S*)-**3e** in $CDCl_3$

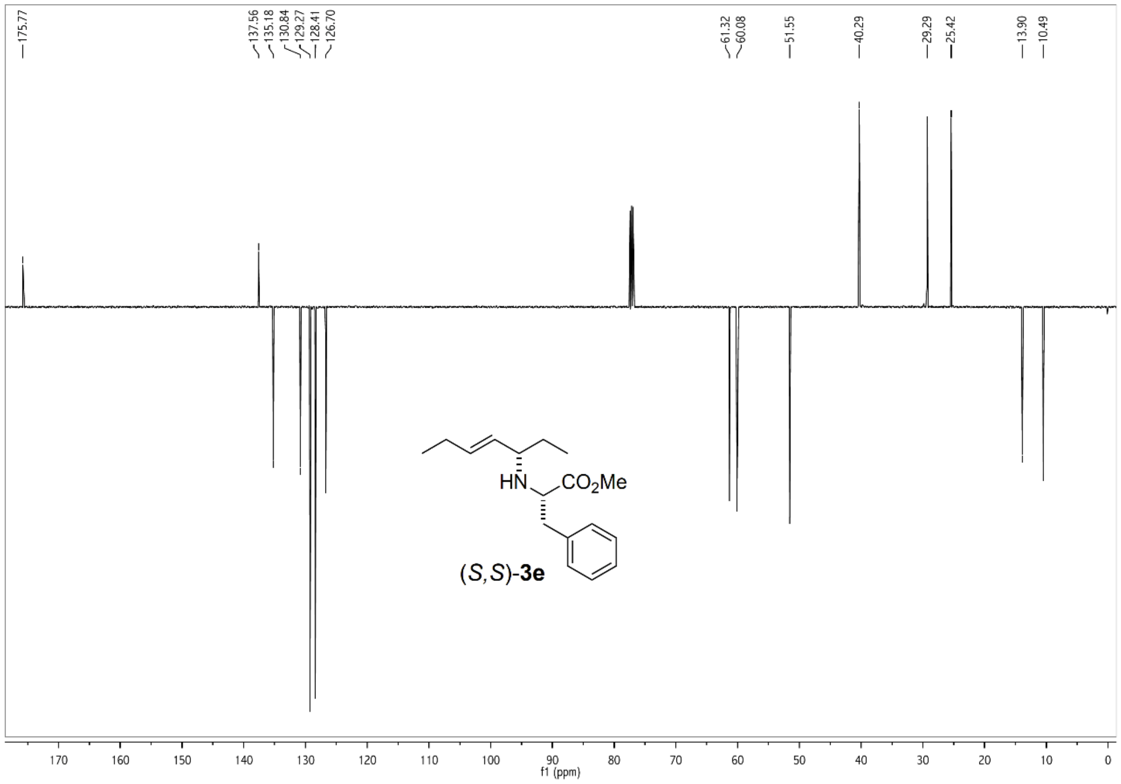

^{13}C-NMR (125 MHz) of (*S,S*)-**3e** in $CDCl_3$

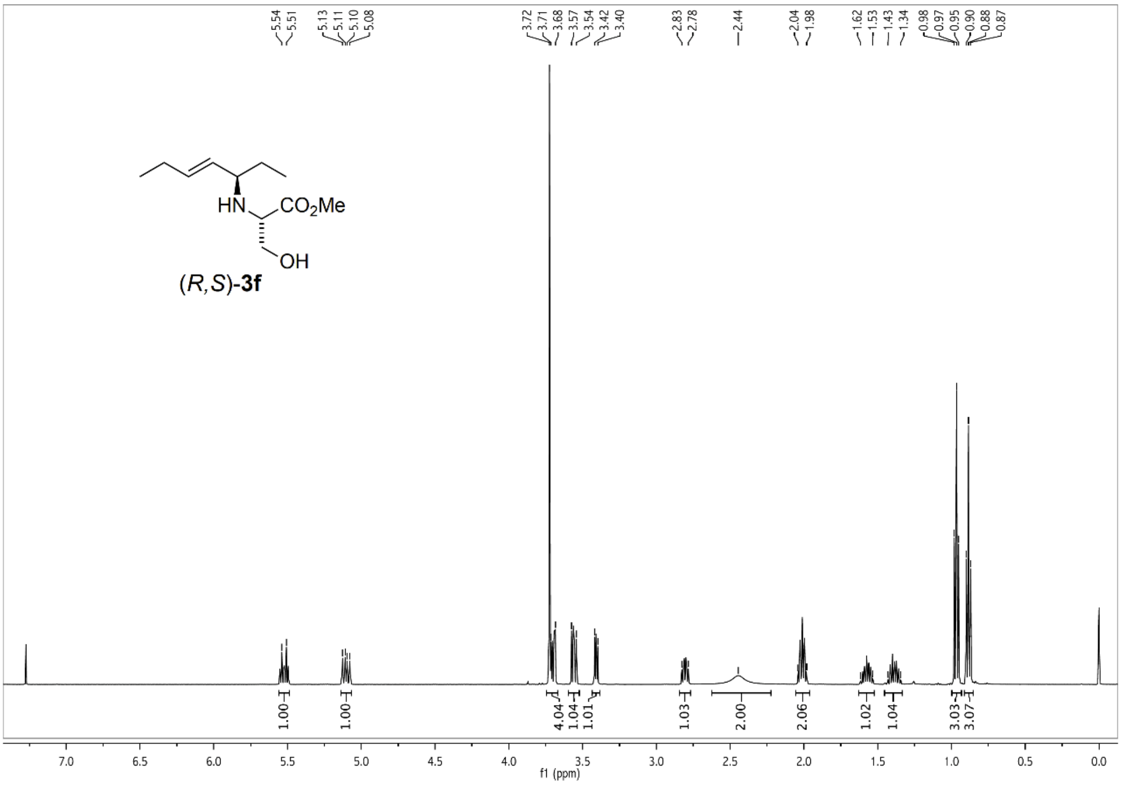

^{1}H NMR (500 MHz) of (*R,S*)-**3f** in $CDCl_3$

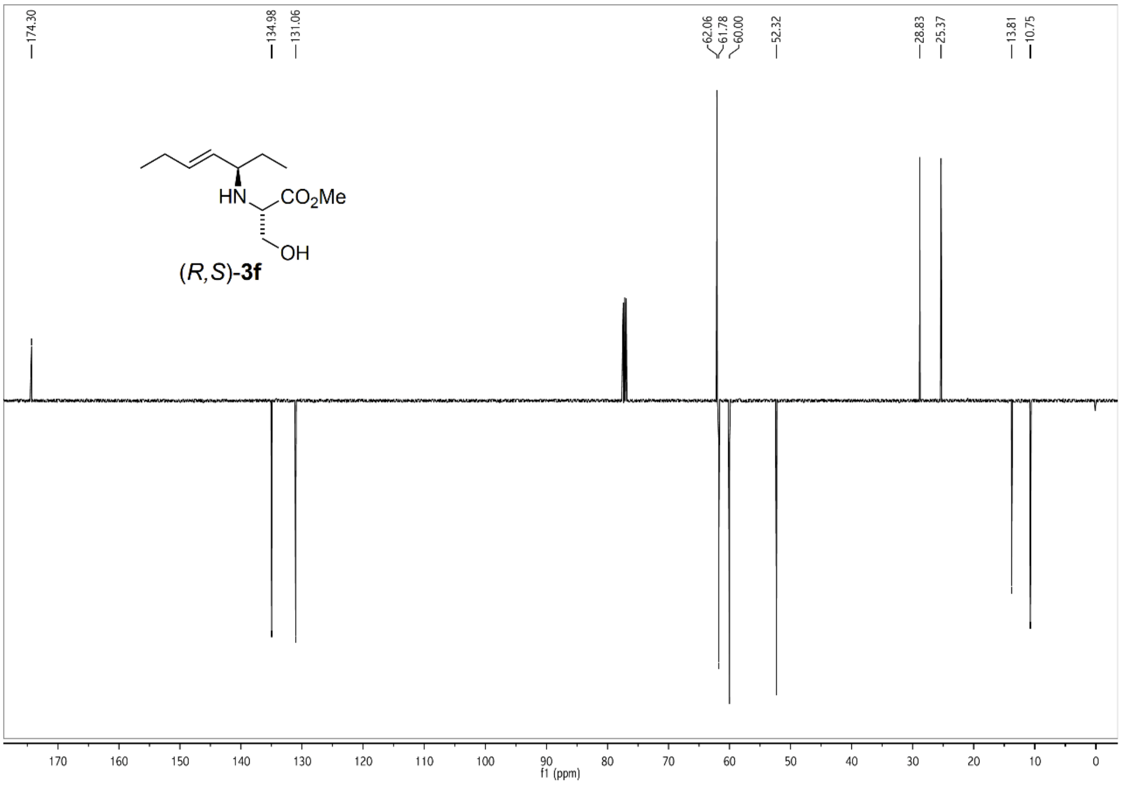

^{13}C-NMR (125 MHz) of (*R,S*)-**3f** in $CDCl_3$

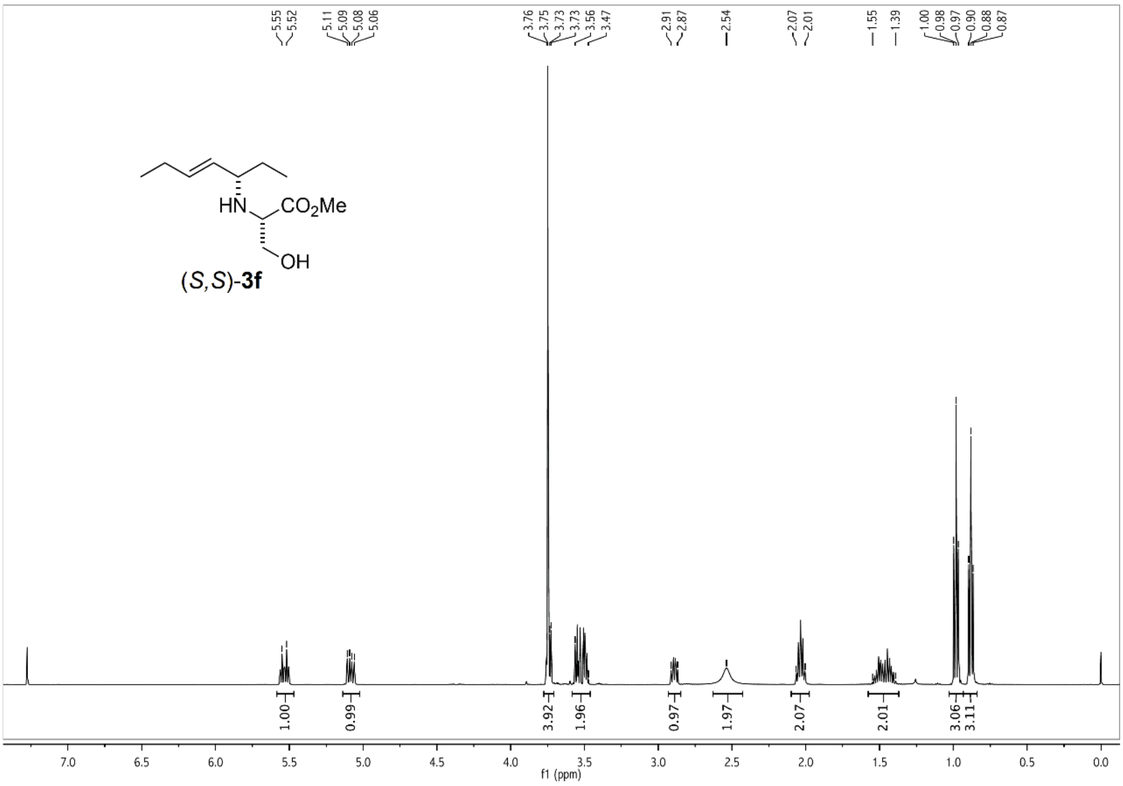

^{1}H NMR (500 MHz) of (*S,S*)-**3f** in $CDCl_3$

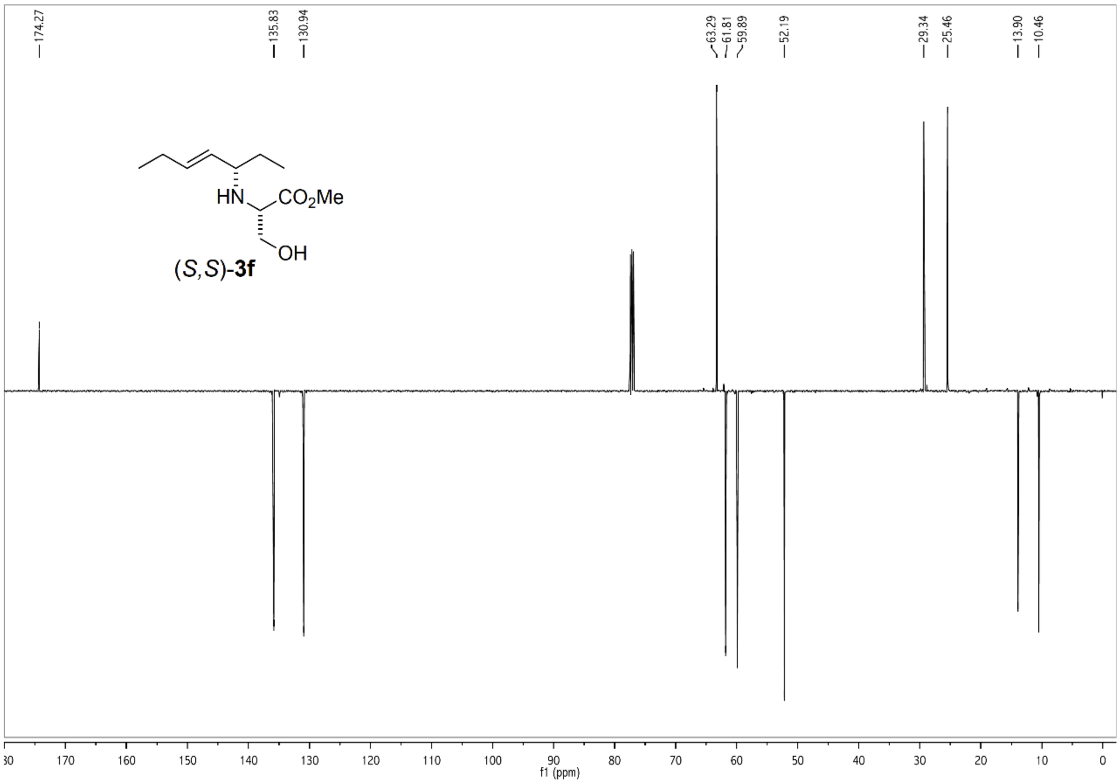

^{13}C-NMR (125 MHz) of *(S,S)*-**3f** in $CDCl_3$

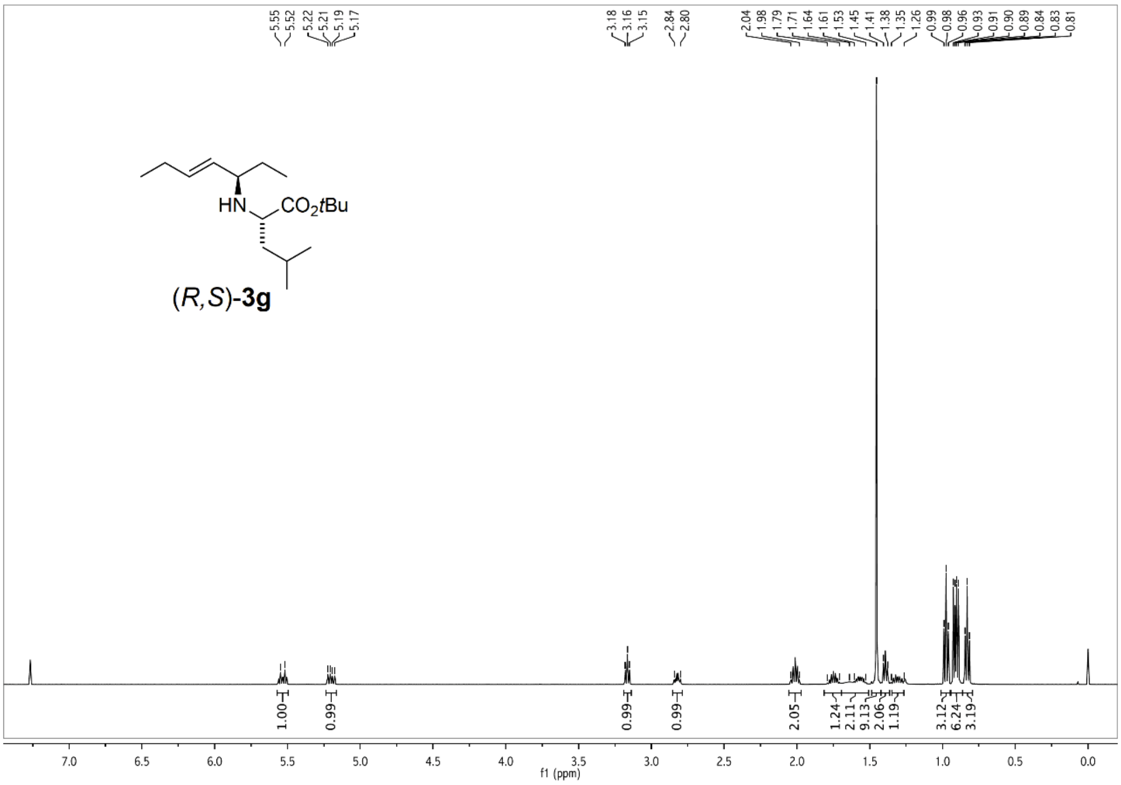

^{1}H NMR (500 MHz) of (*R,S*)-**3g** in $CDCl_3$

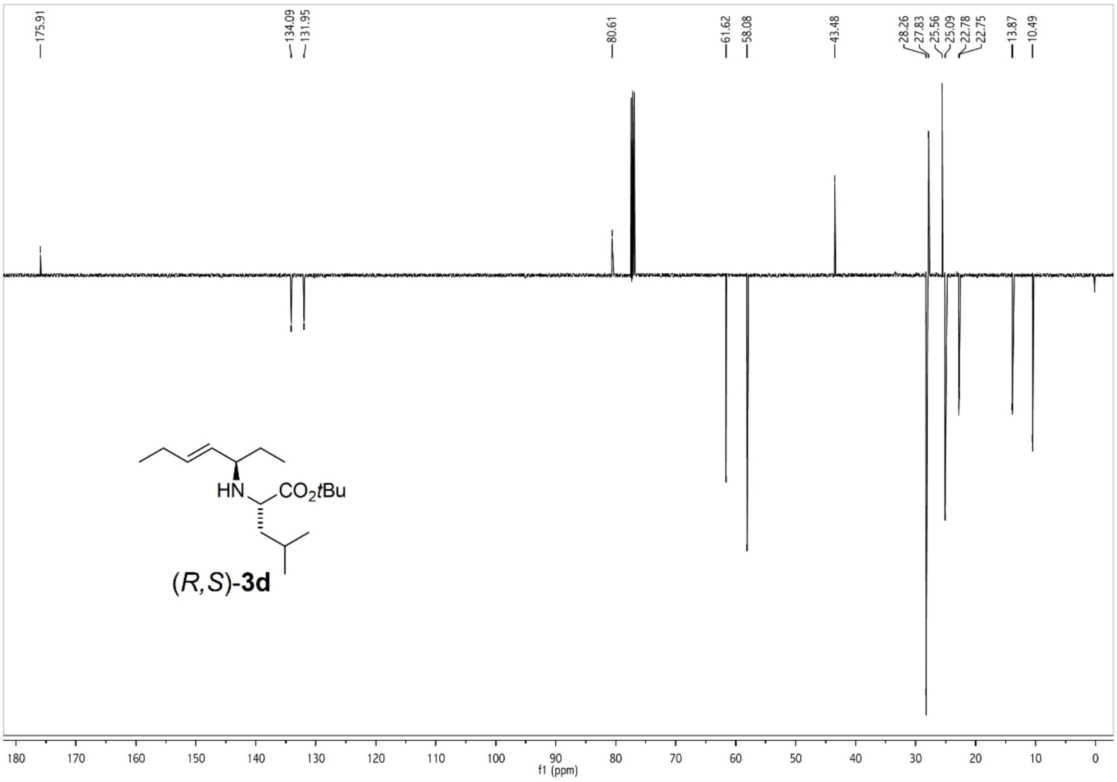

^{13}C-NMR (125 MHz) of (*R,S*)-**3g** in $CDCl_3$

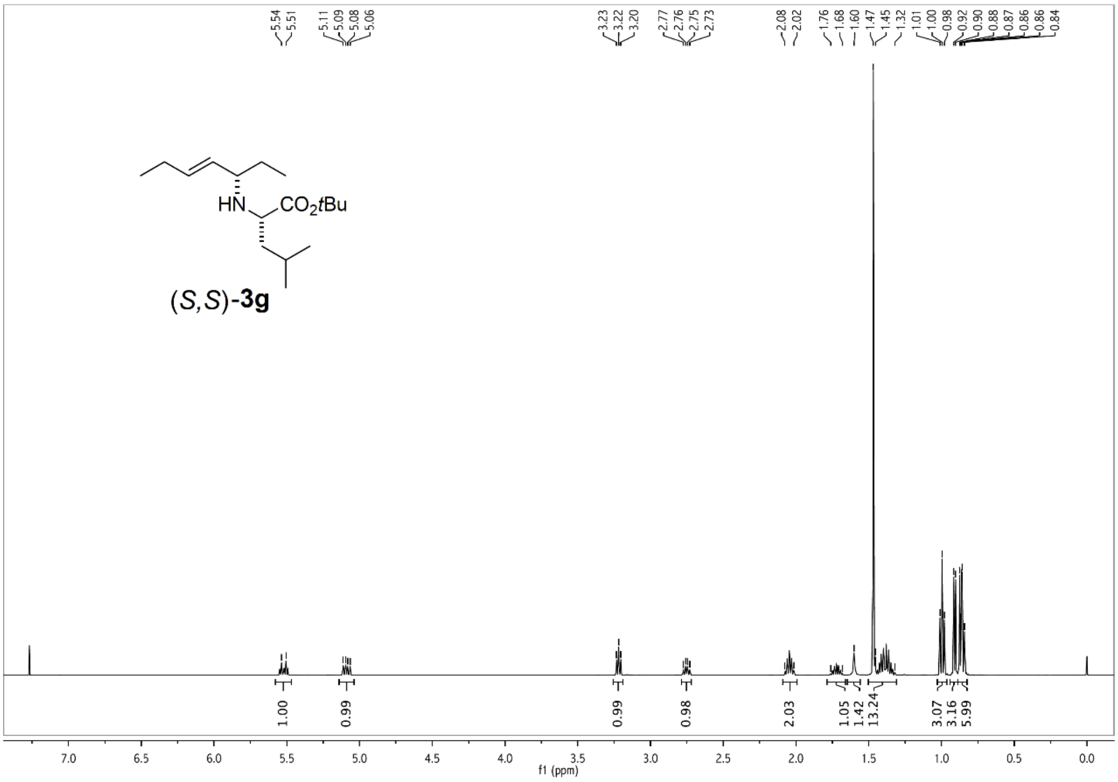

^{1}H NMR (500 MHz) of (*S,S*)-**3g** in $CDCl_3$

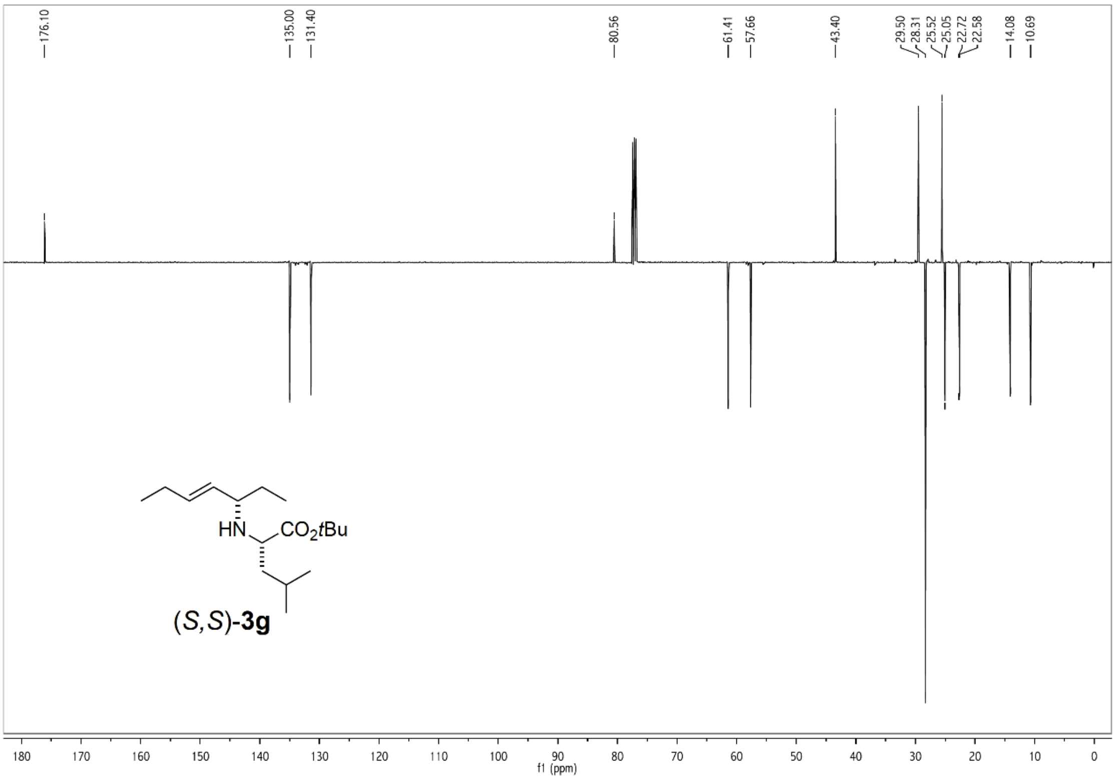

^{13}C-NMR (125 MHz) of (*S,S*)-**3g** in $CDCl_3$

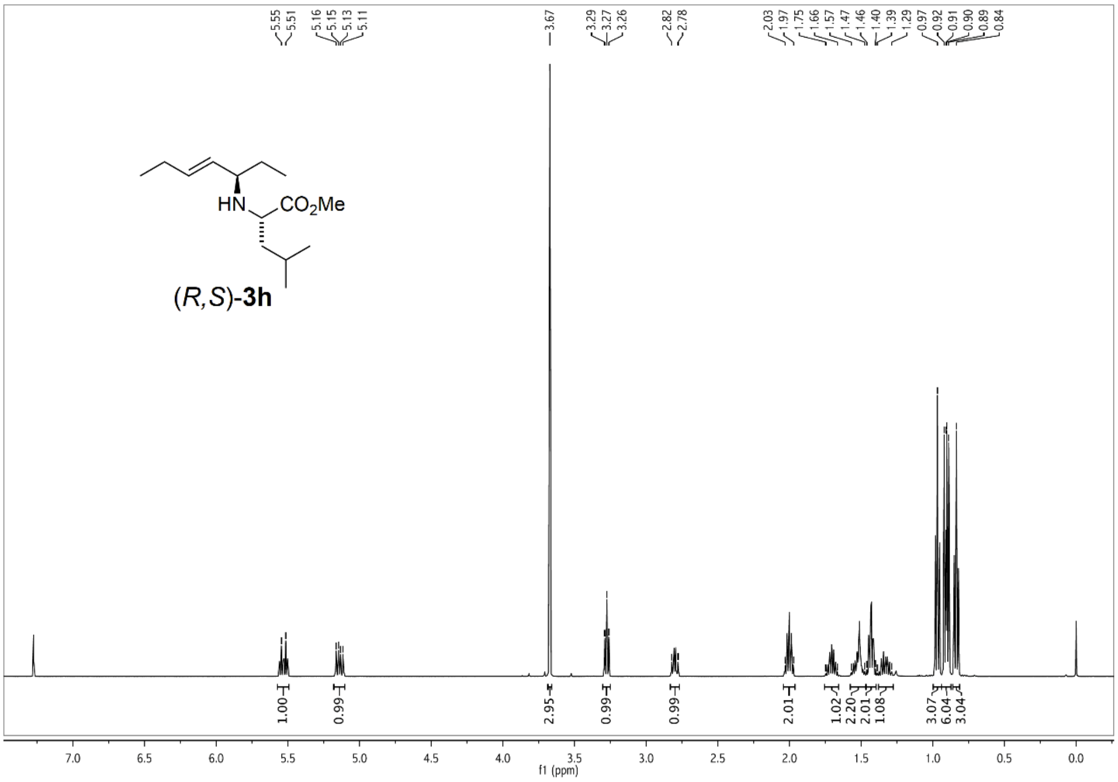

^{1}H NMR (500 MHz) of (*R*,*S*)-**3h** in $CDCl_3$

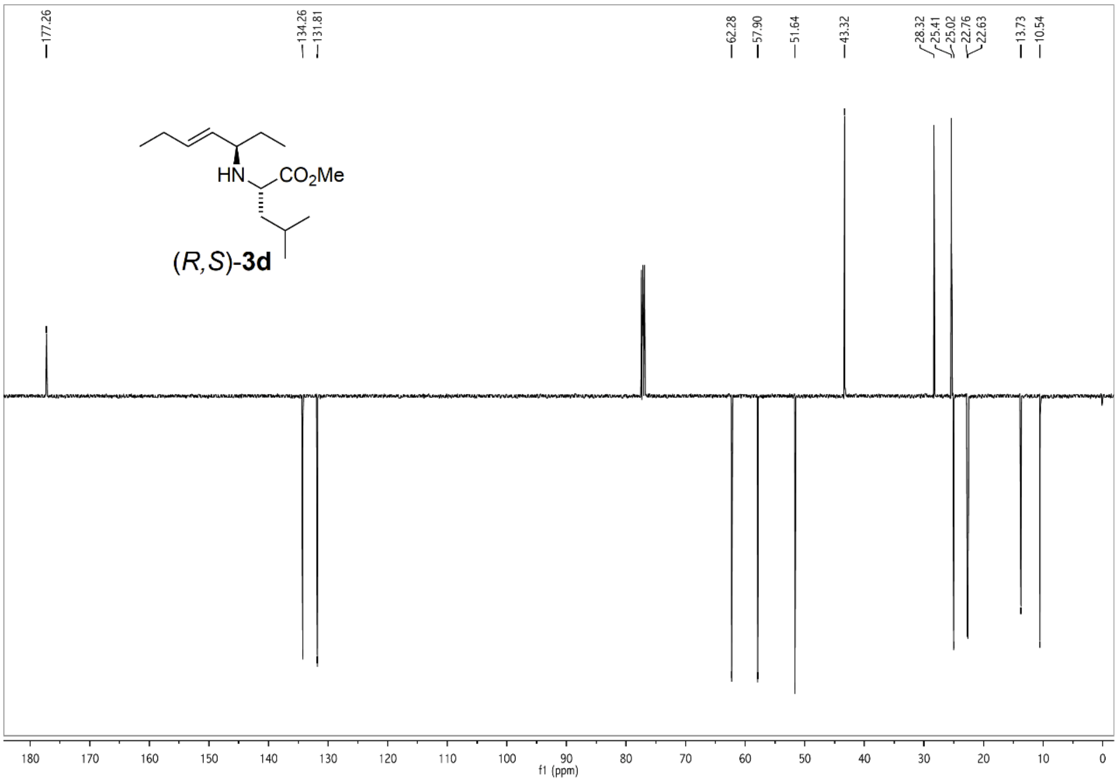

^{13}C-NMR (125 MHz) of (*R*,*S*)-**3h** in $CDCl_3$

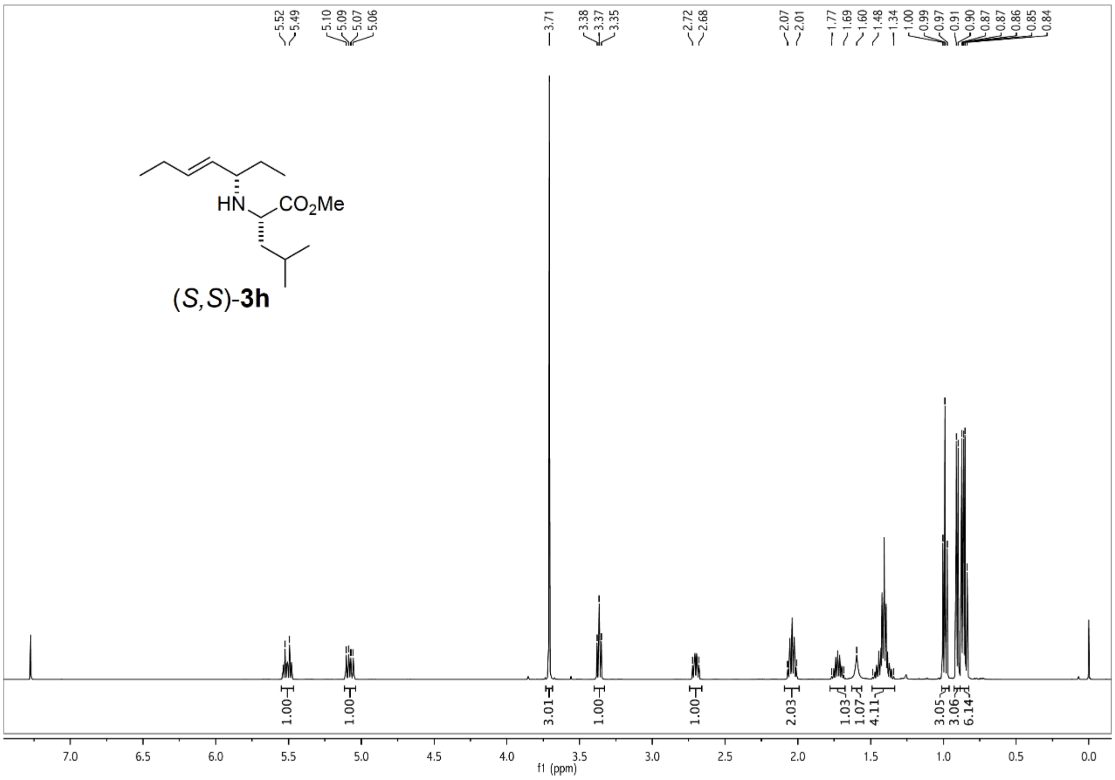

^{1}H NMR (500 MHz) of (*S,S*)-**3h** in $CDCl_3$

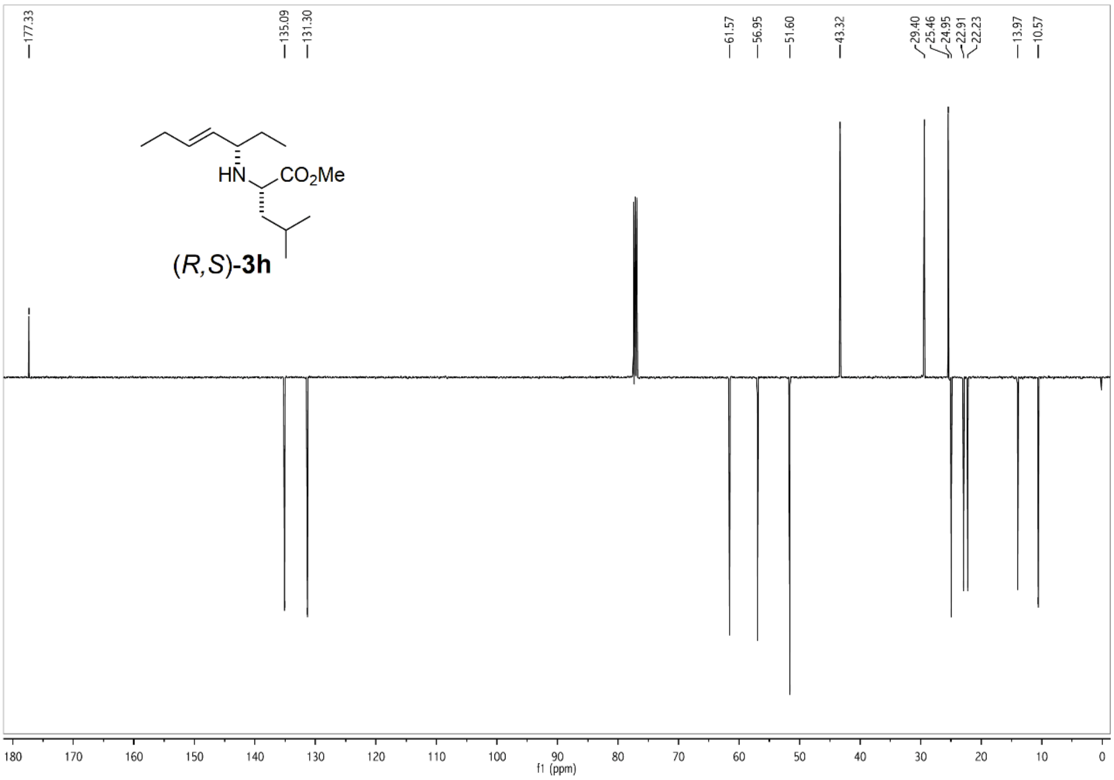

^{13}C-NMR (125 MHz) of (*S,S*)-**3h** in $CDCl_3$

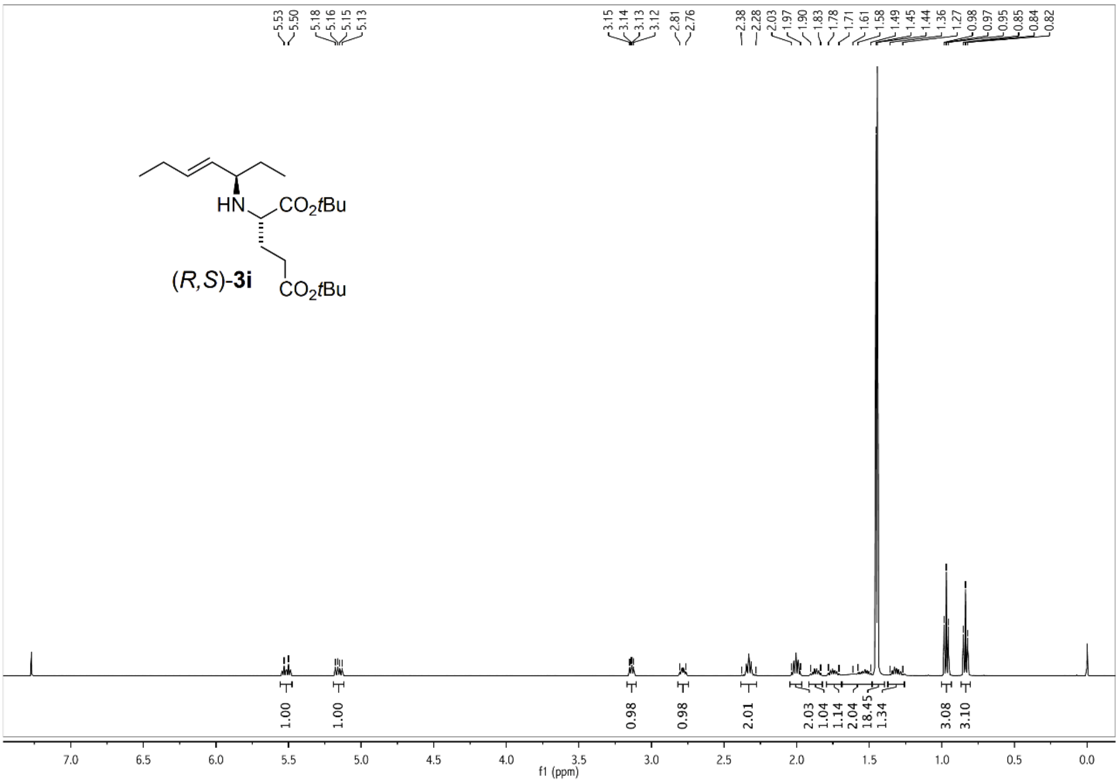

^{1}H NMR (500 MHz) of (*R,S*)-**3i** in $CDCl_3$

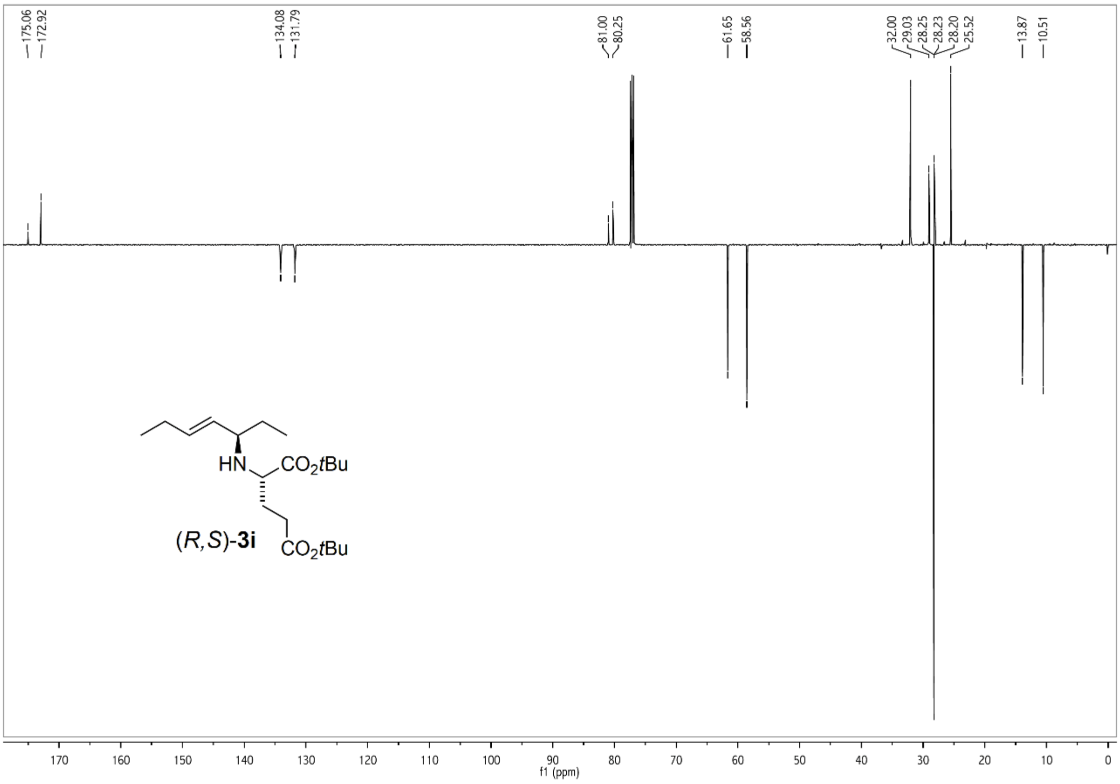

^{13}C-NMR (125 MHz) of (*R,S*)-**3i** in $CDCl_3$

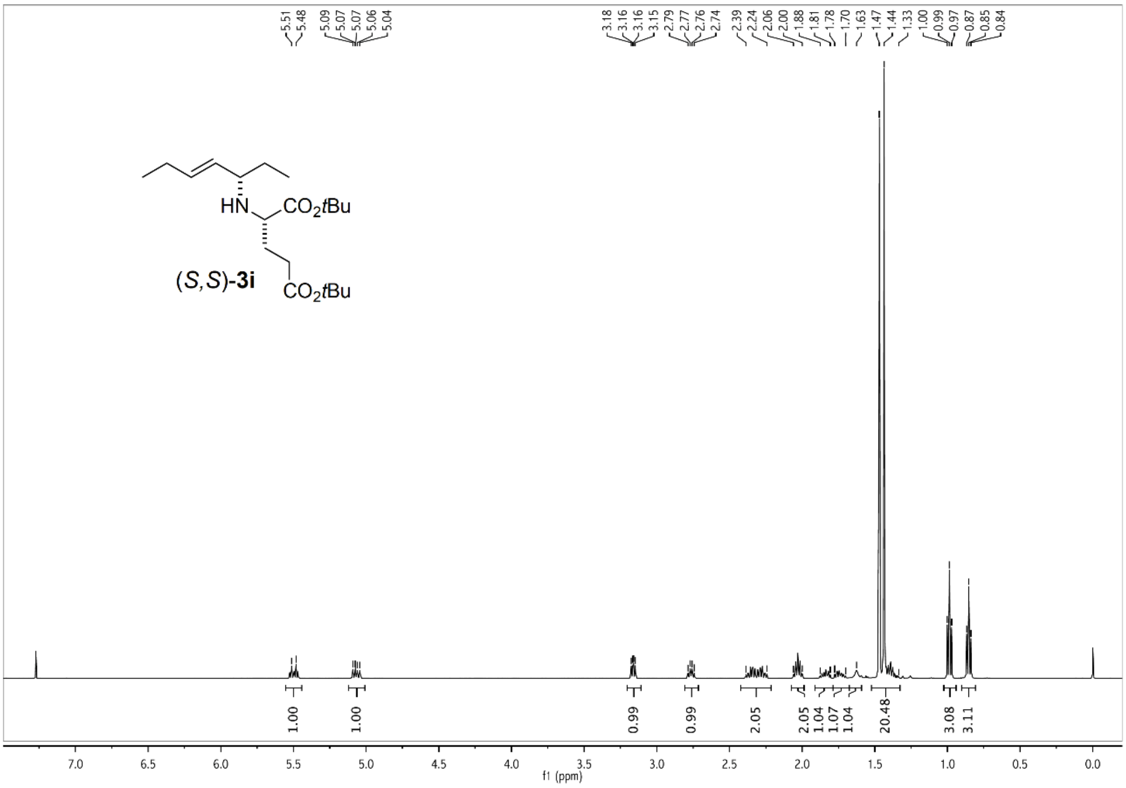

^{1}H NMR (500 MHz) of (*S*,*S*)-**3i** in $CDCl_3$

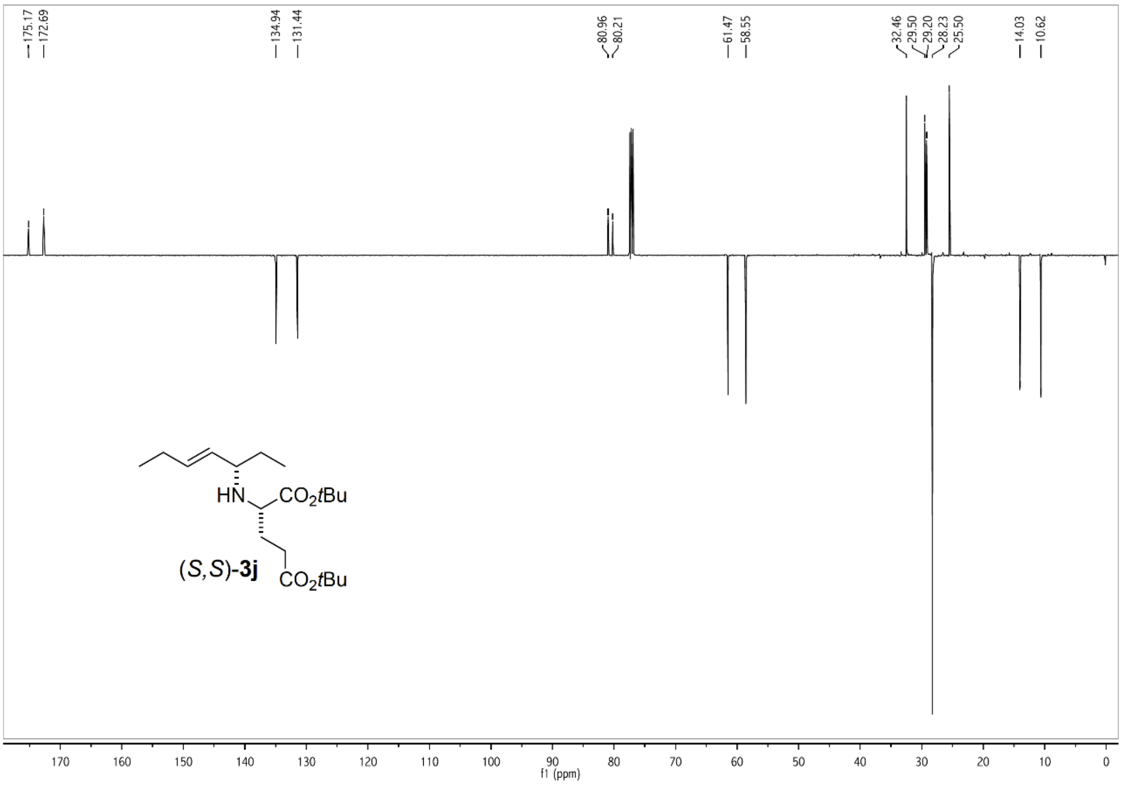

^{13}C-NMR (125 MHz) of (*S*,*S*)-**3i** in $CDCl_3$

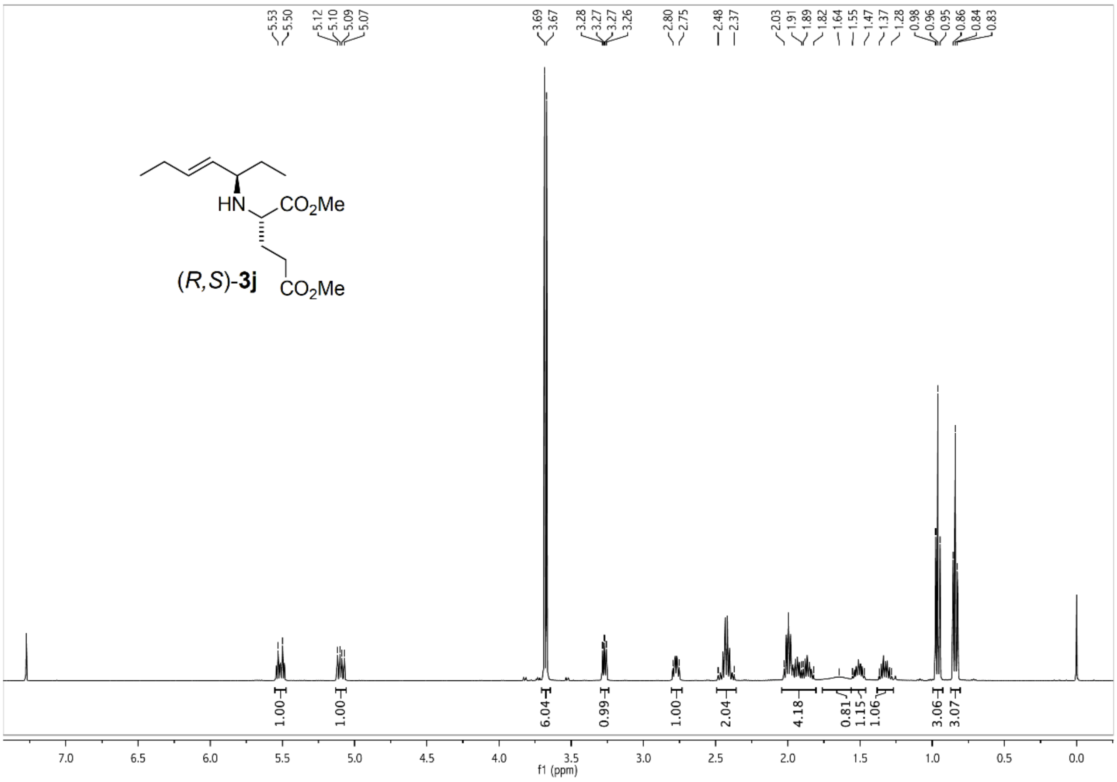

^{1}H NMR (500 MHz) of (*R*,*S*)-**3j** in $CDCl_3$

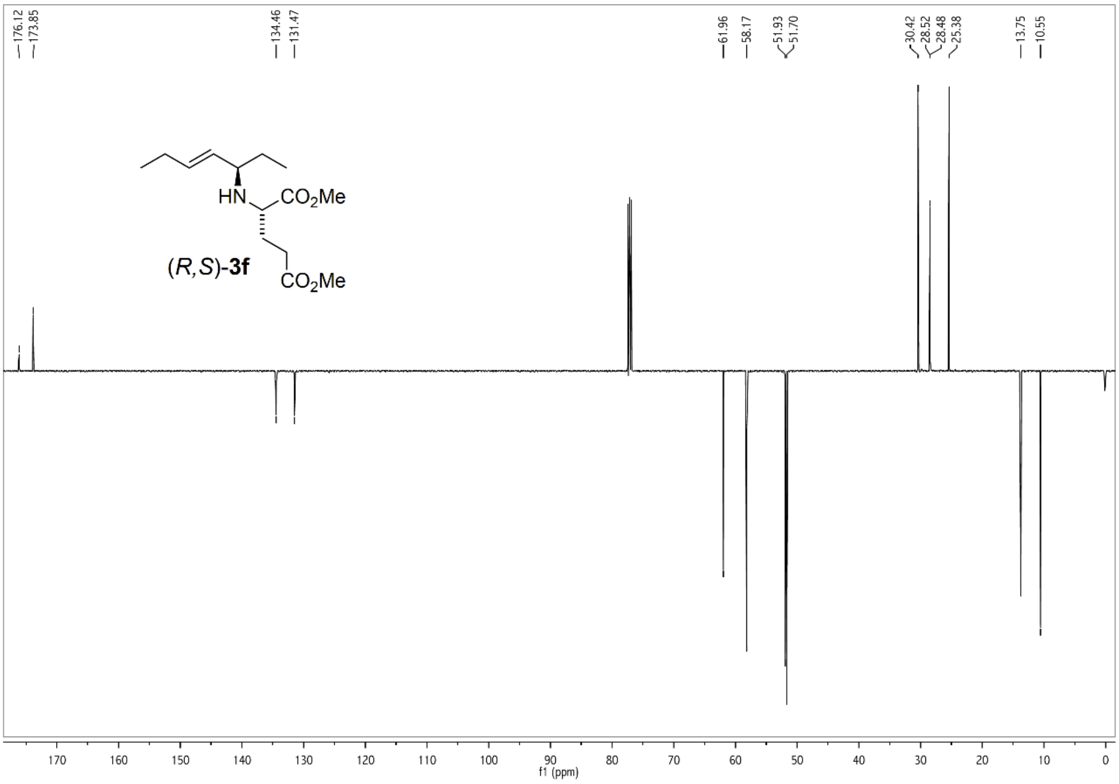

^{13}C-NMR (125 MHz) of (*R*,*S*)-**3j** in $CDCl_3$

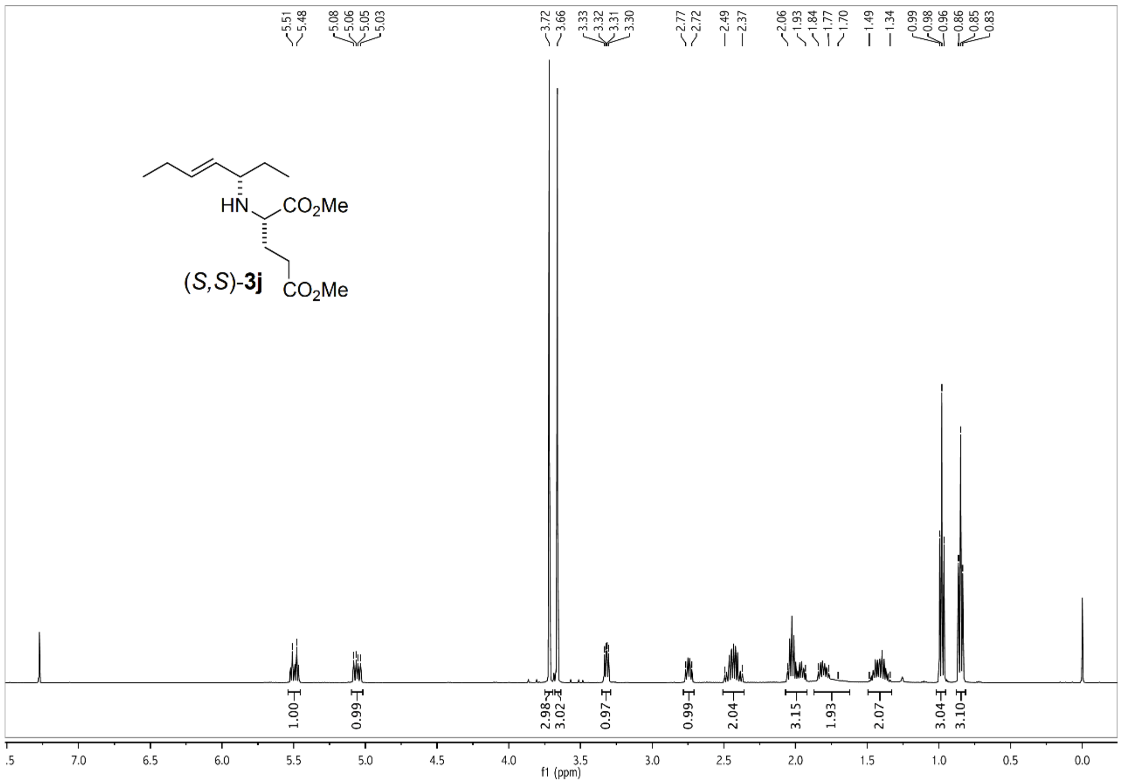

^{1}H NMR (500 MHz) of (*S,S*)-**3j** in $CDCl_3$

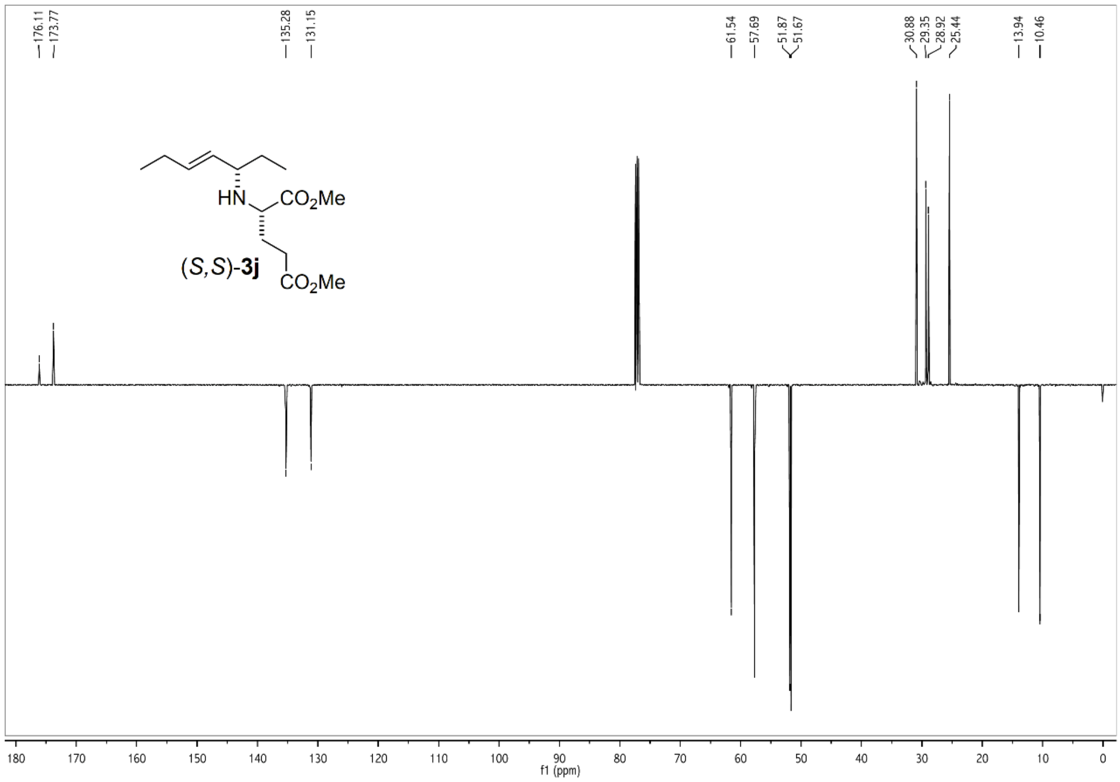

^{13}C-NMR (125 MHz) of (*S,S*)-**3j** in $CDCl_3$

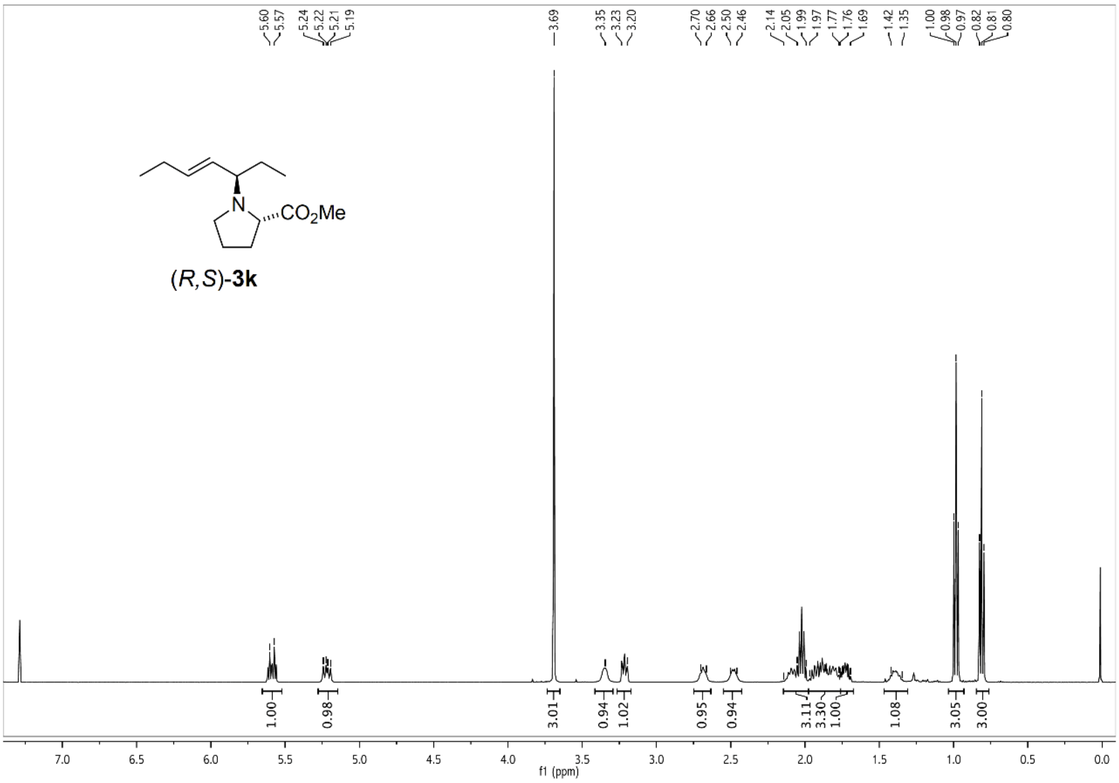

^{1}H NMR (500 MHz) of (*R*,*S*)-**3k** in $CDCl_3$

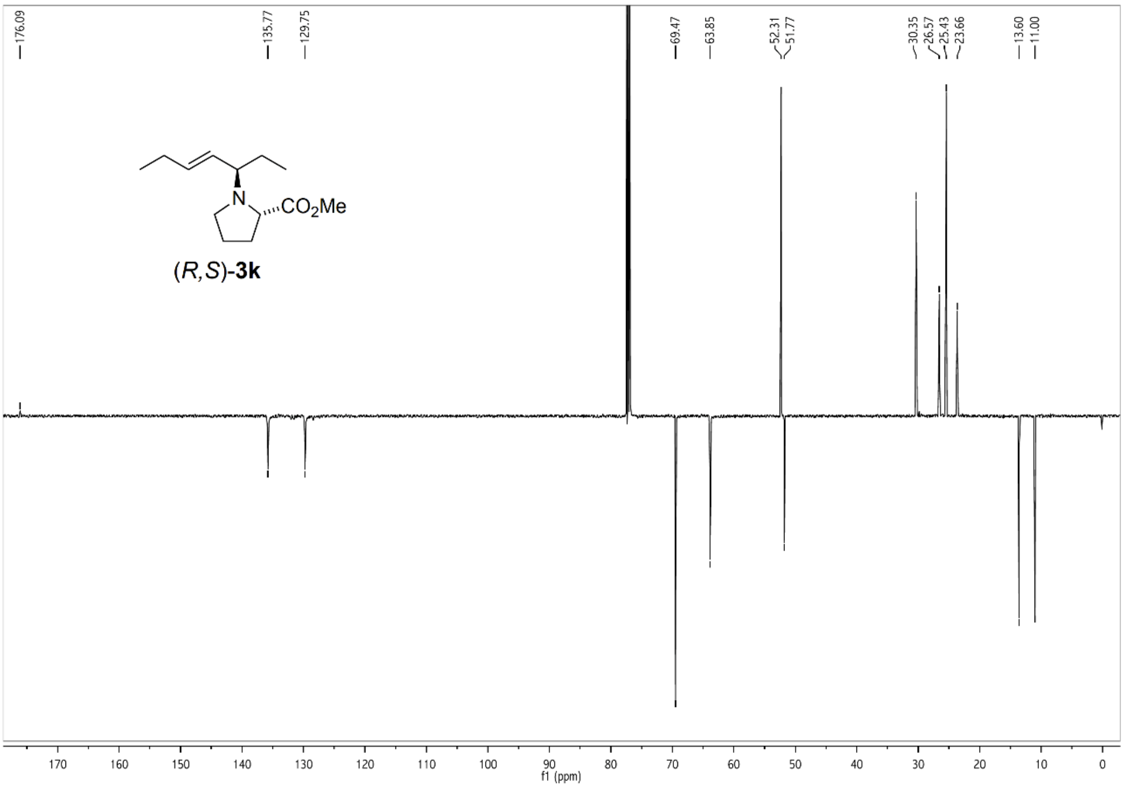

^{13}C-NMR (125 MHz) of (*R*,*S*)-**3k** in $CDCl_3$

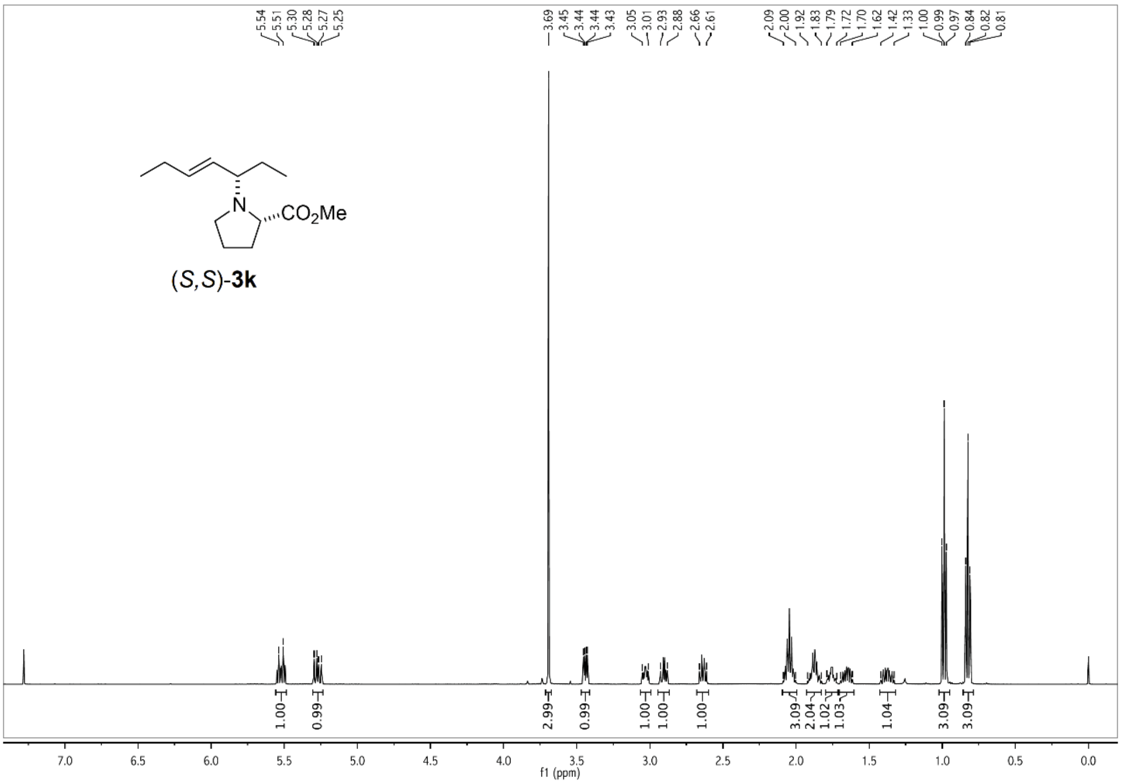

^{1}H NMR (500 MHz) of (*S,S*)-**3k** in $CDCl_3$

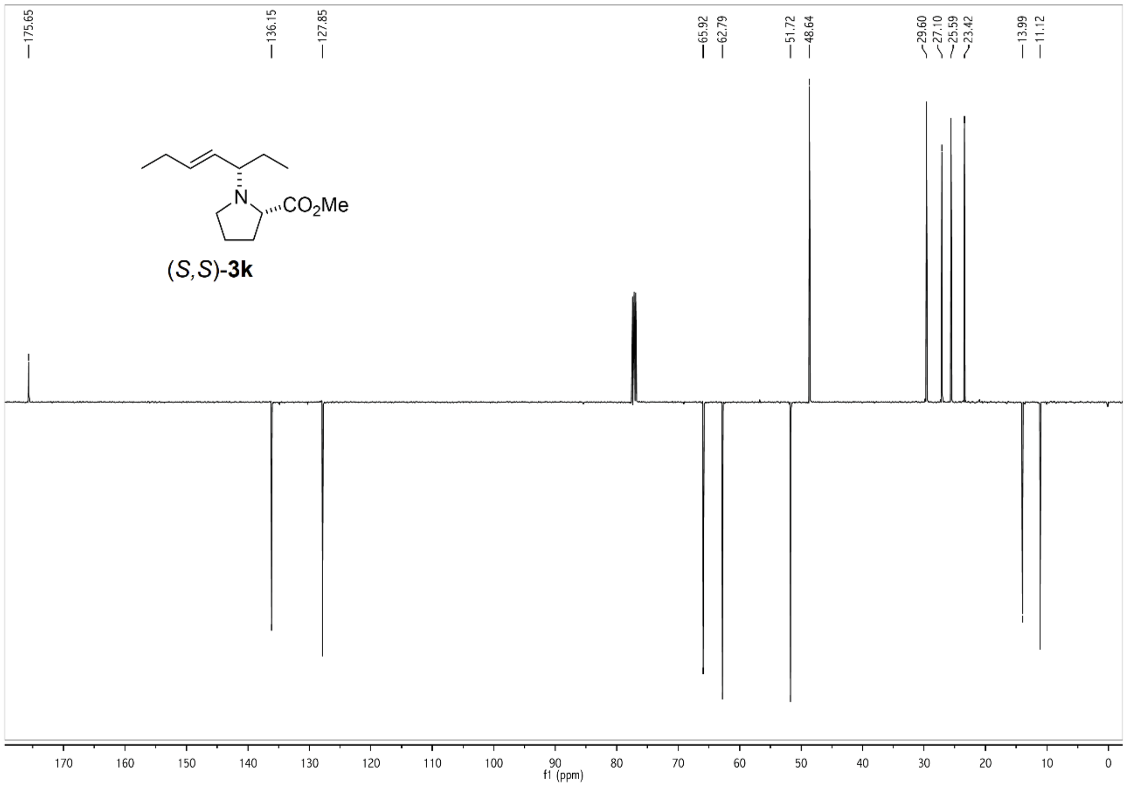

^{13}C-NMR (125 MHz) of (*S,S*)-**3k** in $CDCl_3$

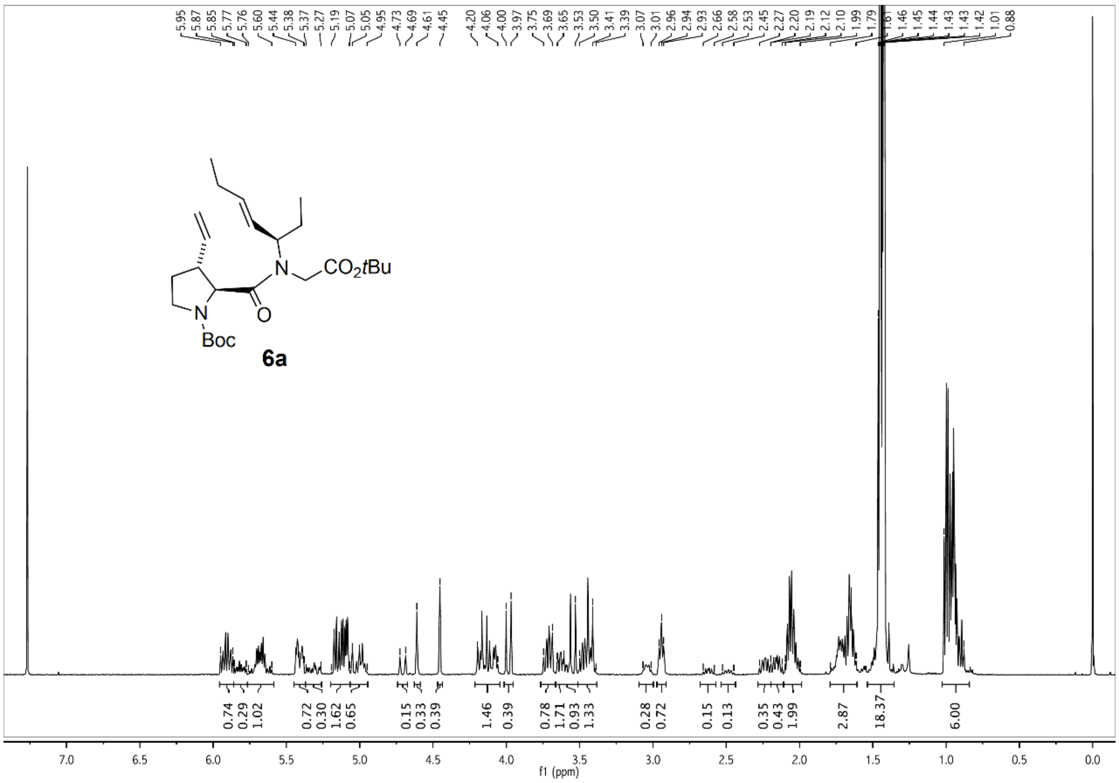

^{1}H NMR (500 MHz) of **6a** in $CDCl_3$

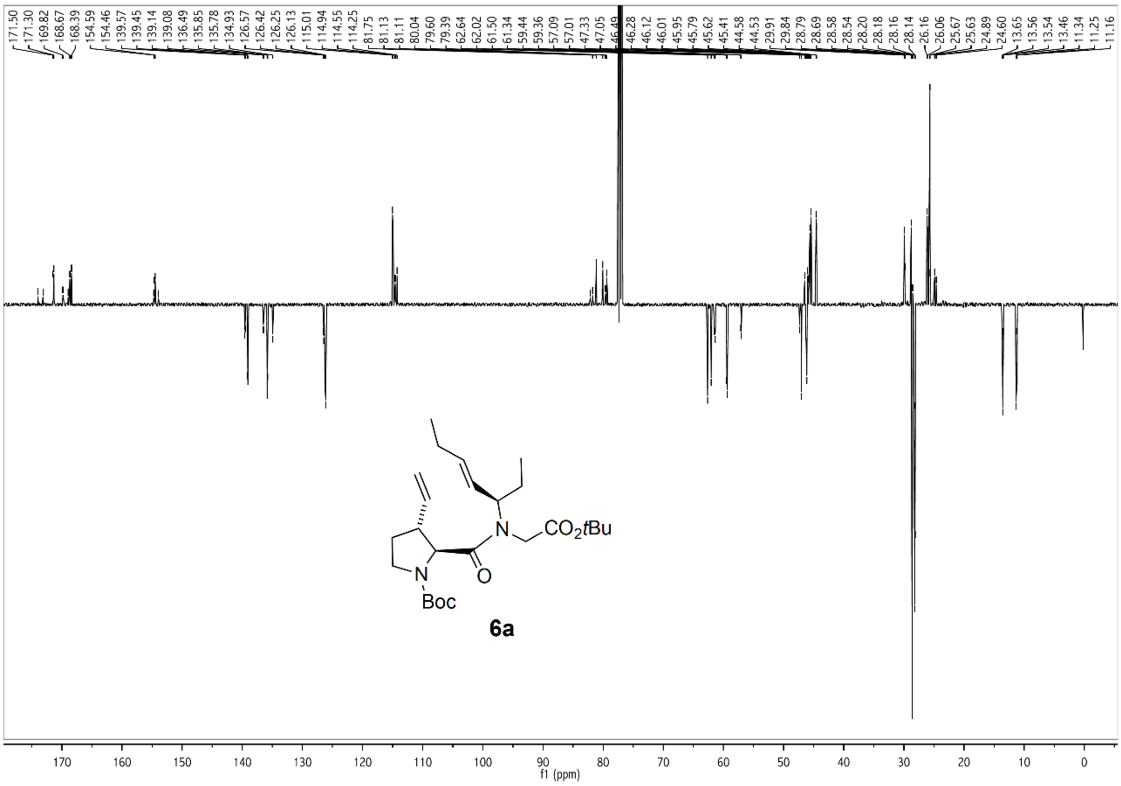

^{13}C-NMR (125 MHz) of **6a** in $CDCl_3$

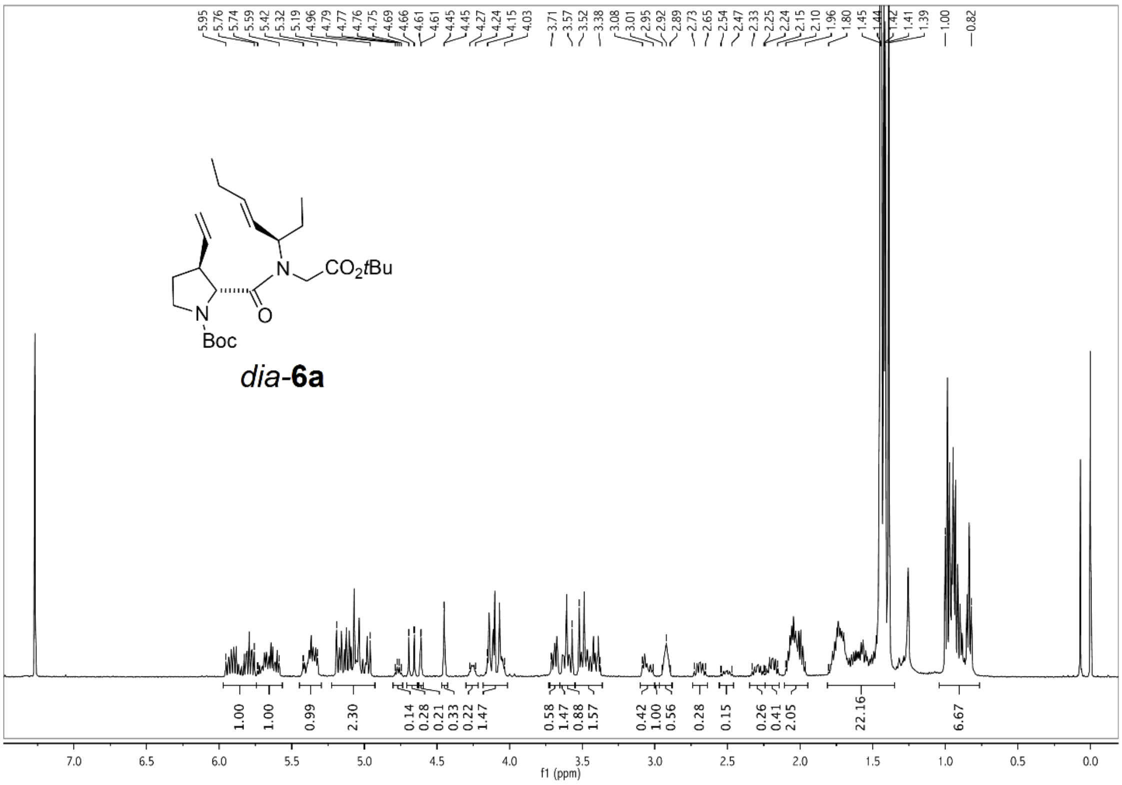

^{1}H NMR (500 MHz) of *dia*-**6a** in $CDCl_3$

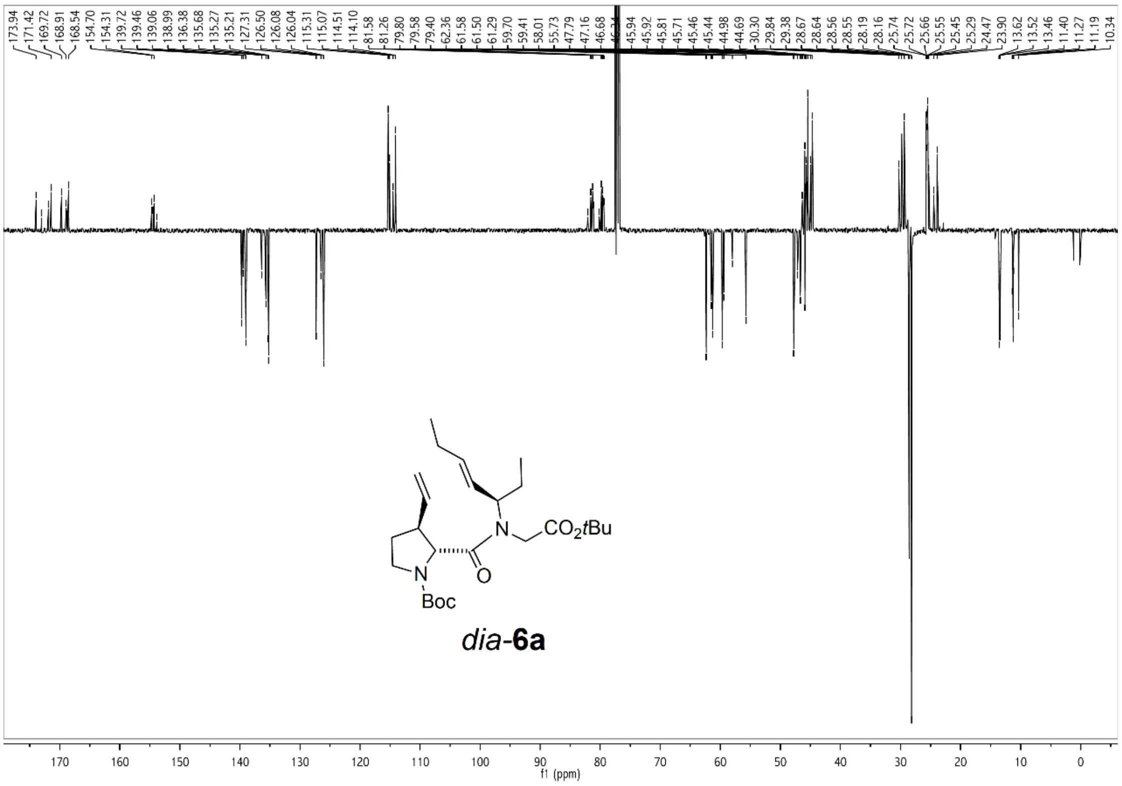

^{13}C-NMR (125 MHz) of *dia*-**6a** in $CDCl_3$

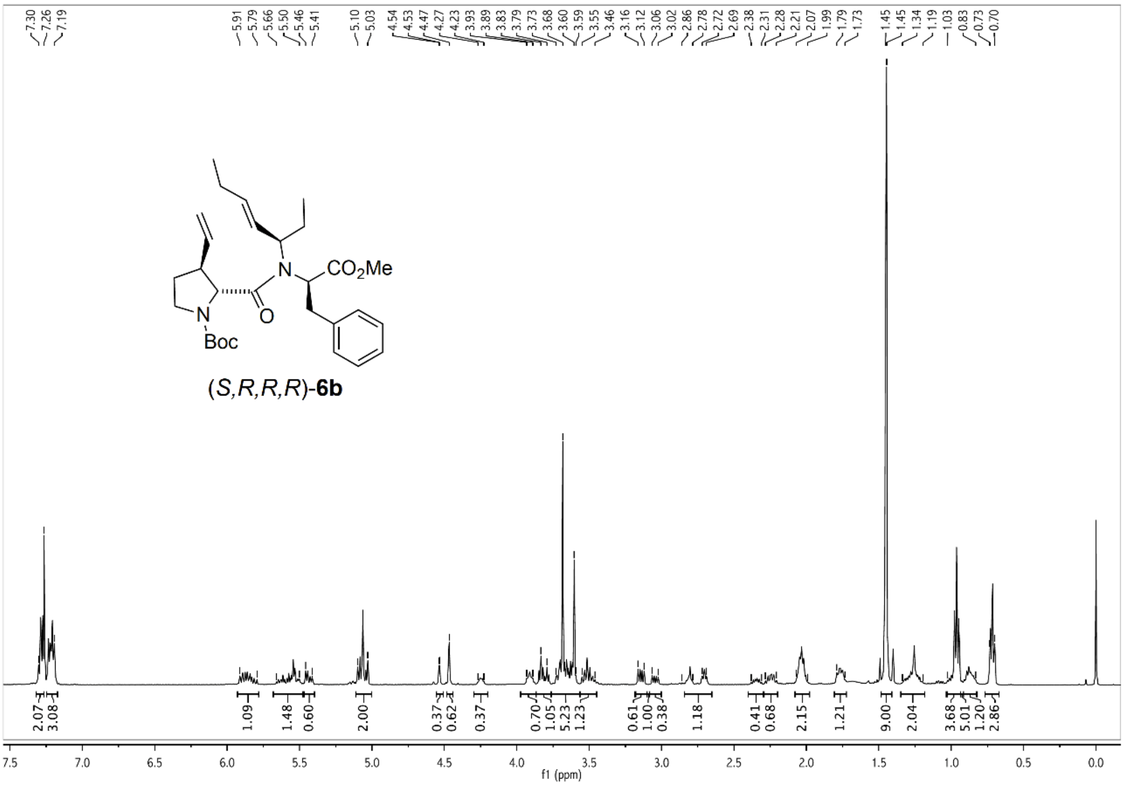

^{1}H NMR (500 MHz) of *(S,R,R,R)*-**6b** in $CDCl_3$

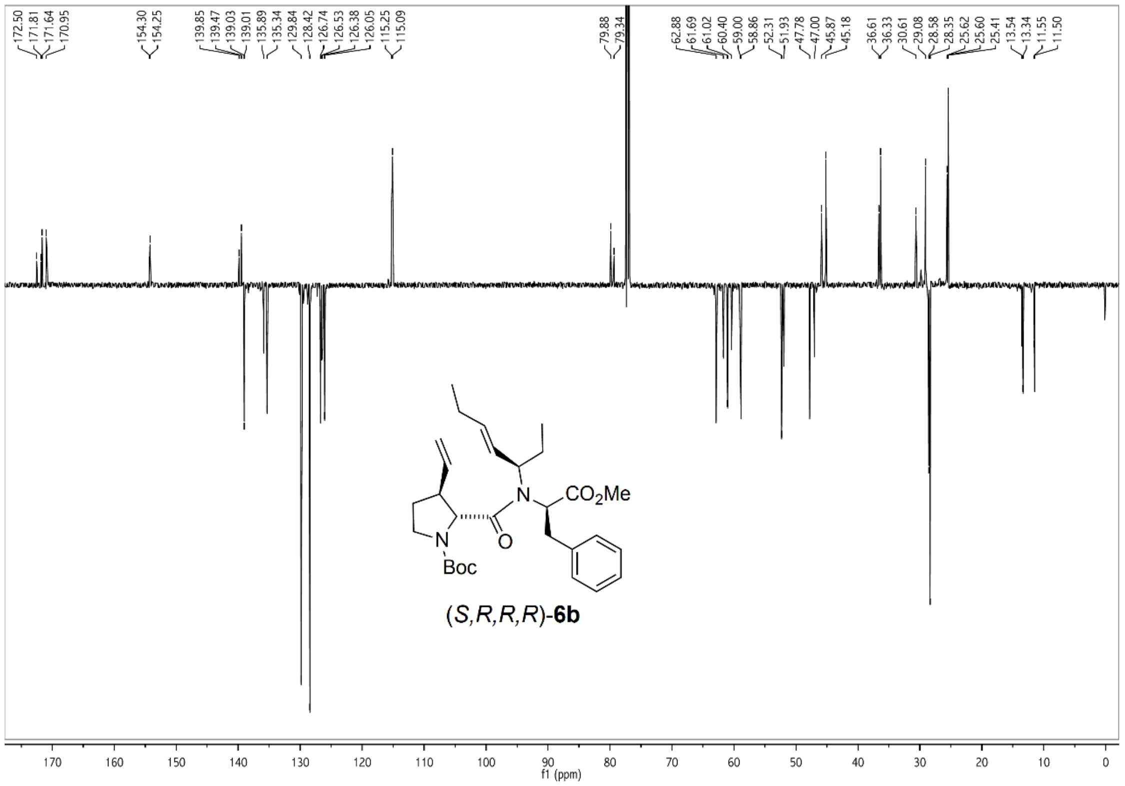

^{13}C-NMR (125 MHz) of *(S,R,R,R)*-**6b** in $CDCl_3$

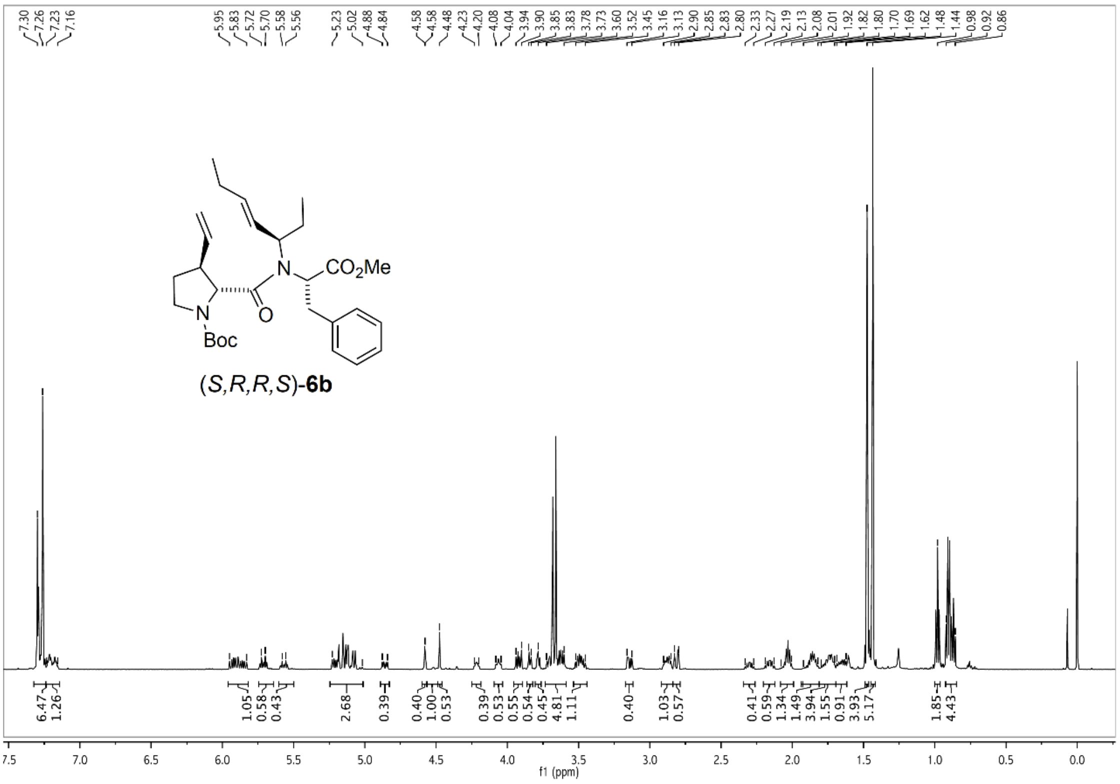

^{1}H NMR (500 MHz) of *(S,R,R,S)*-**6b** in $CDCl_3$

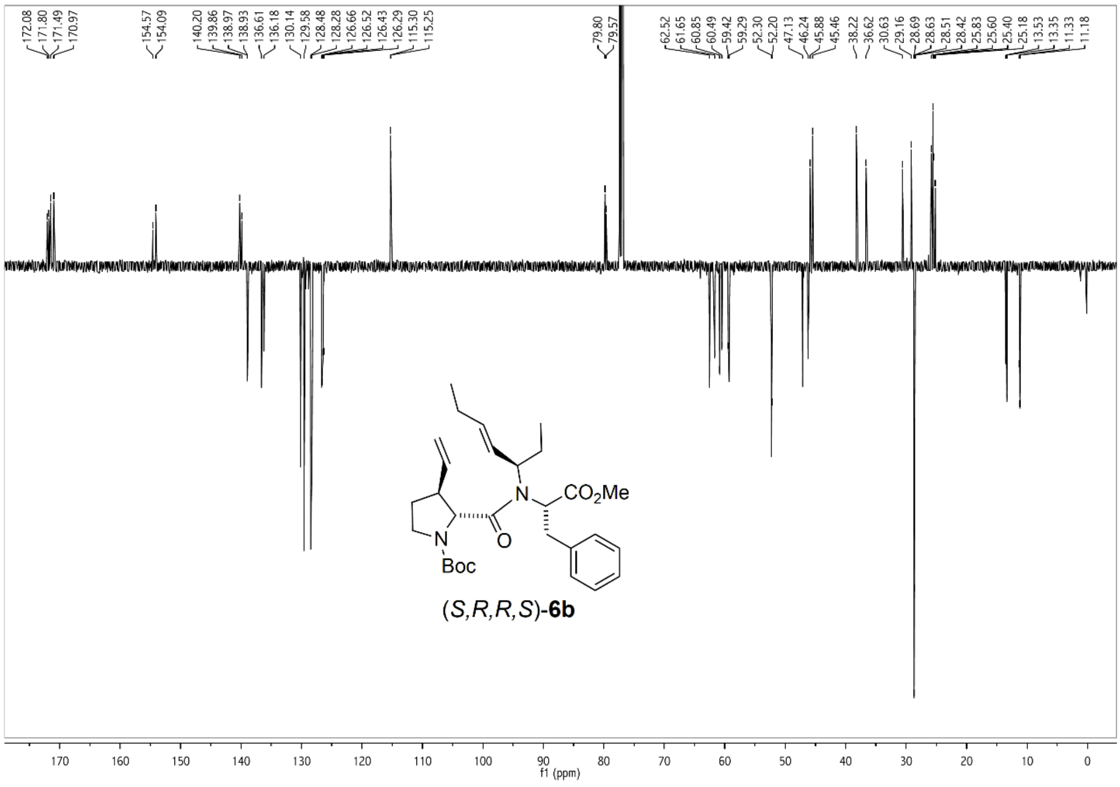

^{13}C-NMR (125 MHz) of *(S,R,R,S)*-**6b** in $CDCl_3$

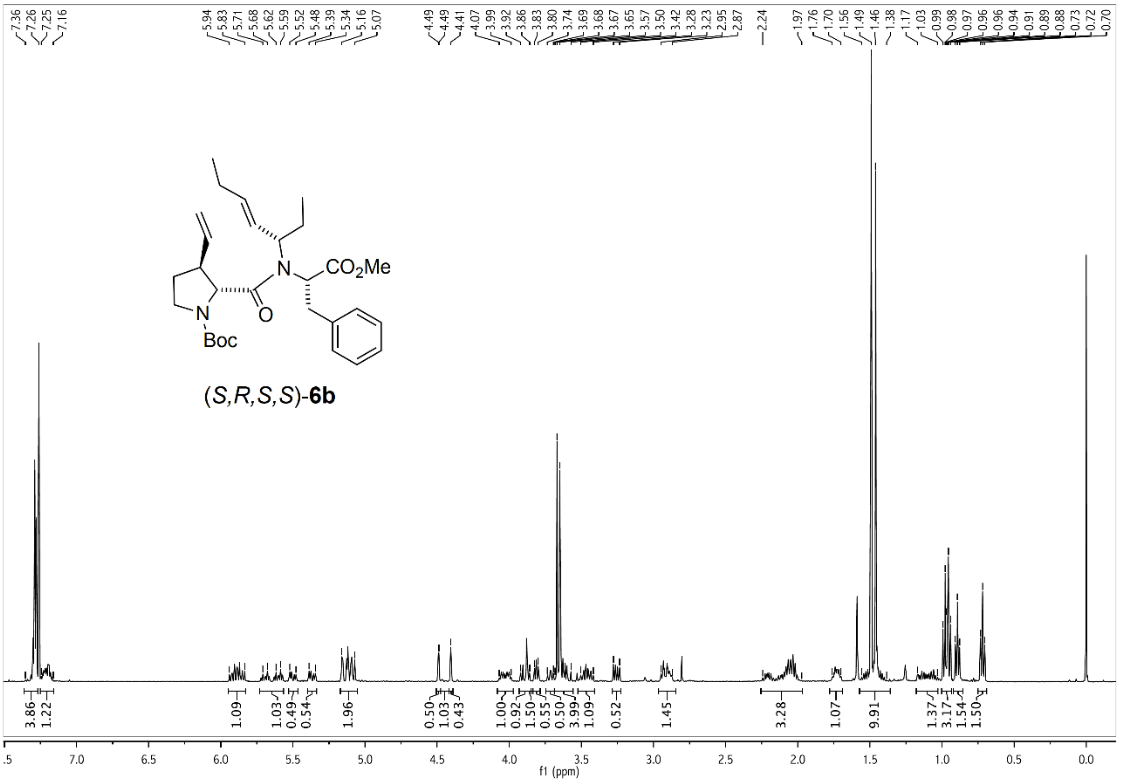

^{1}H NMR (500 MHz) of *(S,R,S,S)*-**6b** in $CDCl_3$

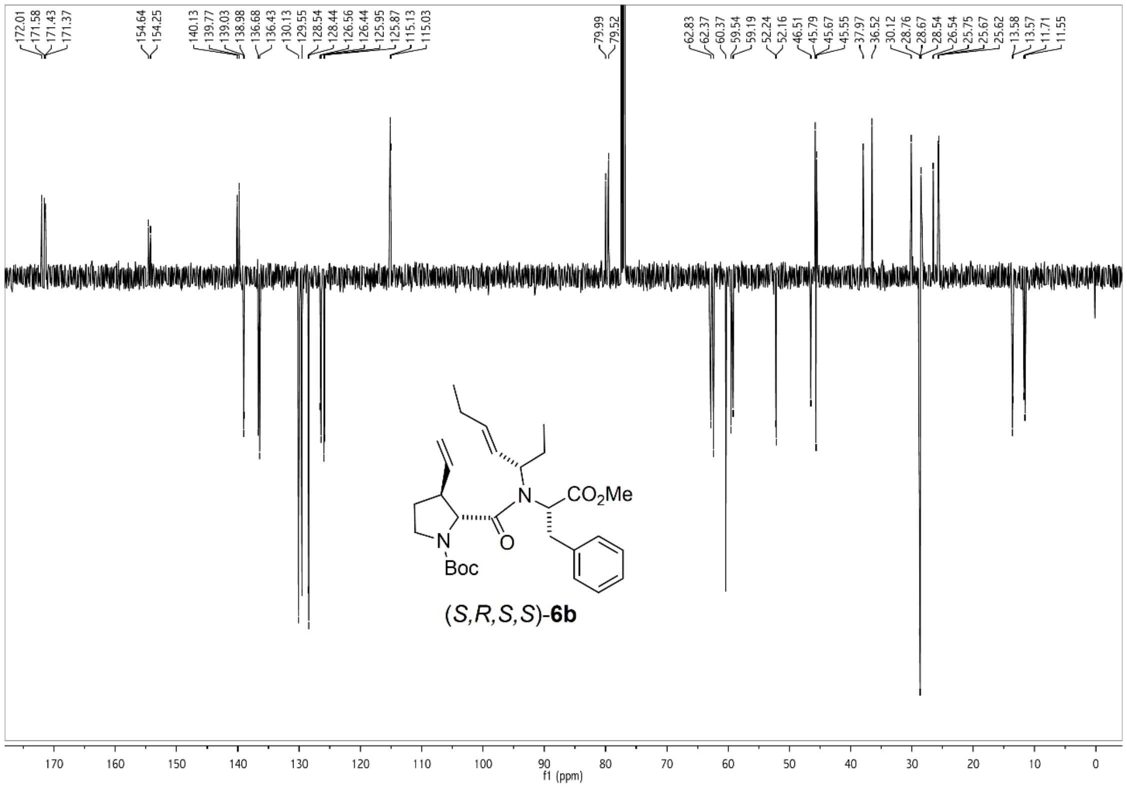

^{13}C-NMR (125 MHz) of *(S,R,S,S)*-**6b** in $CDCl_3$

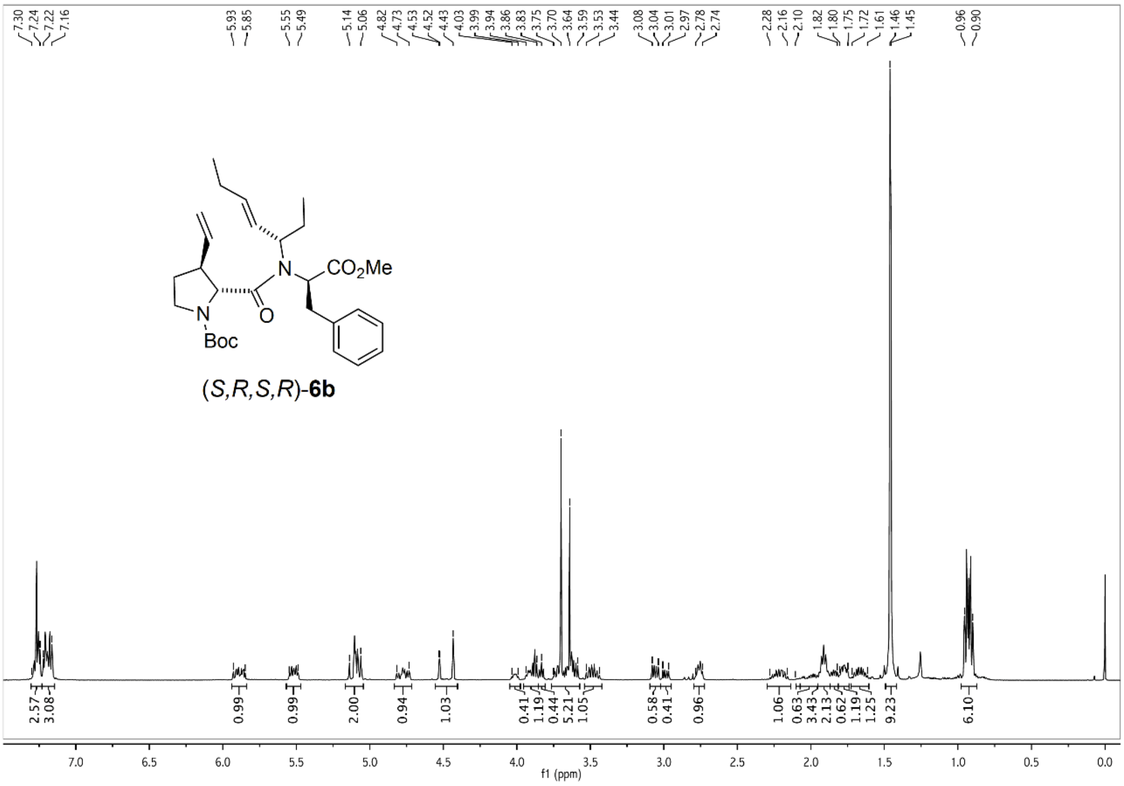

^{1}H NMR (500 MHz) of *(S,R,S,R)*-**6b** in $CDCl_3$

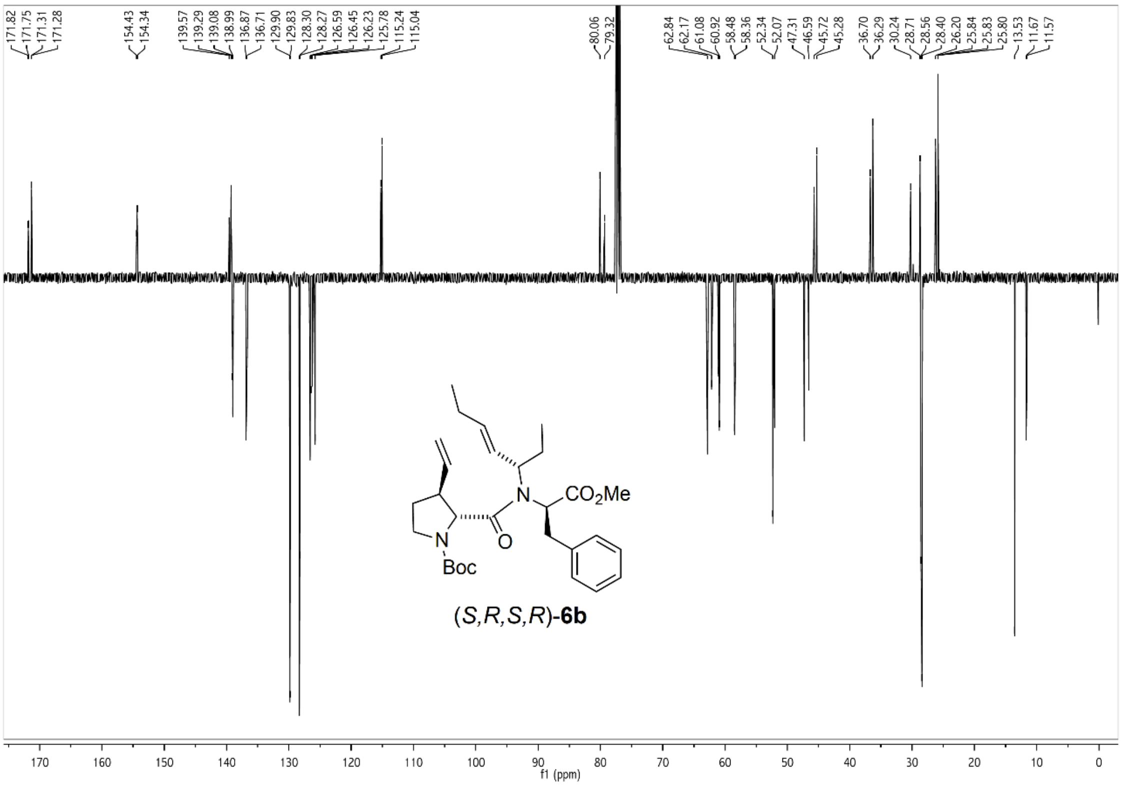

^{13}C-NMR (125 MHz) of *(S,R,S,R)*-**6b** in $CDCl_3$

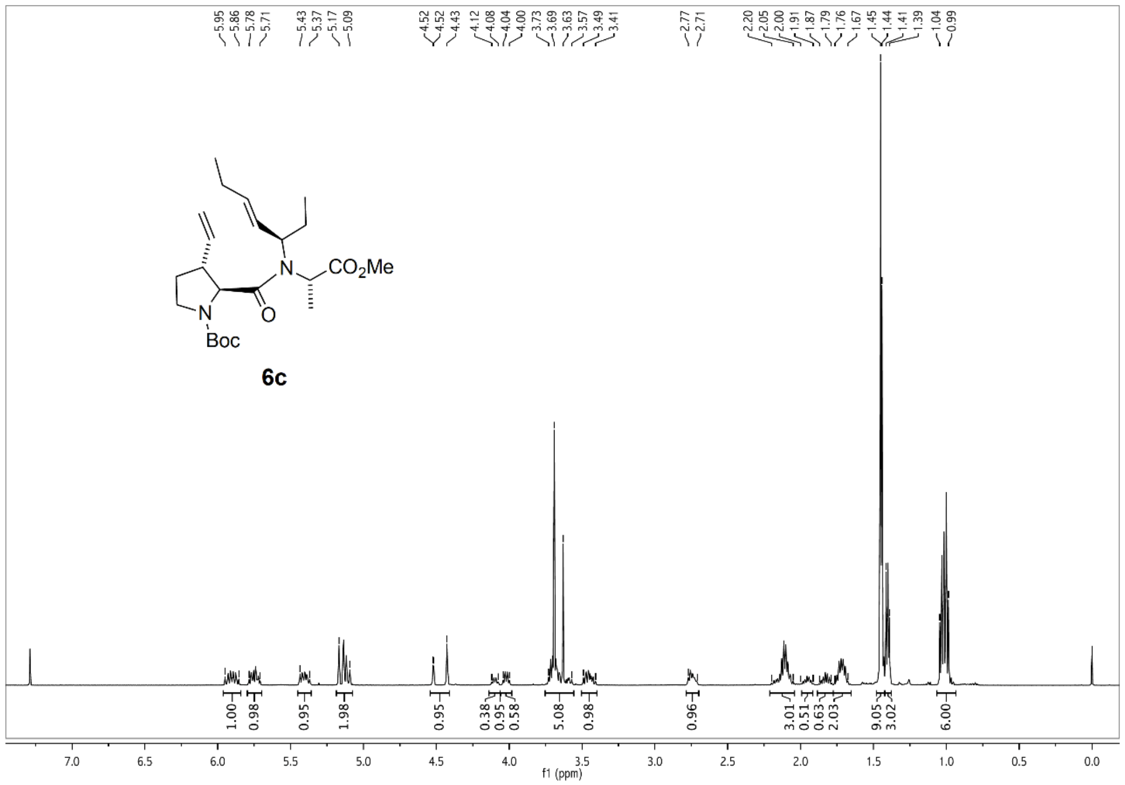

^{1}H NMR (500 MHz) of **6c** in $CDCl_3$

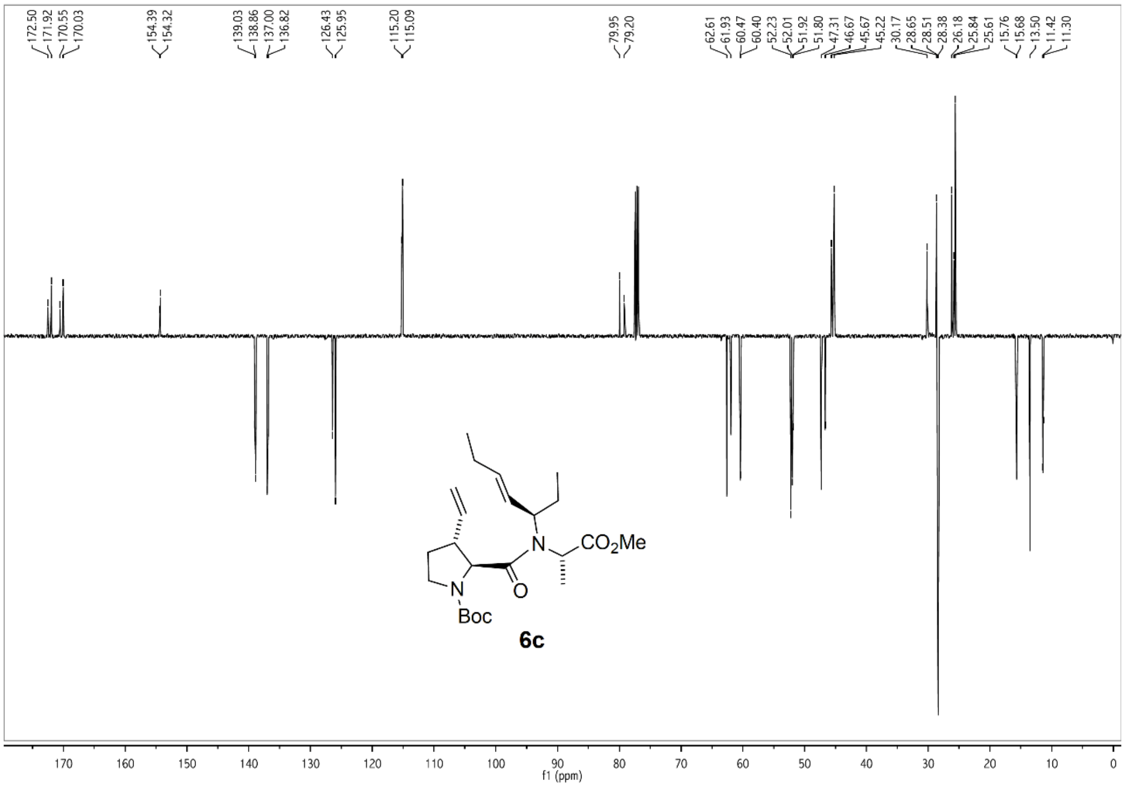

^{13}C-NMR (125 MHz) of **6c** in $CDCl_3$

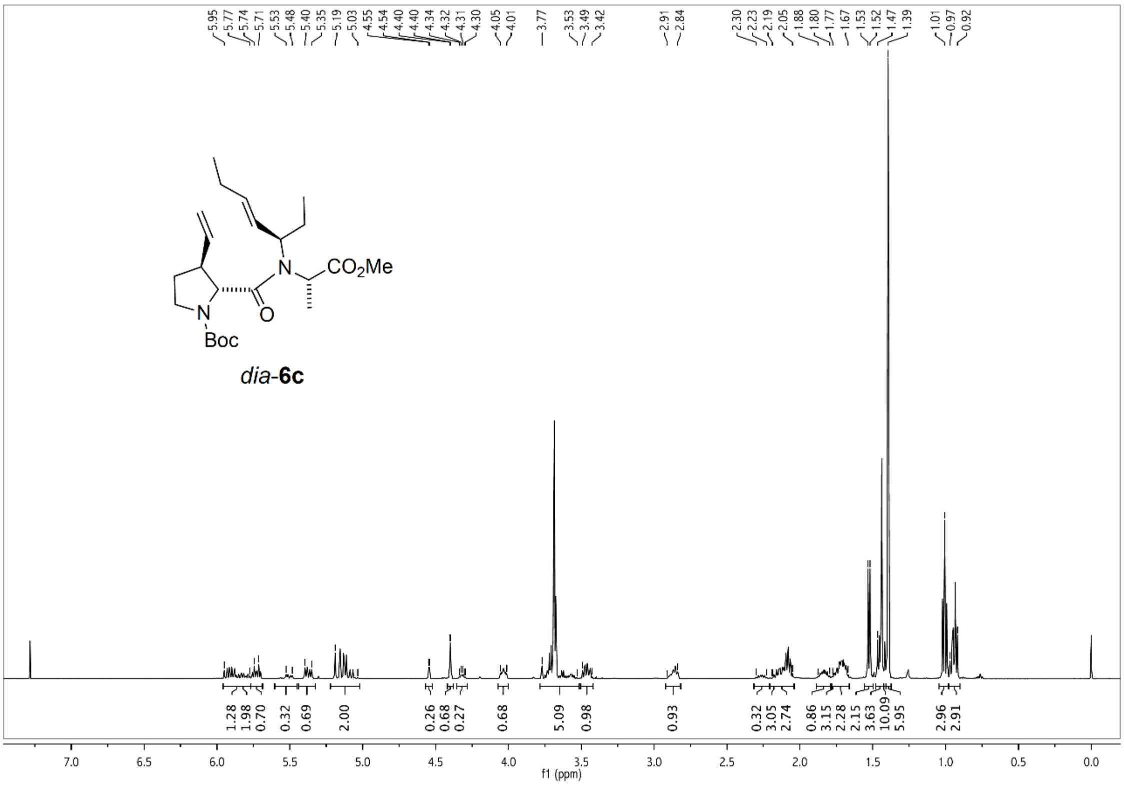

^{1}H NMR (500 MHz) of *dia*-**6c** in $CDCl_3$

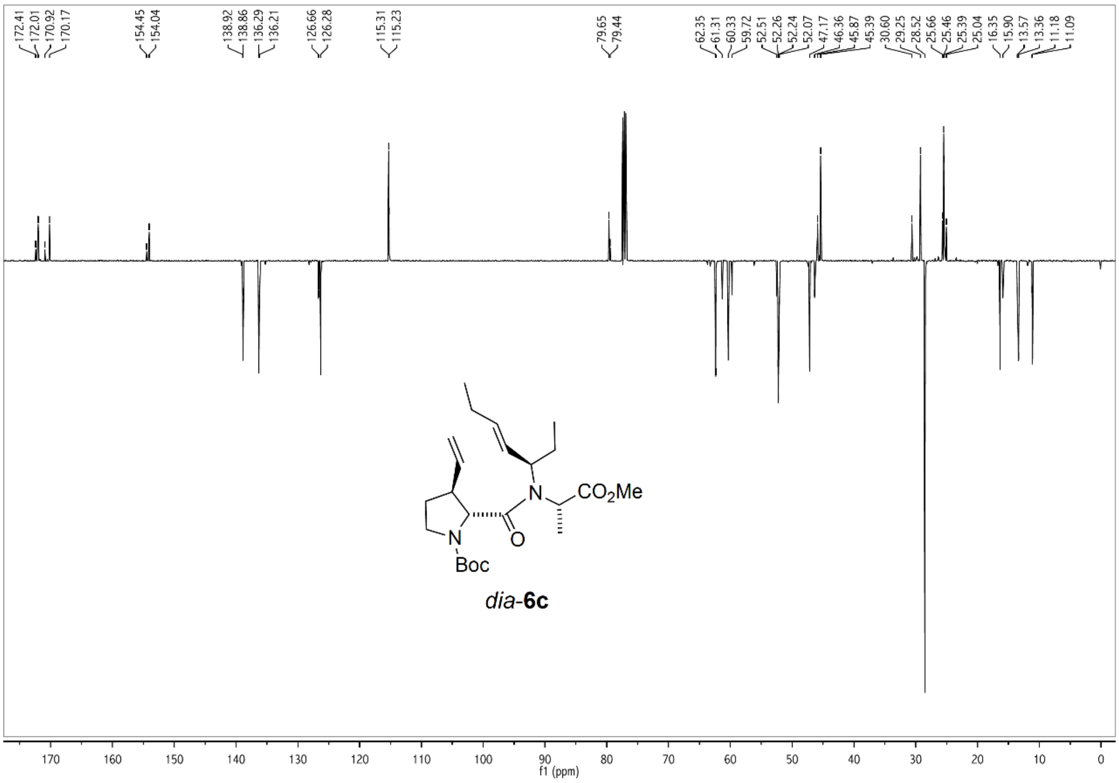

^{13}C-NMR (125 MHz) of *dia*-**6c** in $CDCl_3$

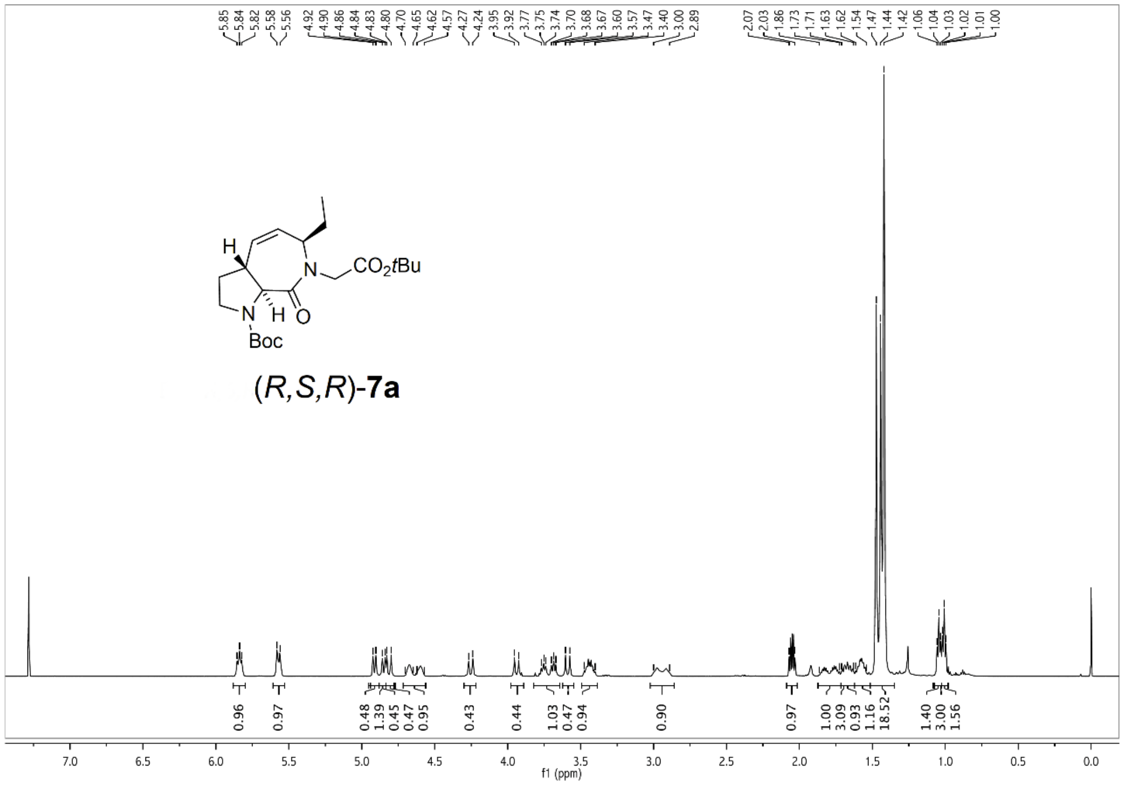

^{1}H NMR (500 MHz) of (*R,S,R*)-**6a** in $CDCl_3$

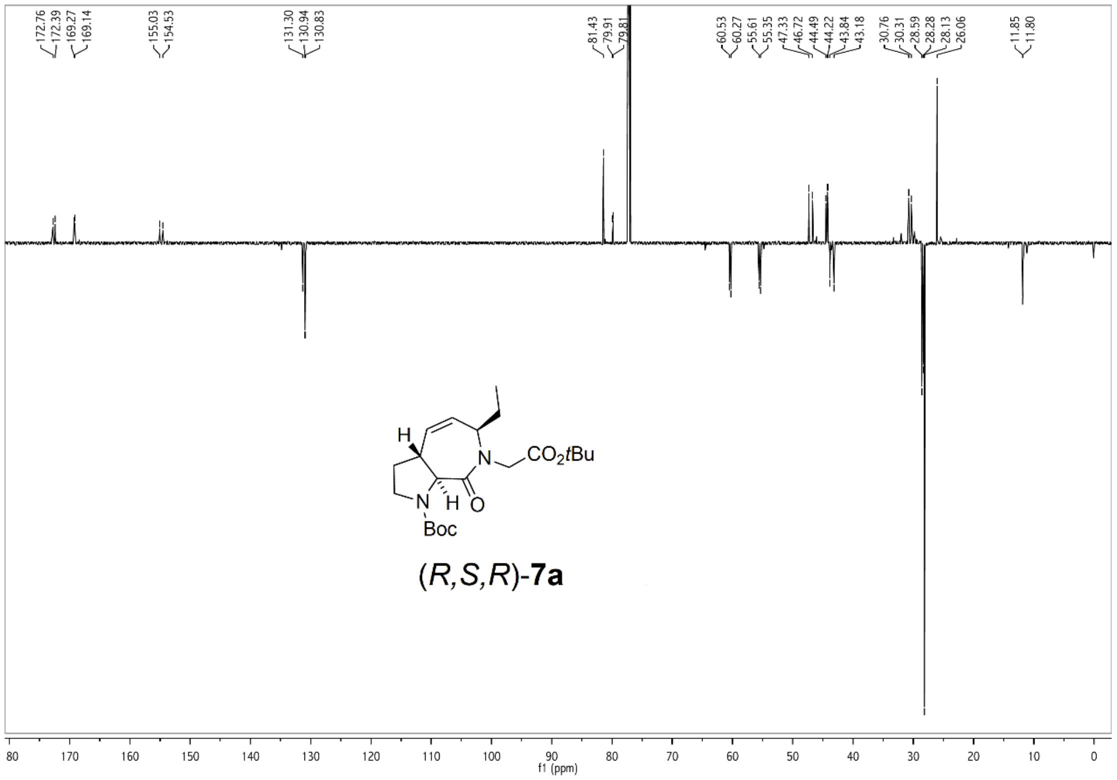

^{13}C-NMR (125 MHz) of (*R,S,R*)-**7a** in $CDCl_3$

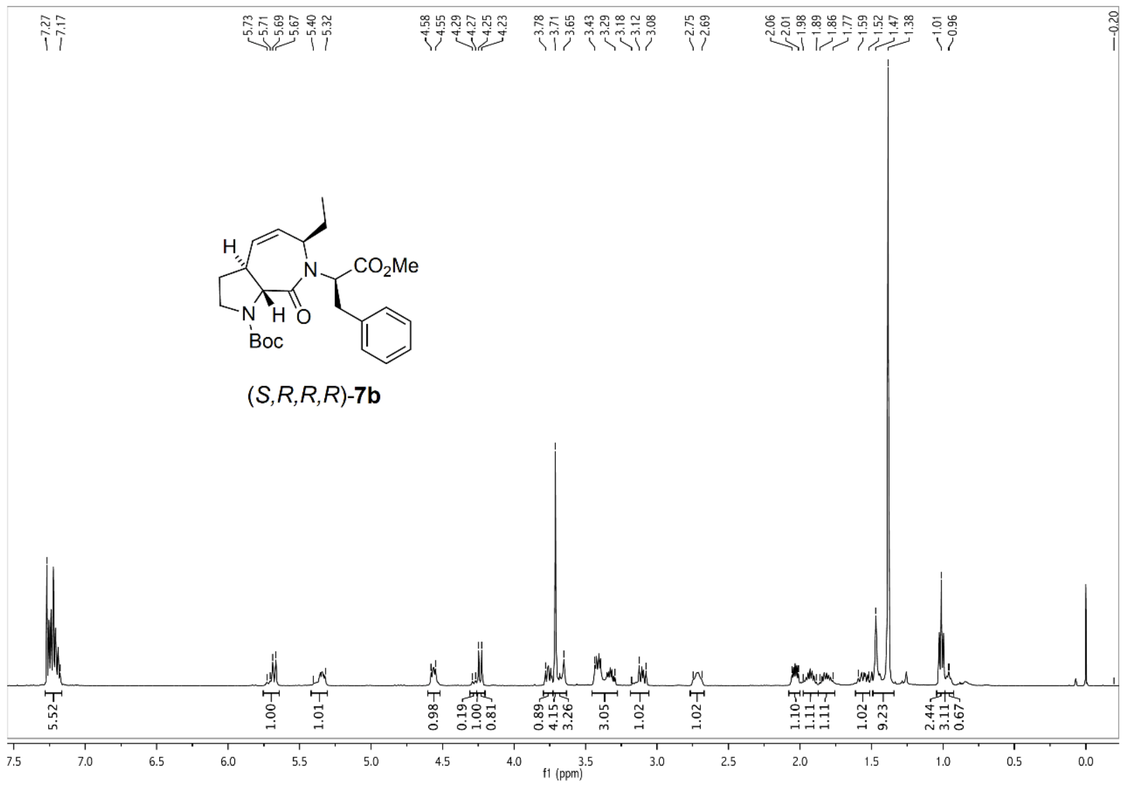

^{1}H NMR (500 MHz) of (*S,R,R,R*)-**7b** in $CDCl_3$

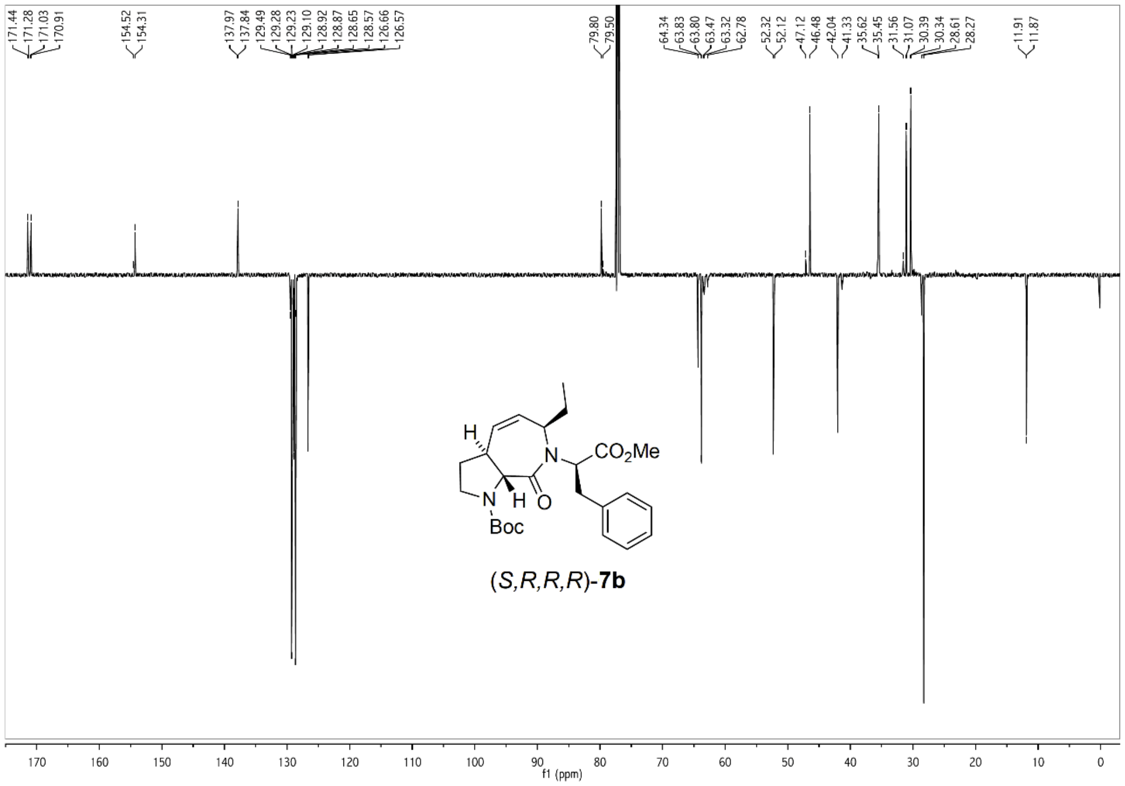

^{13}C-NMR (125 MHz) of (*S,R,R,R*)-**7b** in $CDCl_3$

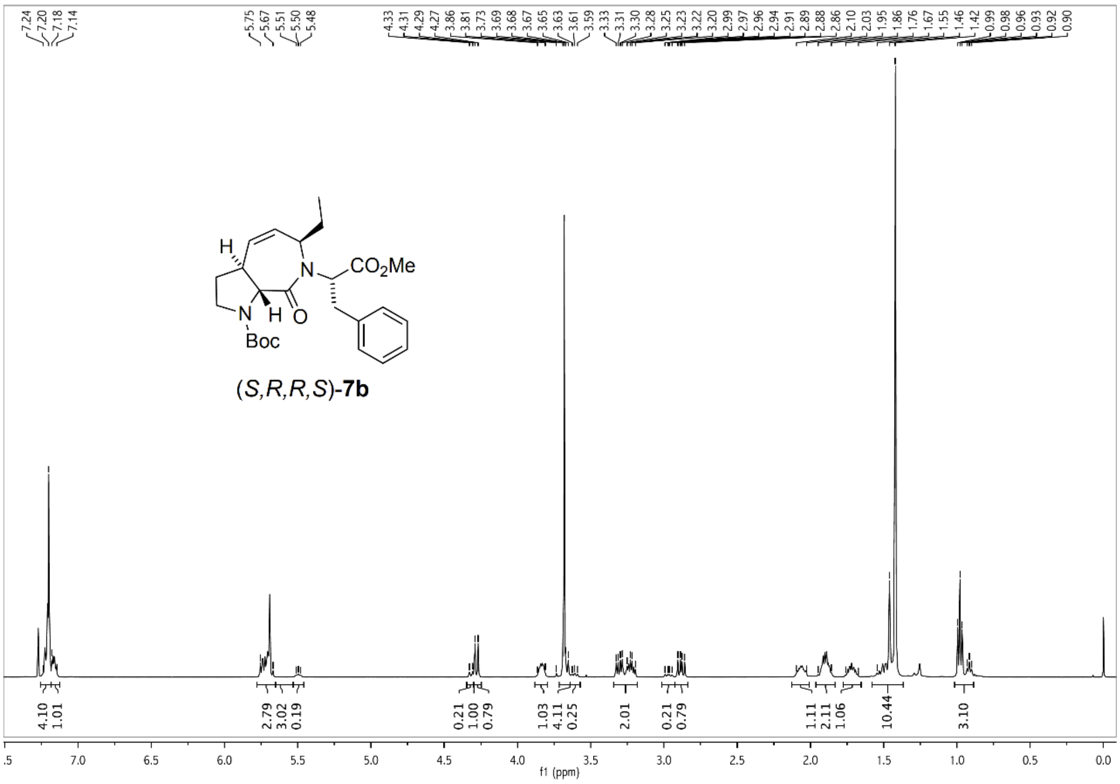

^{1}H NMR (500 MHz) of (*S,R,R,S*)-**7b** in $CDCl_3$

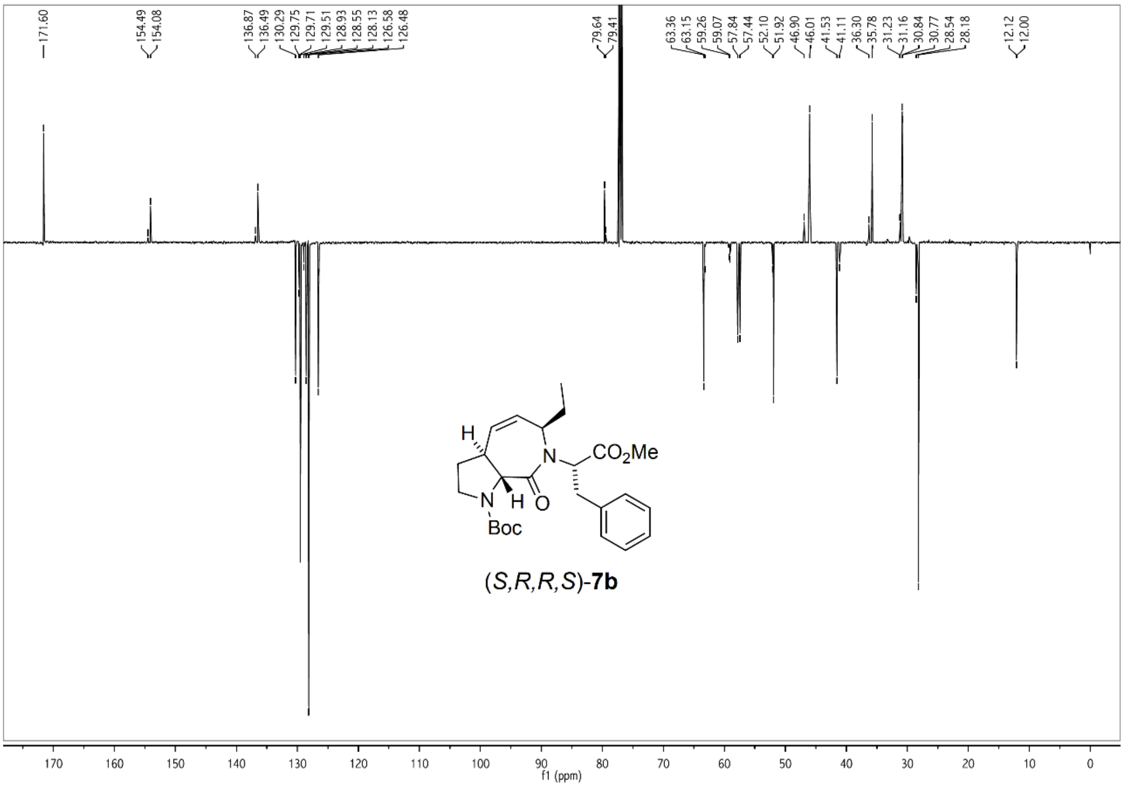

^{13}C-NMR (125 MHz) of (*S,R,R,S*)-**7b** in $CDCl_3$

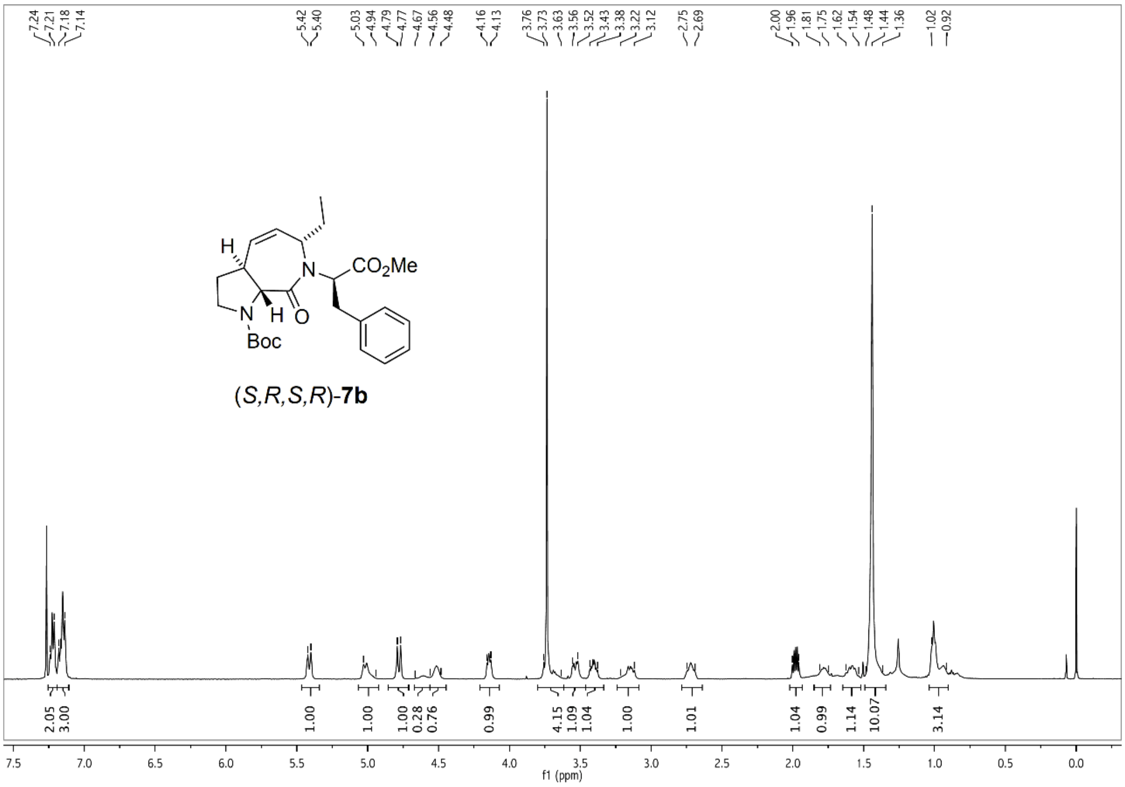

^{1}H NMR (500 MHz) of (*S,R,S,R*)-**7b** in $CDCl_3$

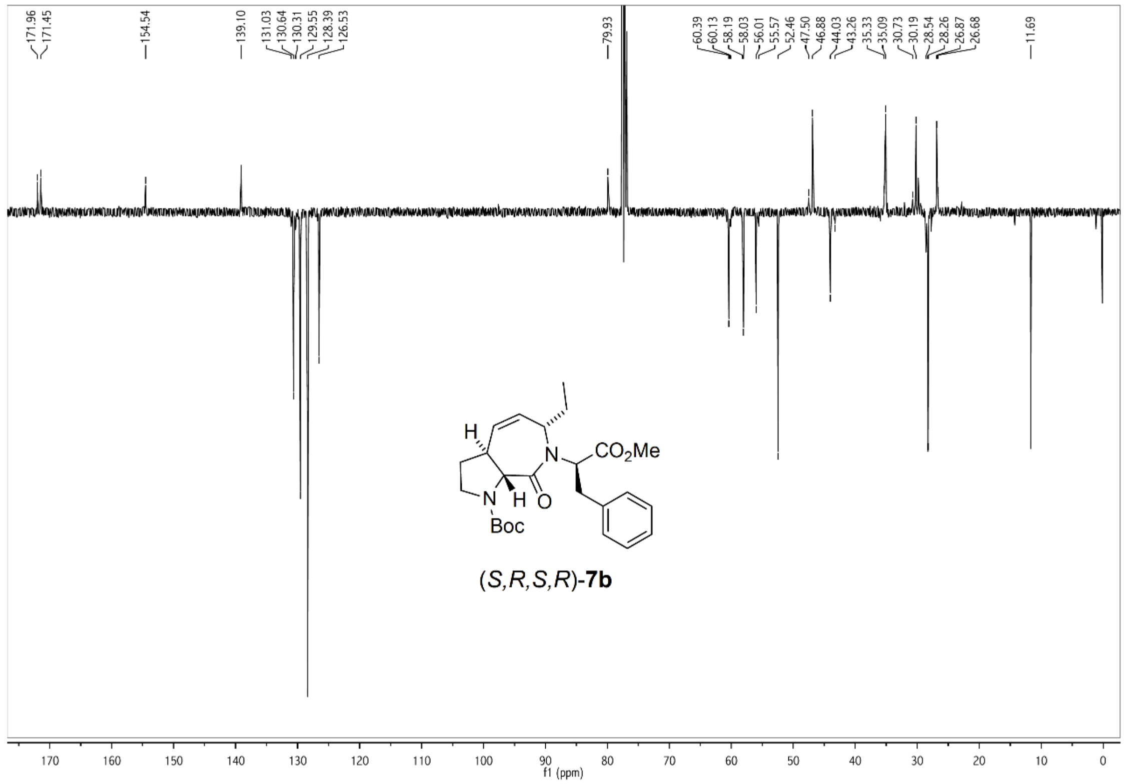

^{13}C-NMR (125 MHz) of (*S,R,S,R*)-**7b** in $CDCl_3$

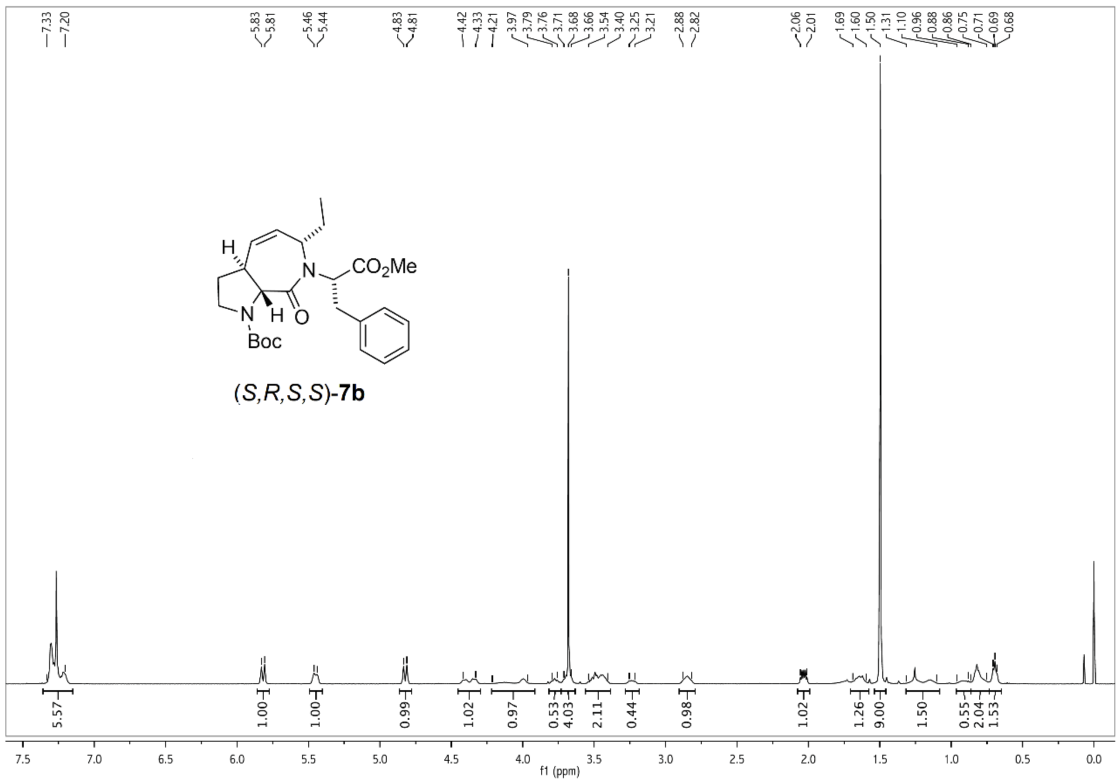

^{1}H NMR (500 MHz) of (*S,R,S,S*)-**7b** in $CDCl_3$

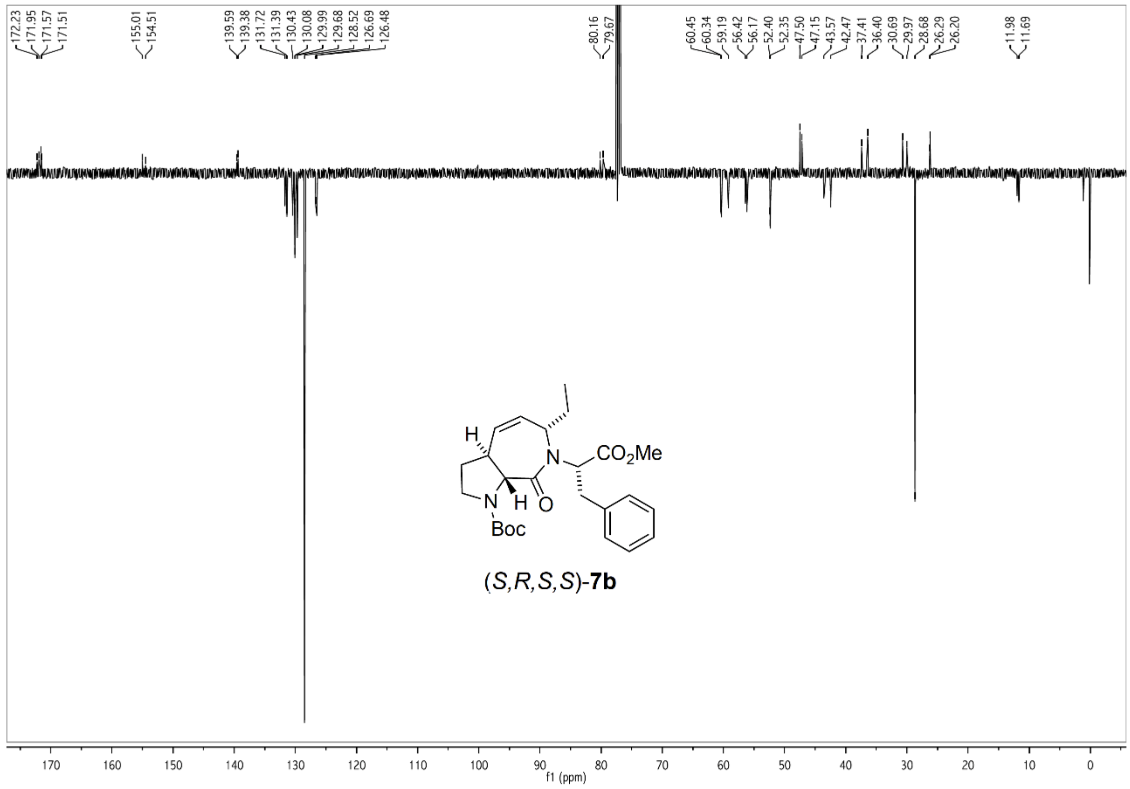

^{13}C-NMR (125 MHz) of (*S,R,S,S*)-**7b** in $CDCl_3$

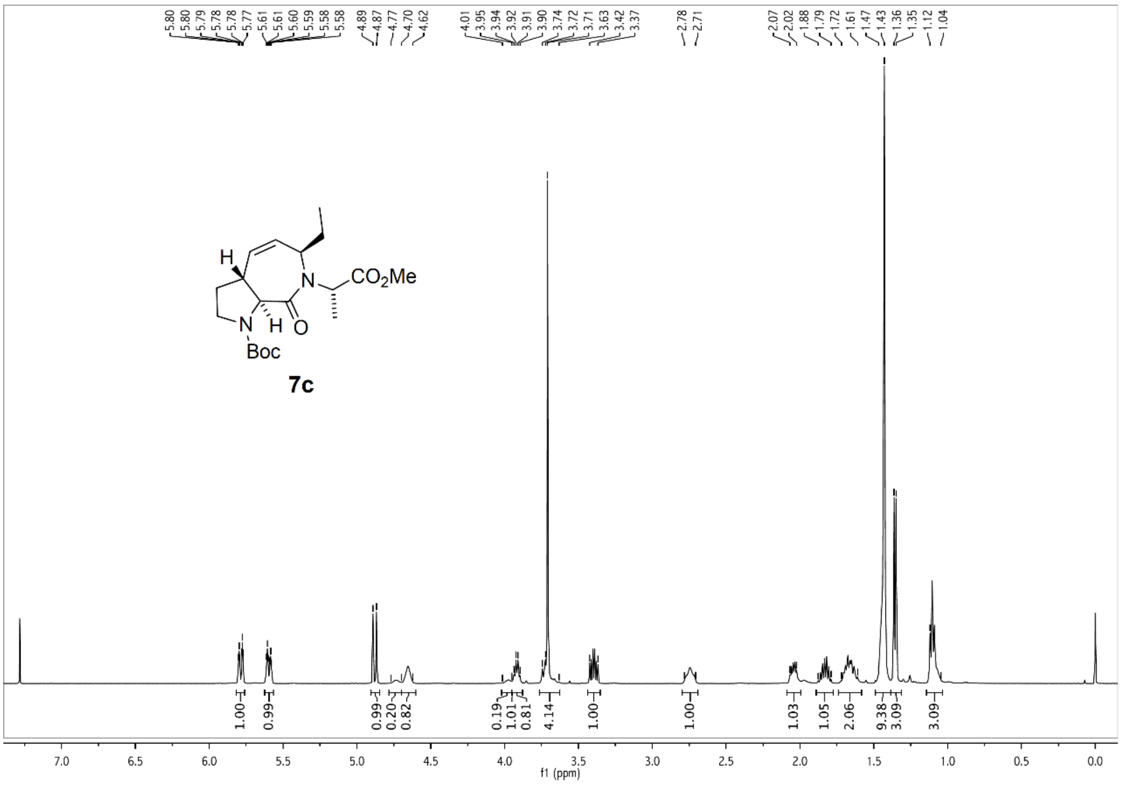

^{1}H NMR (500 MHz) of **7c** in $CDCl_3$

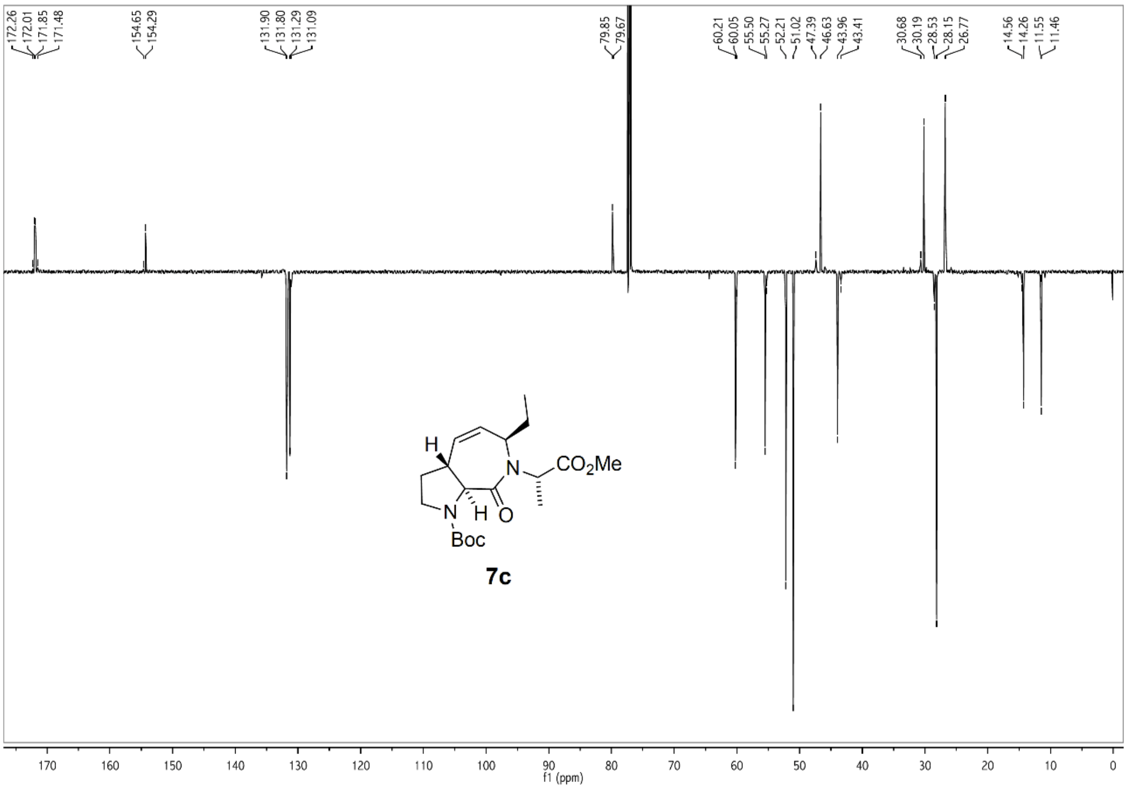

^{13}C-NMR (125 MHz) of **7c** in $CDCl_3$

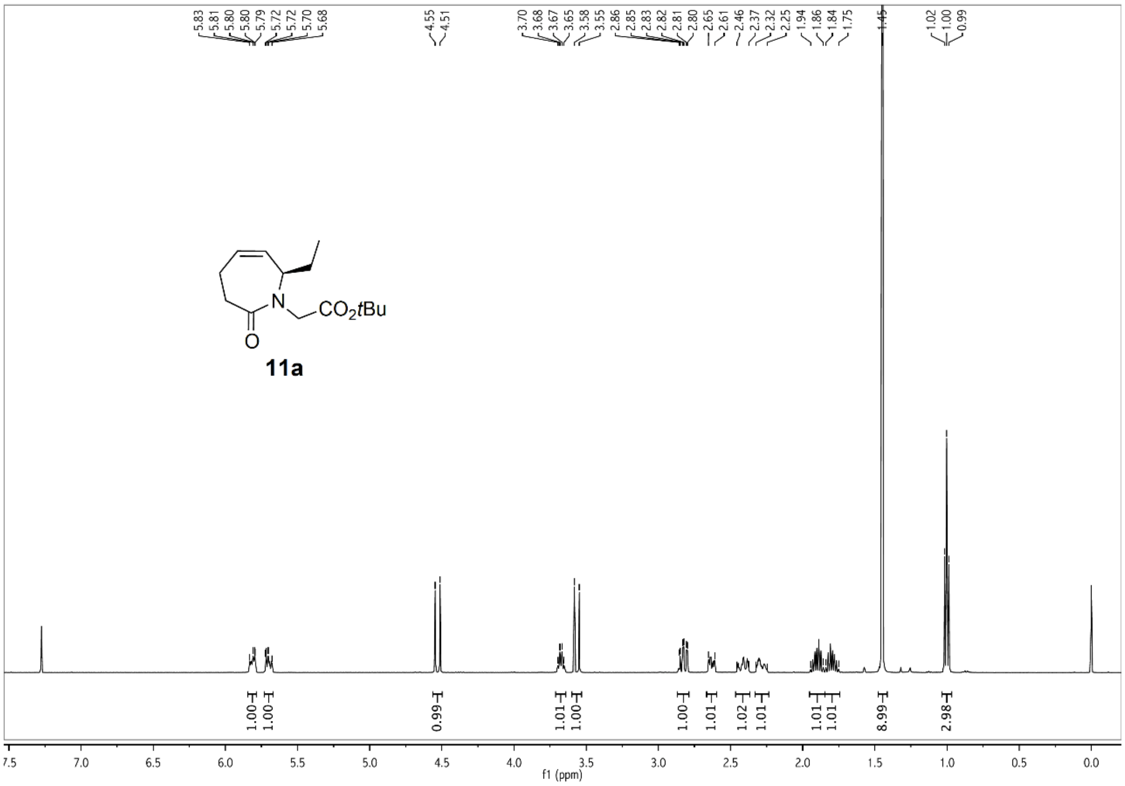

^{1}H NMR (500 MHz) of **11a** in $CDCl_3$

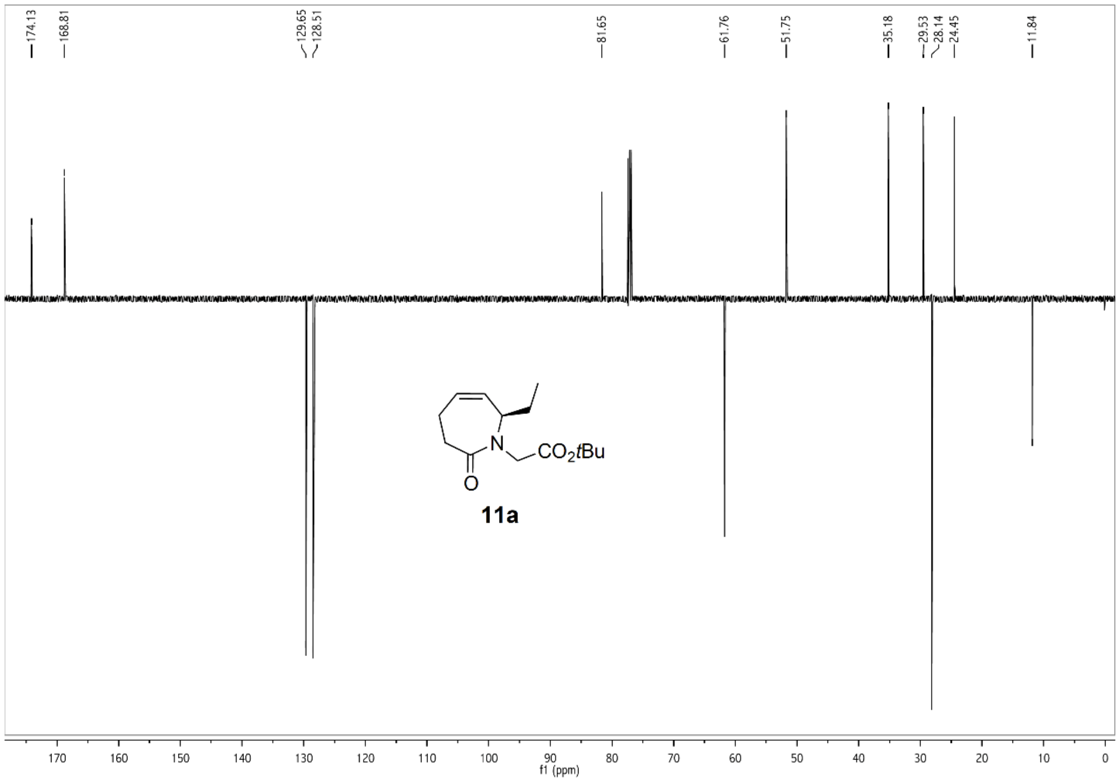

^{13}C-NMR (125 MHz) of **11a** in $CDCl_3$

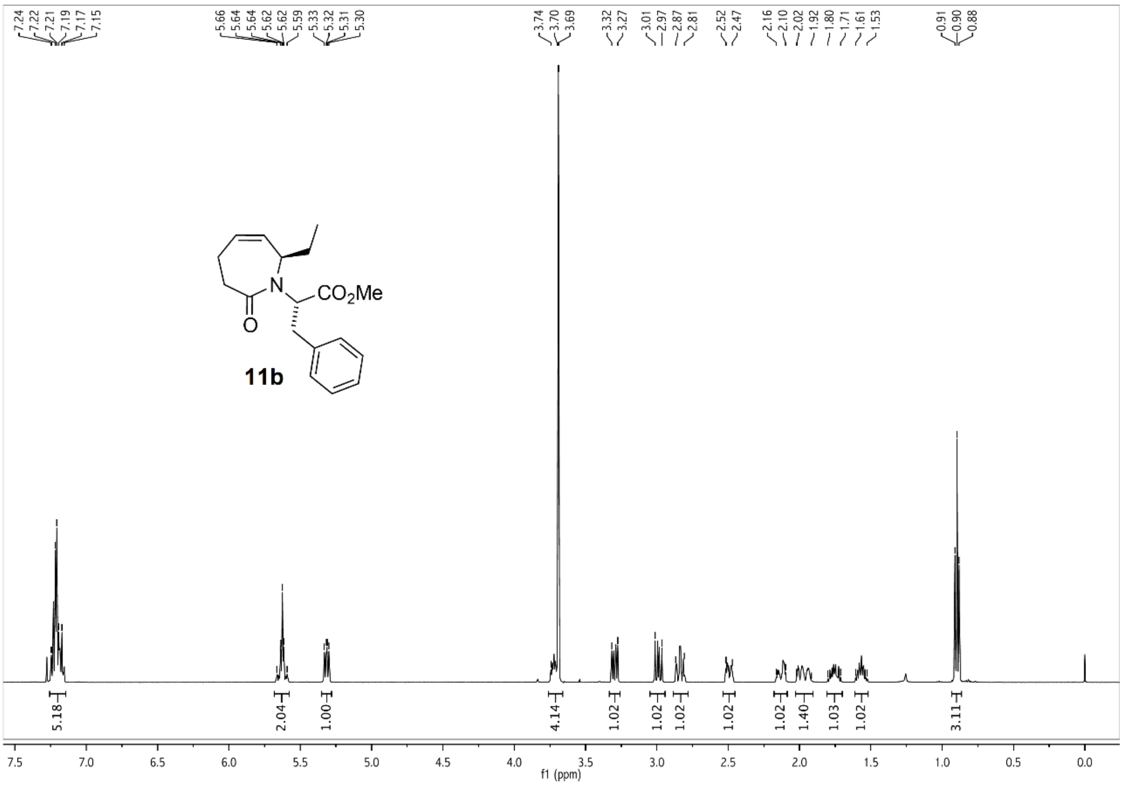

^{1}H NMR (500 MHz) of **11b** in $CDCl_3$

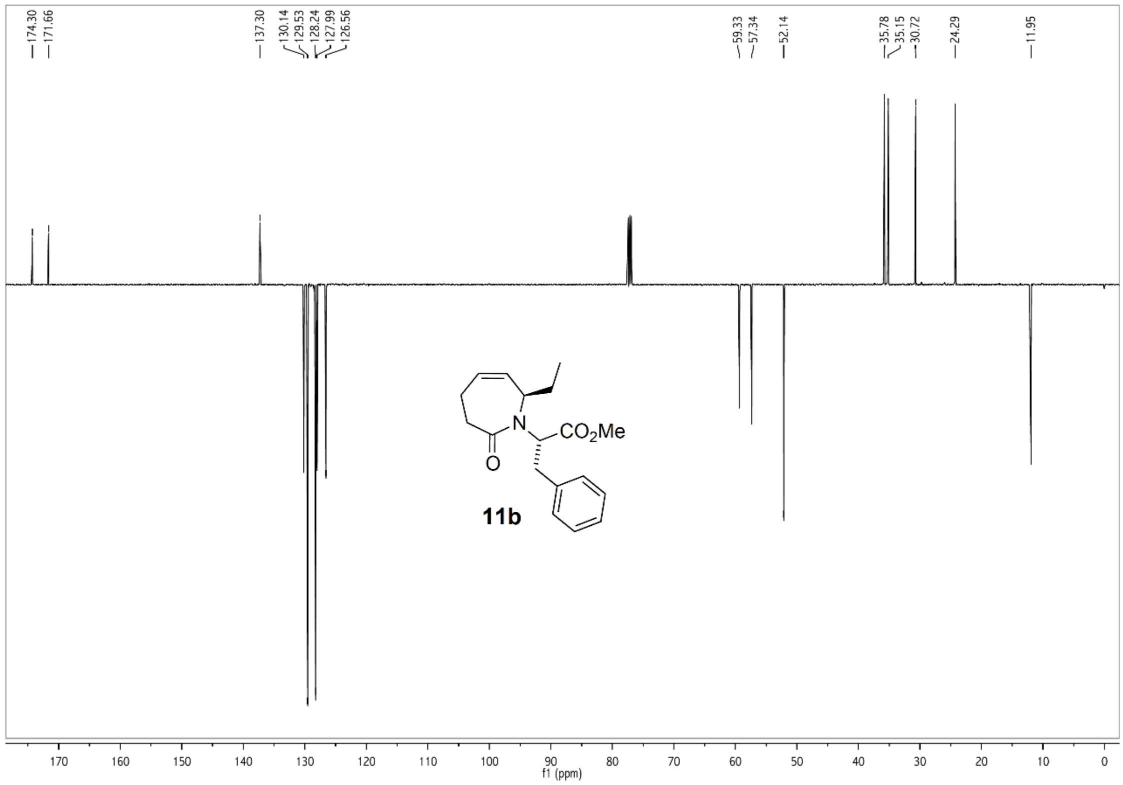

^{13}C-NMR (125 MHz) of **11b** in $CDCl_3$

Gas chromatograms of *rac*-3a and (*S*)-3a

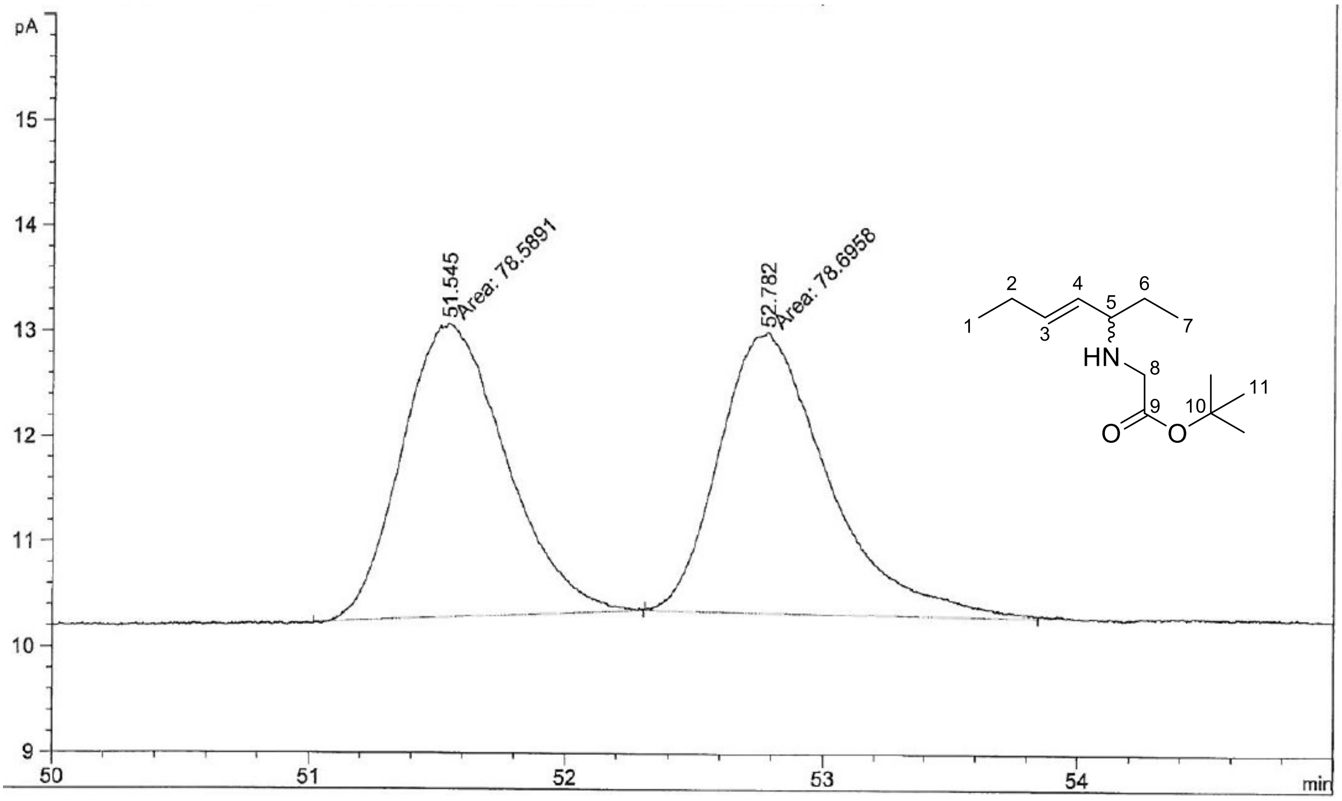

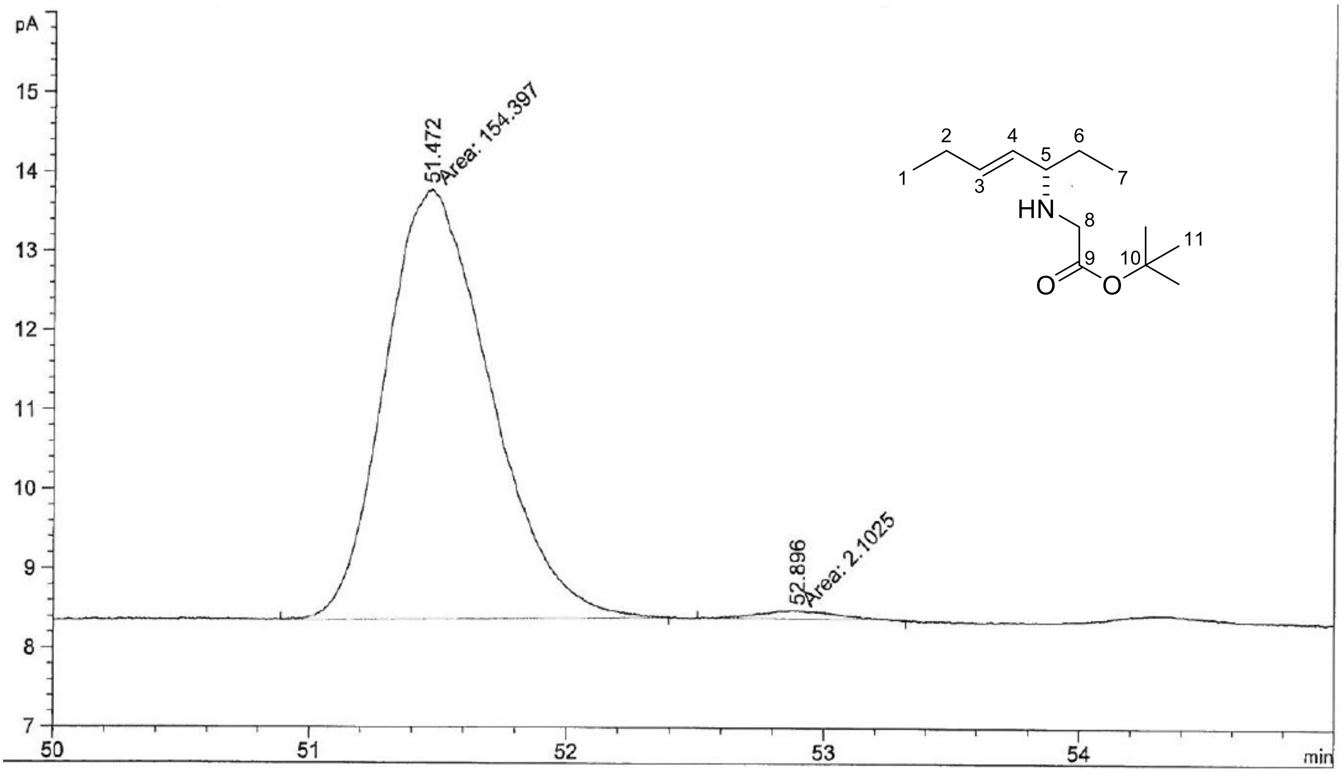

SUPPORTING INFORMATION

X-ray crystallographic data:

Data for **7a**

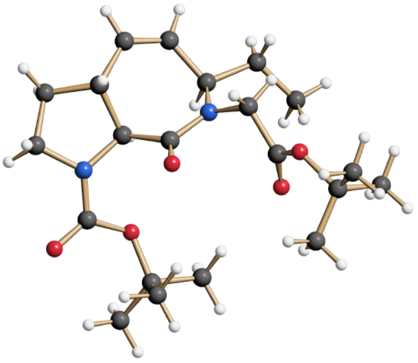

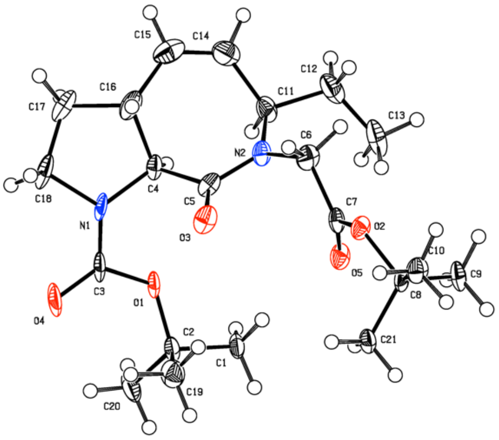

Identification code	sd759	
Empirical formula	$C_{21}H_{34}N_2O_5$	
Moiety formula	$C_{21}H_{34}N_2O_5$	
Formula weight	394.50	
Temperature	100(2) K	
Wavelength	1.54178 Å	
Crystal system	Monoclinic	
Space group	$P2_1$	
Unit cell dimensions	a = 10.5339(14) Å	α = 90°.
	b = 9.6639(12) Å	β = 104.865(11)°.
	c = 11.0835(19) Å	γ = 90°.
Volume	1090.5(3) $Å^3$	
Z	2	
Density (calculated)	1.201 Mg/m^3	
Absorption coefficient	0.693 mm^{-1}	
F(000)	428	
Crystal size	0.250 x 0.200 x 0.040 mm^3	
Theta range for data collection	4.127 to 65.226°.	
Index ranges	-12<=h<=12, -11<=k<=11, -13<=l<=13	
Reflections collected	13096	
Independent reflections	3700 [R(int) = 0.0520]	
Completeness to theta = 65.226°	99.9%	
Absorption correction	Semi-empirical from equivalents	
Max. and min. transmission	0.7526 and 0.4523	
Refinement method	Full-matrix least-squares on F^2	
Data / restraints / parameters	3700 / 1 / 260	
Goodness-of-fit on F^2	1.067	
Final R indices [I>2sigma(I)]	R1 = 0.0405, wR2 = 0.0985	
R indices (all data)	R1 = 0.0433, wR2 = 0.1012	
Absolute structure parameter	0.14(11)	
Extinction coefficient	n/a	
Largest diff. peak and hole	0.178 and -0.286 e.$Å^{-3}$	

Data for 7c

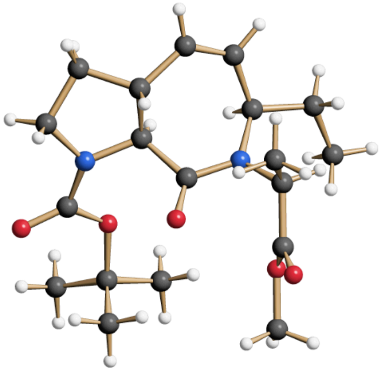

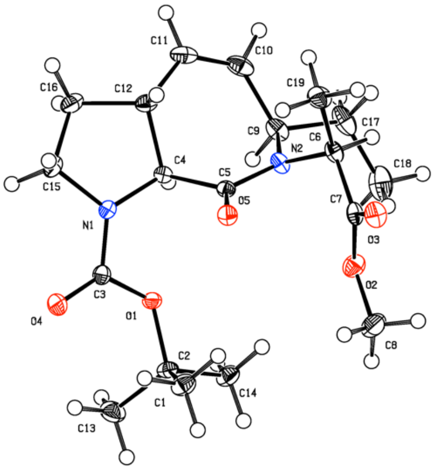

Identification code	sd665n	
Empirical formula	$C_{19}H_{30}N_2O_5$	
Moiety formula	$C_{19}H_{30}N_2O_5$	
Formula weight	366.45	
Temperature	100(2) K	
Wavelength	1.54178 Å	
Crystal system	Orthorhombic	
Space group	$P2_12_12_1$	
Unit cell dimensions	a = 10.7523(3) Å	α = 90°.
	b = 12.4063(3) Å	β = 90°.
	c = 14.8461(4) Å	γ = 90°.
Volume	1980.41(9) $Å^3$	
Z	4	
Density (calculated)	1.229 Mg/m^3	
Absorption coefficient	0.726 mm^{-1}	
F(000)	792	
Crystal size	0.200 x 0.160 x 0.120 mm^3	
Theta range for data collection	4.645 to 77.610°.	
Index ranges	-13<=h<=13, -15<=k<=15, -16<=l<=18	
Reflections collected	60691	
Independent reflections	4216 [R(int) = 0.0335]	
Completeness to theta = 67.679°	100.0%	
Absorption correction	Semi-empirical from equivalents	
Max. and min. transmission	0.8646 and 0.8047	
Refinement method	Full-matrix least-squares on F^2	
Data / restraints / parameters	4216 / 0 / 242	
Goodness-of-fit on F^2	1.047	
Final R indices [I>2sigma(I)]	R1 = 0.0233, wR2 = 0.0604	
R indices (all data)	R1 = 0.0238, wR2 = 0.0608	
Absolute structure parameter	0.01(2)	
Extinction coefficient	0.0035(3)	
Largest diff. peak and hole	0.246 and -0.133 e.$Å^{-3}$	

Data for (*S*,*R*,*S*,*S*)-7b

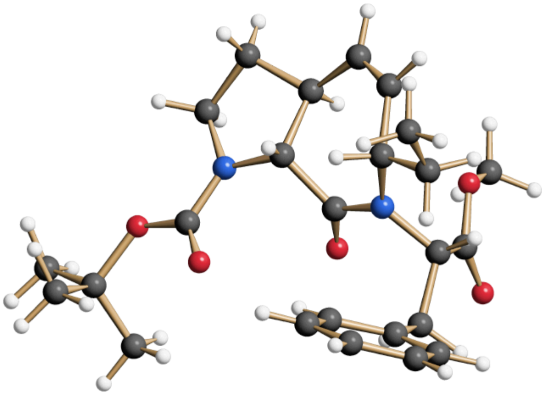

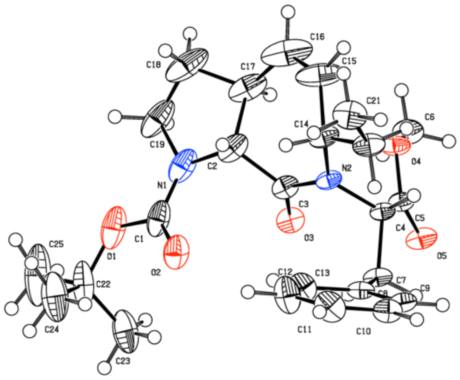

Identification code	sd626_1	
Empirical formula	$C_{25}H_{34}N_2O_5$	
Moiety formula	$C_{25}H_{34}N_2O_5$	
Formula weight	442.54	
Temperature	100(2) K	
Wavelength	1.54178 Å	
Crystal system	Orthorhombic	
Space group	$P2_12_12_1$	
Unit cell dimensions	a = 8.4270(2) Å	α = 90°.
	b = 9.3160(2) Å	β = 90°.
	c = 31.6237(8) Å	γ = 90°.
Volume	2482.65(10) $Å^3$	
Z	4	
Density (calculated)	1.184 Mg/m^3	
Absorption coefficient	0.666 mm^{-1}	
F(000)	952	
Crystal size	0.150 x 0.150 x 0.030 mm^3	
Theta range for data collection	4.949 to 72.203°.	
Index ranges	-10<=h<=10, -11<=k<=11, -39<=l<=38	
Reflections collected	31065	
Independent reflections	4897 [R(int) = 0.0366]	
Completeness to theta = 67.679°	100.0%	
Absorption correction	Semi-empirical from equivalents	
Max. and min. transmission	0.7536 and 0.6626	
Refinement method	Full-matrix least-squares on F^2	
Data / restraints / parameters	4897 / 0 / 294	
Goodness-of-fit on F^2	1.068	
Final R indices [I>2sigma(I)]	R1 = 0.0452, wR2 = 0.1125	
R indices (all data)	R1 = 0.0468, wR2 = 0.1137	
Absolute structure parameter	0.07(4)	
Extinction coefficient	n/a	
Largest diff. peak and hole	0.593 and -0.268 e.$Å^{-3}$	

Data for (*S*,*R*,*R*,*S*)-7b

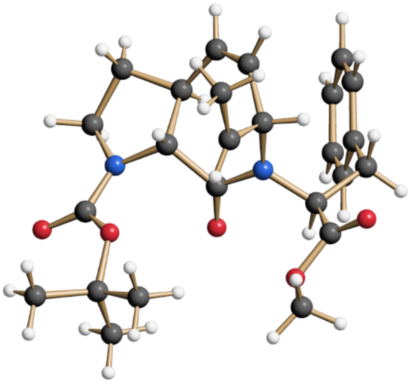

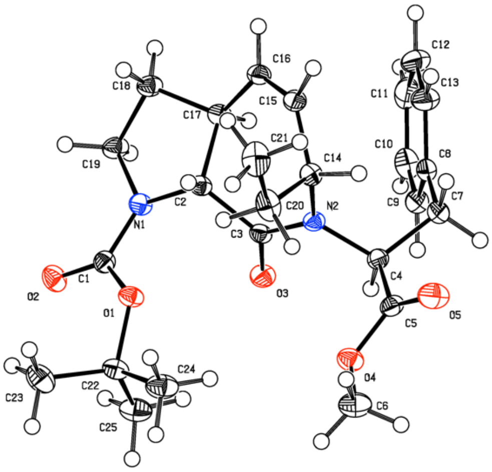

Identification code	sd620	
Empirical formula	$C_{25}H_{34}N_2O_5$	
Moiety formula	$C_{25}H_{34}N_2O_5$	
Formula weight	442.54	
Temperature	100(2) K	
Wavelength	1.54178 Å	
Crystal system	Monoclinic	
Space group	$P2_1$	
Unit cell dimensions	a = 9.3016(3) Å	α = 90°.
	b = 21.2294(7) Å	β = 92.7160(10)°.
	c = 12.1312(4) Å	γ = 90°.
Volume	2392.83(14) Å^3	
Z	4	
Density (calculated)	1.228 Mg/m^3	
Absorption coefficient	0.691 mm^{-1}	
F(000)	952	
Crystal size	0.200 x 0.200 x 0.100 mm^3	
Theta range for data collection	3.647 to 72.365°.	
Index ranges	-11<=h<=11, -26<=k<=26, -13<=l<=14	
Reflections collected	27458	
Independent reflections	9274 [R(int) = 0.0351]	
Completeness to theta = 67.679°	99.2%	
Absorption correction	Semi-empirical from equivalents	
Max. and min. transmission	0.7536 and 0.5586	
Refinement method	Full-matrix least-squares on F^2	
Data / restraints / parameters	9274 / 1 / 587	
Goodness-of-fit on F^2	1.074	
Final R indices [I>2sigma(I)]	R1 = 0.0330, wR2 = 0.0888	
R indices (all data)	R1 = 0.0343, wR2 = 0.0935	
Absolute structure parameter	0.13(5)	
Extinction coefficient	n/a	
Largest diff. peak and hole	0.718 and -0.230 e.Å^{-3}	

Data for (*S*,*R*,*S*,*R*)-**7b**

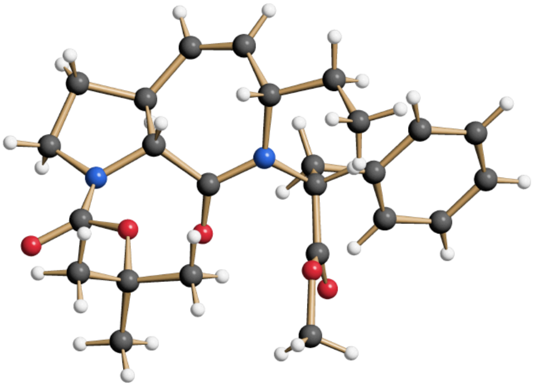

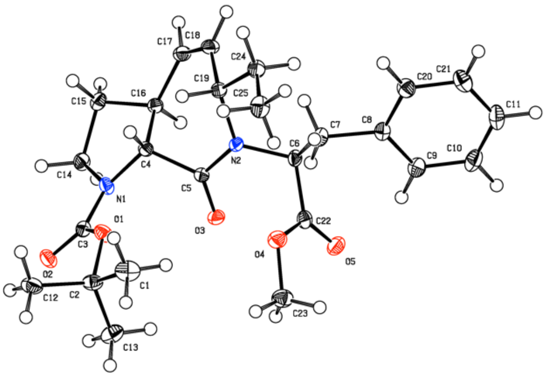

Identification code	sd758	
Empirical formula	$C_{25}H_{34}N_2O_5$	
Moiety formula	$C_{25}H_{34}N_2O_5$	
Formula weight	442.54	
Temperature	100(2) K	
Wavelength	1.54178 Å	
Crystal system	Orthorhombic	
Space group	$P2_12_12_1$	
Unit cell dimensions	a = 9.5447(11) Å	α = 90°.
	b = 14.5784(17) Å	β = 90°.
	c = 16.887(3) Å	γ = 90°.
Volume	2349.8(5) $Å^3$	
Z	4	
Density (calculated)	1.251 Mg/m^3	
Absorption coefficient	0.704 mm^{-1}	
F(000)	952	
Crystal size	0.150 x 0.030 x 0.020 mm^3	
Theta range for data collection	4.006 to 72.348°.	
Index ranges	-11<=h<=11, -18<=k<=12, -20<=l<=20	
Reflections collected	24130	
Independent reflections	4623 [R(int) = 0.0580]	
Completeness to theta = 67.679°	100.0%	
Absorption correction	Semi-empirical from equivalents	
Max. and min. transmission	0.7536 and 0.5330	
Refinement method	Full-matrix least-squares on F^2	
Data / restraints / parameters	4623 / 0 / 294	
Goodness-of-fit on F^2	1.091	
Final R indices [I>2sigma(I)]	R1 = 0.0302, wR2 = 0.0749	
R indices (all data)	R1 = 0.0320, wR2 = 0.0761	
Absolute structure parameter	0.05(6)	
Extinction coefficient	n/a	
Largest diff. peak and hole	0.277 and -0.226 e.$Å^{-3}$	

Data for (*S*,*R*,*R*,*R*)-7b

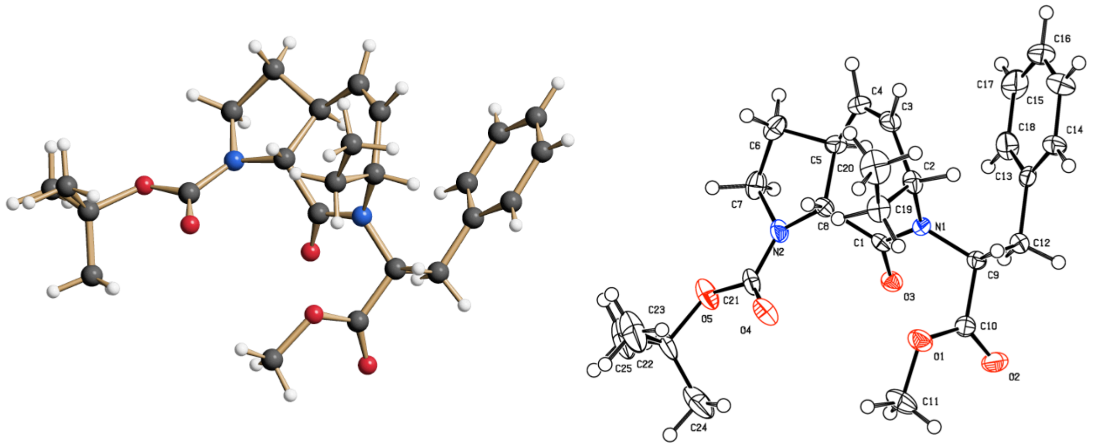

Identification code	sd777	
Empirical formula	$C_{30}H_{46}N_2O_6$	
Moiety formula	$C_{25}H_{34}N_2O_5$, $C_5H_{12}O$	
Formula weight	530.69	
Temperature	100(2) K	
Wavelength	1.54178 Å	
Crystal system	Orthorhombic	
Space group	$P2_12_12_1$	
Unit cell dimensions	a = 10.2223(5) Å	α = 90°.
	b = 10.4398(5) Å	β = 90°.
	c = 27.5410(13) Å	γ = 90°.
Volume	2939.1(2) $Å^3$	
Z	4	
Density (calculated)	1.199 Mg/m^3	
Absorption coefficient	0.666 mm^{-1}	
F(000)	1152	
Crystal size	0.070 x 0.070 x 0.010 mm^3	
Theta range for data collection	3.209 to 66.652°.	
Index ranges	-11<=h<=12, -12<=k<=11, -30<=l<=32	
Reflections collected	21611	
Independent reflections	5107 [R(int) = 0.0526]	
Completeness to theta = 66.652°	99.5%	
Absorption correction	Semi-empirical from equivalents	
Max. and min. transmission	0.7528 and 0.4728	
Refinement method	Full-matrix least-squares on F^2	
Data / restraints / parameters	5107 / 0 / 352	
Goodness-of-fit on F^2	1.135	
Final R indices [I>2sigma(I)]	R1 = 0.0707, wR2 = 0.2043	
R indices (all data)	R1 = 0.0775, wR2 = 0.2106	
Absolute structure parameter	0.28(11)	
Extinction coefficient	n/a	
Largest diff. peak and hole	0.609 and -0.642 e.$Å^{-3}$	

Data for 11a

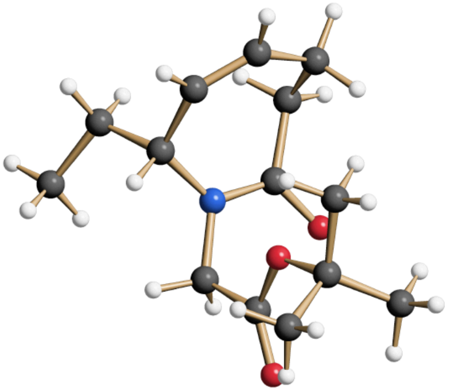

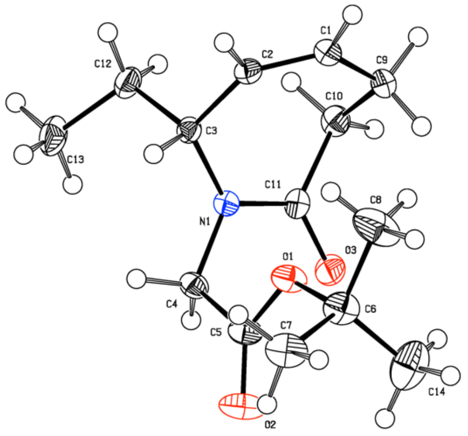

Identification code	sd726	
Empirical formula	$C_{14}H_{23}NO_3$	
Moiety formula	$C_{14}H_{23}NO_3$	
Formula weight	253.33	
Temperature	100(2) K	
Wavelength	1.54178 Å	
Crystal system	Monoclinic	
Space group	$P2_1$	
Unit cell dimensions	a = 8.6307(10) Å	α = 90°.
	b = 9.7649(14) Å	β = 106.921(8)°.
	c = 9.1877(19) Å	γ = 90°.
Volume	740.8(2) $Å^3$	
Z	2	
Density (calculated)	1.136 Mg/m^3	
Absorption coefficient	0.637 mm^{-1}	
F(000)	276	
Crystal size	0.600 x 0.200 x 0.200 mm^3	
Theta range for data collection	6.194 to 72.224°.	
Index ranges	-10<=h<=10, -10<=k<=11, -11<=l<=11	
Reflections collected	24734	
Independent reflections	2827 [R(int) = 0.0383]	
Completeness to theta = 67.679°	99.6%	
Absorption correction	Semi-empirical from equivalents	
Max. and min. transmission	0.7536 and 0.5623	
Refinement method	Full-matrix least-squares on F^2	
Data / restraints / parameters	2827 / 1 / 168	
Goodness-of-fit on F^2	1.054	
Final R indices [I>2sigma(I)]	R1 = 0.0284, wR2 = 0.0805	
R indices (all data)	R1 = 0.0287, wR2 = 0.0807	
Absolute structure parameter	0.04(5)	
Extinction coefficient	0.059(5)	
Largest diff. peak and hole	0.218 and -0.178 e.$Å^{-3}$	

Data for 11e

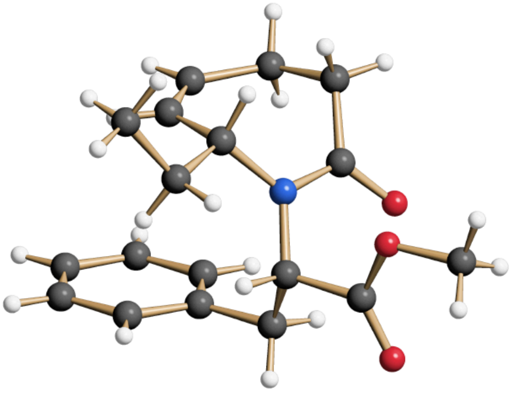

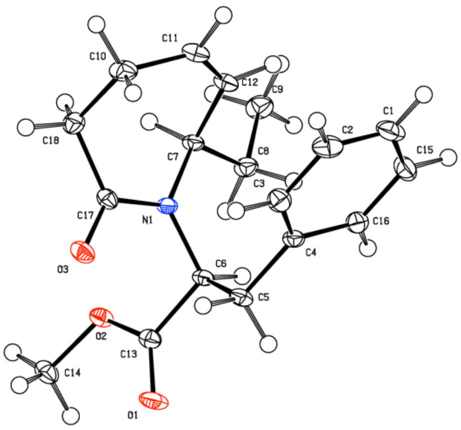

Identification code	sd678	
Empirical formula	$C_{18}H_{23}NO_3$	
Moiety formula	$C_{18}H_{23}NO_3$	
Formula weight	301.37	
Temperature	100(2) K	
Wavelength	1.54178 Å	
Crystal system	Monoclinic	
Space group	$P2_1$	
Unit cell dimensions	a = 7.4223(9) Å	α = 90°.
	b = 10.3697(13) Å	β = 90.479(8)°.
	c = 10.3426(10) Å	γ = 90°.
Volume	796.01(16) $Å^3$	
Z	2	
Density (calculated)	1.257 Mg/m^3	
Absorption coefficient	0.683 mm^{-1}	
F(000)	324	
Crystal size	0.500 x 0.100 x 0.100 mm^3	
Theta range for data collection	6.043 to 72.179°.	
Index ranges	-9<=h<=9, -11<=k<=12, -12<=l<=11	
Reflections collected	10683	
Independent reflections	3036 [R(int) = 0.0354]	
Completeness to theta = 67.679°	99.5%	
Absorption correction	Semi-empirical from equivalents	
Max. and min. transmission	0.7536 and 0.5982	
Refinement method	Full-matrix least-squares on F^2	
Data / restraints / parameters	3036 / 1 / 202	
Goodness-of-fit on F^2	1.038	
Final R indices [I>2sigma(I)]	R1 = 0.0292, wR2 = 0.0743	
R indices (all data)	R1 = 0.0296, wR2 = 0.0748	
Absolute structure parameter	-0.02(6)	
Extinction coefficient	0.025(2)	
Largest diff. peak and hole	0.230 and -0.195 e.$Å^{-3}$	

References

[1] Y. Dai, F. Wu, Z. Zang, H. You, H. Gong, *Chem. Eur. J.* **2012**, *18*, 808–812.

[2] X. Fu, J. M. Cook, *J. Org. Chem.* **1993**, *58*, 661–672.

[3] P. Y. Hayes, S. Chow, F. Rahm, P. V. Bernhardt, J. J. De Voss, W. Kitching, *J. Org. Chem.* **2010**, *75*, 6489–6501.

Author Contributions

Stephan Dohmen performed the majority of the experiments and developed the methodology. Martin Reiher contributed to data collection and to manuscript preparation. Dominik Albat performed experiments. Sema Akyol contributed to ligand synthesis. Matthias Barone and Ronald Kühne designed the ProM scaffolds. Jörg-Martin Neudörfl performed X-ray crystal structure analyses. Hans-Günther Schmalz conceived and supervised the research and wrote the manuscript.
